# Light Activated Release of Nitrile Ligands from *trans*-Ru(L)(PPh_3_)_2_(nitrile) Complexes

**DOI:** 10.1021/acsomega.4c04917

**Published:** 2024-07-25

**Authors:** Ross J. Davidson, Yu-Ting Hsu, Dmitry S. Yufit, Andrew Beeby

**Affiliations:** Department of Chemistry, Durham University, South Rd, Durham DH1 3LE, U.K.

## Abstract

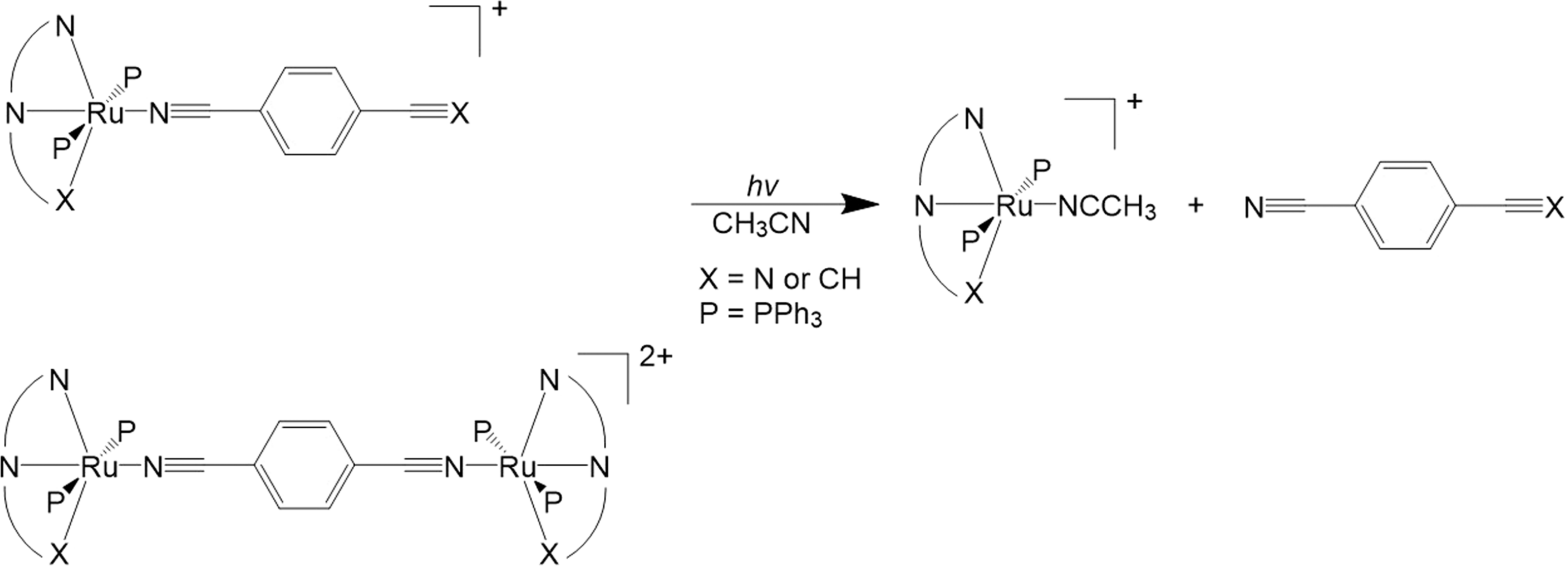

A series of *trans*-RuL(PPh_3_)_2_(nitrile) and {RuL(PPh_3_)_2_}._2_-μ-(nitrile)-based complexes [where L = 2,2′-(3,4-diphenyl-pyrrole-2,5-diyl)dipyridine (dpp), di(pyridin-2-yl)isoindoline-1,3-diimine (bpi), or 4-(4-methoxyphenyl)-6-phenyl-2,2′-bipyridine (Pbpy); and nitrile = 1,4-dibenzontirile, 4-ethynylbenzonitrile, or dicyanamide] were synthesized and characterized, and their electrochemical and photochemical behaviors were investigated. Those complexes that contained a significant nitrile contribution to their ^3^MLLCT show a release of their nitrile ligand (when L = dpp or Pbpy and the nitrile ligand = 4-dibenzontirile, or 4-ethynylbenzonitrile) with dissociation constants up to 8.09 × 10^–4^ s^–1^.

## Introduction

The ability to control the release of a ligand through the use of light has found a variety of applications including initiating catalysts and molecular logic gates,^1,2^ particularly in the field of photoactivated chemotherapy.^3,4^ In these applications, ligand liberation allows the free ligand to take part in further reactions, the loss of a ligand leaves a vacancy on the metal site making it accessible for further reactions, or it simply causes a change in the complex’s physical behavior. One common aspiration of these applications is to design systems that require a minimal amount of energy to trigger ligand release, i.e., have light absorption at a longer wavelength. This reduces the chances of unintended reactions from occurring and allows for improved tissue penetration of the light source in the case of biological applications.

This project examines what combination of ligands could form a complex with absorption in the visible range that is still capable of photoactivated ligand release. Although complexes with a range of metal ions meeting many of these criteria exist, this work focused on ruthenium-based complexes,^5^ of which some of the most prevalent are the ruthenium-nitrile family often consisting of pyridyl ligands (e.g., 2,2′-bipyridine or 2,2′:6′,2″-terpyridine) to generate ^1^MLCT bands for photon absorption. The nitrile group coordinated to the ruthenium ion can be simple (e.g., acetonitrile or benzonitrile) or a drug made inert by the metal coordination (e.g., 5-cyanouracil).^6,7^ Upon excitation, the former releases the nitrile ligand, and typically, in turn, the metal coordinates to a new ligand, often a solvent.

In this work, we explored the use of *trans*-RuL(PPh_3_)_2_(nitrile)-based complexes for photoreactivity, where L is the tridentate ligands di(pyridin-2-yl)isoindoline-1,3-diimine (bpi), 2,5-di(pyridin-2-yl)-1*H*-pyrrole, and 6-phenyl-2,2′-bipyridine. Previous work using di(pyridin-2-yl)isoindoline-1,3-diimine (bpi) has shown that the RuL(PPh_3_)_2_Cl complexes have particularly labile chlorides due to the trans-effect of the indolate,^8^ suggesting that other ligands coordinated to this site may also be similarly labile. Additionally, each of the chosen ligands has been selected to produce transition-metal complexes with high extinction coefficient ^1^MLCT bands extending well into the visible range. In principle, combining these features should result in the favorable release of the nitrile ligand.

## Results and Discussion

### Synthesis

In addition to the previously reported complexes *trans*-Ru(bpi)(PPh_3_)_2_Cl (**Clb**), two new *trans*-Ru(L)(PPh_3_)_2_Cl complexes were synthesized, where L is 2,2′-(3,4-diphenyl-pyrrole-2,5-diyl)dipyridine (dppH)^9^ or 4-(4-methoxyphenyl)-6-phenyl-2,2′-bipyridine (PbpyH).^10^ The complexes *trans*-Ru(dpp)(PPh_3_)_2_Cl (**Cla**) and *trans*-Ru(Pbpy)(PPh_3_)_2_Cl (**Clc**) were prepared by heating the respective ligand with *cis*-Ru(PPh)_3_Cl_2_ in an ethanolic solution containing the base Et_3_N. The isolation of the pure compound was dependent upon the formation of crystals. Alternate 6-phenyl-2,2′-bipyridine ligands were trialled for the synthesis of **Clc** analogues but could not be successfully purified as the most effective route for purification of these complexes is reliant on recrystallization, and it appears that the presence of a anisole group favors this. Attempts were also made to synthesize an 1,3-di(pyridin-2-yl)benzene (N∧C∧N) analogue to **Clc** under identical reaction conditions using 2,2′-(4′-methoxy-[1,1′-biphenyl]-3,5-diyl)dipyridine, but no evidence of product formation was observed.

**Figure 1:**
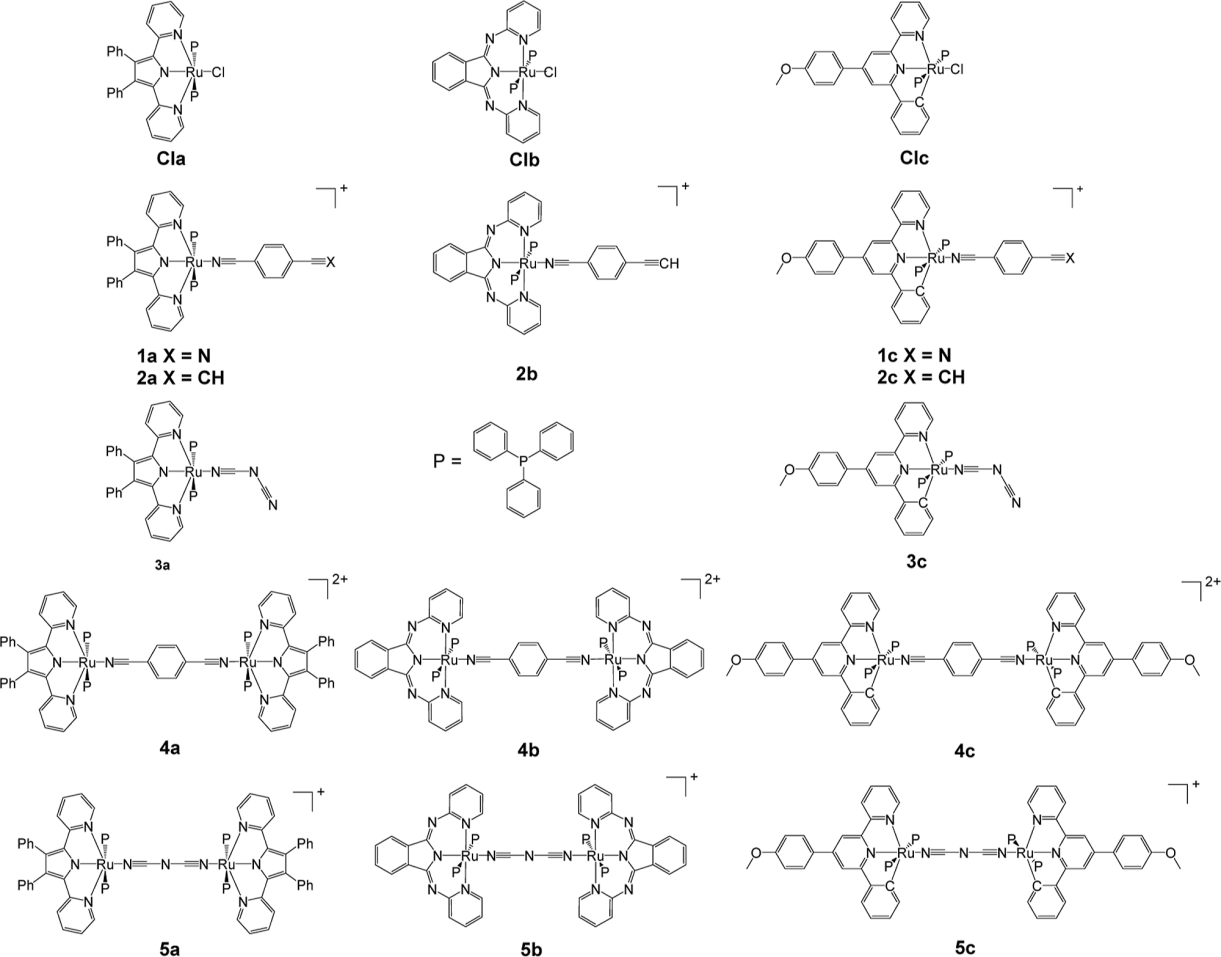
Schematic view of the complexes synthesized and used in this study.

Equipped with the precursors **Cla**, **Clb**, and **Clc**, each was reacted with either 0.5 or 5 eq of the chosen nitrile ligand (1,4-dibenzontirile, 4-ethynylbenzonitrile, or sodium dicyanamide) by heating in MeOH with NH_4_PF_6_ as the halide abstractor, similar to the conditions used by Cordiner et al.^11^ The schematic structures of all synthesized complexes are detailed in Figure 1.

For both **Cla** and **Clc**, when an excess of the nitrile ligand was used, the corresponding monometallic complexes (**1a**, **2a**, **3a**, **1c**, **2c**, and **3c**) were formed, with the nitrile ligand replacing the chloride. When 0.5 eq of the nitrile ligand was used, both 1,4-dibenzontirile and sodium dicyanamide formed bimetallic complexes (**4a**, **5a**, **4c**, and **5c**) with the nitrile acting as a bridging ligand between the two ruthenium centers, see Figure 1. However, under the same conditions, 4-ethynylbenzonitrile only formed the monometallic complexes (**2a** and **2c**) with only the nitrile group coordinating to the metal, with no evidence of vinylidene formation. The reaction was repeated with the addition of DBU as a base to promote acetylide formation from any transient vinylidenes formed, but again, no coordination via the terminal alkyne was observed. This finding suggests notably different behavior to RuCp(PPh_3_)_2_ observed by Cordiner et al.^11^

When using **Clb** with excess nitrile ligand, only 4-ethynylbenzonitrile formed a monometallic complex (**2b**), with the nitrile group coordinated to the ruthenium center, while 1,4-dibenzontirile and sodium dicyanamide both formed bimetallic complexes (**4b** and **5b**). Upon repeating the reactions with 0.5 equiv, the same results were obtained, albeit with higher yields, suggesting that the using bpi ligand resulted in the bimetallic complexes being enthalpically favorable. As observed for the previous systems, even where the reaction was performed with DBU and 4-ethynylbenzonitrile, there was no evidence of coordination via the terminal alkyne. This contrasts with the propensity for acetylide formation with Ru(bpi)(PPh_3_)_2_Cl, as observed by Zhang.^8^

#### Molecular Structures

**Figure 2:**
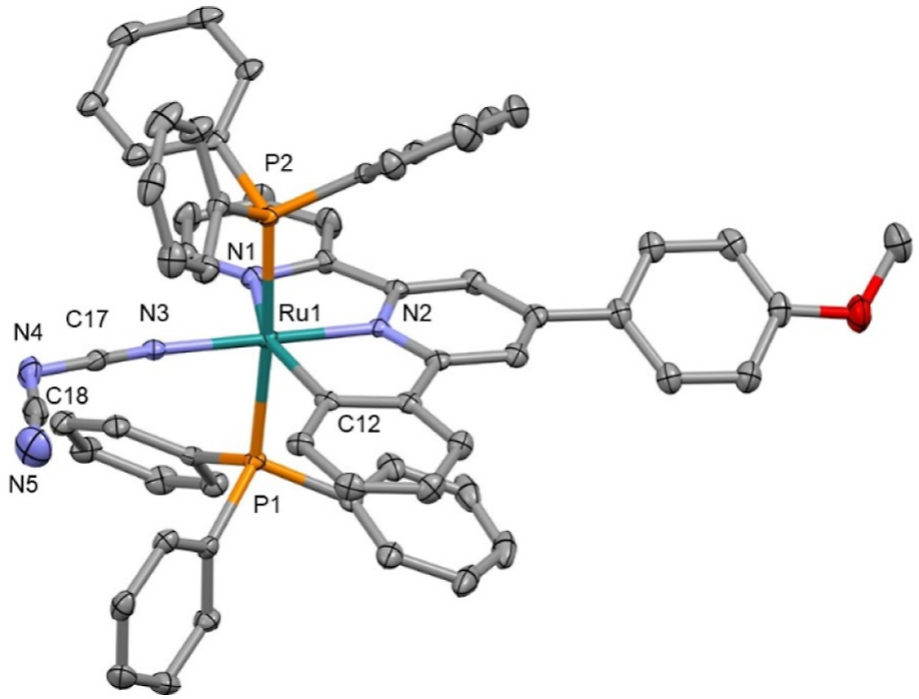
Crystal structure of **3c**; solvent molecule, disorder removed for clarity, and thermal ellipsoids displayed at 50% probability.

**Figure 3:**
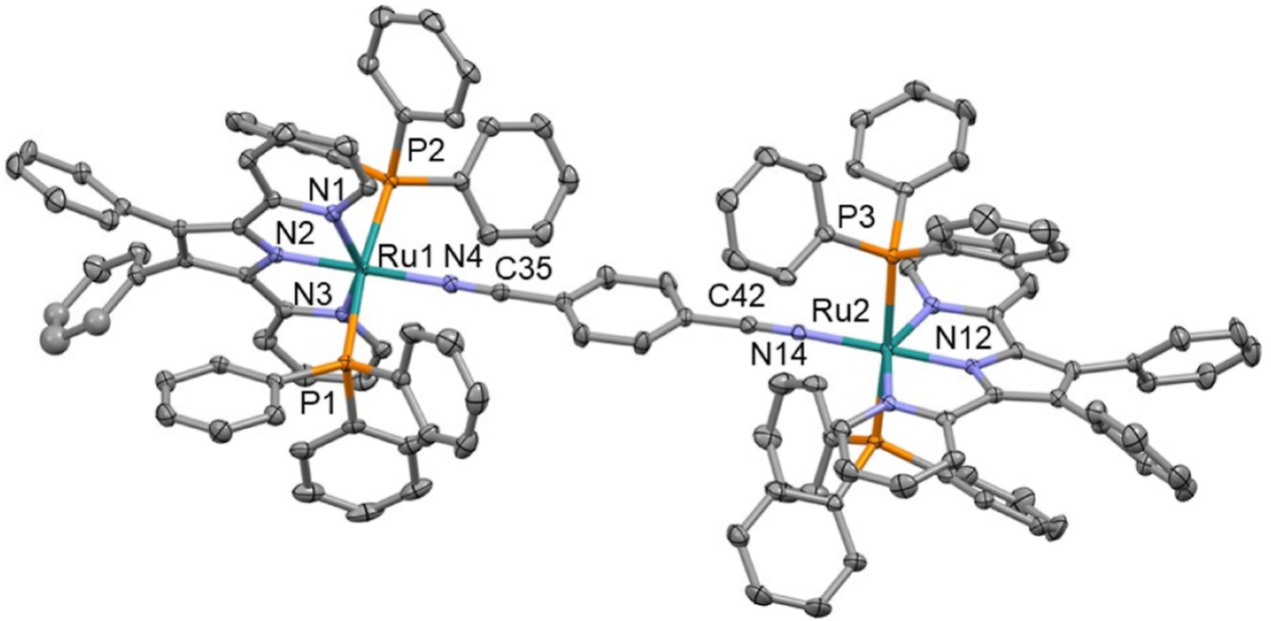
Crystal structure of **4a**; solvent molecule, disorder removed for clarity, and thermal ellipsoids displayed at 50% probability.

**Figure 4:**
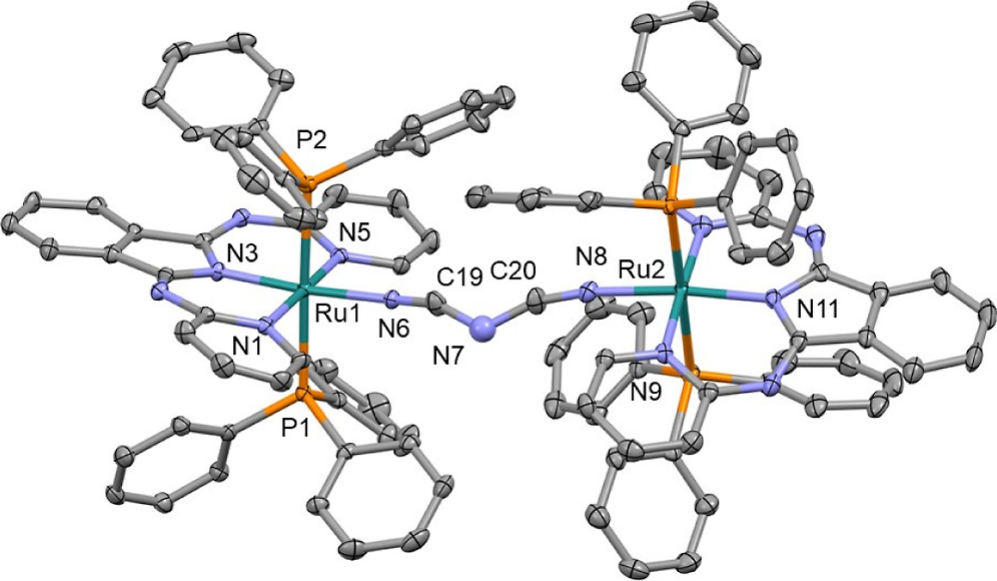
Crystal structure of **5b**; solvent molecule, disorder removed for clarity; and thermal ellipsoids displayed at 50% probability.

The structures of compounds **1a**, **2a**, **4a**, **5a**, **2b**, **4b**, **5b**, **1c**, **2c**, **3c**, **4c**, **5c**, **Cla**, and **Clc** were confirmed by SCXRD (CCDC 2205853–2205864, 2368041, and 2359149). Example structures of **3c**, **4a**, and **5b** are shown in Figures 2–4, respectively (with the remaining structures shown in the Supporting Information). In all of the studied complexes, the central Ru atom adopts slightly distorted octahedral coordination with *trans* configuration of PPh_3_ ligands. To date the CDS contains just one Ru complex, containing a heterocyclic N-coordinated ligand, a benzonitrile ligand, and two PPh_3_ groups.^12^ Neither the Ru–N_nitrile_ nor C≡N bond lengths show a variation associated with the tridentate ligand, the nitrile ligand, or a comparison between the mono- and bimetallic complexes. This behavior is due to the poor mixing of the Ru(d) and nitrile (π) orbitals.^11^ The orientation of the benzene ring connected to the nitrile group is noteworthy. In complexes with dpp and bpi ligands, this ring is almost coplanar with the plane of the heterocyclic ligand, while in complexes with the Pbpy ligand, these planes are nearly perpendicular to each other. Thus, most probably the “steric factors” between PPh_3_ and NC-C_6_H_4_ fragments are not the major contributors to the coplanar geometry. Interestingly, the central planar NCNCN fragment in **5c** is also significantly out of the plane of both chelate cycles (26.6 and 35.5°), while in **5a** and **5b**, the fragments are almost coplanar (see the Supporting Information). The chelate heterocycles of the two metal centers in **5c** are rotated by 60.9(1)°, and the corresponding values for **5a** and **5b** are 7.2(2) and 17.8(1)°, respectively. Thus, while the coordination geometry of the metal centers is similar for all of the studied complexes, the molecular geometries of the complexes with the Pbpy ligand are different from those with dpp and bpi ligands.

### Electrochemistry

Cyclic voltammograms were recorded for all the complexes in 0.1 M tetrabutylammonium hexafluorophosphate (TBAPF_6_) in dichloromethane (DCM). All the bimetallic complexes (**4a**, **4b**, **4c**, **5a**, **5b**, and **5c**) were additionally recorded in 0.1 M tetrabutylammonium tetrakis(3,5-bis(trifluoromethyl)phenyl)borate (TBArF_24_). All measurements were referenced against an internal reference of ferrocene [*E*_1/2_ Fc/Fc^+^] = 0.00 V (see Table 1). Each of the monometallic complexes **1a**, **1c**, **2a**, **2b**, **2c**, **3a**, **3c**, **Cla**, **Clb**, and **Cl**c had a single reversible oxidation event attributed to the Ru(II)/Ru(III) redox couple. Each of the complexes containing either 1,4-dibenzontirile or 4-ethynylbenzonitrile displayed a cathodic shift of 0.43–0.81 V relative to the respective parent chloride complexes **Cla**, **Clb**, and **Clc**. However, for the dicyanamide complexes (**3a** and **3c**), the cathodic shifts were only 0.25 and 0.11 V, respectively, which can be attributed to the anionic nature of dicyanamide.

**Table 1:** Electrochemical Data for the Reported Complexes

	*E*_1/2OX_ (V)		*E*_1/2OX_ (V)	
complex	TBAPF_6_	complex	TBAPF_6_	TBArF_24_
**Cla**	–0.24	**4a**	0.32, 0.35	0.43
**Clb**	0.08	**4b**	0.54	0.63
Clc	–0.51	**4c**	0.27, 0.33	0.44
**1a**	0.32	**5a**	0.07	0.09, 0.24
**1c**	0.30	**5b**	0.27	0.29, 0.48
**2a**	0.28	**5c**	–0.16, −0.01	–0.12, −0.14
**2b**	0.51			
**2c**	0.28			
**3a**	0.01			
**3c**	–0.40			

The bimetallic complexes **4a** and **4c** each showed two oxidation events, separated by 0.03 and 0.06 V, respectively, with oxidation potentials near that of monometallic analogues **1a** and **1c**, while **4b** demonstrated only a single oxidation wave. This suggests that complexes have negligible electronic coupling via the 1,4-dibenzontirile, i.e., each of the metal centers behave independently.

To examine whether any through space interactions were possible with these systems, TBArF_24_ was used as the electrolyte. Vincent et al. previously demonstrated that the use of such a large anion typically increases the separation of oxidation events linked by through-space interactions.^13,14^ However, for **4a**, **4b**, and **4c**, only a single oxidation event was observed, confirming the absence of electronic coupling between the metal centers. This is consistent with density functional theory (DFT) predicting negligible contributions to the HOMO orbitals from 1,4-dibenzontirile and in line with observations made by Cordiner et al. for analogous RuCp(PPh_3_)_2_ systems.^11^

Complexes **5a** and **5b** showed only a single-step oxidation event, **5a** having a similar oxidation potential to **3a**, indicating a lack of electronic coupling between the metal centers. **5c** showed a two-step oxidation separated by 150 mV, with the first oxidation 240 mv higher than its monometallic analogue **3c**, indicative of electronic coupling. Similar behavior has been observed by Cordiner for RuCp(PPh_3_)_2_.^11^ However, upon using the TBArF_24_ electrolyte, complexes **5a**, **5b**, and **5c** displayed two-step oxidations separated by 150, 190, and 20 mV. This indicates that these systems are capable of through-space interactions between the metal centers, which may not be surprising given the Ru–Ru distance of ca. 8.5 Å. Unfortunately, the oxidized complexes proved to be insufficiently stable for spectro-electrochemical measurement to be able to confirm the nature of the coupling, but the results are consistent with the low presence of dicyanamide character in the HOMOs described by DFT.

### Electronic Absorbance

The UV–visible spectra of each of the complexes was recorded in acetonitrile and assigned using TD-DFT (see the Supporting Information for details). Each of the complexes displayed π → π* transitions, λ < 330 nm and CT λ = 300–650 nm. For the bpi complexes (**2b**, **4b**, and **5b**), the CT bands were attributed to ^1^MLCT transitions, with the relevant HOMO orbitals consisting of ruthenium(d) character and LUMOs being significantly bpi in character. The CT transitions of the dicyanamide complexes (**3a**, **3c**, **4a**, and **4c**) are assigned to be ^1^MLLCT, with the relevant HOMOs containing varied contributions from ruthenium(d), the tridentate ligand (π), and dicyanamide(π), while the LUMOs contain negligible contribution from the dicyanamide. For **3a** and **5a**, the triphenylphosphine(π*) orbitals contribute, while for **3c** and **5c**, the LUMOs are dominated by the bipyridine(π*) orbitals.

The dpp complexes with 1,4-dibenzontirile or 4-ethynylbenzonitrile (**1a**, **2a**, and **4a**) show similar CT bands for both the mono- and bimetallic analogues, with the most significant difference being that **4a** has a ε approximately twice that of **1a** and **2a**, a result of orbital degeneracy. The CT band was determined to contain both ^1^MLCT and ^1^MLLCT transitions, with the relevant HOMOs containing varied combinations of ruthenium and pyrrole character, while the LUMOs were dominated by either 1,4-dibenzontirile or 4-ethynylbenzonitrile character.

**Figure 5:**
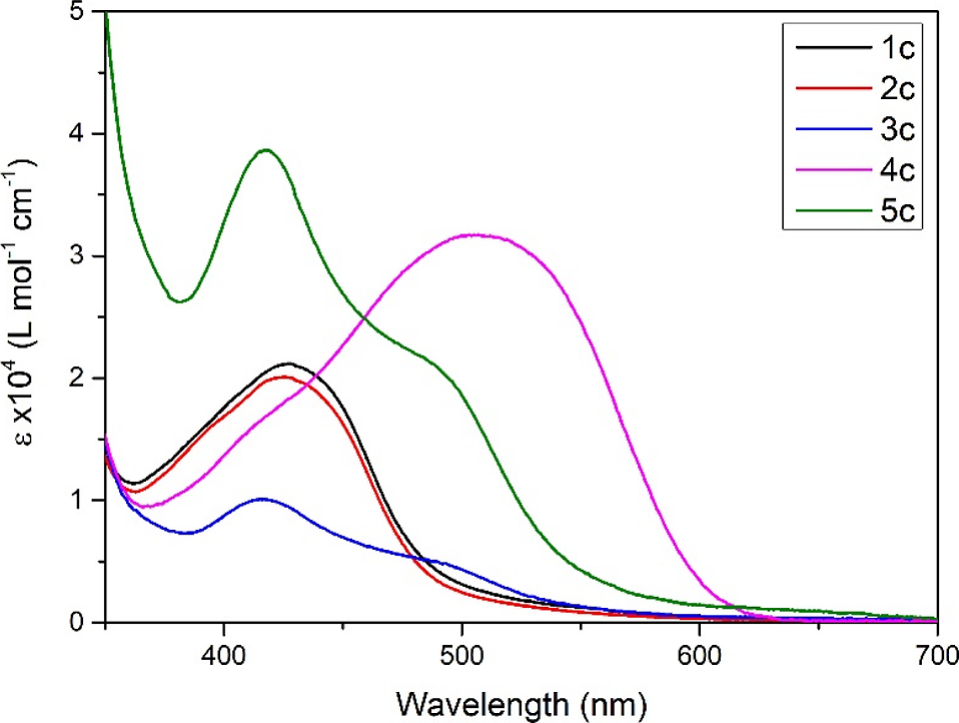
UV–visible data for complexes **1c**, **2c**, **3c**, **4c**, and **5c** were recorded in acetonitrile.

The CT absorbance bands of the Pbpy **1c** and **2c** display little difference with a λ_max_ = 428 nm; however, the bimetallic complex **4c** had both the enhanced ε observed for the other complexes but also showed a significant red-shift of the CT band to λ_max_ = 507 nm (see Figure 5). While the CT band can be assigned to ^1^MLLCT for both mono- and bimetallic complexes, the orbital contributions are different. The relevant HOMOs for both sets of complexes are both ruthenium and phenylate in nature. The LUMOs of **1c** and **2c** contain a mixture of contributions from the bipyridine and nitrile ligand, while **4c** is exclusively localized to the 1,4-dibenzontirile (π*) orbitals.

#### Photodissociation

**Figure 6:**
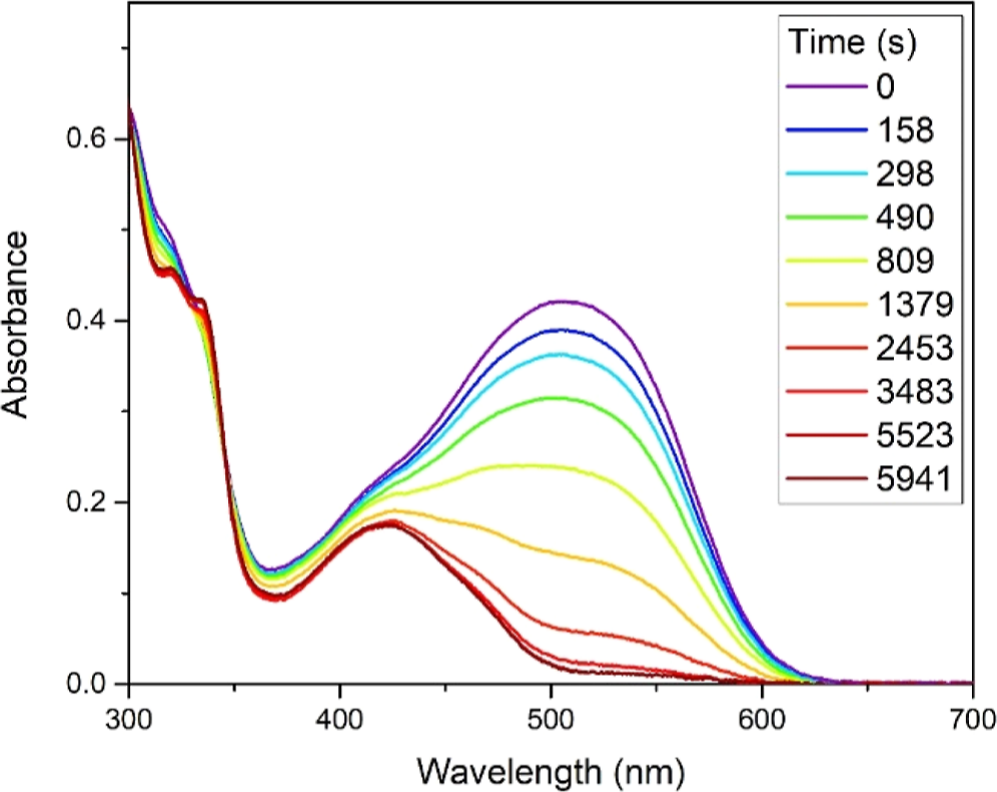
UV–visible spectra of complex **4c** irradiated with light at specified times.

Based upon the work of Zhao, Respondek, and Sgambellone et al.,^6,15,16^ when some ruthenium nitrile complexes are irradiated by visible light, the Ru–N_nitrile_ bond dissociates releasing the free nitrile ligand. To examine the photodissociation behavior of the complexes, they were irradiated. To this end, solutions containing the respective complex in 0.1 M tetrabutylammonium chloride (TBACl) in anhydrous acetonitrile were degassed by three freeze–pump–thaw cycles. Each of the solutions were irradiated by a xenon lamp (300 W) with an UV cutoff filter. At the specified intervals, the UV–visible spectra of the solution were recorded. The dpp and Pbpy complexes with 1,4-dibenzontirile and 4-ethynylbenzonitrile (**1a**, **2a**, **1c**, **2c**, **4a**, and **4c**) displayed a significant decrease in the ^1^MLLCT band associated with the nitrile ligands (see example Figure 6), fitting the behavior reported by Respondek et al. for their Ru(2,2′-bpyridine)_2_(nitrile)_2_ system.^16^ By measuring the change in absorbance of these transitions, the dissociation constant (*k*_dis_) was determined to be 2.3–8.0 × 10^–4^ s^–1^ (summarized in Table 2), notably higher than that of Respondek’s system *k*_dis_ = 2.3–2.8 × 10^–7^ s^–1^.

**Table 2:** Ru–N_nitrile_ Bond Dissociation Constants for Complexes **1a**, **2a**, **4a**, **1c**, **2c**, and **4c**

complex	*k*_dis_ (s^–1^) × 10^–4^
**1a**	2.64
**2a**	8.09
**4a**	6.77
**1c**	2.33
**2c**	5.63
**4c**	7.80

The bpi complexes (**2b**, **4b**, and **5b**) displayed a decrease in absorbance in the region λ < 450 nm and an increase in the region λ > 600 nm. Based on comparisons to the work of Zhang et al., this is more consistent with generation of a Ru(III) species, rather than the release of the nitrile ligand.^8^ The dicyanamide complexes (**3a**, **3b**, **5a**, **5b**, and **5c**) displayed negligible differences, even after having been irradiated for over 1 h.

**Figure 7:**
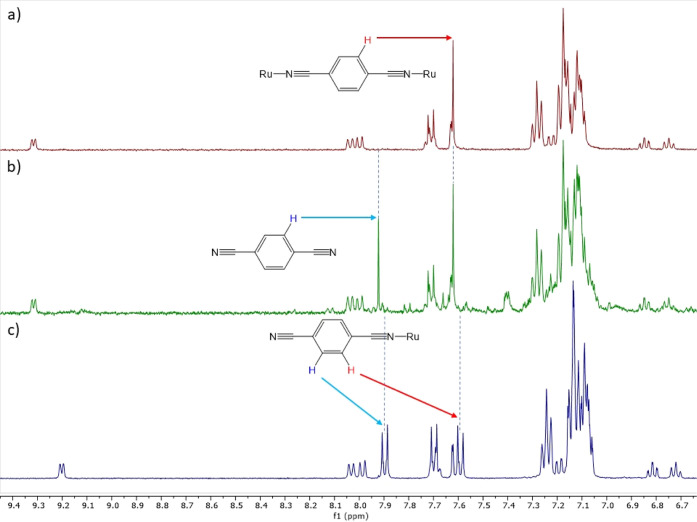
^1^H NMR spectra of **4c**; (a) before irradiation, (b) after irradiation for 60 s, and (c) **1c** as a reference.

To further elucidate the behavior of the photodissociation and NMR study of the complexes **1a**, **2a**, **4a**, **1c**, **2c**, and **4c**, the photodissociation experiments were repeated in a solution of 0.1 M TBACl in CD_3_CN. For each complex, the “free” nitrile was observed; however, for the bimetallic complexes, **4a** and **4c** (see example Figure 7) were observed, no evidence of the monometallic analogues **1a** and **1c** was observed. This indicates that upon irradiation, both Ru–N_nitrile_ bonds dissociate simultaneously, which may account for the similar dissociation constants between the band monometallic complexes. Despite being performed in a TABCl solution, no signals relating to the chloride precursors (**Cla** and **Clc**) were observed rather it is most likely that the solvent (acetonitrile) occupying the vacant coordination site as Sgambellone et al. has observed.^6^ In order to compare this, a TD-DFT comparison was made between the observed spectra and the corresponding acetonitrile complexes (CH_3_CN-a and CH_3_CN). The calculated spectra provide a reasonable fit for the irradiation product (see the Supporting Information). However, upon further irradiation, an intractable mixture of species is formed, making it impossible to isolate the proposed acetonitrile complexes and suggests that they may not be stable.

#### DFT Calculations

**Figure 8:**
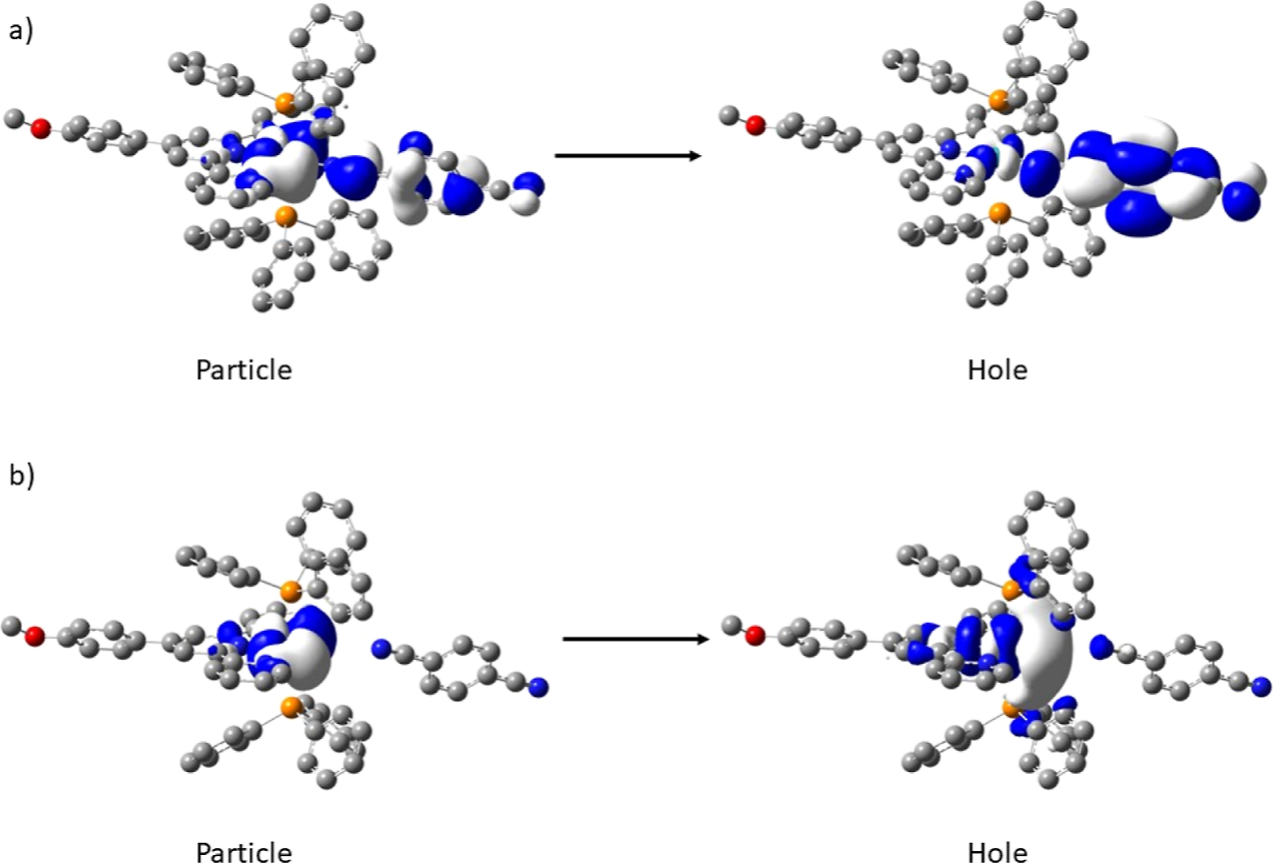
Plot of natural transition orbitals of **1c** for S_0_ → T_1_ for Ru–N_nitrile_ length: (a) 2.08 and (b) 3.80 Å.

To provide insights for the behavior of the complexes, calculations were performed to establish the energy associated with the nitrile dissociation. To this end, the a scan calculation was performed along the Ru–N_nitirile_ bond sequentially increasing the distance until a length of 4 Å was achieved, a distance greater than the sum of Van der Waal radii of ruthenium and nitrogen (see the Supporting Information). Using this approach, we can observe that the energy difference ranges from 58.19 (**4b**) to 105.15 (**3a**) kJ mol^–1^, but there is no correlation between those that dissociate and the energy difference. Tu et al. determined that the Ru–N_nitrile_ bond dissociation is facilitated by the ^3^MLCT states.^17^ The S_0_ → T_*n*_ transitions were determined by using TD-DFT to calculate the energies of the lowest triplet states (see the Supporting Information). Although each of the transitions was determined to be CT in nature, no correlation between the T_1_ energy and *k*_dis_ was determined. However, both the T_1–3_ states of the bpi and dicyanamide complexes were dominated by the tridentate ligand π* orbitals, while the same triplet states for complexes **1a**, **2a**, **4a**, **1c**, **2c**, and **4c** were dominated by the respective nitrile ligand π* orbitals, indicating that this is a requirement for the dissociation to occur. This was further examined by following the change in behavior for the T_1_ transition as the ruthenium nitrile bond length was increased. This demonstrated a significant change in the T_1_ energy for **1a**, **2a**, **4a**, **1c**, **2c**, and **4c** as the length was increased coupled with the character of the transition changing from that of CT (involving the nitrile ligand) to metal centered (see Figure 8 and Supporting Information). However, the cyanamide and bpi complexes (**2b**, **4b**, **5a**, **5b**, and **5c**) each only displayed a modest change in energy, and the CT transition remained localized to the tridentate ligand system.

## Conclusions

A series of mono- and bimetallic *trans*-RuL(PPh_3_)_2_(nitrile) complexes (where L = dpp, bpi, or Pbpy and nitrile = dicyanamide, 1,4-dibenzontirile or 4-ethynylbenzonitrile) were synthesized and characterized including X-ray crystallography. Through the use of electrochemistry, the metal centers of the bimetallic complexes were proven to be electronically independent, regardless of the tridentate ligand or nitrile used.

By studying the photodissociation behavior of this series, the crucial role of the ligand character in the triplet states was demonstrated. This shows that for effective photodissociation to occur, the triplet states must contain significant contributions from the nitrile ligand and that this can be regulated by both the choice of nitrile and complementary ligands in the complex. Due to the stability of these complexes in the dark, rapid dissociation, and modular construction, these systems will provide a useful construct for examining photoactivated ligand dissociation.

## Materials and Methods

### Instrumentation

Nuclear magnetic resonance (NMR) spectra were recorded in deuterated solvent solutions on a VNMRS-600 and VNMRS-700 spectrometer (Varian Technologies, Palo Alto, CA, USA) and were referenced against solvent resonances (^1^H, ^13^C{^1^H}, ^31^P{^1^H}), compressive sampling for all unstable compounds.^6−8^ Electrospray mass spectra data were recorded on a TQD mass spectrometer (Waters Ltd., UK) in acetonitrile; MALDI–TOF MS data were recorded on an Autoflex II TOF/TOF (Bruker Daltonics, Inc., Billerica, MA, USA), using a DCTB as the matrix. Vibrational data were recorded by using a PerkinElmer Spectrum Two FT-IT spectrometer. Microanalyses were performed by the Elemental Analysis Service, London Metropolitan University, UK or Elemental Microanalysis Service, Durham University, UK.

### General Details

The compounds 4-(4-methoxyphenyl)-6-phenyl-2,2′-bipyridine (PbpyH),^10^*cis*-Ru(PPh)_3_Cl_2_,^18^ 2,2′-(3,4-diphenyl-1*H*-pyrrole-2,5-diyl)dipyridine (Hdpp),^9^*tran*s-Ru(bpi)(PPh_3_)_2_Cl^8^, and 4-ethynylbenzonitrile^19^ were prepared according to literature methods. All other chemicals were sourced from standard chemical suppliers.

### Synthesis

#### Ru(dpp)(PPh_3_)_2_Cl (**Cla**)

*Cis*-Ru(PPh_3_)_3_Cl_2_ (1.28 g, 1.34 mmol) and Hdpp (0.5 g, 1.34 mmol) were added to ethanol (100 mL) and refluxed for 3 h before Et_3_N (20 mL) was added to the solution, and refluxing continued for an additional hour. The solution was cooled, forming a precipitate that was collected via filtration and thoroughly diluted with MeOH. The solid was purified via column silica chromatography eluted with neat DCM, collecting the orange band. The solvent was removed, leaving a solid that was washed with MeOH a final time, leaving a bright orange powder. Crystals were grown by layering MeOH onto a DCM solution. **Yield**: 1.14 g (80%). ^**1**^**H NMR** (700 MHz; CD_2_Cl_2_): δ_H_ 8.09 (dd, ^3^*J*_HH_ = 5.7 Hz, ^4^*J*_HH_ = 1.7 Hz, 2H, H_a_), 7.29–7.22 (m, 24H, H_f_ + H_g_ + H_h_ + H_j_), 7.12 (t, ^3^*J*_HH_ = 7.67 Hz, 12H, H_i_), 6.97 (d, ^3^*J*_HH_ = 7.4 Hz, 4H, H_e_), 6.93 (ddd, ^3^*J*_HH_ = 8.5 Hz, ^3^*J*_HH_ = 7.2 Hz, ^4^*J*_HH_ = 1.6 Hz, 2H, H_c_), 6.75 (dd, ^3^*J*_HH_ = 8.0 Hz, ^4^*J*_HH_ = 1.4 Hz, 2H, H_d_), 6.25 (ddd, ^3^*J*_HH_ = 7.1 Hz, ^3^*J*_HH_ = 5.5 Hz, ^4^*J*_HH_ = 1.4 Hz, 2H, H_b_) ppm. ^**13**^**C{**^**1**^**H} NMR** (176 MHz; CD_2_Cl_2_): δ_C_ 159.5, 157.5, 135.9, 135.4, 134.8, 133.9, 133.2, 132.1, 131.5, 128.7, 127.7, 127.4, 125.6, 119.3, 115.8 ppm. ^**31**^**P{**^**1**^**H} NMR** (283 MHz; CD_2_Cl_2_): δ_P_ 23.3 (s) ppm. **Anal. Calc.** for C_62_H_48_ClN_3_P_2_Ru: C, 72.05; H, 4.68; N, 4.07%. **Found**: C, 71.93; H, 4.61; N, 4.14%.^a^

#### *tran*s-Ru(PBpy)(PPh_3_)_2_Cl (**Clc**)

A suspension of PbpyH (1.00 g, 2.95 mmol), Ru(PPh_3_)_3_Cl_2_ (2.83 g, 2.95 mmol), and Et_3_N (10 mL) in ethanol (150 mL) was refluxed under nitrogen for 16 h. The solvent was removed under vacuo, and the residue was passed through a silica plug, the red fraction was collected, and the solvent was removed. The solid was dissolved in a minimal amount of DCM, and then a layer of methanol (MeOH) was added before being refrigerated overnight, producing dark red crystals that were collected by filtration. **Yield**: 702 mg (23%).^b^**MS** (MALDI): *m*/*z* 998.3 [M]^+^, 736.1 [M-PPh_3_]^+^. **Anal. Calc.** for C_59_H_47_ClN_2_OP_2_Ru: C, 70.97; H, 4.74; N, 2.81%. **Found**: C, 70.66; H, 4.67; N, 2.73%.

#### *tran*s-[Ru(L)(PPh_3_)_2_(NC-R)]^*n*+^ General Synthesis

A suspension of *tran*s-Ru(L)(PPh_3_)_2_Cl (1 equiv), nitrile (5 equiv), and NH_4_PF_6_ (5 equiv) in MeOH (40 mL) was refluxed for 12 h. The solution was cooled to room temperature, and the precipitate was collected by filtration and washed thoroughly with MeOH. Final purification was achieved by crystallization. Full synthetic and characterization details are given in the Supporting Information.

#### [{*trans*-Ru(L)(PPh_3_)_2_}_2_(μ-NC-R-CN)]^*n*+^ General Synthesis

A suspension of *trans*-Ru(L)(PPh_3_)_2_Cl (1 equiv), NC-R-CN (0.5 equiv), and NH_4_PF_6_ (5 equiv) in MeOH (40 mL) was refluxed for 12 h. After cooling to room temperature, the precipitate was collected by filtration and thoroughly washed with MeOH. Final purification was achieved by crystallization. Full synthetic and characterization details are given in the Supporting Information.

### Electrochemistry

Electrochemical analyses of the ruthenium complexes were carried out using a PalmSens EmStat^2^ potentiometer, with platinum working, platinum counter, and platinum pseudo reference electrodes, from solutions in acetonitrile containing 0.1 M supporting electrolyte (tetrabutylammonium hexafluorophosphate, TBAPF_6_). All of the bimetallic complexes (**4a**, **4b**, **4c**, **5a**, **5b**, and **5c**) were additionally recorded in 0.1 M tetrabutylammonium tetrakis(3,5-bis(trifluoromethyl)phenyl)borate (TBArF_24_). The ferrocene/ferrocenium couple was used as the internal reference.

### Electronic Absorbance

The UV–visible spectra were obtained using an Unicam UV2–100 spectrometer operated with Unicam Vison software in quartz cuvettes with path length *l* = 1 cm. Solutions of the respective complexes in acetonitrile were prepared in the absence of light.

### Photodissociation

To monitor the photodissociation of the complexes by electronic absorbance, a solution of the respective complex was prepared with 0.1 M TBACl in anhydrous acetonitrile and degassed by three freeze–pump–thaw cycles. Young’s tap-modified quartz cuvettes with path length *l* = 1 cm were used for both irradiation and UV–visible absorbance measurements. Each of the solutions was irradiated by a xenon lamp (300 W) with an UV cutoff filter. At the specified intervals, the UV–visible spectrum of the solution was recorded by using an Unicam UV2–100 spectrometer operated with Unicam Vison software.

To monitor the photodissociation of the complexes by NMR, a solution of the respective complex was prepared with 0.1 M TBACl in CD_3_CN and degassed by three freeze–pump–thaw cycles. Young’s NMR tubes were used for both irradiation and UV–visible absorbance measurements. Each of the solutions was irradiated by a xenon lamp (300 W) with an UV cutoff filter. At the specified intervals, the ^1^H and ^31^P NMR spectra of the solution were recorded using a Varian VNMRS-600 spectrometer and referenced against solvent resonances.^c^

### DFT Calculations

DFT calculations were carried out using the Gaussian 09 package (Gaussian, Inc.).^20^ All results were displayed using GaussView^21^ and GaussSum.^22^ All calculations used the B3LYP level set employing a 6-31G(d)/LANL2DZ basis set, geometrically optimized in a acetonitrile solvent field using the SCRF-PCM method, where available initial structures were based on crystallographic data. Frequency calculations were performed on each of the structures to confirm that a minimum has been reached. TD-DFT calculations were performed on the geometrically optimized structures using the same method.

## Supporting Information Available

Experimental procedures, NMR spectra, photophysical data, and computational details (PDF)
Crystallographic data (CIF)
DFT optimized structures (XYZ)

## Supplementary Material

ao4c04917_si_001.pdf

ao4c04917_si_002.cif

ao4c04917_si_003.xyz

## Footnotes

a No identifiable ions were observed using MALDI or ES.

b Recorded NMR spectra gave broad unresolved signals (see Figure S4a,b) on both 400 and 600 MHz NMR indicating ligand (likely PPh_3_) dissociation in solution and making signal assignment impossible.

c Complex **4a′**s low solubility in CD_3_CN gave a poor signal-to-noise ratio, preventing the measurement.
