# Supplementary material for: Light Activated Release of Nitrile Ligands from trans-Ru(L)(PPh3)2(nitrile) Complexes
Source: ACS Omega. 2024 Jul 25;9(31):34098–105. doi: 10.1021/acsomega.4c04917 (PMC11307298; doi:10.1021/acsomega.4c04917)
Supplement: Supplementary file 1 — ao4c04917_si_001.pdf [file ao4c04917_si_001.pdf]

SUPPORTING INFORMATION

Light activated release of nitrile ligands from of *trans*-Ru(L)(PPh_3)$_2$(nitrile) complexes

Ross J. Davidson,[a*] Yu-Ting Hsu,[a] Dmitry S. Yufit,[a] Andrew Beeby,[a*]

a) Department of Chemistry, Durham University, South Rd, Durham, DH1 3LE, UK

Table of Contents

S1. Synthesis of reported compounds …………………………………. S3

S2. NMR spectra of reported compounds …………………………... S13

S3. Crystallographic data ……………………………..……………….. S49

S4. Physical Measurements …………………………………………… S61

S5. DFT Calculations ..………………………….……………..…….. S93

S6. Photodissociation Products ……………………………………… S129

References ……………………………………………………………... S137

S1. Synthesis of reported compounds

***trans*-[Ru(L)(PPh_3)$_2$(NC-R)]$^{n+}$ general synthesis.** A suspension of *trans*-Ru(L)(PPh_3)$_2$Cl (1 eq), nitrile (5 eq) and NH_4PF_6 (5 eq) in MeOH (40 mL) was refluxed for 12 hours. The solution was cooled to room temperature and the precipitate was collected by filtration and washed thoroughly with MeOH. Final purification was achieved by crystallisation.

[*trans*-Ru(dpp)(PPh_3)$_2$(NC-C_6H_4-CN)](PF_6) (**1a**). Crystallisation was achieved by vapour diffusion of Et_2O into an acetone solution to yield orange crystals. **Yield**: 100 mg (41 %). **^{1}H NMR** (600 MHz; CD_3CN): δ_H 8.21 (dd, $^3J_{HH}$ = 5.6 Hz, $^4J_{HH}$ = 1.6 Hz, 2H, H_a), 7.87 (d $^3J_{HH}$ = 7.9 Hz, 2H, H_l), 7.36-7.33 (m, 8H, H_e+H_k), 7.31-7.28 (m, 4H, H_f), 7.26-7.24 (m, 14H, H_g+H_i), 7.13-7.08 (m, 16H, H_e+H_h), 7.04 (t, $^3J_{HH}$ = 7.9 Hz, 2H, H_c), 6.58 (dd, $^3J_{HH}$ = 8.0 Hz, $^4J_{HH}$ = 1.1 Hz, 2H, H_d), 6.43 (td, $^3J_{HH}$ = 7.2 Hz, $^4J_{HH}$ = 1.4 Hz, 2H, H_b) ppm. **^{13}C{^{1}H} NMR** (150 MHz; CD_3CN): δ_C 158.9, 157.1, 136.3, 135.8, 135.2, 133.2 (t, $^3J_{CP}$ = 5.4 Hz) 132.9 (t, $^3J_{CP}$ = 4.2 Hz), 130.5, 130.1 (t, $^2J_{CP}$ = 9.3 Hz), 129.9, 129.8, 128.4 (t, $^3J_{CP}$ = 4.5 Hz), 128.2, 128.0, 126.3, 120.2, 116.9, 116.5, 115.7 ppm. **^{31}P{^{1}H} NMR** (242 MHz; CD_3CN): δ_P 29.1 (s, 2P), -144.4 (sept, $^2J_{PF}$ = 707 Hz, 1P) ppm. **MS**(MALDI): m/z 998.5 [M-$C_8H_4N_2$-PF_6]$^+$. **Anal. Calc. for** $C_{70}H_{52}F_6N_5P_3Ru$: C, 66.14; H, 4.12; N, 5.51 %. **Found**: C, 66.52; H, 4.26; N, 5.43 %. **IR** (C≡N) ν 2199 cm^{-1}.

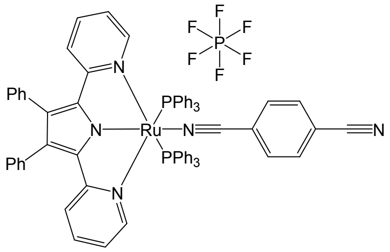

[*trans*-Ru(PBpy)(PPh_3)$_2$(NC-C_6H_4-CN)](PF_6) (**1c**). Crystallisation was achieved by layering of MeOH onto a DCM solution of the complex to yield orange crystals. **Yield**: 36 mg (42%). **^{1}H NMR** (600 MHz; $CDCl_3$): δ_H 9.30 (d, $^3J_{HH}$ = 5.0 Hz, 1H, H_a), 7.72-7.67 (m, 4H, H_d+H_g+H_q), 7.55 (d, $^3J_{HH}$ = 8.0 Hz, 2H, H_k), 7.51 (td, $^3J_{HH}$ = 7.6 Hz, $^4J_{HH}$ =1.4 Hz, 1H, H_c), 7.46 (d, $^4J_{HH}$ =1.5 Hz, 1H, H_e), 7.36 (d, $^3J_{HH}$ = 8.0 Hz, 2H, H_r), 7.30 (ddd, $^3J_{HH}$ = 7.1 Hz, $^3J_{HH}$ = 5.4 Hz, $^4J_{HH}$ = 1.2 Hz, 1H, H_b), 7.17 (t, $^3J_{HH}$ = 7.4 Hz, 6H, H_p), 7.11-7.06 (m, 14H, H_L+H_o), 7.03-7.02 (m, 2H, H_f+H_j), 6.99-6.97 (m, 12H, H_n), 6.84 (t, $^3J_{HH}$ = 7.3 Hz, 1H, H_h), 6.72 (t, $^3J_{HH}$ = 7.3 Hz, 1H, H_i), 3.93 (s, 3H, H_m) ppm. **^{13}C{^{1}H} NMR** (150 MHz; $CDCl_3$): δ_C 164.8, 160.9, 155.9, 155.3, 154.7, 147.0, 146.2, 142.6, 136.5, 132.9 (t, $^3J_{CP}$ = 5.2 Hz), 132.7, 132.5, 130.5 (t, $^2J_{CP}$ =19.8 Hz), 129.9, 128.3, 128.0 (t, $^3J_{CP}$ = 4.5 Hz), 127.0, 123.4, 120.6, 116.9, 115.8, 115.1, 114.8, 55.4 ppm. **^{31}P{^{1}H} NMR** (242 MHz; $CDCl_3$): δ_P 31.4 (s, 2P), -144.4 (sept, $^2J_{PF}$ =707 Hz, 1P) ppm. **MS**(MALDI): m/z 965.1 [M-$C_8H_4N_2$-PF_6]$^+$.**Anal. Calc.** for $C_{67}H_{51}F_6N_4OP_3Ru$: C, 65.10; H, 4.16; N, 4.53 %. **Found**: C, 65.06; H, 4.12; N, 4.52 %. **IR** (C≡N) ν 2187 cm^{-1}.

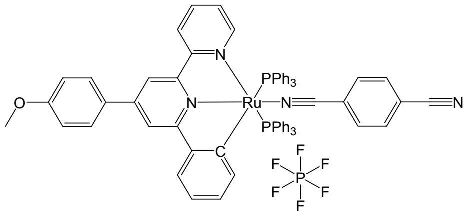

[*trans*-Ru(dpp)(PPh_3)$_2$(NC-C_6H_4-CCH)](PF_6) (**2a**). Crystallisation was achieved by layering of MeOH onto a DCM solution of the complex to yield orange crystals. **Yield**: 125 mg (51%). **^{1}H NMR** (600 MHz; TCE-d$_2$): δ_H 7.72 (d, $^3J_{HH}$ =6.6 Hz, 2H, H_a), 7.59 (d, $^3J_{HH}$ = 8.3 Hz, 2H, H_k), 7.35-7.25 (m, 6H, H_j), 7.32-7.30 (m, 4H, H_f), 7.28-7.22 (m, 14H, H_g+H_i), 7.04-6.98 (m, 18H, H_c+H_e+H_h), 6.93 (d, $^3J_{HH}$ = 7.2 Hz, 2H, H_l), 6.76 (d, $^3J_{HH}$ = 8.0 Hz, 2H, H_d), 6.37 (td, $^3J_{HH}$ = 7.2 Hz, $^4J_{HH}$ =5.7 Hz, 2H, H_b), 3.47 (s, 1H, H_m) ppm. **^{13}C{^{1}H} NMR** (150

MHz; TCE-d_2): δ_C 159.1, 155.5, 136.3, 136.1, 134.8, 133.2, 133.1 (t, $^3J_{CP}$ = 5.5 Hz), 131.8, 130.43, 130.4, 129.9 (t, $^2J_{CP}$ = 19.6 Hz), 128.7, 128.5 (t, $^3J_{CP}$ = 5.0 Hz), 128.3, 127.4, 126.4, 120.3, 117.5, 111.2, 83.3, 81.8 ppm. **^{31}P{^{1}H} NMR** (242 MHz; TCE-d_2): δ_P 28.7 (s, 2P), -144.4 (sept, $^2J_{PF}$ = 707 Hz, 1P) ppm. **MS**(MALDI): m/z 965.1 [M- C_9H_5N-PF_6]$^+$. **Anal. Calc.** for $C_{71}H_{53}F_6N_4P_3Ru$: C, 67.14; H, 4.21; N, 4.41 %. **Found**: C, 67.00; H, 4.14; N, 4.39 %. **IR** (C≡N) ν 2205 cm^{-1}.

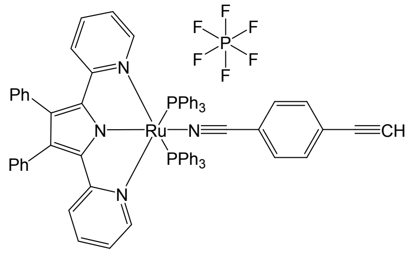

[*trans*-Ru(BPI)(PPh_3)$_2$(NC-C_6H_4-CCH)](PF_6) (**2b**). Crystallisation was achieved by layering of MeOH onto a DCM solution of the complex to yield purple crystals. **Yield**:152 mg (61%). **^{1}H NMR** (600 MHz; acetone-d_6): δ_H 8.93 (d, $^3J_{HH}$ = 7.3 Hz, 2H, H_a), 7.77 (dd, $^3J_{HH}$ = 5.6 Hz, $^3J_{HH}$ = 5.6 Hz, 2H, H_e), 7.74 (td, $^3J_{HH}$ = 7.9 Hz, $^4J_{HH}$ = 2.3 Hz, 2H, H_c), 7.66 (d, $^3J_{HH}$ = 8.5 Hz, 2H, H_j), 7.58 (dd, $^3J_{HH}$ = 5.6 Hz, $^3J_{HH}$ = 5.6 Hz, 2H, H_f), 7.43 (d, $^3J_{HH}$ = 8.5 Hz, 2H, H_k), 7.35 (d, $^3J_{HH}$ = 9.6 Hz, 2H, H_d), 7.24 (t, $^3J_{HH}$ = 8.4 Hz, 6H, H_i), 7.02 (t, $^3J_{HH}$ = 8.4 Hz, 12H, H_h), 6.83 (t, $^3J_{HH}$ = 7.5 Hz, 2H, H_b), 6.77-6.75 (m, 12H, H_g), 4.15 (s, 1H, H_l) ppm. **^{13}C{^{1}H} NMR** (150 MHz; acetone-d_6): δ_C 157.6, 154.6, 153.9, 140.7, 136.5, 132.8 (t, $^3J_{CP}$ = 5.6 Hz), 132.7, 132.5, 129.9, 129.6, 129.2 (t, $^2J_{CP}$ = 17.8 Hz), 128.9, 128.2 (t, $^3J_{CP}$ = 4.5 Hz), 127.7, 120.6, 118.8, 111.3, 83.9, 81.6, 54.0 ppm. **^{31}P{^{1}H} NMR** (242 MHz; TCE-d_2): δ_P 26.4 (s, 2P), -144.4 (sept, $^2J_{PF}$ = 757Hz, 1P) ppm.[i] Anal. Calc. for $C_{63}H_{47}F_6N_6P_3Ru\cdot½CH_2Cl_2$: C, 61.58; H, 3.91; N, 6.79 %. Found: C, 61.79; H, 3.94; N, 6.86 %. **IR** (C≡N) ν 2214 cm^{-1}.

[i] Using MALDI no peaks corresponding to the parent ion or fragments could be identified.

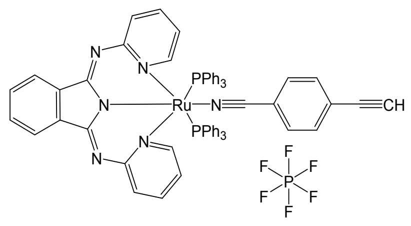

[*trans*-Ru(PBpy)(PPh_3)$_2$(NC-C_6H_4-CCH)](PF_6) (**2c**). Crystallisation was achieved by layering of MeOH onto a DCM solution of the complex to yield orange crystals. **Yield**: 82 mg (66%). **^{1}H NMR** (600 MHz; Acetone-d$_6$): δ_H 9.30 (dd, $^3J_{HH}$ = 5.4 Hz, $^4J_{HH}$ = 1.3 Hz, 1H, H_a), 8.20 (d, $^3J_{HH}$ = 7.3 Hz, 1H, H_d), 8.06 (d, $^3J_{HH}$ = 7.3 Hz, 1H, H_g), 7.91 (d, $^4J_{HH}$ = 1.6 Hz, 1H, H_e), 7.80-7.75 (m, 3H, H_c+H_k), 7.68 (d, $^3J_{HH}$ = 8.0 Hz, 2H, H_q), 7.48 (d, $^3J_{HH}$ = 8.0 Hz, 2H, H_r), 7.31 (d, $^4J_{HH}$ = 1.5 Hz, 1H, H_f), 7.27-7.23 (m, 7H, H_j+H_p), 7.22-7.20 (m, 1H, H_b), 7.16-7.12 (m, 26H, H_l+H_n+H_o), 6.82 (td, $^3J_{HH}$ = 7.3 Hz, $^4J_{HH}$ = 1.4 Hz, 1H, H_i), 6.70 (td, $^3J_{HH}$ = 7.3 Hz, $^4J_{HH}$ = 1.4 Hz, 1H), 4.09 (s, 1H, H_s), 3.93 (s, 3H, H_m) ppm. **^{13}C{^{1}H} NMR** (150 MHz; Acetone-d$_6$): δ_C 174.6, 164.9, 161.0, 156.6, 154.9, 154.7, 147.2, 146.9, 143.2, 136.7, 133.0 (t, $^3J_{CP}$ = 4.9 Hz), 132.8, 131.8, 131.3 (t, $^2J_{CP}$ = 19.9 Hz), 131.1, 129.3, 128.6, 127.9 (t, $^3J_{CP}$ = 4.6 Hz), 126.3, 126.1, 123.7, 122.0, 120.5, 116.3, 114.4, 113.1, 83.1, 81.9, 54.9 ppm. **^{31}P{^{1}H} NMR** (242 MHz; Acetone-d$_6$): δ_P 31.5 (s, 2P), -144.4 (sept, $^2J_{PF}$ = 707 Hz, 1P) ppm. **MS**(MALDI): m/z 963.7 [M- C_9H_5N-PF_6]$^+$. **Anal. Calc.** for $C_{68}H_{52}F_6N_3OP_3Ru$: C, 66.12; H, 4.24; N, 3.40 %. **Found**: C, 66.15; H, 4.23; N, 3.38 %. **IR** (C≡N) ν 2203 cm^{-1}.

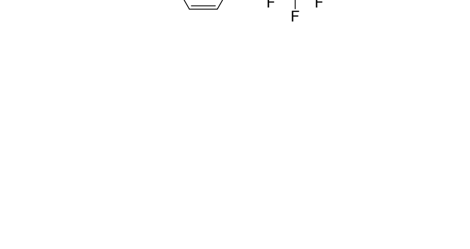

trans-Ru(dpp)(PPh_3)$_2$(NC-N-CN) (**3a**). Crystallisation was achieved by layering of MeOH onto a DCM solution of the complex to yield an orange microcrystalline solid. **Yield**: 160 mg (78%). **^{1}H NMR** (600 MHz; TCE-d$_2$): δ_H 7.53 (d, $^3J_{HH}$ = 5.9 Hz, 2H, H$_a$), 7.31-7.26 (m, 10H, H$_f$+H$_j$), 7.21-7.19 (m, 14H, H$_g$+H$_i$), 7.07-7.04 (m, 12H, H$_h$), 7.02 (d, $^3J_{HH}$ = 6.9 Hz, 4H, H$_e$), 6.93 (t, $^3J_{HH}$ = 6.9 Hz, 2H, H$_c$), 6.74 (d, $^3J_{HH}$ = 7.6 Hz, 2H, H$_d$), 6.20 (td, $^3J_{HH}$ = 7.1 Hz, $^4J_{HH}$ = 1.4 Hz, 2H, H$_b$) ppm. **^{13}C{^{1}H} NMR** (150 MHz; TCE-d$_2$): δ_C 159.5, 155.9, 138.3 (t, $^3J_{CP}$ = 4.7 Hz), 135.8, 135.0, 133.4, 133.3, 131.9 (t, $^2J_{CP}$ = 17.1 Hz), 131.7, 130.6, 129.4, 128.0 (t, $^3J_{CP}$ = 4.2 Hz), 127.9, 125.7, 119.6, 117.8, 116.5 ppm. **^{31}P{^{1}H} NMR** (242 MHz; TCE-d$_2$): δ_P 30.3 (s) ppm. **Anal. Calc.** for $C_{64}H_{48}N_6P_2Ru\cdot\frac{1}{4}CH_2Cl_2$: C, 71.10; H, 4.50; N, 7.74 %. **Found**: C, 70.96; H, 4.46; N, 7.70 %. **IR** (C≡N) ν 2154, 2256 cm^{-1}.

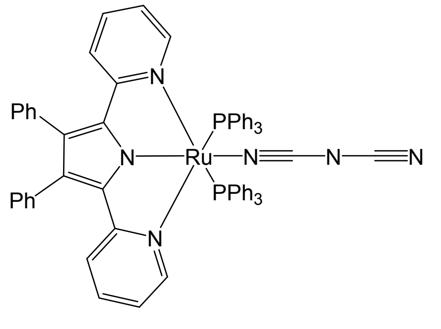

trans-Ru(Pbpy)(PPh_3)$_2$(NC-N-CN) (**3c**). Crystallisation was achieved by layering of MeOH onto a DCM solution of the complex to yield red crystals. **Yield**: 60 mg (59%). **^{1}H NMR** (600 MHz; Acetone-d$_6$): δ_H 8.72 (d, $^3J_{HH}$ = 6.5 Hz, 1H, H$_a$), 8.16 (d, $^3J_{HH}$ = 8.0 Hz, 1H, H$_d$), 8.12 (d, $^3J_{HH}$ = 7.2 Hz, 1H, H$_j$), 7.80 (d, $^4J_{HH}$ = 1.5 Hz, 1H, H$_e$), 7.71-7.68 (m, 3H, H$_c$+H$_k$), 7.17-7.13 (m, 19H, H$_b$+H$_n$+H$_p$), 7.10-7.03 (m, 14H, H$_l$+H$_o$), 7.04 (d, $^3J_{HH}$ = 7.8 Hz, 1H, H$_g$), 6.96 (d, $^4J_{HH}$ = 1.3 Hz, 1H, H$_f$), 6.72 (t, $^3J_{HH}$ = 7.4 Hz, 1H, H$_i$), 6.57 (t, $^3J_{HH}$ = 7.4 Hz, 1H, H$_h$), 3.90 (s, 3H, H$_m$) ppm. **^{13}C{^{1}H} NMR** (150 MHz; Acetone-d$_6$): δ_C 166.4, 160.5, 157.0, 153.4, 148.1, 144.3, 135.3, 133.0 (t, $^3J_{CP}$ = 4.6 Hz), 132.5 (t, $^2J_{CP}$ = 19.0 Hz), 130.9, 128.3, 128.3, 127.4 (t, $^3J_{CP}$ = 4.8 Hz), 126.9, 125.9, 123.0, 121.1, 119.3, 115.5, 114.3, 54.8, 54.0,

48.8 ppm. **$^{31}P\{^1H\}$ NMR** (242 MHz; Acetone-d_6): δ_P 31.5 (s) ppm. **MS**(MALDI): m/z 1029.1 $[M]^+$. **Anal. Calc.** for $C_{61}H_{47}N_5P_2Ru\cdot¼CH_2Cl_2$: C, 68.93; H, 4.51; N, 6.54 %. **Found**: C, 68.83; H, 4.59; N, 6.49 %. **IR** (C≡N) ν 2149, 2252 cm^{-1}.

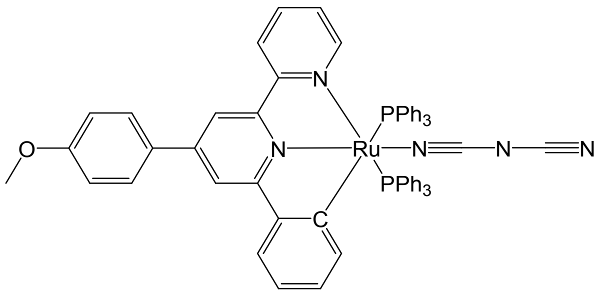

[{*trans*-Ru(L)(PPh$_3$)$_2$}$_2$(μ-NC-R-CN)]$^{n+}$ general synthesis. A suspension of *trans*-Ru(L)(PPh$_3$)$_2$Cl (1 eq), NC-R-CN (0.5 eq) and NH_4PF_6 (5 eq) in MeOH (40 mL) was refluxed for 12 hours. After cooling to room temperature, the precipitate was collected by filtration and thoroughly washed with MeOH. Final purification was achieved by crystallisation.

[{*trans*-Ru(dpp)(PPh$_3$)$_2$}$_2$(μ-NC-C_6H_4-CN)](PF$_6$)$_2$ (**4a**). Crystallisation was achieved by vapour diffusion of Et_2O into an acetone solution to yield orange crystals. **Yield**: 220 (91 %). **^1H NMR** (600 MHz; Acetone-d_6): δ_H 8.50 (d, $^3J_{HH}$ = 6.1 Hz, 4H, H_a), 7.60 (s, 4H, H_k), 7.42 (t, $^3J_{HH}$ = 7.4 Hz, 12H, H_j), 7.34-7.29 (m, 32H, H_f+H_i), 7.25-7.21 (m, 28H, H_g+H_h), 7.14-7.12 (m, 12H, H_c+H_e), 6.70 (d, $^3J_{HH}$ = 8.0 Hz, 4H, H_d), 6.58 (t, $^3J_{HH}$ = 7.2 Hz, 4H, H_b) ppm. **$^{13}C\{^1H\}$ NMR** (150 MHz; Acetone-d_6): δ_C 159.0, 157.1, 136.3, 136.0, 135.2, 133.3 (t, $^3J_{CP}$ = 4.8 Hz), 133.2, 130.5, 130.1 (t, $^2J_{CP}$ = 10.4 Hz), 129.9, 128.5 (t, $^3J_{CP}$ = 5.7 Hz), 128.0, 126.2, 120.6, 117.1, 115.9 ppm.[ii] **$^{31}P\{^1H\}$ NMR** (242 MHz; Acetone-d_6): δ_P 28.6 (s, 4P), -144.4

[ii] Not all carbon signals visible

(sept, $^2J_{PF}$ = 707 Hz, 2P) ppm. **MS**(MALDI): m/z 736.1 $[Ru(dpp)(PPh_3)_2]^+$. **Anal. Calc.** for $C_{132}H_{100}F_{12}N_8P_6Ru_2$: C, 65.67; H, 4.17; N, 4.64 %. **Found**: C, 65.60; H, 4.14; N, 4.62 %. **IR** (C≡N) ν 2207 cm^{-1}.

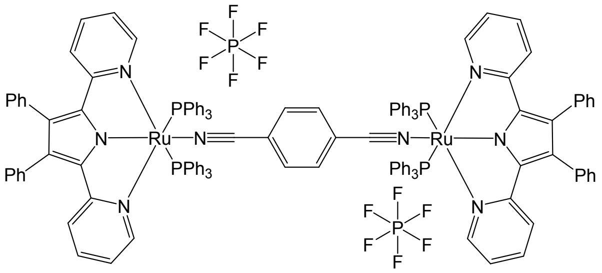

$[\{trans\text{-}Ru(BPI)(PPh_3)_2\}_2(\mu\text{-}NC\text{-}C_6H_4\text{-}CN)](PF_6)_2$ (**4b**). Crystallisation was achieved by vapour diffusion of Et_2O into an acetone solution to yield purple crystals. **Yield**: 196 mg (83%). **^{1}H NMR** (600 MHz; TCE-d$_2$): δ_H 8.90 (dd, $^3J_{HH}$ = 6.1 Hz, $^4J_{HH}$ = 1.7 Hz, 4H, H_a), 7.79-7.75 (m, 8H, H_c+H_e), 7.59 (dd, $^3J_{HH}$ = 5.4 Hz, $^4J_{HH}$ = 2.9, 4H, H_f), 7.56 (s, 4H, H_j), 7.37 (dd, $^3J_{HH}$ = 8.1 Hz, $^4J_{HH}$ = 1.7 Hz, 4H, H_d), 7.26 (t, $^3J_{HH}$ = 7.4 Hz, 12H, H_i), 7.05 (td, $^3J_{HH}$ = 8.6 Hz, $^4J_{HH}$ = 1.3 Hz, 24H, H_h), 6.89 (td, $^3J_{HH}$ = 7.2 Hz, $^4J_{HH}$ = 6.2 Hz, 4H, H_b), 6.78-6.75 (m, 24H, Hg) ppm.[iii] **^{31}P{^{1}H} NMR** (242 MHz; TCE-d$_2$): δ_P 26.4 (s, 4P), -144.4 (sept, $^2J_{PF}$ = 707 Hz, 2P) ppm.[iv] **Anal. Calc.** for $C_{116}H_{88}F_{12}N_{12}P_6Ru_2$: C, 61.49; H, 3.91; N, 7.42 %. **Found**: C, 61.36; H, 3.89; N, 7.41 %. **IR** (C≡N) ν 2211 cm^{-1}.

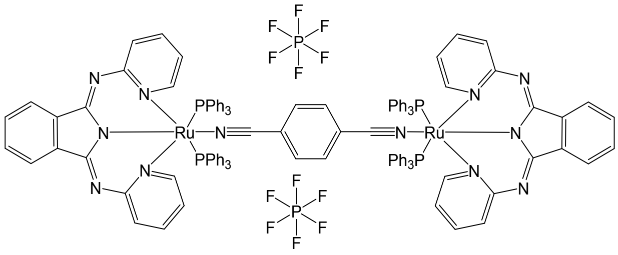

[iii] Too insoluble for ^{13}C NMR

[iv] Using MALDI no peaks corresponding to the parent ion or fragments could be identified.

[{*trans*-Ru(PBpy)(PPh_3)$_2$}$_2$(μ-NC-C_6H_4-CN)](PF_6)$_2$ (**4c**). Crystallisation was achieved by vapour diffusion of Et_2O into an acetone solution to yield red crystals. **Yield**: 190 mg (89 %).**^{1}H NMR** (700 MHz, CD_3CN): δ_H 9.07 (d, $^3J_{HH}$ = 4.9 Hz, 2H. H$_a$), 8.04 (dd, $^3J_{HH}$ = 7.5 Hz, $^4J_{HH}$ = 1.2 Hz, 2H, H$_g$), 7.93 (d, $^3J_{HH}$ = 8.1 Hz, 2H, H$_d$), 7.71-7.67 (m, 6H, H$_c$+H$_k$), 7.59 (d, $^4J_{HH}$ = 1.5 Hz, 2H, H$_e$), 7.45 (s, 4H, H$_q$), 7.28 (t, $^3J_{HH}$ = 7.4 Hz, 12H, H$_e$), 7.22 (d, $^3J_{HH}$ = 8.0 Hz, 2H, H$_j$), 7.17-7.14 (m, 30H, H$_f$+H$_l$+H$_o$), 7.12-7.09 (m, 26H, H$_b$+H$_n$), 6.85 (t, $^3J_{HH}$ = 7.1 Hz, 2H, H$_l$), 6.75 (t, $^3J_{HH}$ = 7.1 Hz, 2H, H$_h$), 3.93 (s, 6H, H$_m$) ppm. **^{13}C{^{1}H} NMR** (150 MHz; CD_3CN): δ_C 174.4, 164.4, 160.9, 156.4, 154.9, 154.2, 147.3, 146.8, 143.3, 136.8, 132.9 (t, $^3J_{CP}$ = 4.9 Hz), 132.3, 130.9 (t, $^2J_{CP}$ = 13.4 Hz), 130.0, 129.5, 128.7 (t, $^3J_{CP}$ = 4.5 Hz), 128.1, 127.8, 126.9, 125.7, 123.8, 122.0, 120.6, 116.6, 116.5, 114.5, 55.2 ppm. . **^{31}P{^{1}H} NMR** (242 MHz; CD_3CN): δ_P 31.3 (s, 4P), -144.4 (sept, $^2J_{PF}$ = 707 Hz, 2P) ppm. **MS**(MALDI): m/z 963.7 [Ru(Pbpy)(PPh_3)$_2$]$^+$. **Anal. Calc.** for $C_{126}H_{98}F_{12}N_6O_2P_6Ru_2 \cdot H_2O$: C, 64.07; H, 4.27; N, 3.56 %. **Found**: C, 64.03; H, 4.26; N, 3.49 %. **IR** (C≡N) ν 2194 cm^{-1}.

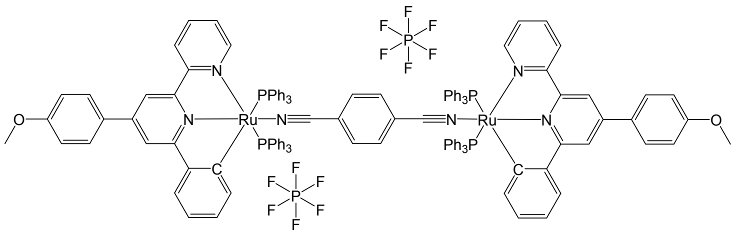

[{*trans*-Ru(dpp)(PPh_3)$_2$}$_2$(μ-NC-N-CN)](PF_6) (**5a**). Crystallisation was achieved by layering of MeOH onto a DCM solution of the complex to yield orange crystals. **Yield**: 160 mg (75%). **^{1}H NMR** (600 MHz, TCE-d$_2$): δ_H 7.70 (d, $^3J_{HH}$ = 5.6 Hz, 4H, H$_a$), 7.28 (t, $^3J_{HH}$ = 7.5 Hz, 8H, H$_f$), 7.23 (t, $^3J_{HH}$ = 7.3 Hz, 12H, H$_j$), 7.22 (t, $^3J_{HH}$ = 7.4 Hz, 4H, H$_g$), 7.16-7.13 (m, 24H, H$_h$), 7.10 (t, $^3J_{HH}$ = 7.5 Hz, 14H, H$_i$), 7.02 (t, $^3J_{HH}$ = 7.6 Hz, 4H, H$_c$), 6.98 (d, $^3J_{HH}$ = 7.3 Hz, 8H, H$_e$), 6.79 (d, $^3J_{HH}$ = 8.0 Hz, 4H, H$_d$), 6.31 (t, $^3J_{HH}$ = 6.3 Hz, 4H, H$_b$) ppm. **^{13}C{^{1}H} NMR** (150 MHz; TCE-d$_2$): δ_C 159.7, 155.9, 136.3, 135.5, 135.4, 133.3 (t, $^3J_{CP}$ = 5.6 Hz),

132.0 (t, $^{2}J_{CP}$ = 19.7 Hz), 131.7,130.5, 129.5, 128.1, 128.0 (t, $^{3}J_{CP}$ = 3.9 Hz), 125.9, 119.4, 116.8 ppm. **^{31}P{^{1}H} NMR** (242 MHz; TCE-d$_2$): δ_P 30.5 (s, 4P), -144.5 (sept, $^{2}J_{PF}$ = 762 Hz, 1P) ppm. **MS**(MALDI): m/z 998.1 [Ru(dpp)(PPh$_3$)$_2$]$^+$. **Anal. Calc.** for $C_{126}H_{96}F_6N_9P_5Ru_2{\cdot}\frac{1}{4}CH_2Cl_2$: C, 68.05; H, 4.36; N, 5.66 %. **Found**: C, 67.95; H, 4.32; N, 5.76 %. **IR** (C≡N) ν 2198 cm^{-1}.

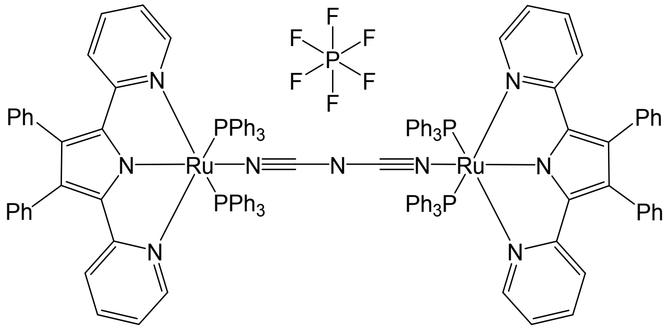

[{*trans*-Ru(BPI)(PPh$_3$)$_2$}$_2$(μ-NC-N-CN)](PF$_6$) (**5b**). Crystallisation was achieved by layering of MeOH onto a DCM solution of the complex to yield purple crystals. **Yield**: 146 mg (68%). **^{1}H NMR** (600 MHz, Acetone-d$_6$): δ_H 8.75 (dd, $^{3}J_{HH}$ = 6.0 Hz, $^{4}J_{HH}$ = 1.7 Hz, 4H, H$_a$), 7.72-7.68 (m,8H, H$_c$+H$_e$), 7.49 (dd, $^{3}J_{HH}$ = 5.4 Hz, $^{4}J_{HH}$ = 2.9 Hz, 4H, H$_f$), 7.37 (dd, $^{3}J_{HH}$ = 8.0 Hz, $^{3}J_{HH}$ = 1.5 Hz, 4H, H$_d$), 7.17 (t, $^{3}J_{HH}$ = 7.4 Hz, 12H, H$_i$), 6.97 (t, $^{3}J_{HH}$ = 7.4 Hz, 24H, H$_h$), 6.80-6.77 (m, 24H, H$_g$), 6.58 (td, $^{3}J_{HH}$ = 7.4 Hz, $^{4}J_{HH}$ = 1.6 Hz, 4H, H$_b$) ppm. **^{13}C{^{1}H} NMR** (150 MHz; Acetone-d$_6$): δ_C 157.6, 154.5, 153.3, 140.8, 135.6, 132.9 (t, $^{3}J_{CP}$ = 4.9 Hz), 131.0, 130.9 (t, $^{2}J_{CP}$ = 17.8Hz), 129.5, 129.0, 128.6, 127.9 (t, $^{3}J_{CP}$ = 4.5 Hz), 120.2, 117.5 ppm. **^{31}P{^{1}H} NMR** (242 MHz; Acetone-d$_6$): δ_P 27.6 (s, 4P), -144.5 (sept, $^{2}J_{PF}$ = 762 Hz, 1P) ppm.[v] **Anal. Calc.** for $C_{110}H_{84}F_6N_{13}P_5Ru_2{\cdot}\frac{1}{4}CH_2Cl_2$: C, 63.66; H, 4.09; N, 8.75 %. **Found**: C, 63.77; H, 4.09; N, 8.86 %. **IR** (C≡N) ν 2190 cm^{-1}.

[v] Using MALDI no peaks corresponding to the parent ion or fragments could be identified.

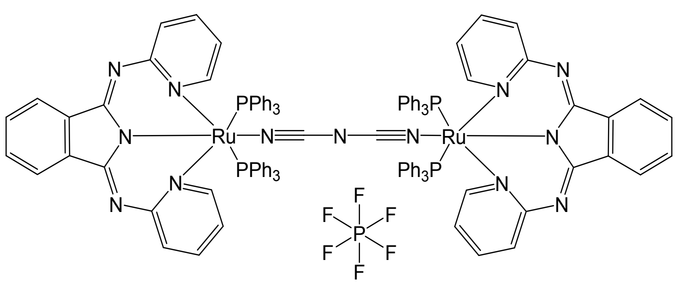

[{*trans*-Ru(PBpy)(PPh_3)$_2$}$_2$(μ-NC-N-CN)](PF_6)$_2$ (**5c**). Crystallisation was achieved by layering of MeOH onto a DCM solution of the complex to yield red crystals. **Yield**: 67 mg (63%). **^{1}H NMR** (600 MHz, Acetone-d$_6$): δ_H 8.69 (d, $^3J_{HH}$ = 5.4 Hz, 2H, H$_a$), 8.44 (d, $^3J_{HH}$ = 7.6 Hz, 2H, H$_g$), 8.22 (d, $^3J_{HH}$ = 8.1 Hz, 2H, H$_d$), 7.90 (d, $^4J_{HH}$ = 1.7 Hz, 2H, H$_e$), 7.75-7.71 (m, 6H, H$_b$+H$_k$), 7.24-7.19 (m, 26H, H$_c$+H$_n$), 7.16-7.11 (m, 16H, H$_l$+H$_p$), 7.08 (dd, $^3J_{HH}$ = 8.1 Hz, $^4J_{HH}$ = 1.4 Hz, 2H, H$_j$), 7.03-6.96 (m, 28H, H$_f$+H$_h$+H$_o$), 6.67 (t, $^3J_{HH}$ = 7.3 Hz, 2H, H$_i$), 3.92 (s, 3H, H$_m$) ppm. **^{13}C{^{1}H} NMR** (150 MHz; Acetone-d$_6$): δ_C 166.2, 160.6, 157.0, 155.6, 153.6, 148.2, 145.1, 144.9, 135.7, 133.1 (t, $^3J_{CP}$ = 5.7 Hz), 132.5 (t, $^2J_{CP}$ = 18.2 Hz), 130.6, 128.8, 128.4 (t, $^3J_{CP}$ = 4.4 Hz), 127.5, 126.9, 126.0, 125.4, 123.4, 121.1, 119.6, 115.8, 114.4, 54.9 ppm. **^{31}P{^{1}H} NMR** (242 MHz; Acetone-d$_6$): δ_P 32.1 (s, 4P), -144.5 (sept, $^2J_{PF}$ = 762 Hz, 1P) ppm. **MS**(MALDI): m/z 963.1 [Ru(Pbpy)(PPh_3)$_2$]$^+$. **Anal. Calc.** for $C_{120}H_{94}F_6N_7O_2P_5Ru_2$·¼$CH_2Cl_2$: C, 66.92; H, 4.41; N, 4.54 %. **Found**: C, 67.03; H, 4.39; N, 4.59 %. **IR** (C≡N) ν 2184 cm^{-1}.

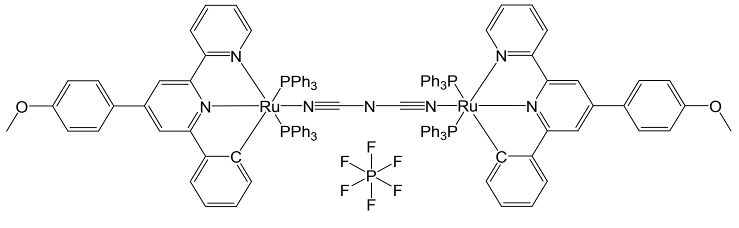

S2. NMR spectra of reported compounds

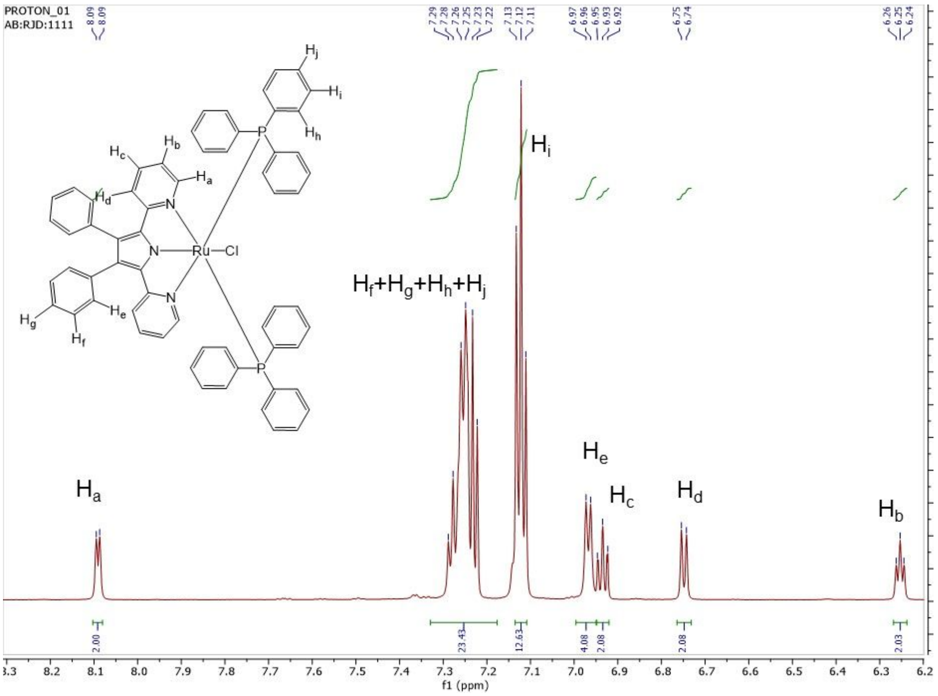

Figure S1a. ^{1}H NMR spectrum of **Cla**, recorded in CD_2Cl_2.

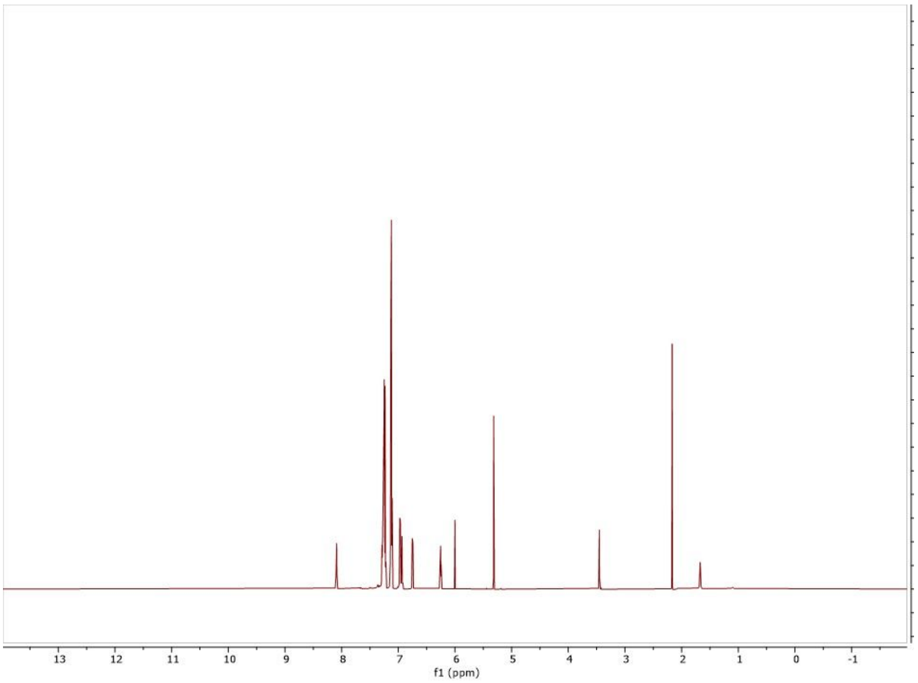

Figure S1b. ^{1}H NMR spectrum of **Cla**, recorded in CD_2Cl_2.

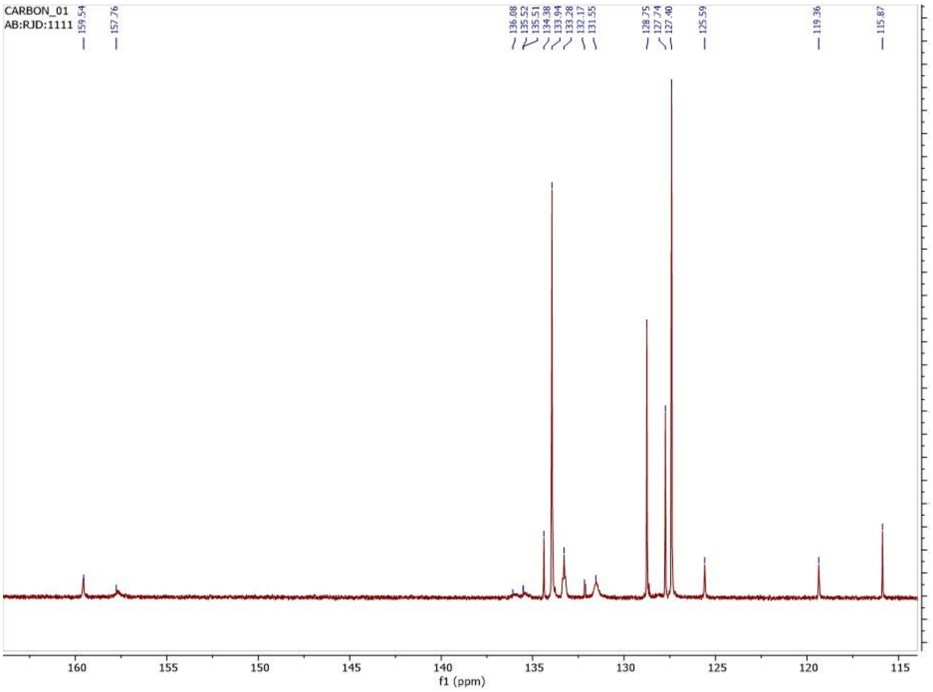

Figure S2a. $^{13}C\{^1H\}$ NMR spectrum of **Cla**, recorded in CD_2Cl_2.

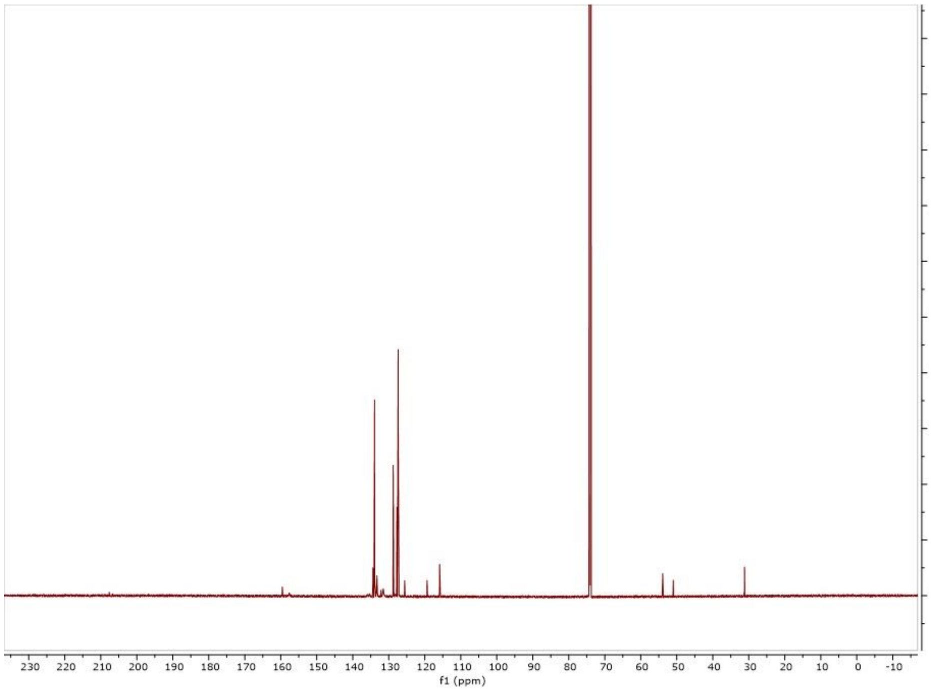

Figure S2b. $^{13}C\{^1H\}$ NMR spectrum of **Cla**, recorded in CD_2Cl_2.

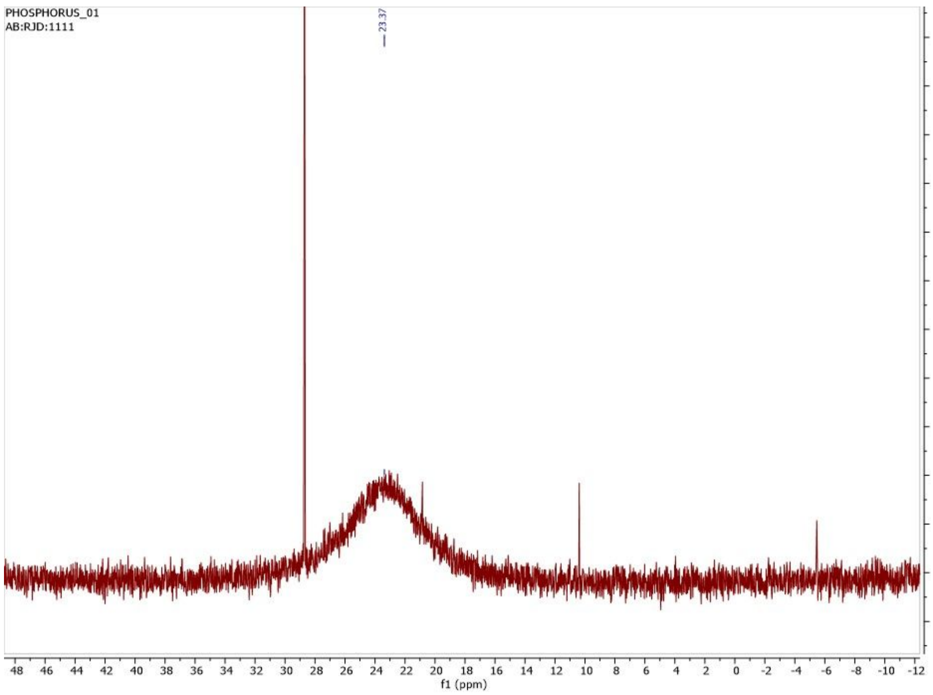

Figure S3. $^{31}P\{^1H\}$ NMR spectrum of **Cla**, recorded in CD_2Cl_2.

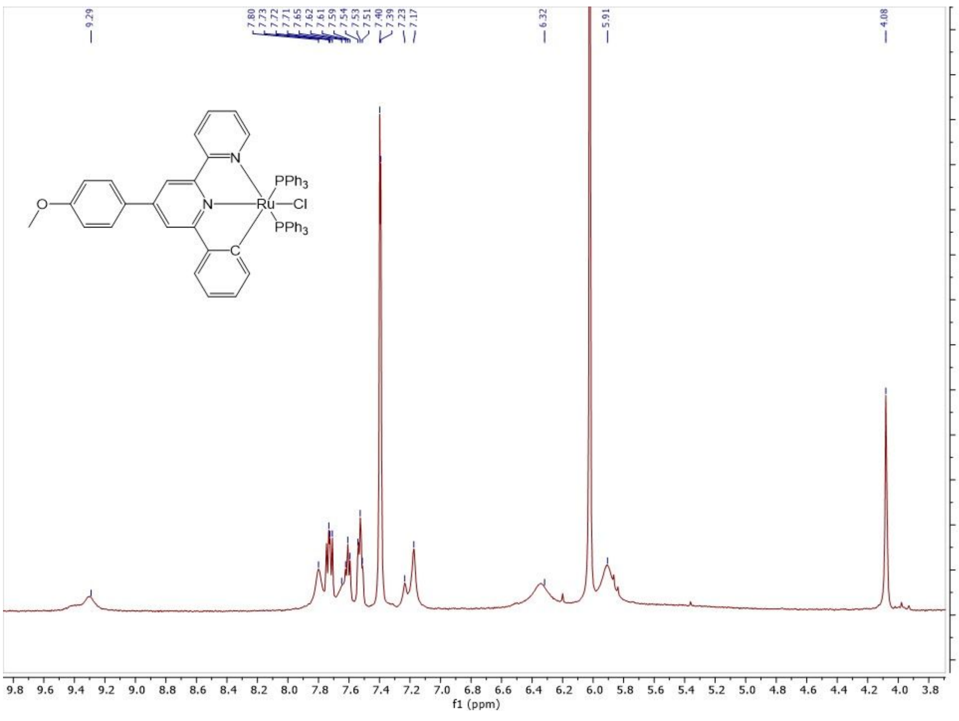

Figure S4a. ^1H NMR spectrum of **Clc**, recorded in TCE-d_2.

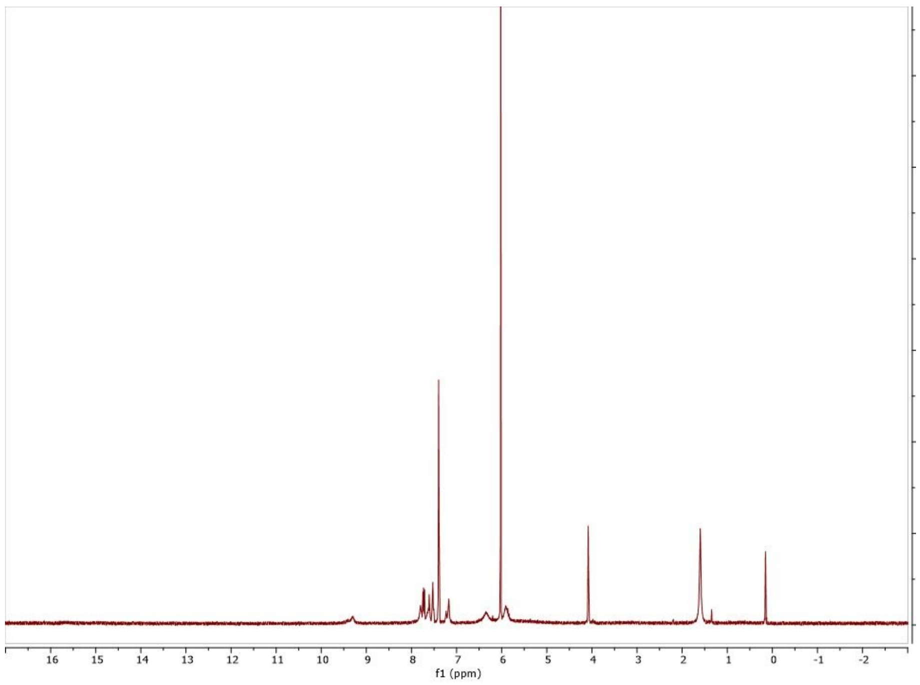

Figure S4b. ^{1}H NMR spectrum of **Clc**, recorded in TCE-d_2.

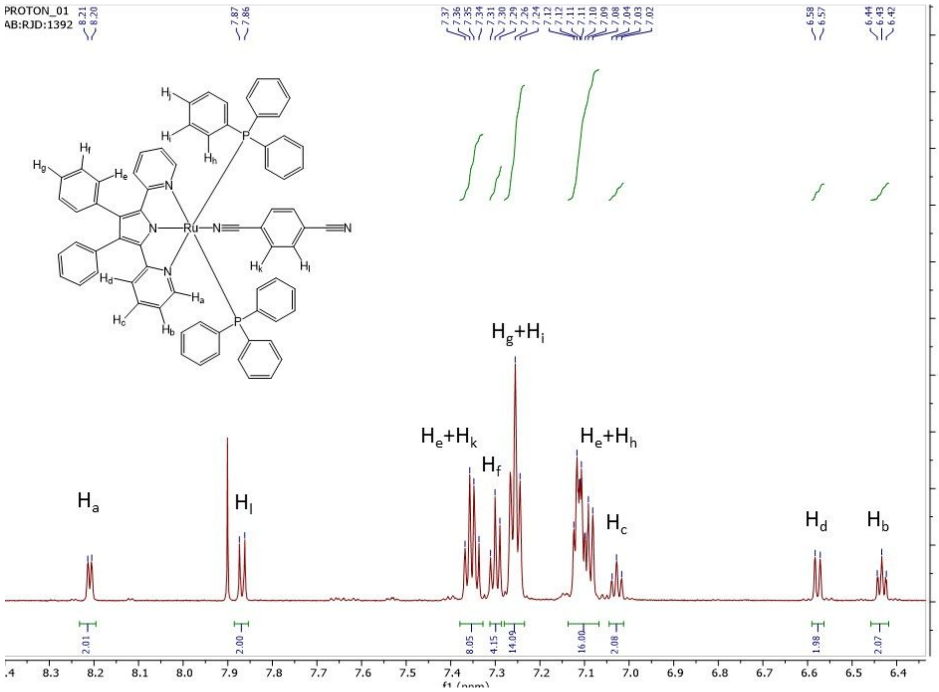

Figure S5a. ^{1}H NMR spectrum of **1a** recorded in CD_3CN.

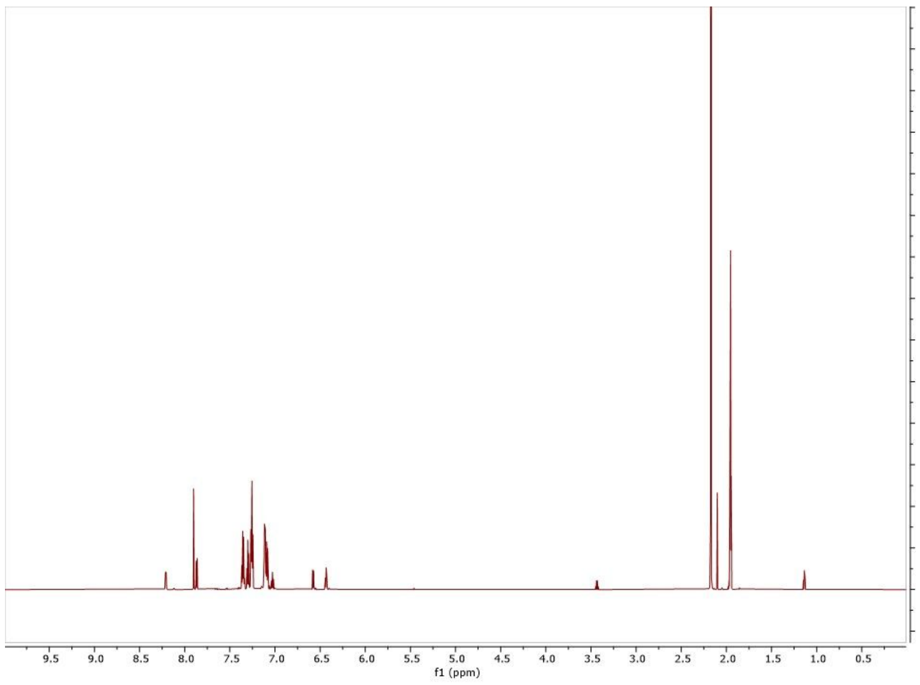

Figure S5b. ^{1}H NMR spectrum of **1a** recorded in CD_3CN.

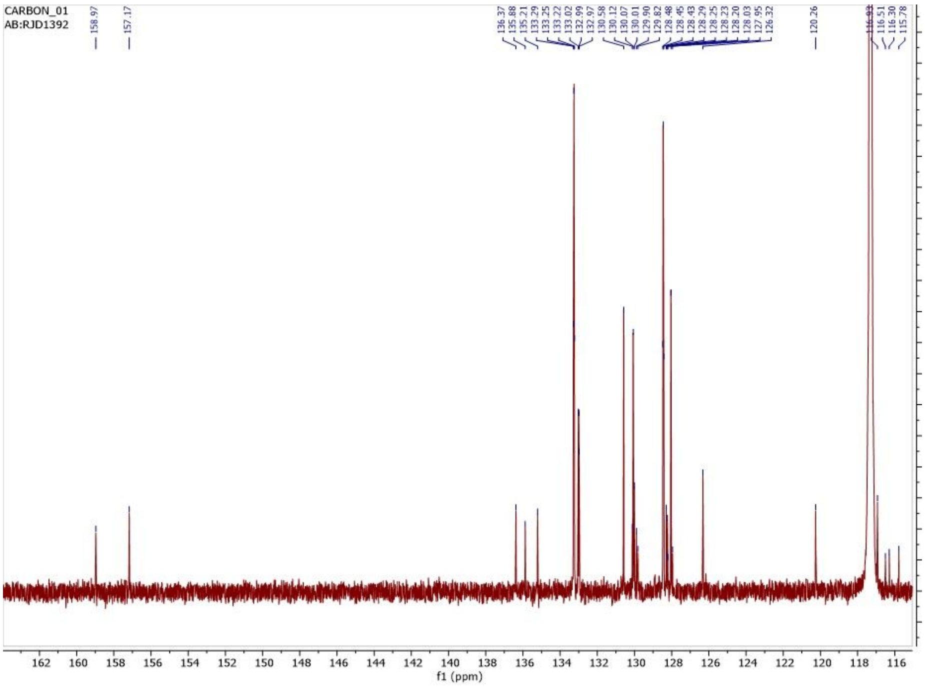

Figure S6a. ^{13}C{^{1}H} NMR spectrum of **1a** recorded in CD_3CN.

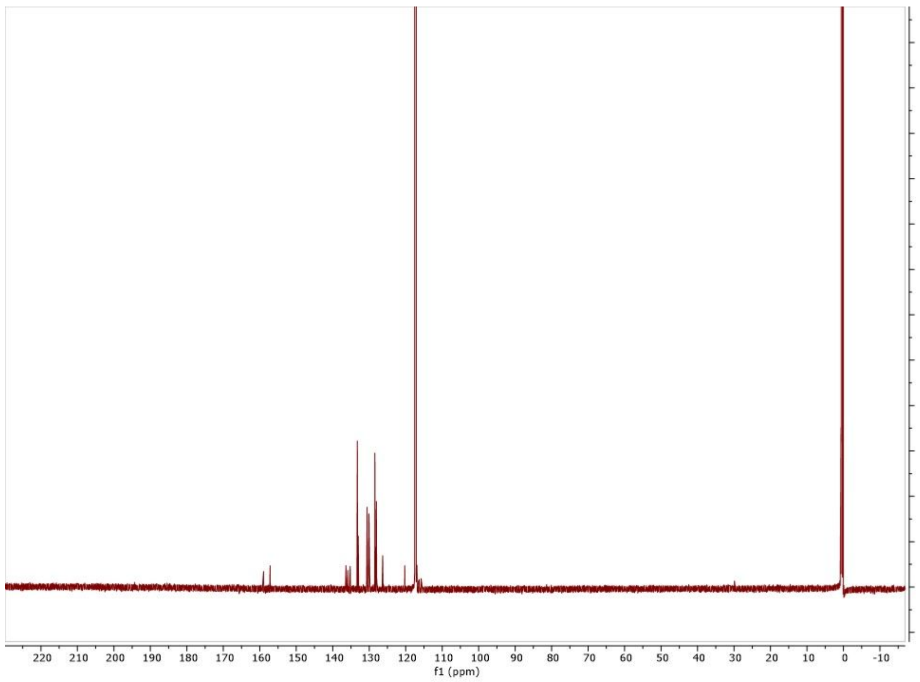

Figure S6b. $^{13}C\{^1H\}$ NMR spectrum of **1a** recorded in CD_3CN.

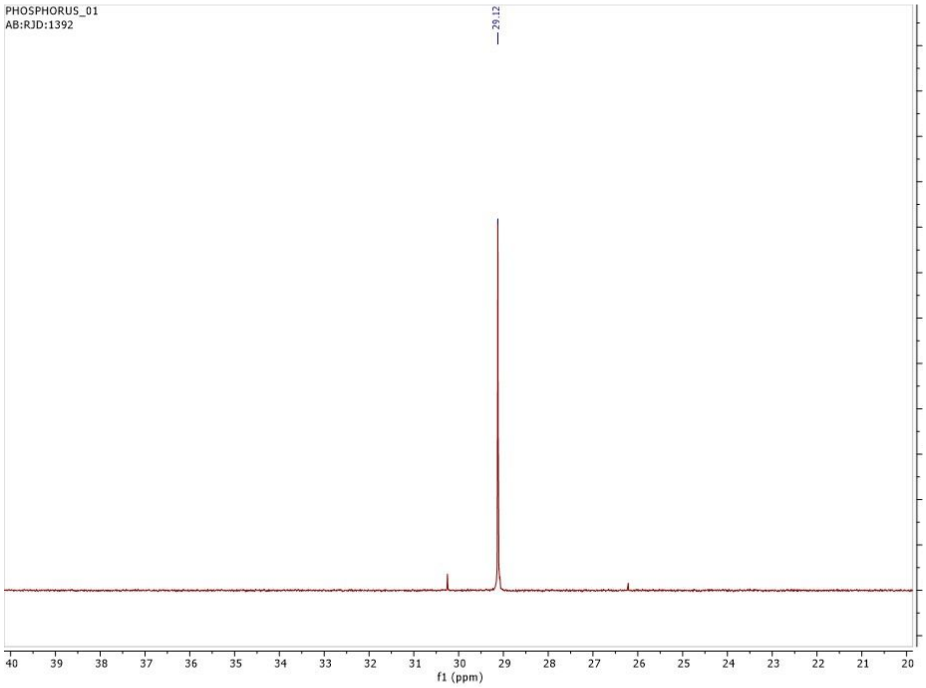

Figure S7. $^{31}P\{^1H\}$ NMR spectrum of **1a** recorded in CD_3CN.

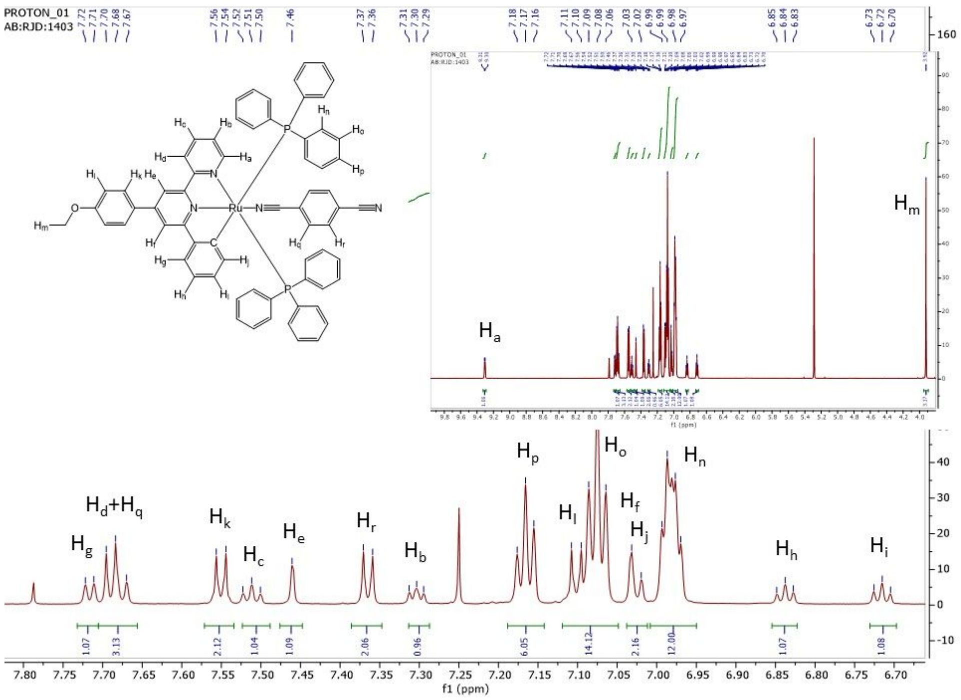

Figure S8a. ^{1}H NMR spectrum of **1c** recorded in $CDCl_3$.

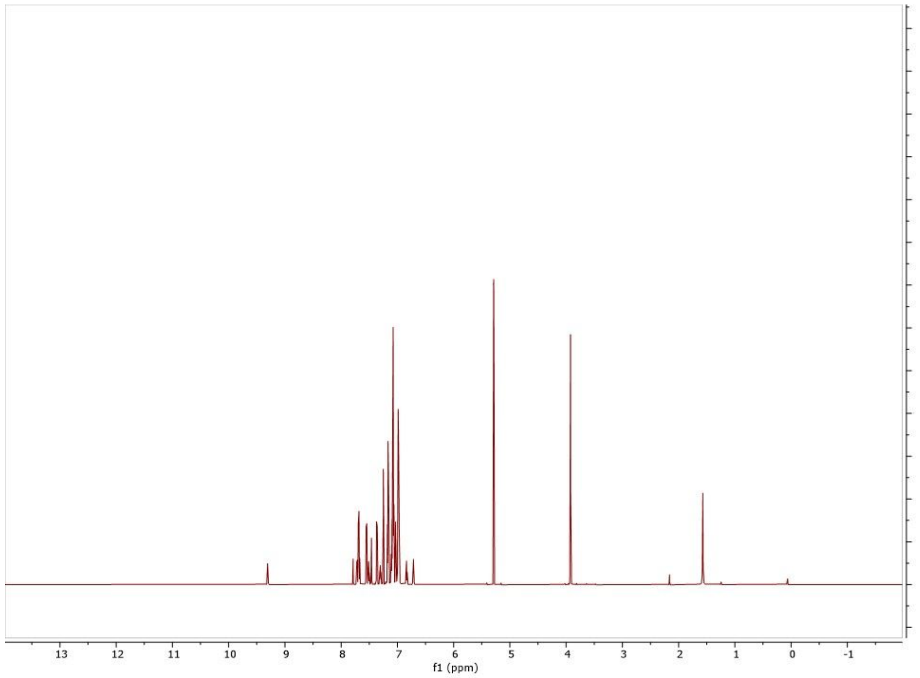

Figure S8b. ^{1}H NMR spectrum of **1c** recorded in $CDCl_3$.

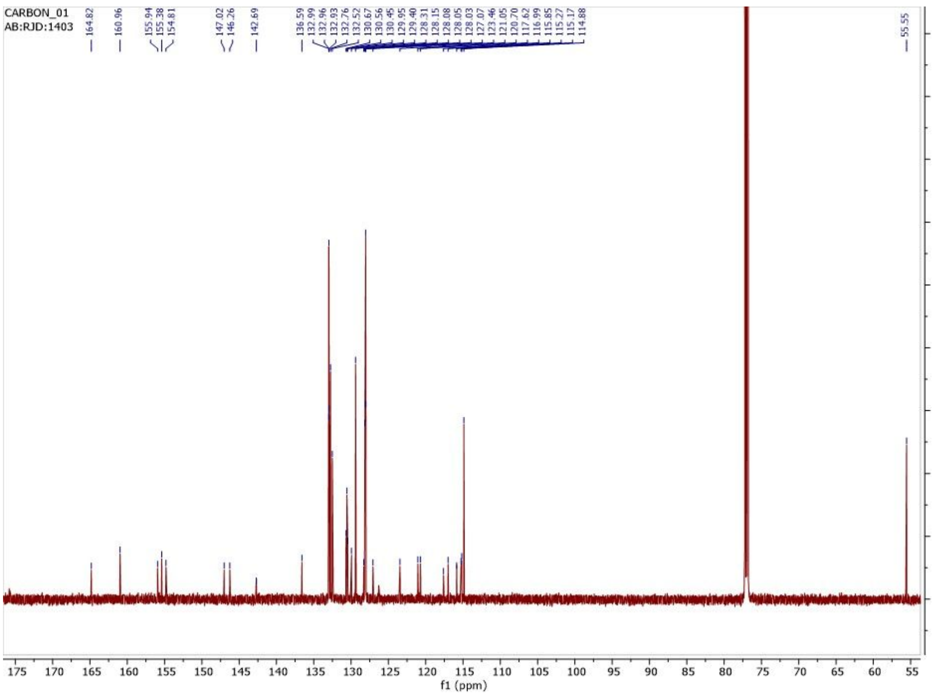

Figure S9a. $^{13}C\{^{1}H\}$ NMR spectrum of **1c** recorded in $CDCl_3$.

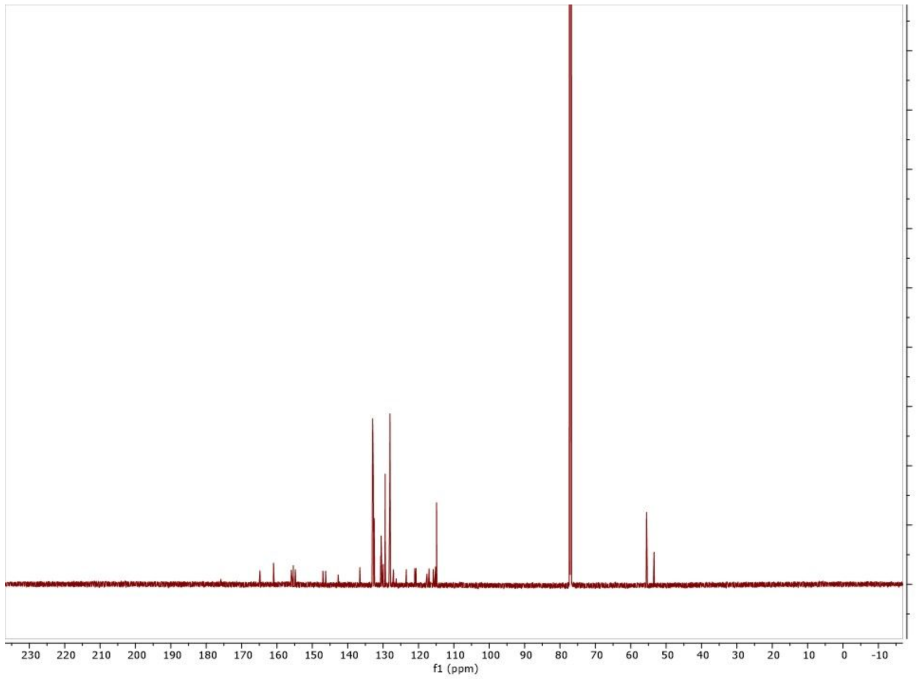

Figure S9b. $^{13}C\{^{1}H\}$ NMR spectrum of **1c** recorded in $CDCl_3$.

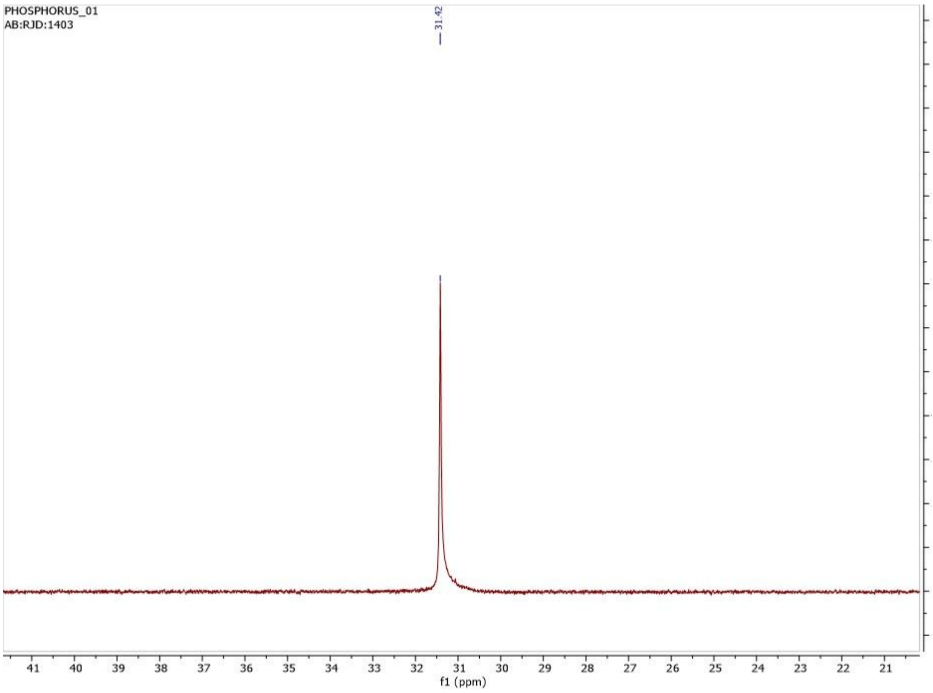

Figure S10. $^{31}P\{^1H\}$ NMR spectrum of **1c** recorded in $CDCl_3$.

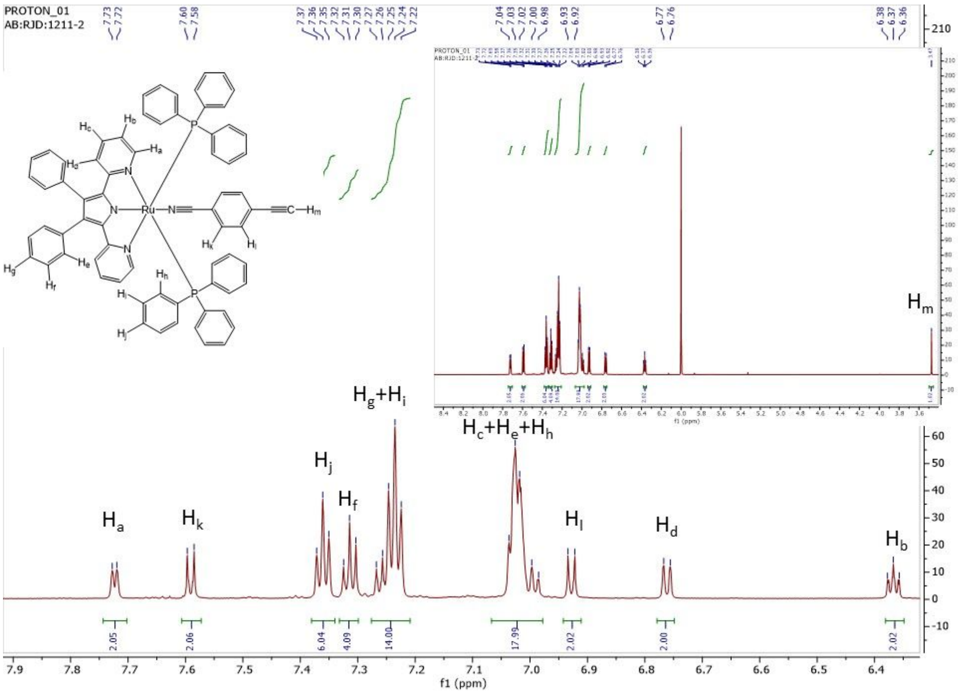

Figure S11a. ^1H NMR spectrum of **2a** recorded in TCE-d_2.

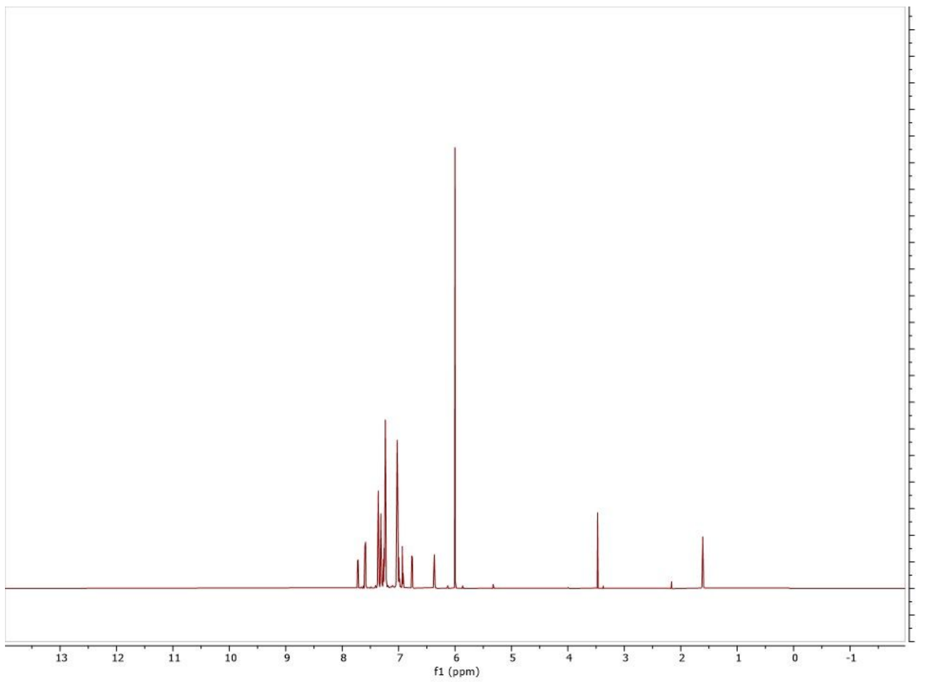

Figure S11b. ^{1}H NMR spectrum of **2a** recorded in TCE-d_2.

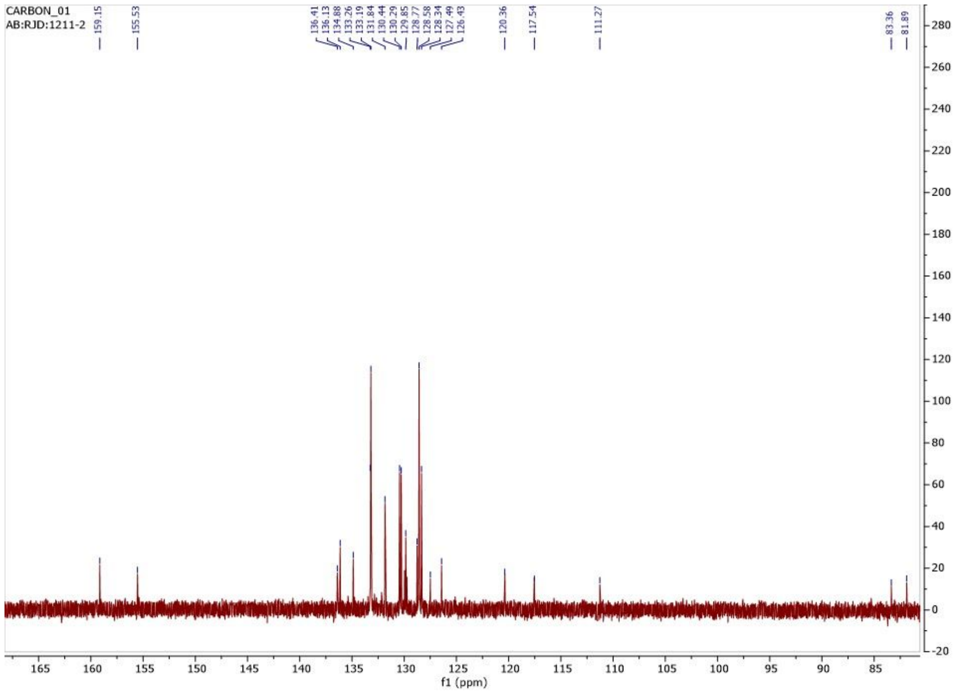

Figure S12a. ^{13}C{^{1}H} NMR spectrum of **2a** recorded in TCE-d_2.

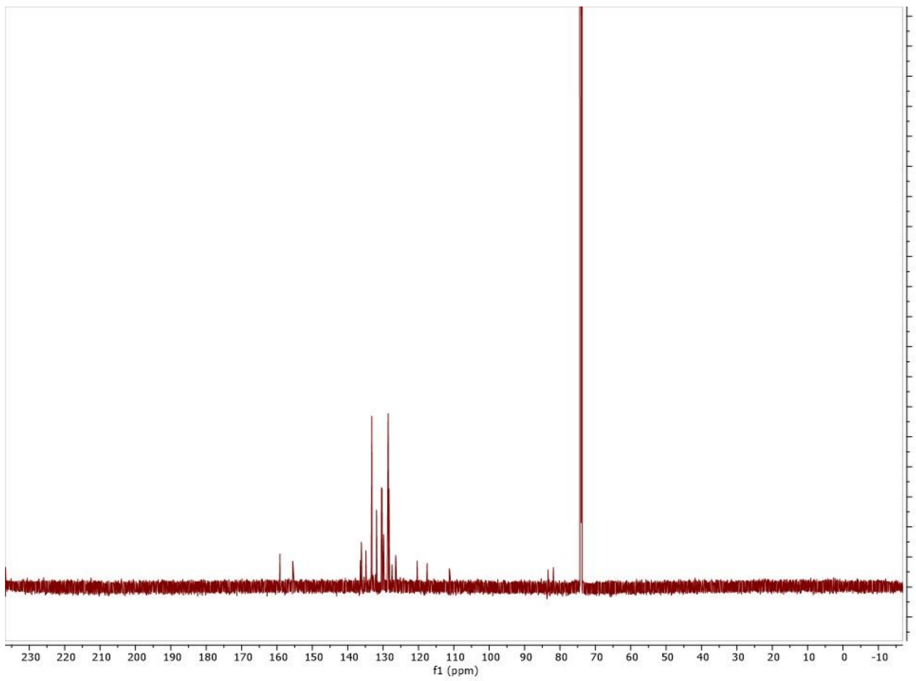

Figure S12b. $^{13}C\{^{1}H\}$ NMR spectrum of **2a** recorded in TCE-d_2.

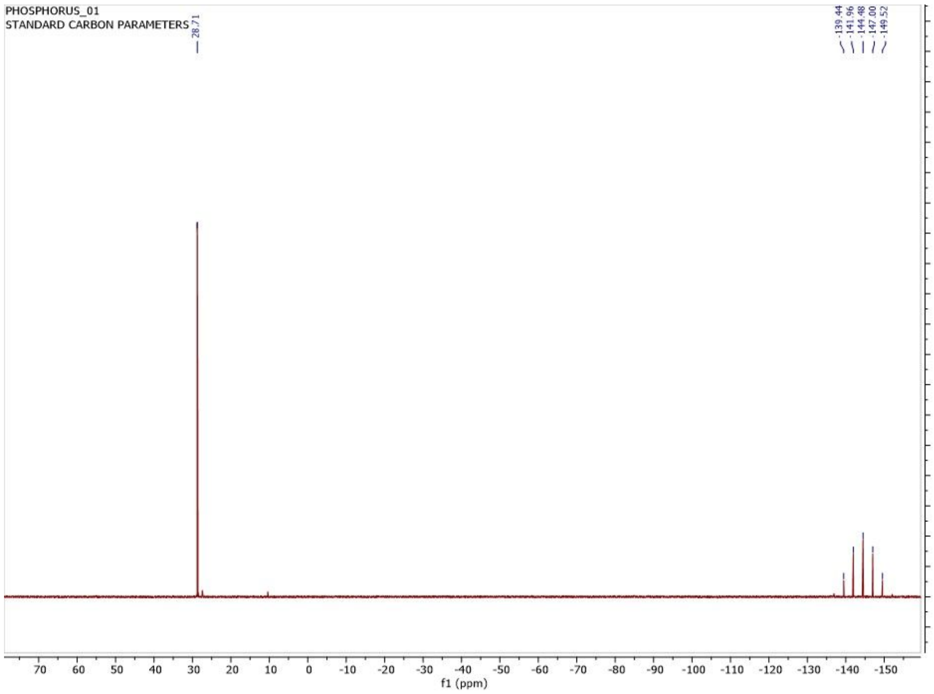

Figure S13. $^{31}P\{^{1}H\}$ NMR spectrum of **2a** recorded in TCE-d_2.

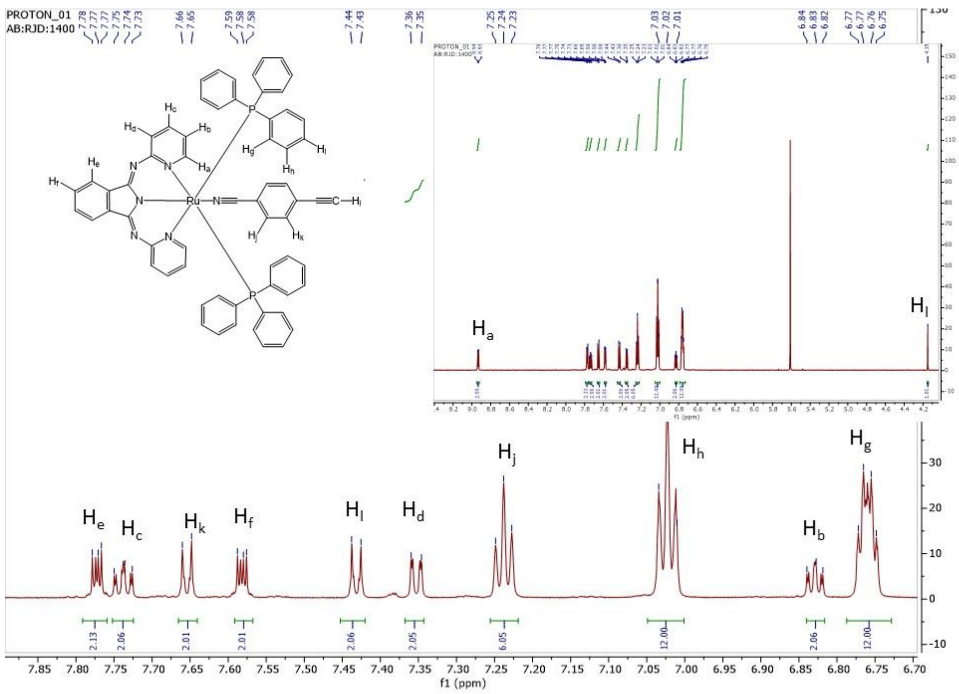

Figure S14a. ^{1}H NMR spectrum of **2b** recorded in acetone-d_6.

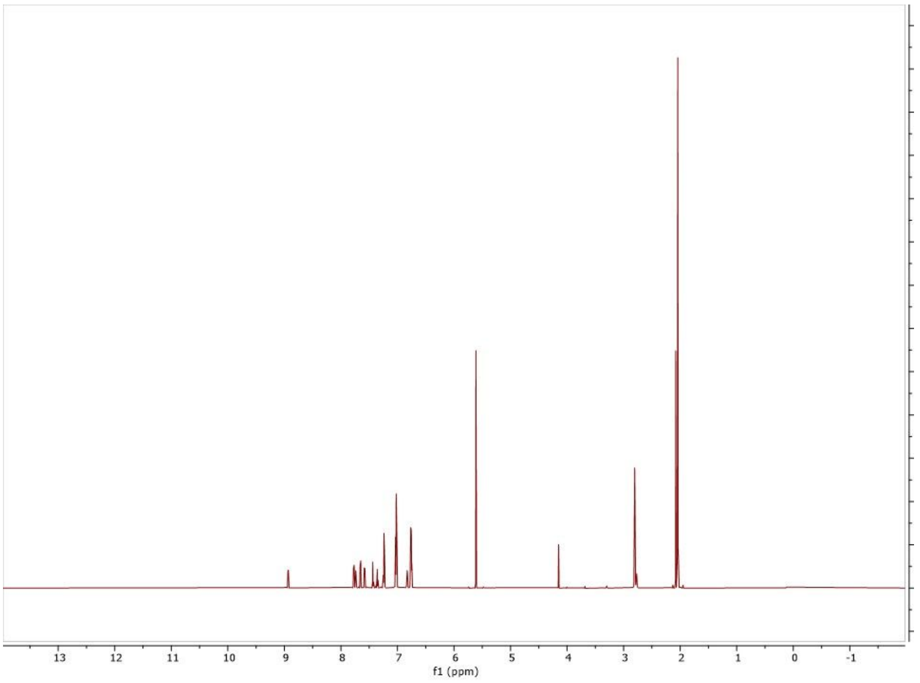

Figure S14b. ^{1}H NMR spectrum of **2b** recorded in acetone-d_6.

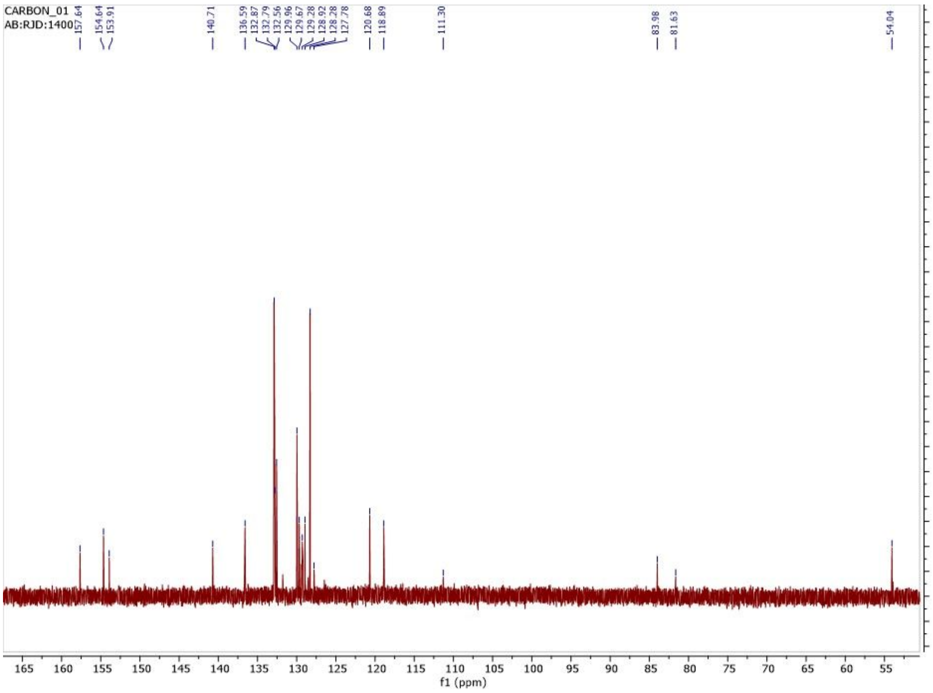

Figure S15a. $^{13}C\{^{1}H\}$ NMR spectrum of **2b** recorded in acetone-d_6.

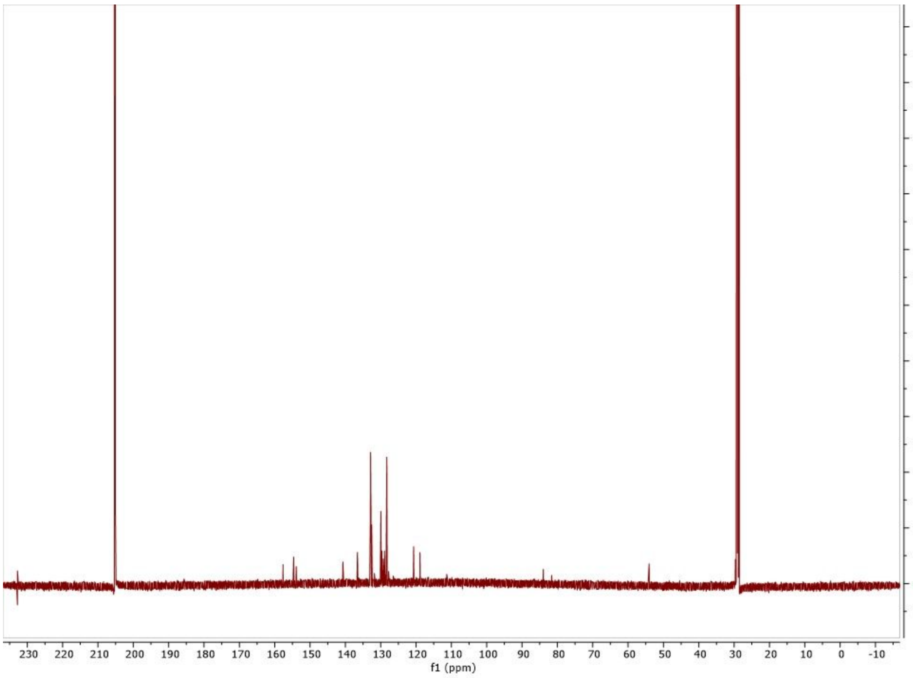

Figure S15b. $^{13}C\{^{1}H\}$ NMR spectrum of **2b** recorded in acetone-d_6.

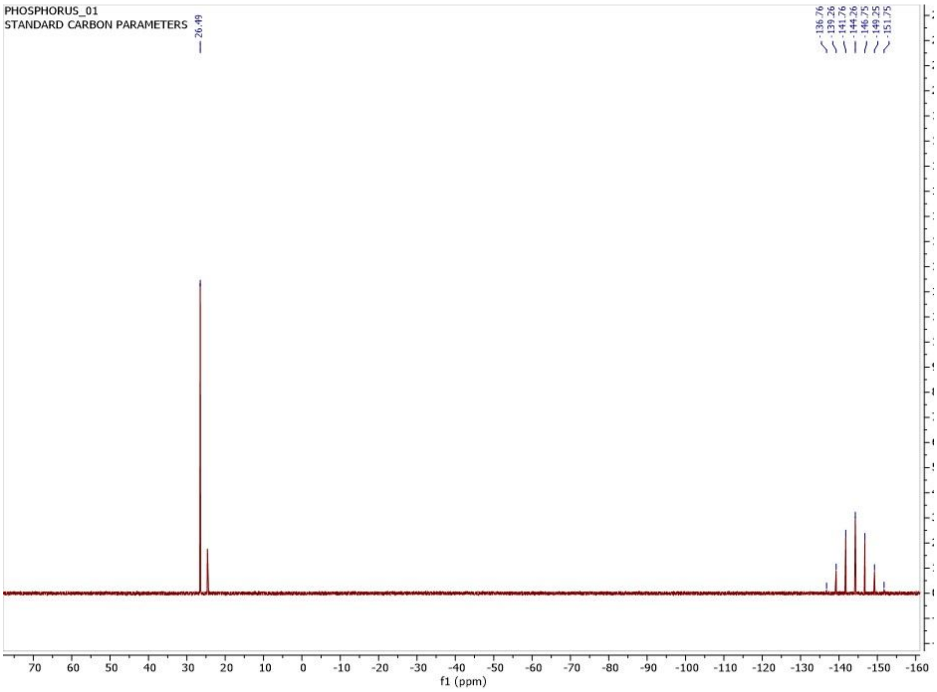

Figure S16. $^{31}P\{^{1}H\}$ NMR spectrum of **2b** recorded in TCE-d$_2$.

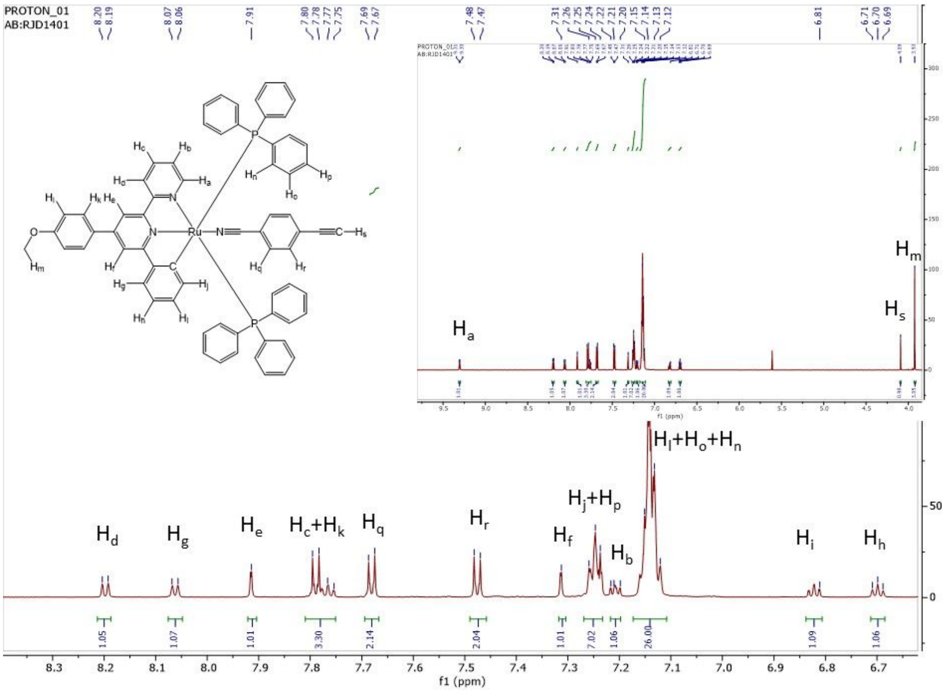

Figure S17a. ^{1}H NMR spectrum of **2c** recorded in Acetone-d$_6$.

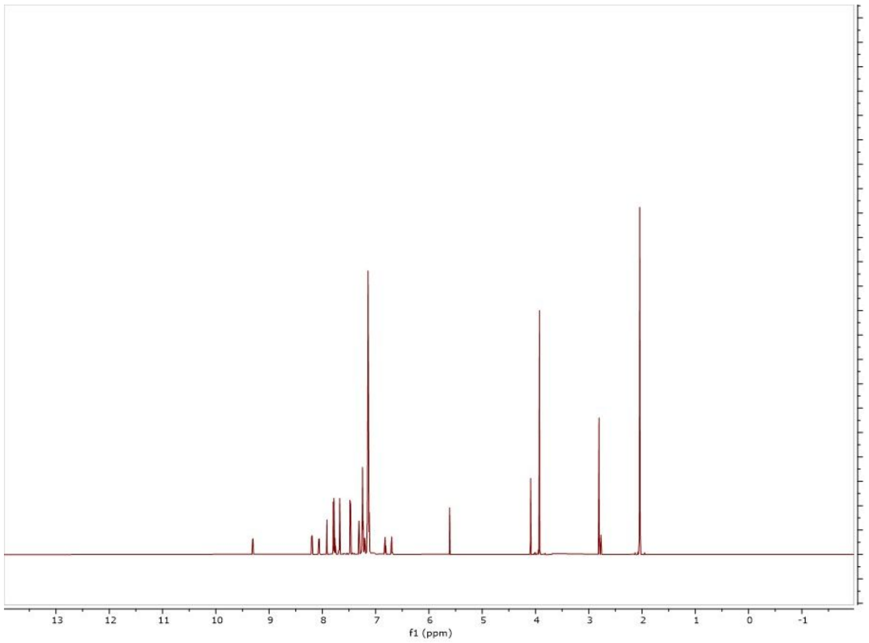

Figure S17b. ^{1}H NMR spectrum of **2c** recorded in Acetone-d_6.

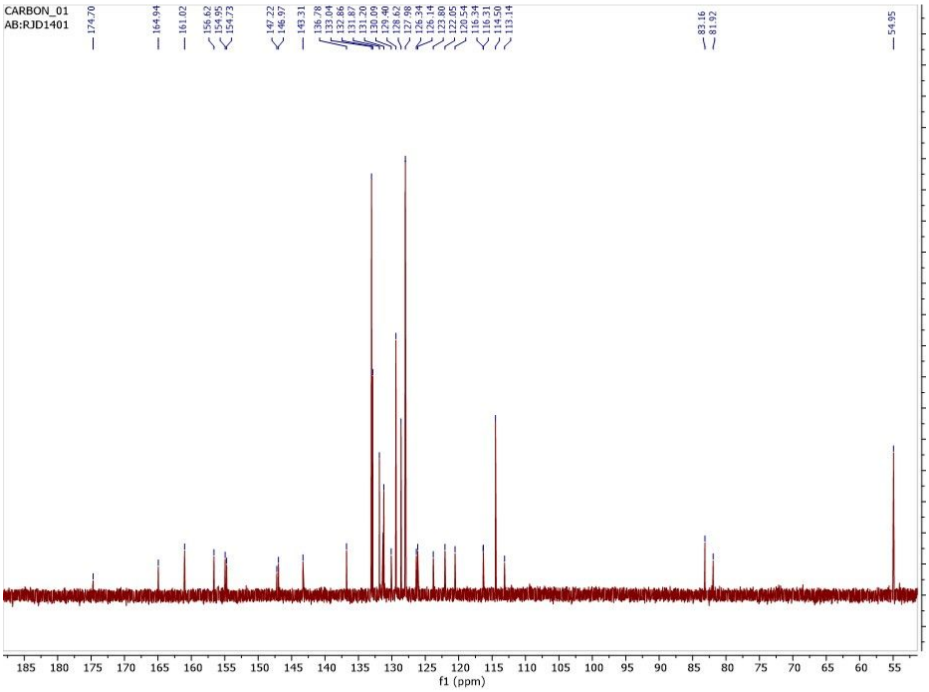

Figure S18a. ^{13}C{^{1}H} NMR spectrum of **2c** recorded in Acetone-d_6.

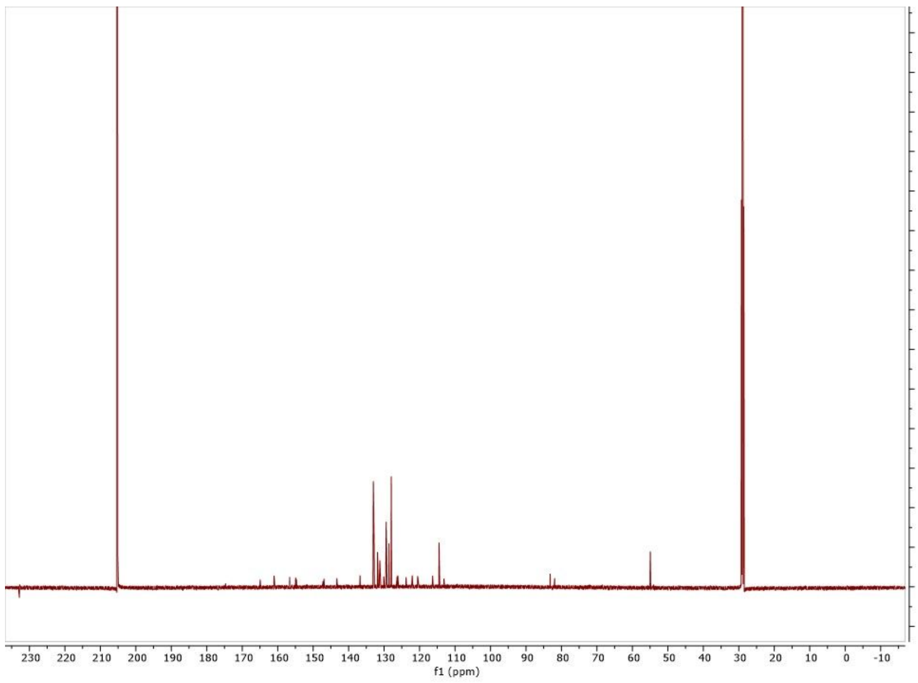

Figure S18b. $^{13}C\{^1H\}$ NMR spectrum of **2c** recorded in Acetone-d_6.

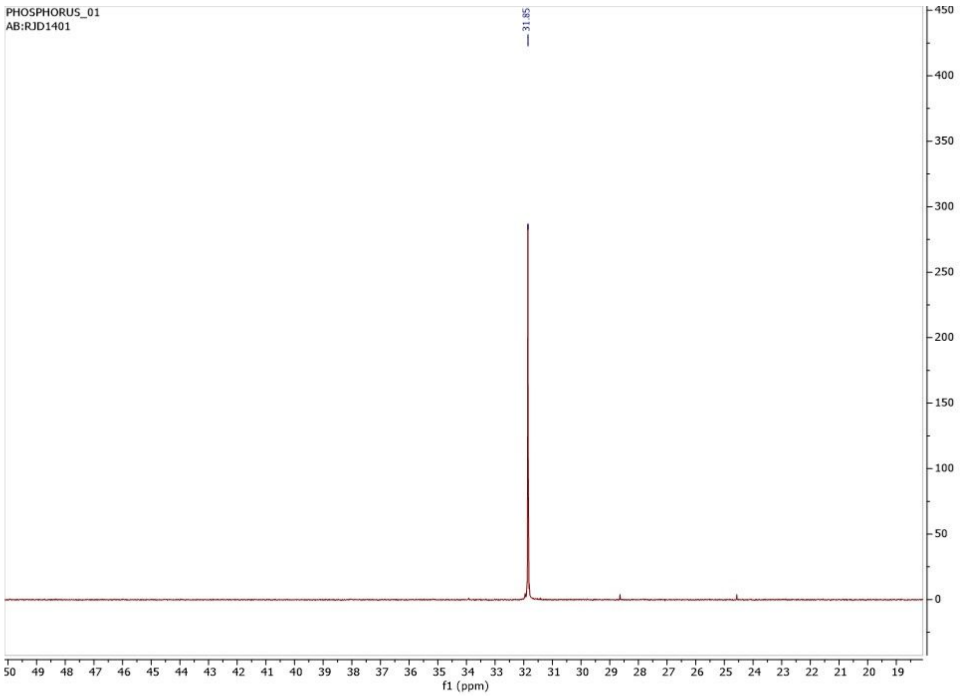

Figure S19. $^{31}P\{^1H\}$ NMR spectrum of **2c** recorded in Acetone-d_6.

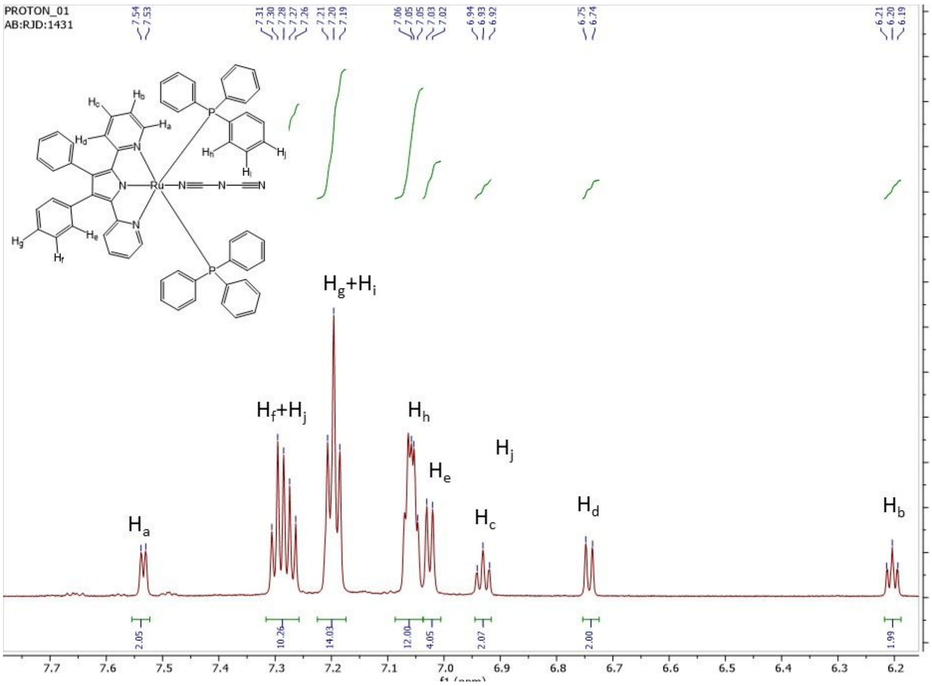

Figure S20a. ^{1}H NMR spectrum of **3a** recorded in TCE-d$_2$.

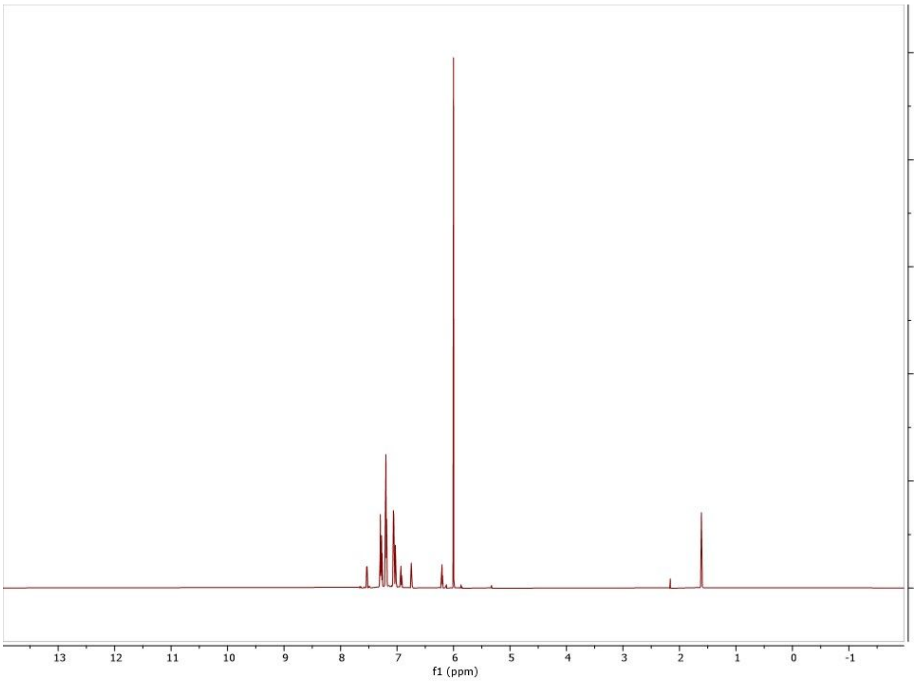

Figure S20b. ^{1}H NMR spectrum of **3a** recorded in TCE-d$_2$.

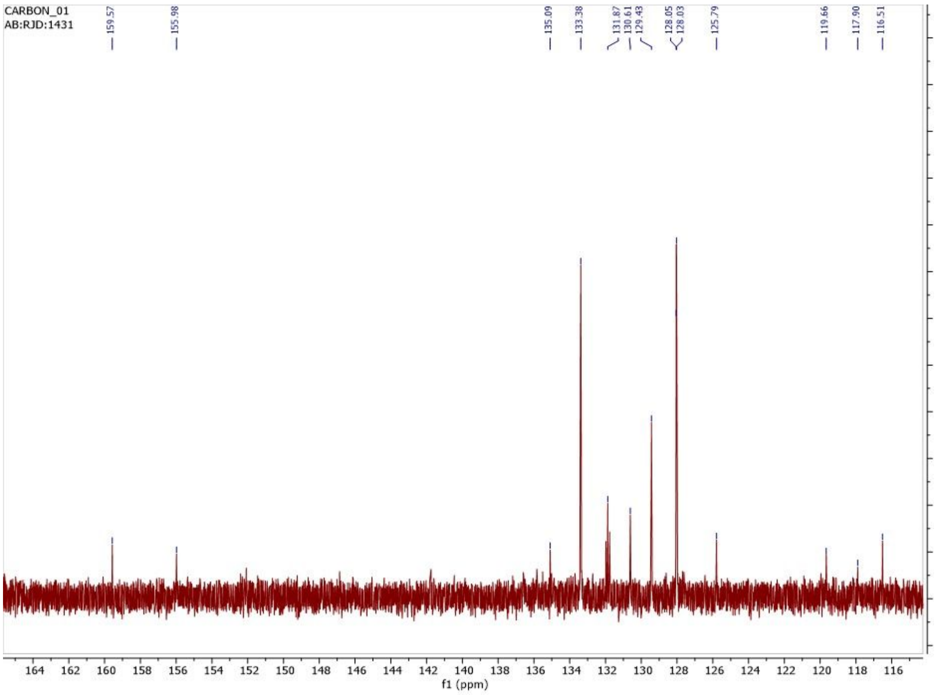

Figure S21a. $^{13}C\{^1H\}$ NMR spectrum of **3a** recorded in TCE-d$_2$.

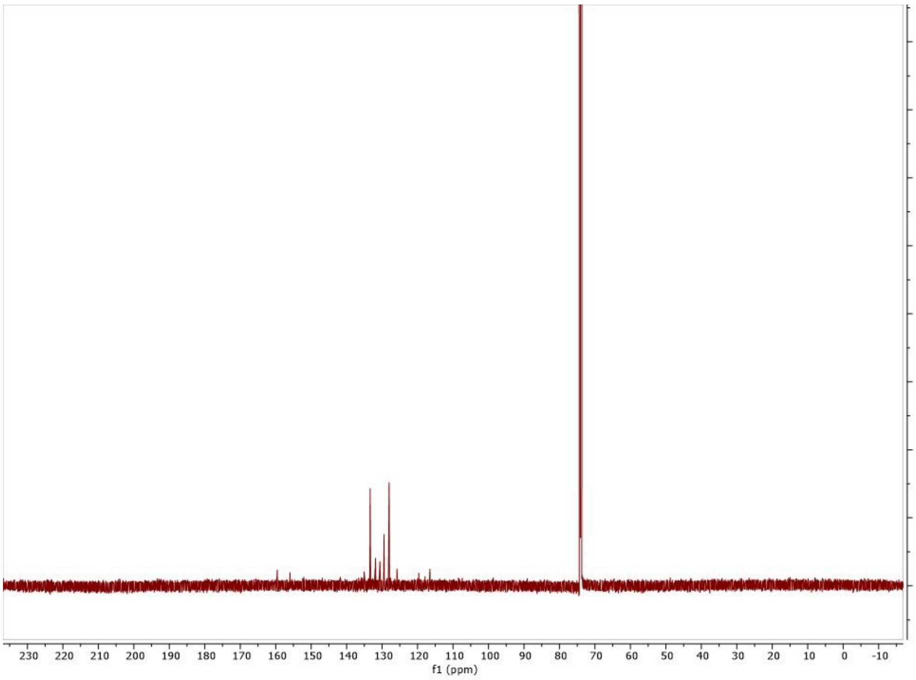

Figure S21b. $^{13}C\{^1H\}$ NMR spectrum of **3a** recorded in TCE-d$_2$.

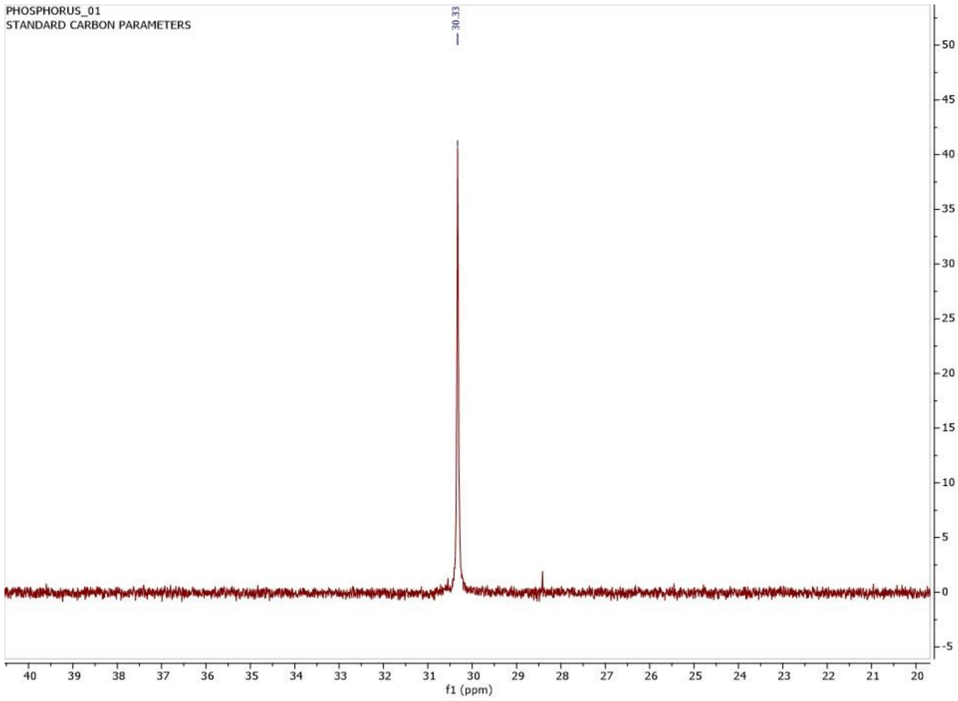

Figure S22. $^{31}P\{^{1}H\}$ NMR spectrum of **3a** recorded in TCE-d_2.

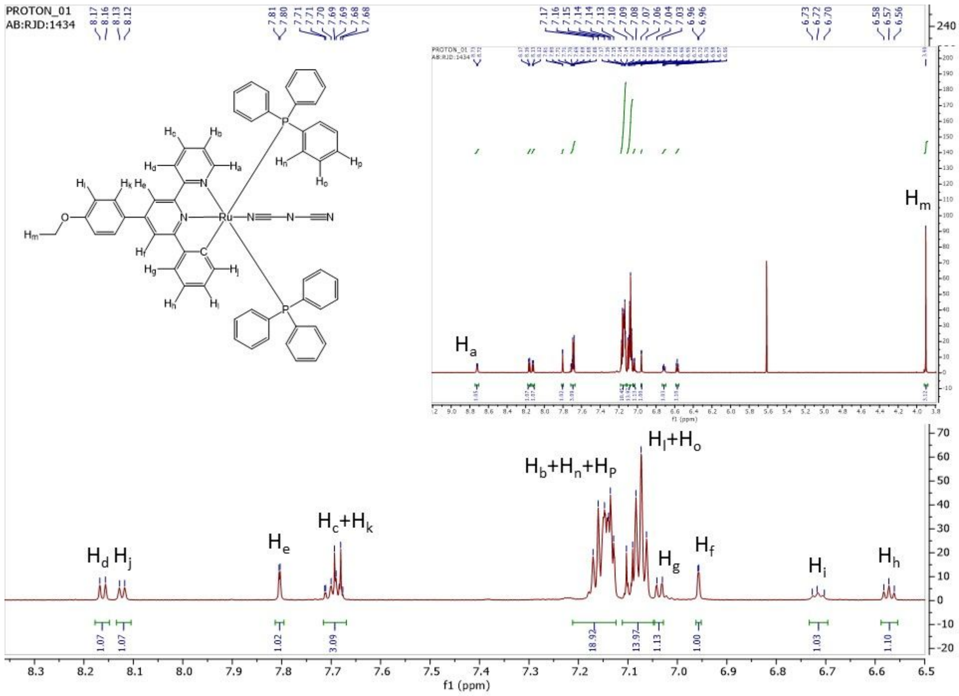

Figure S23a. ^{1}H NMR spectrum of **3c** recorded in Acetone-d_6.

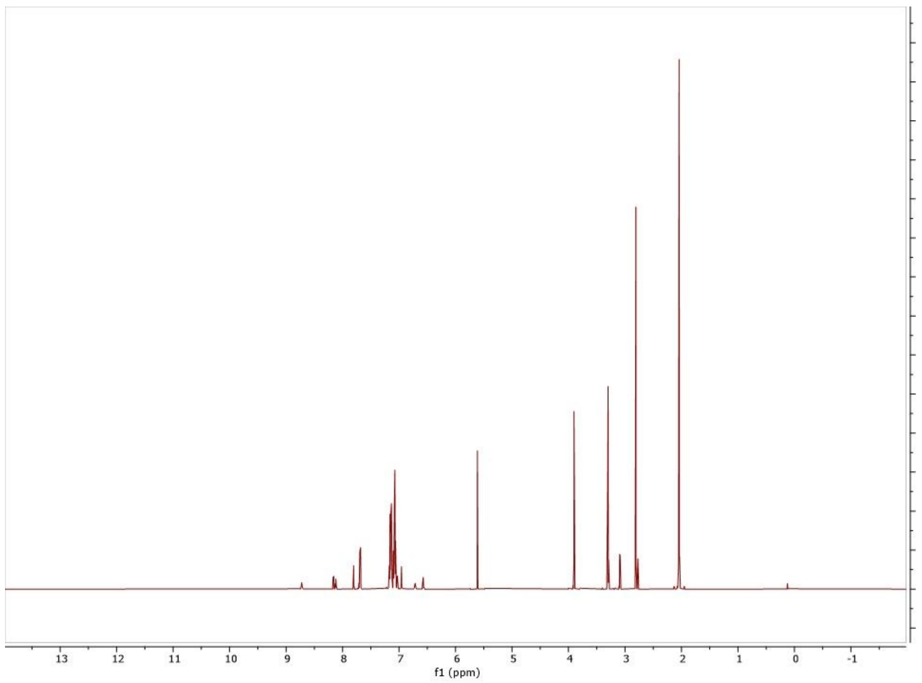

Figure S23b. ^{1}H NMR spectrum of **3c** recorded in Acetone-d$_6$.

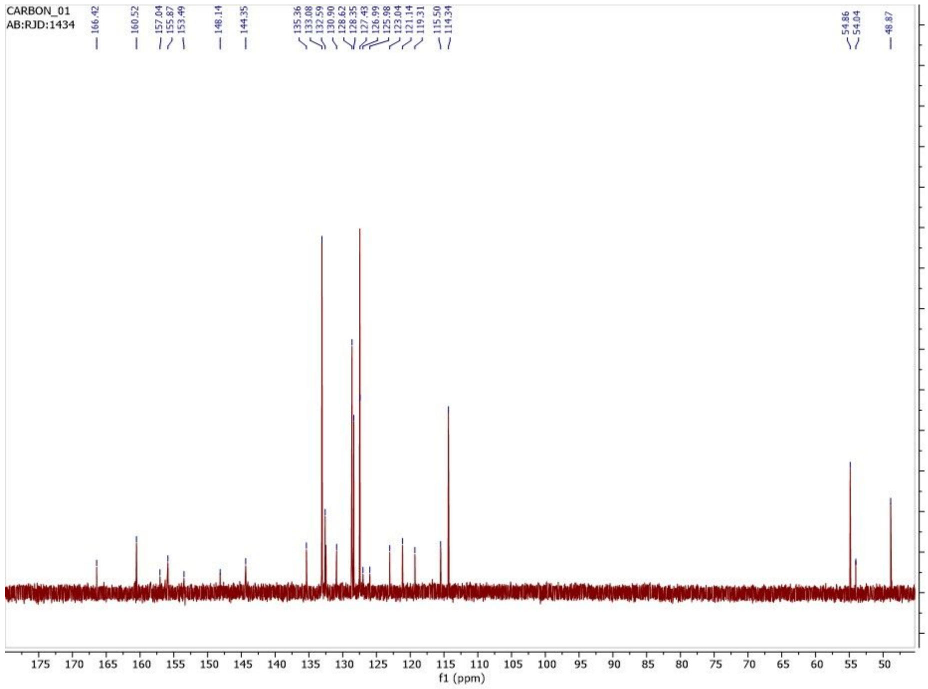

Figure S24a. ^{13}C{^{1}H} NMR spectrum of **3c** recorded in Acetone-d$_6$.

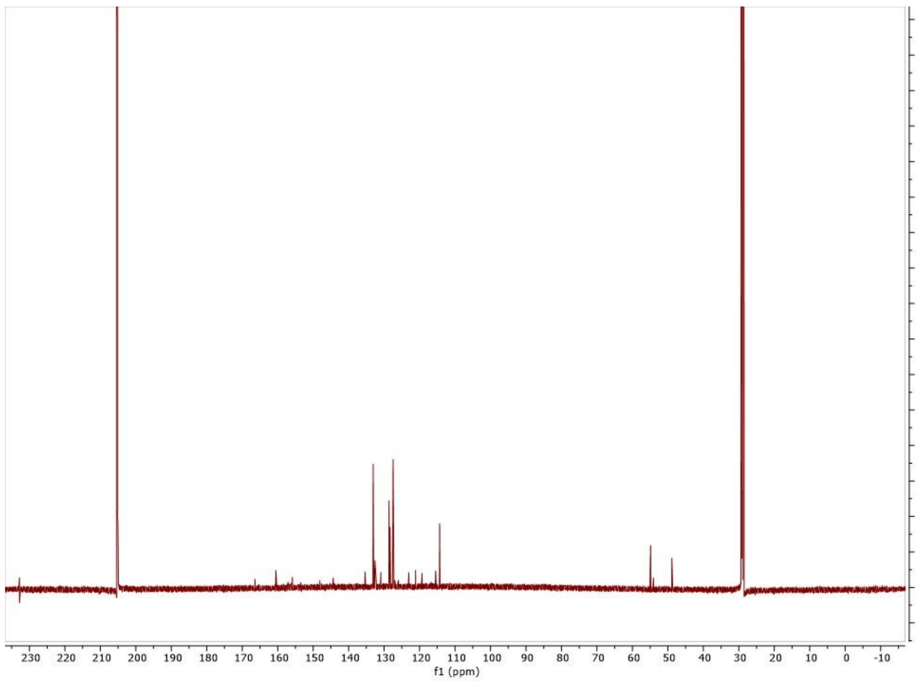

Figure S24b. $^{13}C\{^{1}H\}$ NMR spectrum of **3c** recorded in Acetone-d_6.

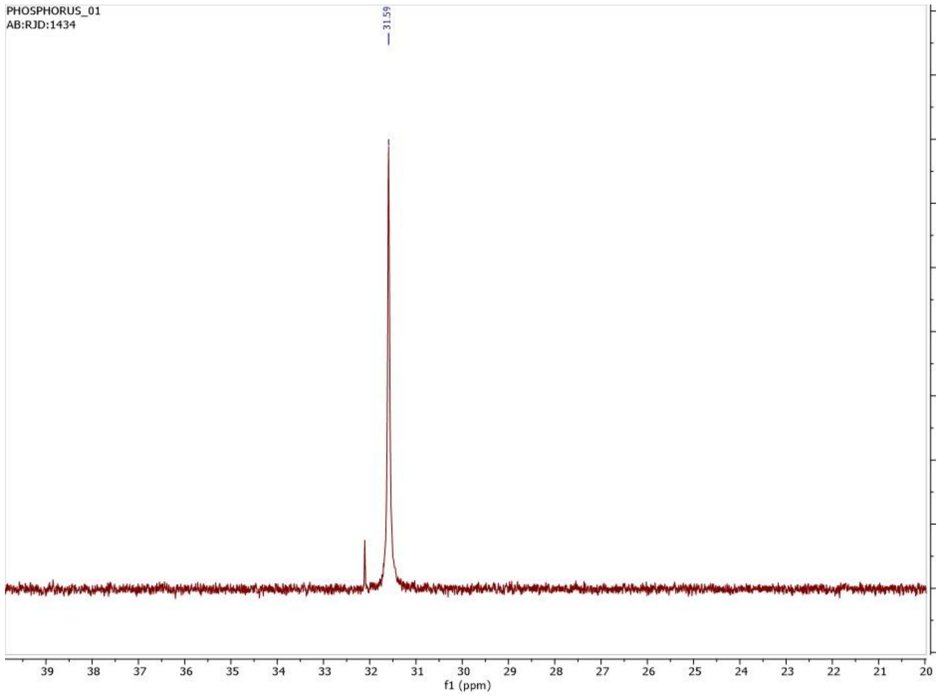

Figure S25. $^{31}P\{^{1}H\}$ NMR spectrum of **3c** recorded in Acetone-d_6.

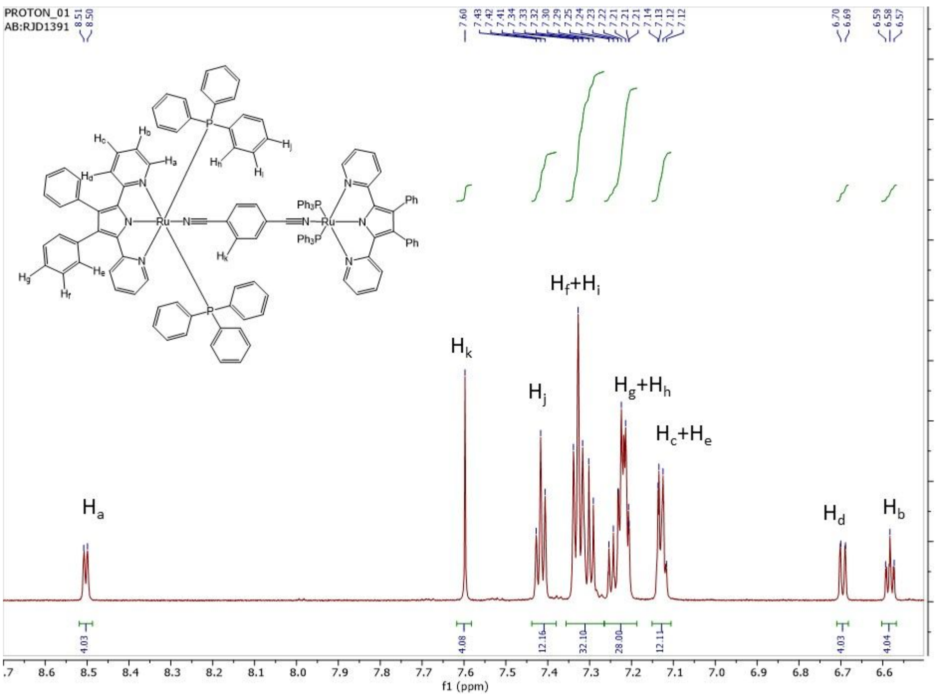

Figure S26a. ^{1}H NMR spectrum of **4a** recorded in Acetone-d_6.

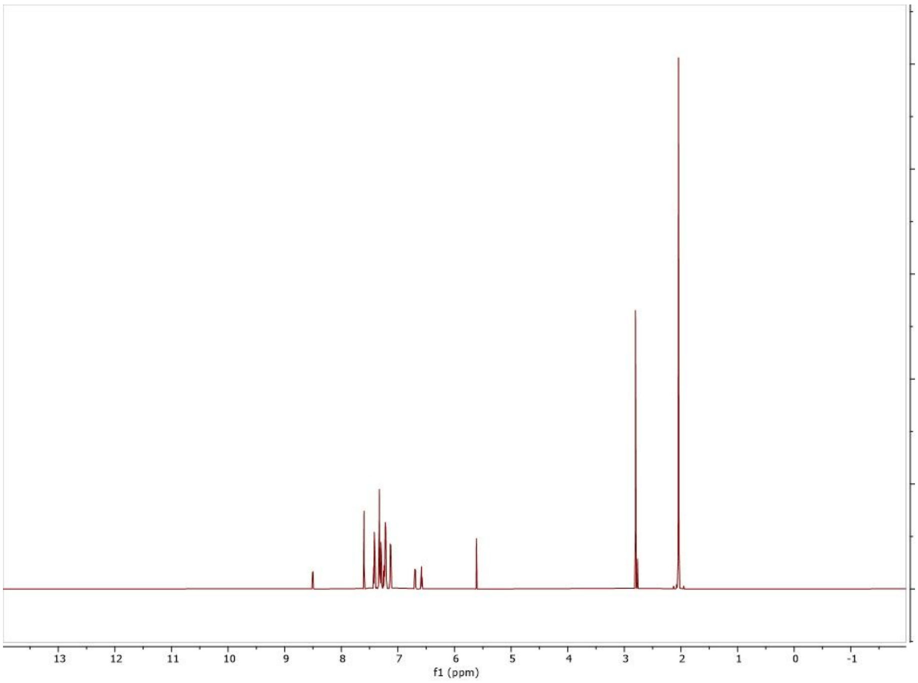

Figure S26b. ^{1}H NMR spectrum of **4a** recorded in Acetone-d_6.

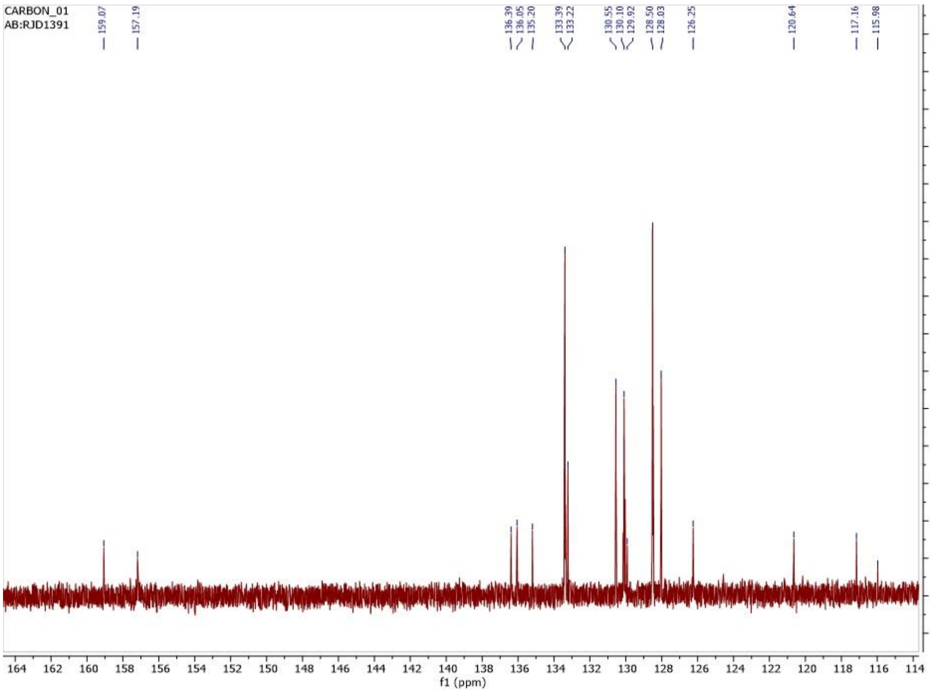

Figure S27a. $^{13}C\{^{1}H\}$ NMR spectrum of **4a** recorded in Acetone-d$_6$.

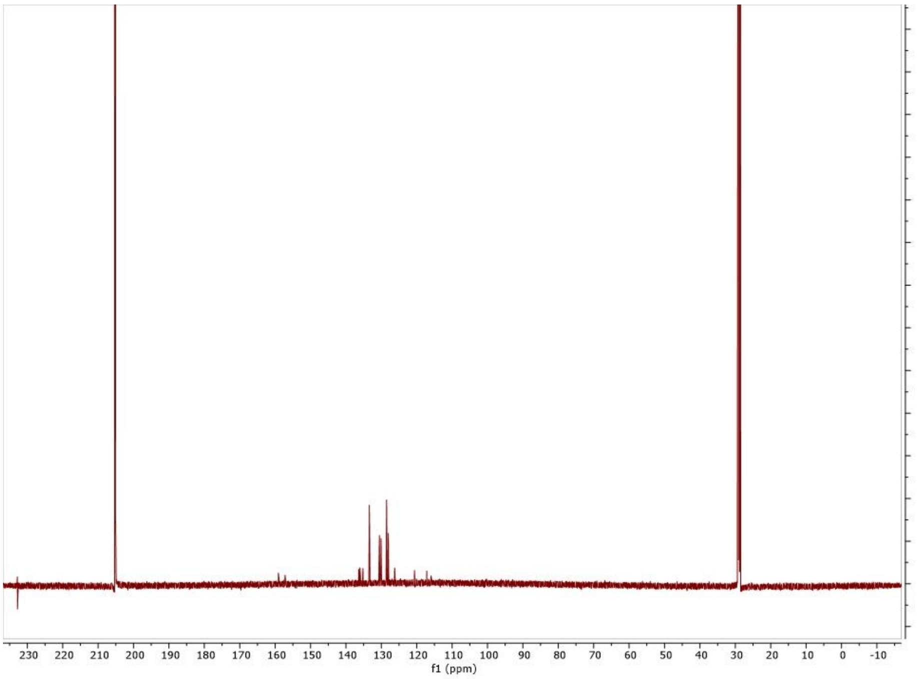

Figure S27b. $^{13}C\{^{1}H\}$ NMR spectrum of **4a** recorded in Acetone-d$_6$.

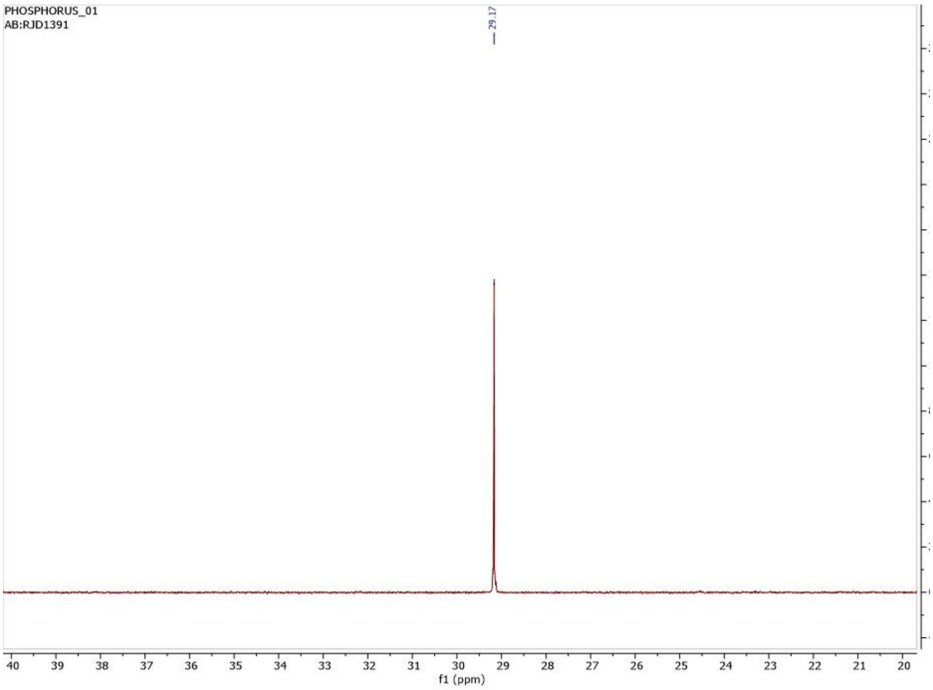

Figure S28. $^{31}P\{^{1}H\}$ NMR spectrum of **4a** recorded in Acetone-d_6.

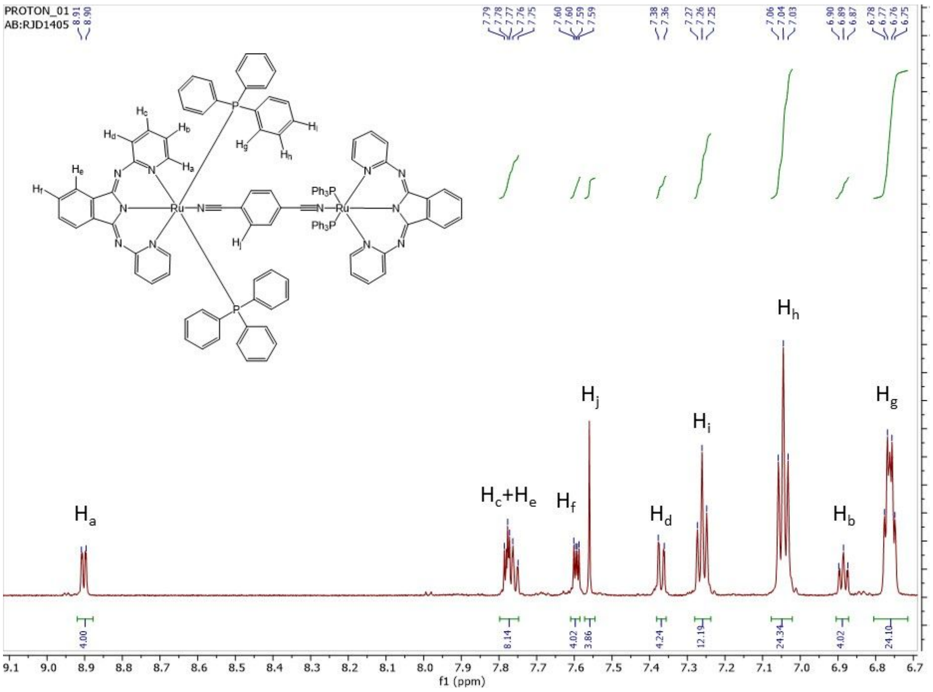

Figure S29a. ^{1}H NMR spectrum of **4b** recorded in TCE-d_2.

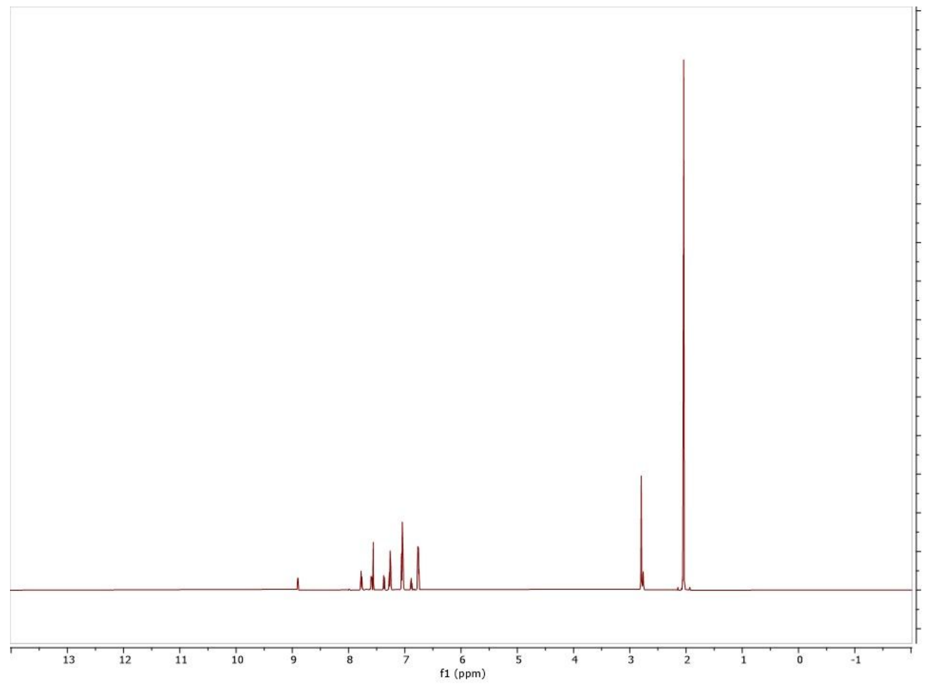

Figure S29b. ^{1}H NMR spectrum of **4b** recorded in TCE-d_2.

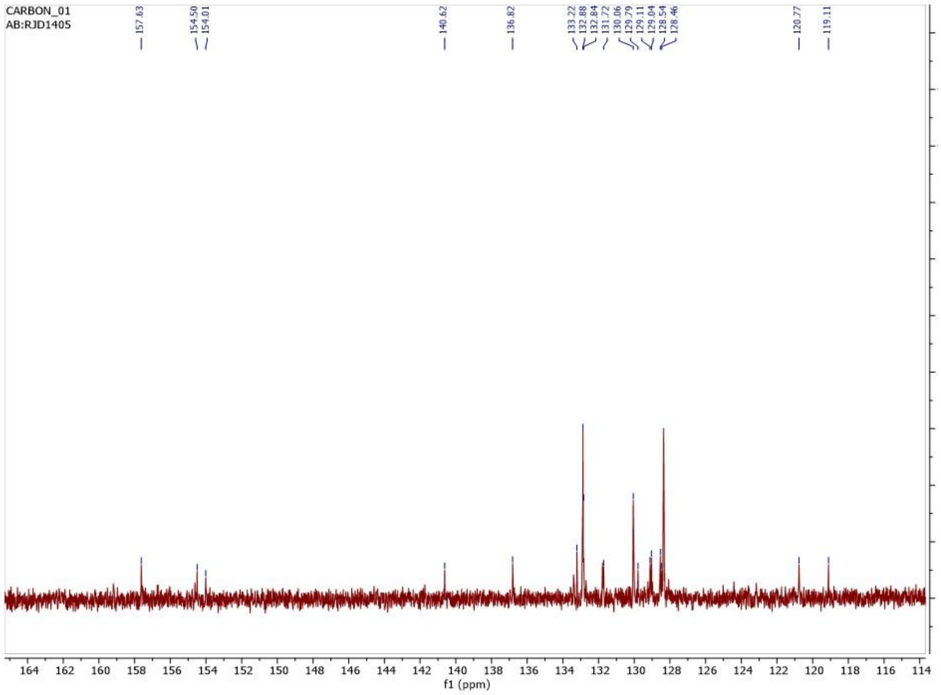

Figure S30a. $^{13}C\{^{1}H\}$ NMR spectrum of **4b** recorded in TCE-d_2.

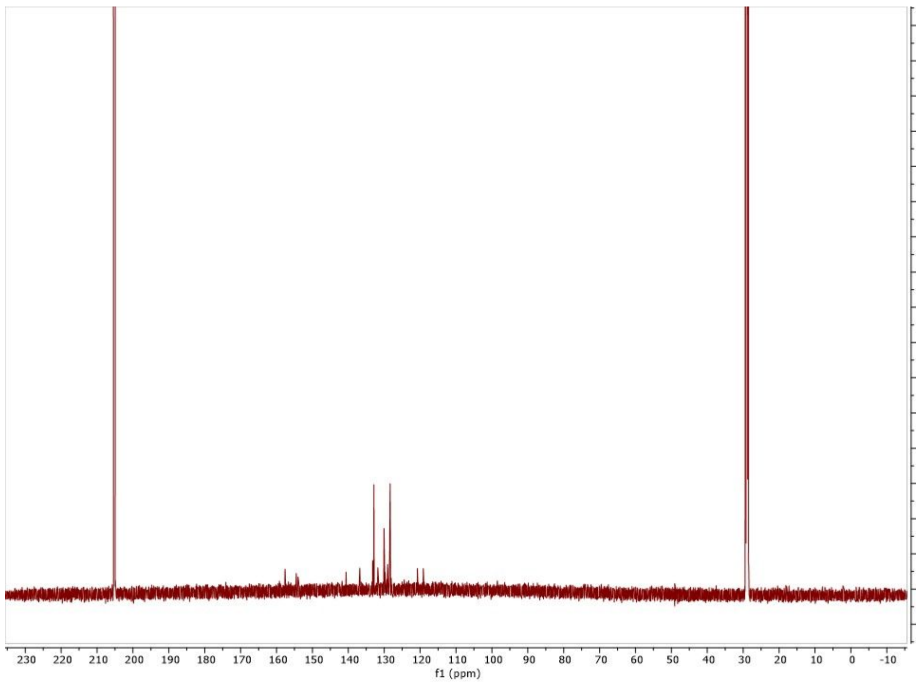

Figure S30b. $^{13}C\{^{1}H\}$ NMR spectrum of **4b** recorded in TCE-d$_2$.

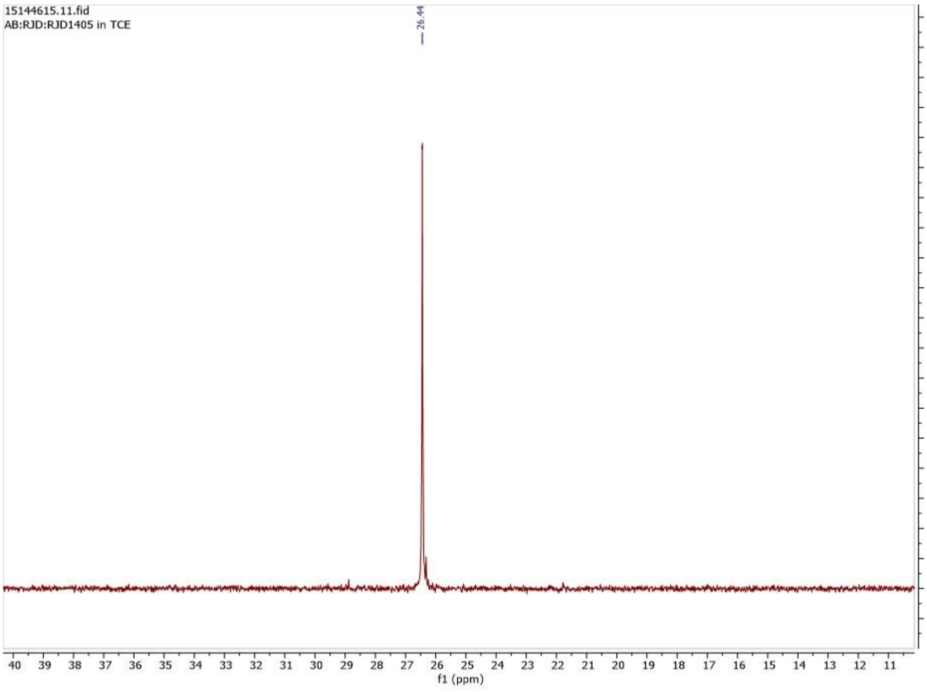

Figure S31. $^{31}P\{^{1}H\}$ NMR spectrum of **4b** recorded in TCE-d$_2$.

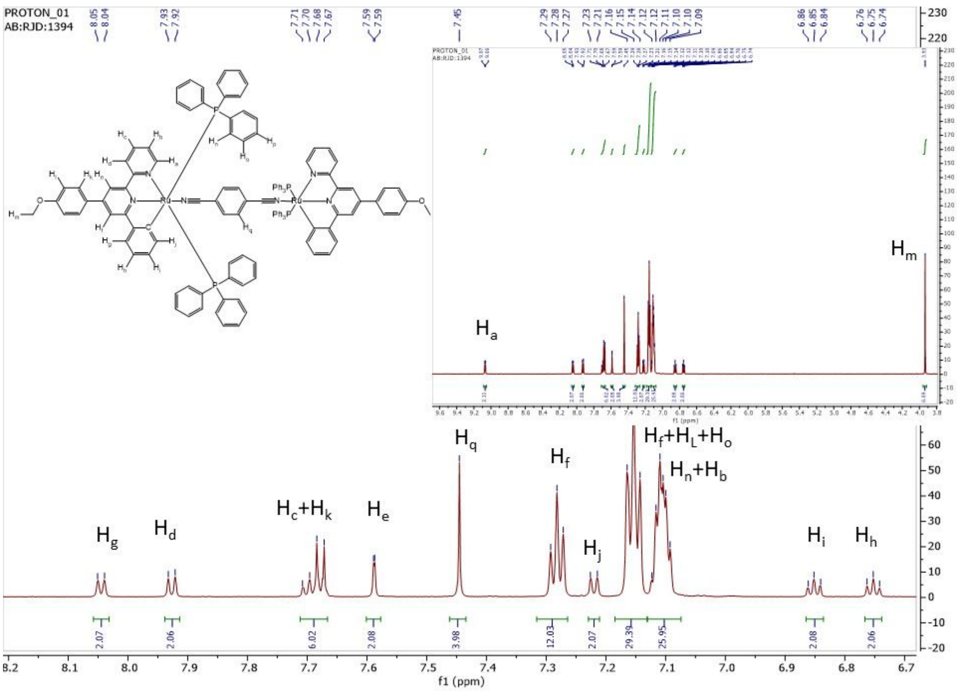

Figure S32a. ^{1}H NMR spectrum of **4c** recorded in CD_3CN.

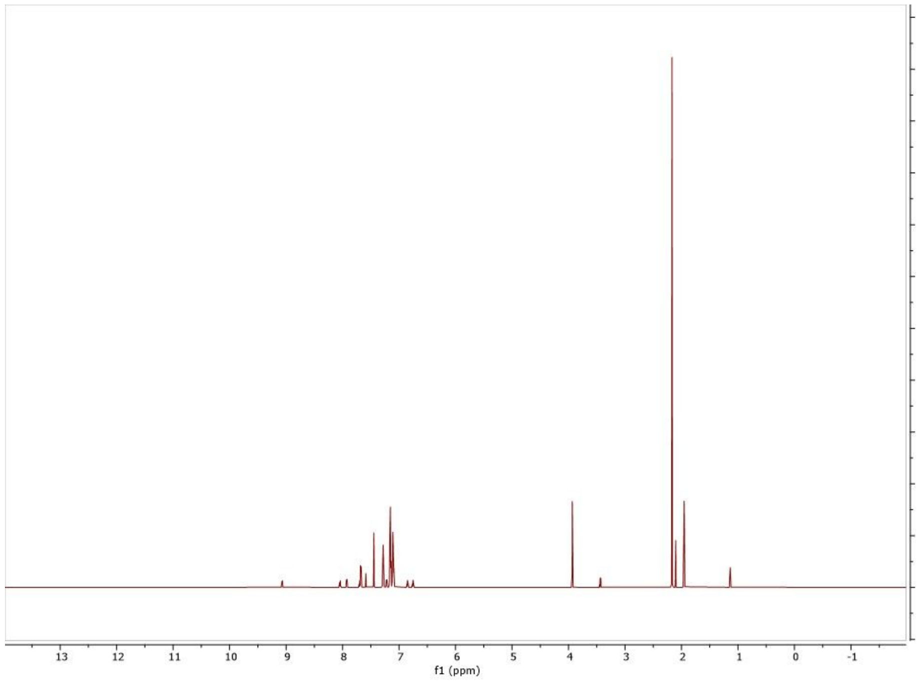

Figure S32b. ^{1}H NMR spectrum of **4c** recorded in CD_3CN.

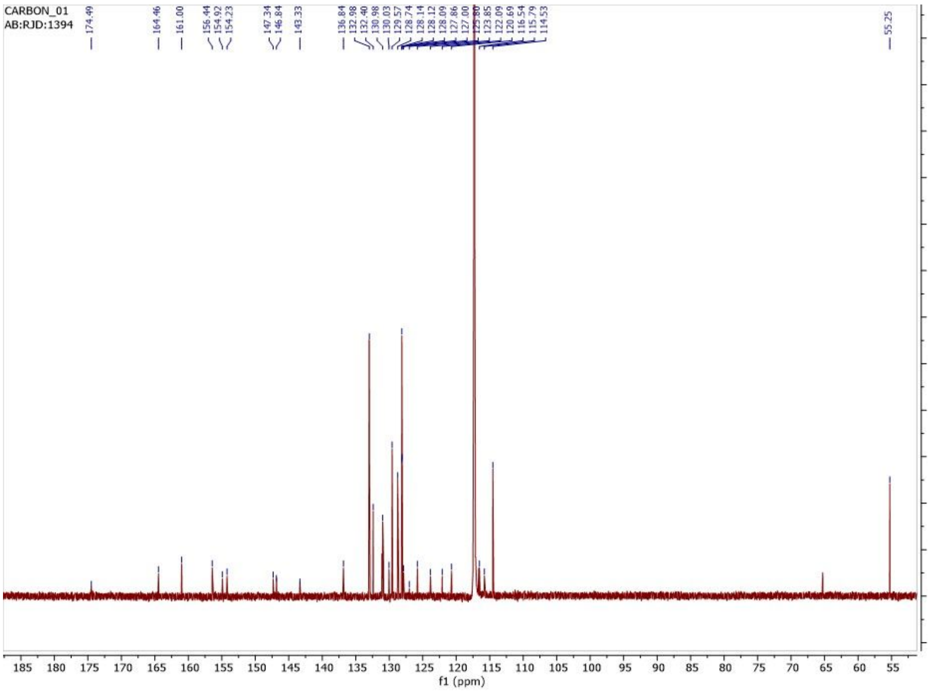

Figure S33a. $^{13}C\{^1H\}$ NMR spectrum of **4c** recorded in CD_3CN.

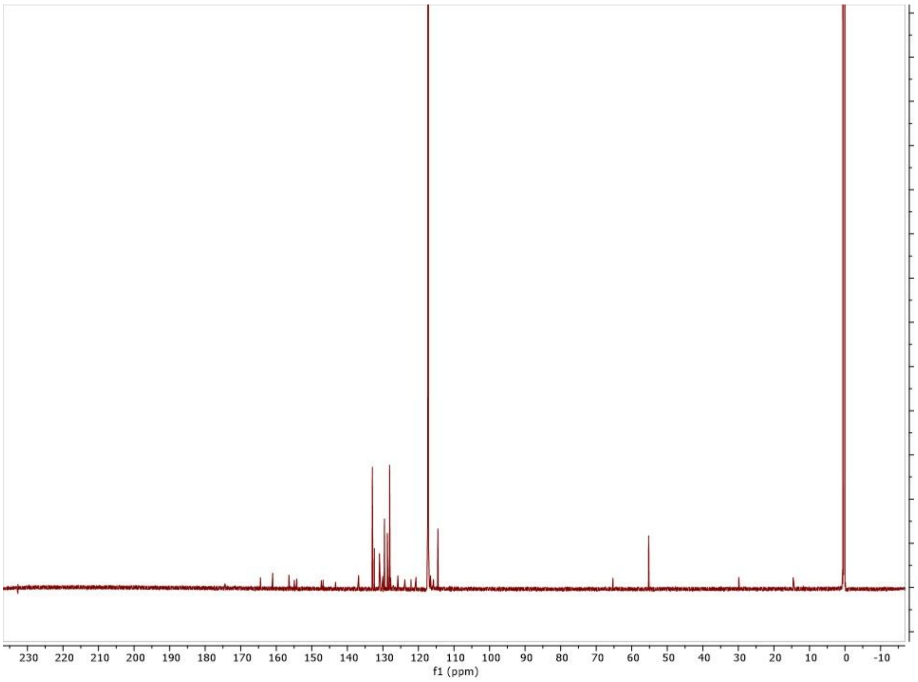

Figure S33b. $^{13}C\{^1H\}$ NMR spectrum of **4c** recorded in CD_3CN.

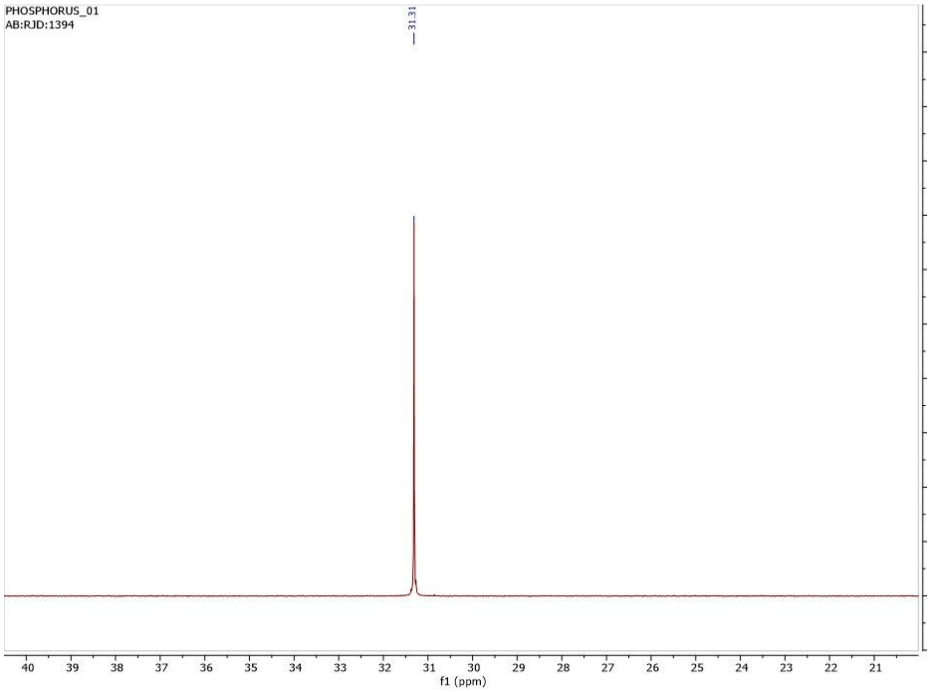

Figure S34. $^{31}P\{^{1}H\}$ NMR spectrum of **4c** recorded in CD_3CN.

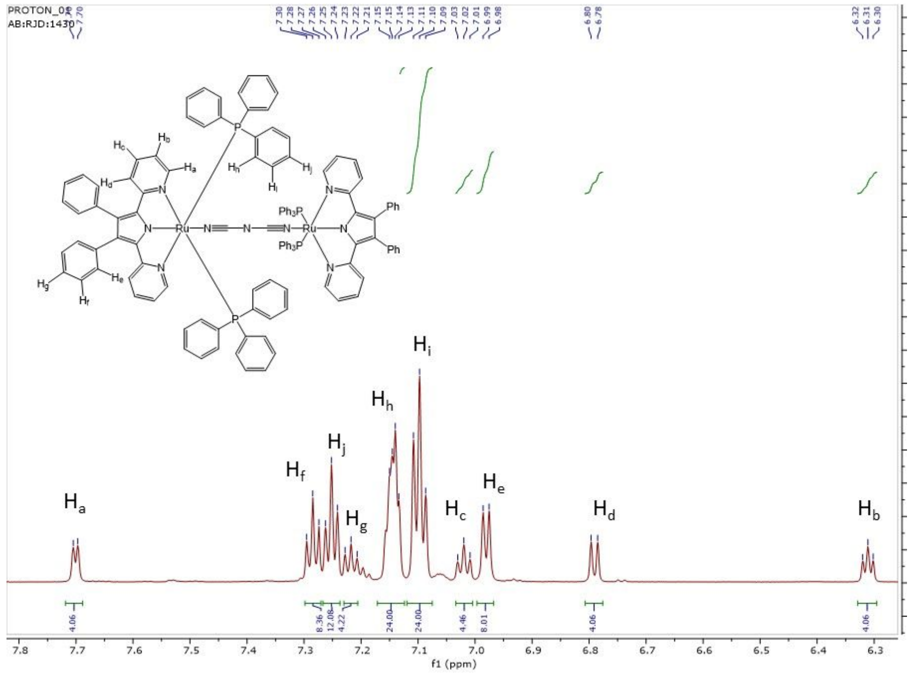

Figure S35a. ^{1}H NMR spectrum of **5a** recorded in TCE-d_2.

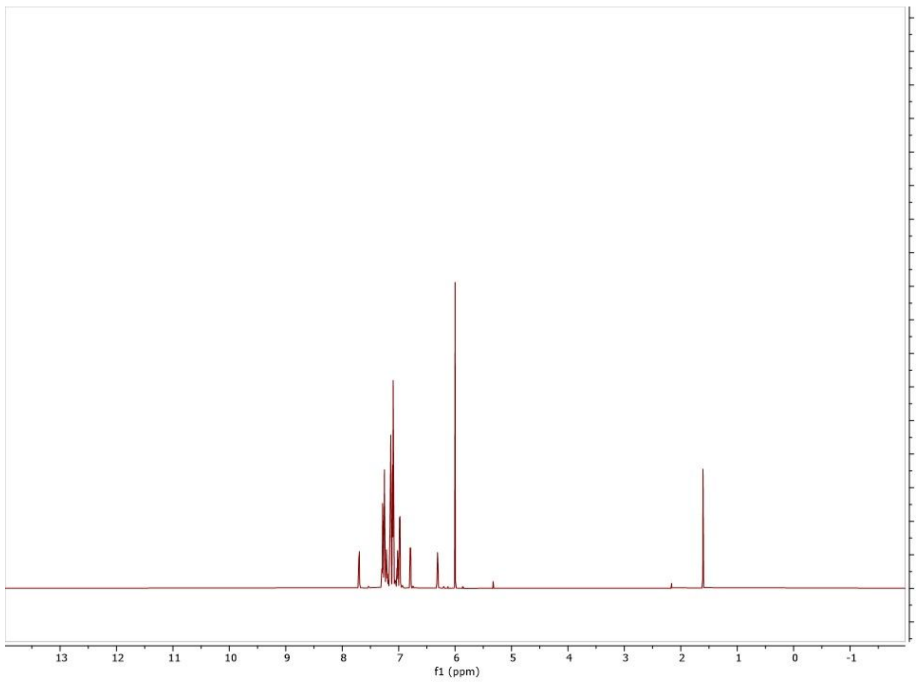

Figure S35b. ^{1}H NMR spectrum of **5a** recorded in TCE-d_2.

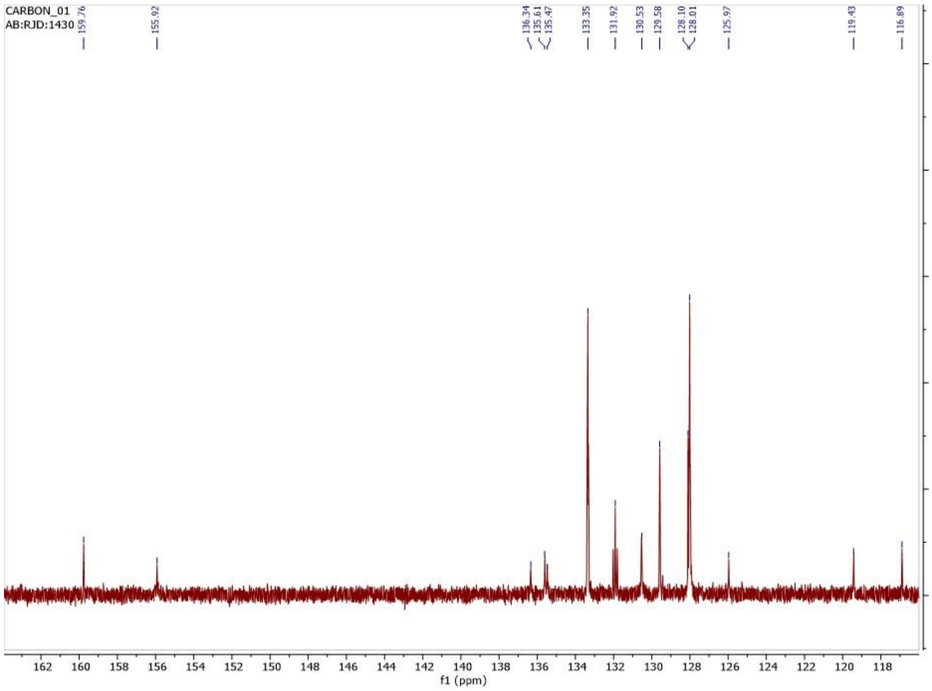

Figure S36a. $^{13}C\{^{1}H\}$ NMR spectrum of **5a** recorded in TCE-d_2.

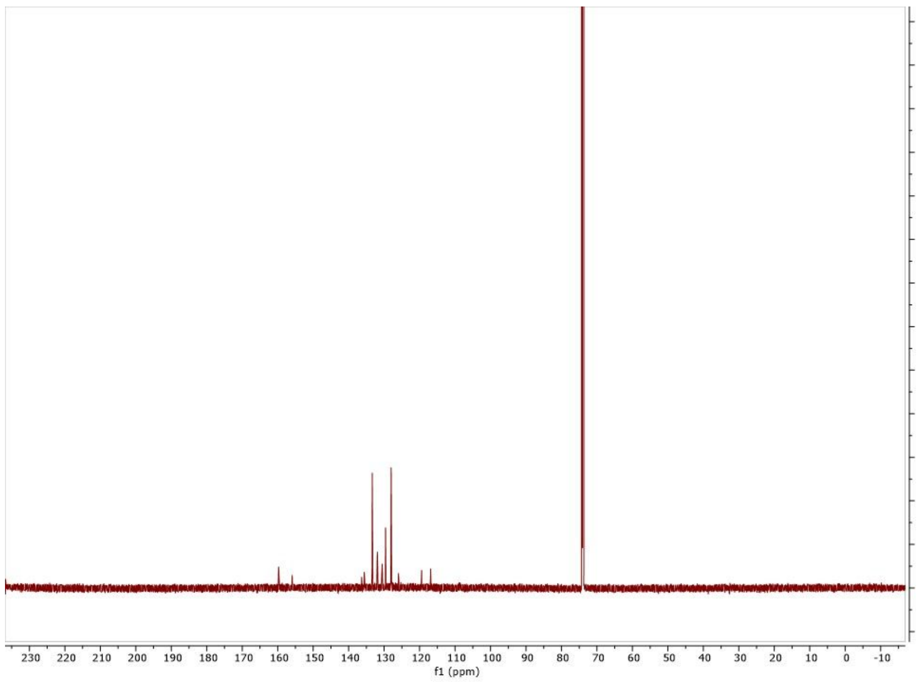

Figure S36b. $^{13}C\{^{1}H\}$ NMR spectrum of **5a** recorded in TCE-d$_2$.

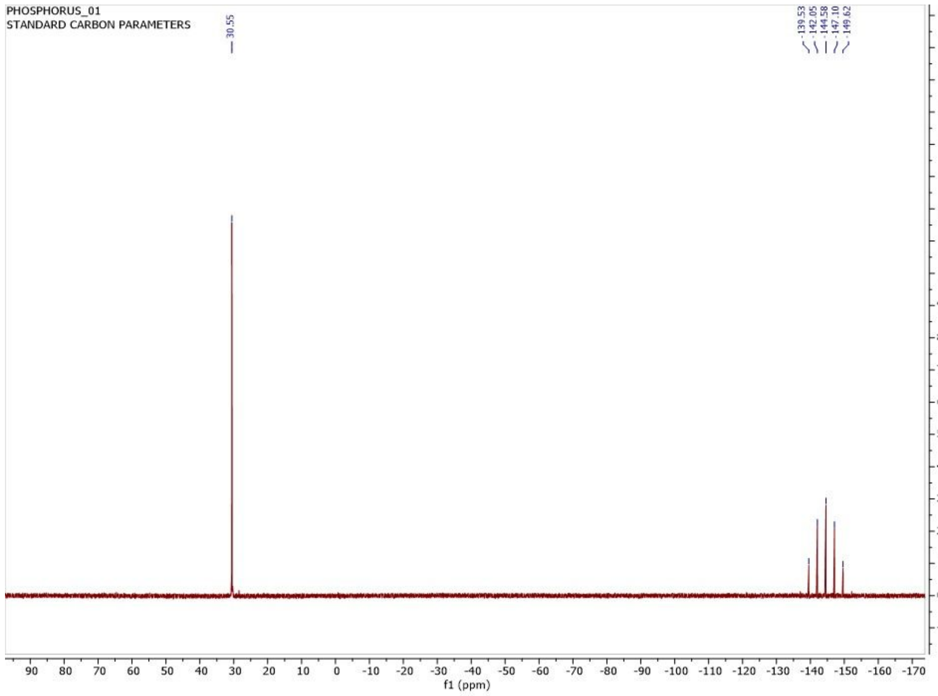

Figure S37. $^{31}P\{^{1}H\}$ NMR spectrum of **5a** recorded in TCE-d$_2$.

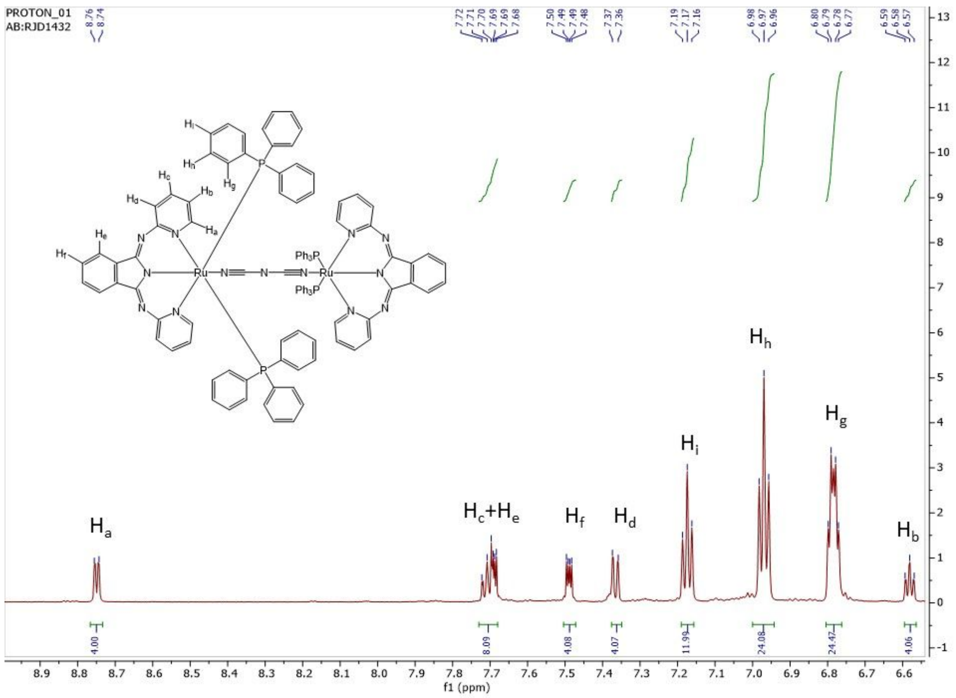

Figure S38a. ^{1}H NMR spectrum of **5b** recorded in Acetone-d$_6$.

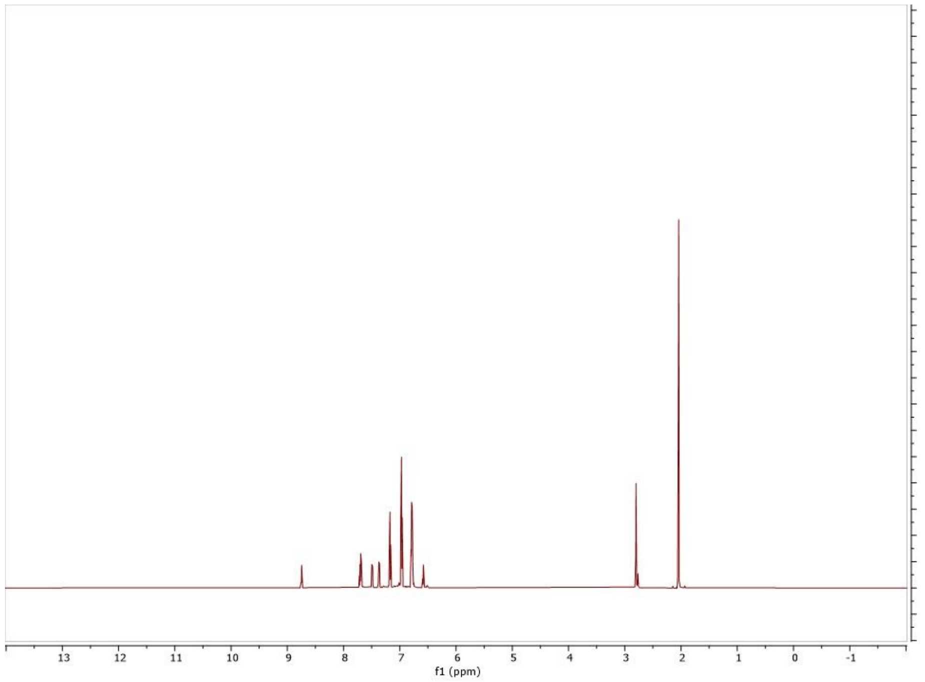

Figure S38b. ^{1}H NMR spectrum of **5b** recorded in Acetone-d$_6$.

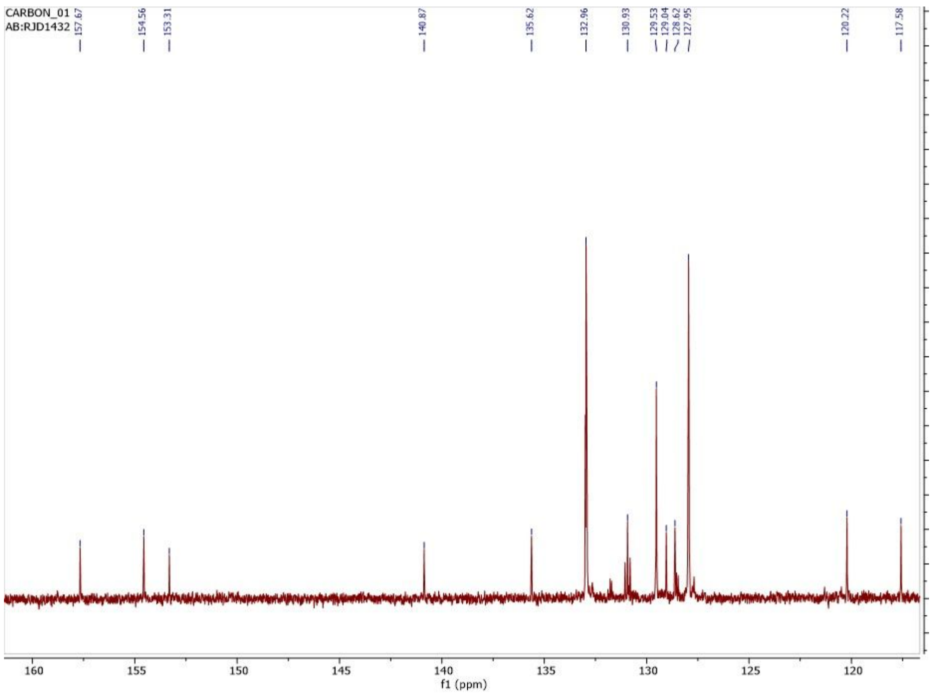

Figure S39a. $^{13}C\{^{1}H\}$NMR spectrum of **5b** recorded in Acetone-d_6.

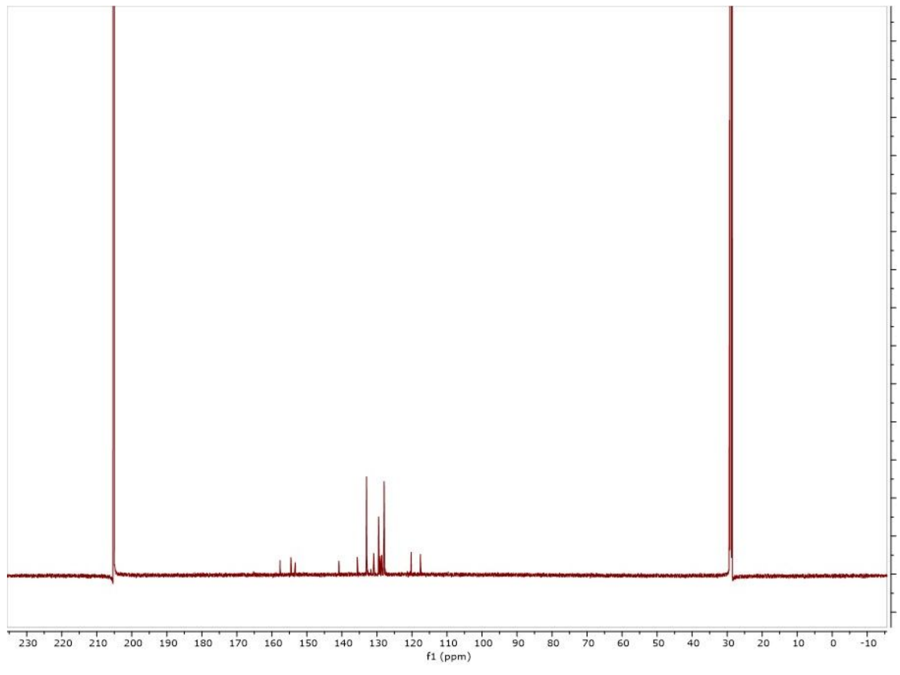

Figure S39b. $^{13}C\{^{1}H\}$NMR spectrum of **5b** recorded in Acetone-d_6.

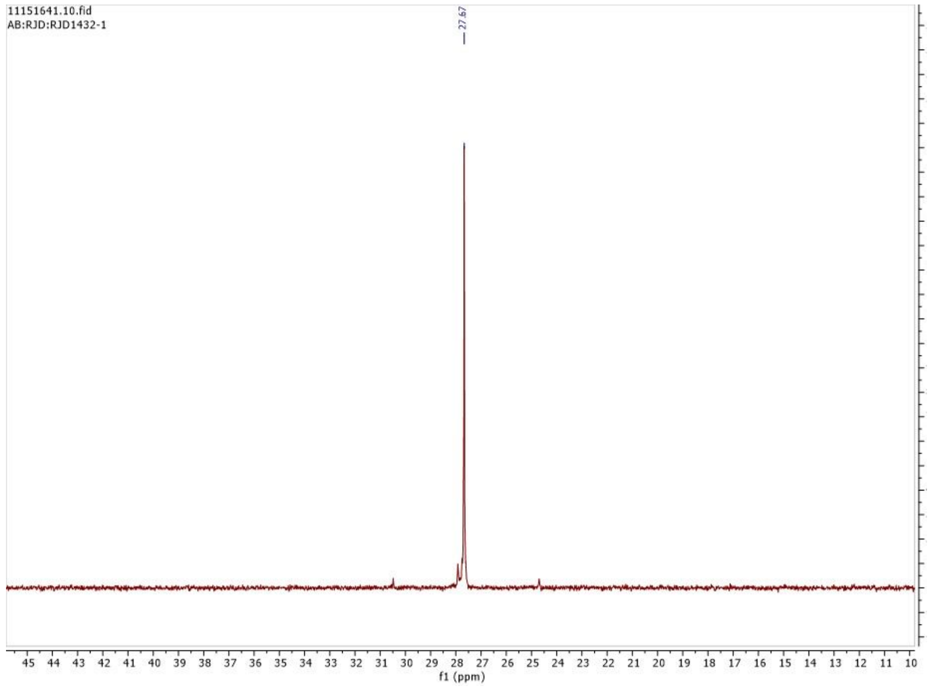

Figure S40. $^{31}P\{^{1}H\}$ NMR spectrum of **5b** recorded in Acetone-d_6.

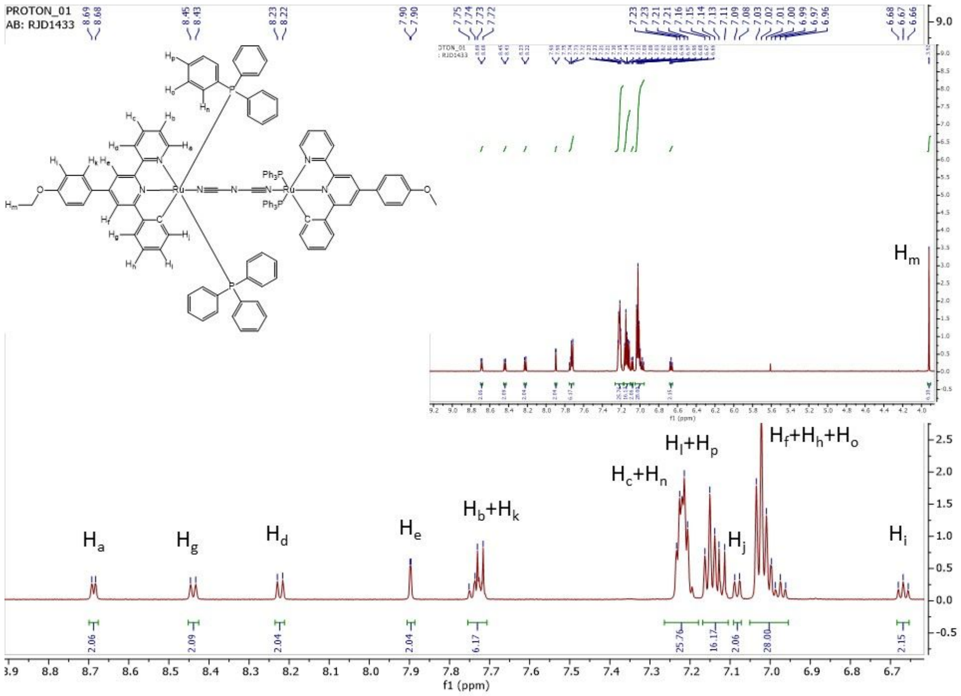

Figure S41a. ^{1}H NMR spectrum of **5c** recorded in Acetone-d_6.

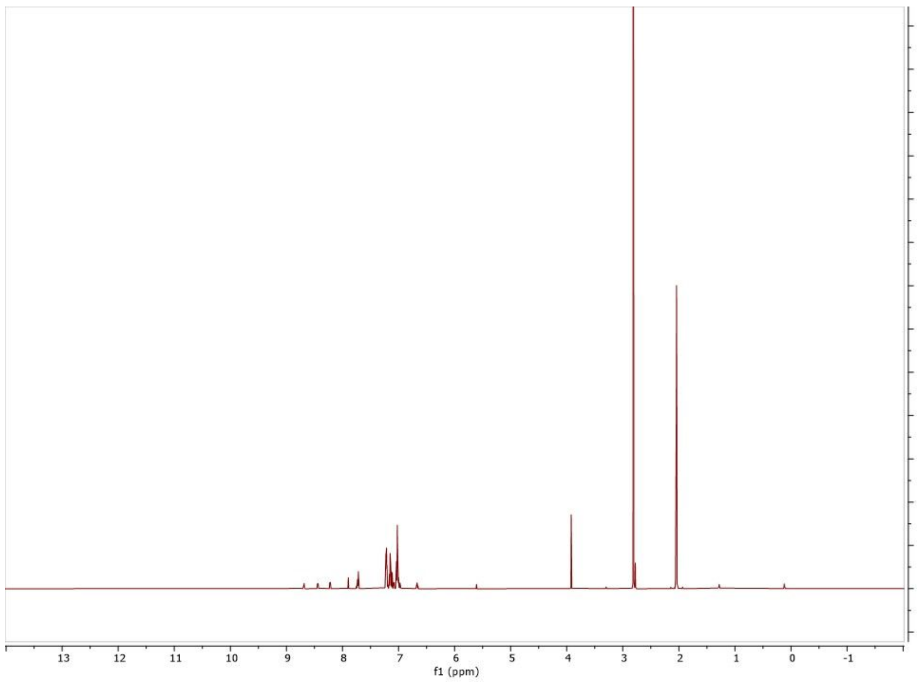

Figure S41b. ^{1}H NMR spectrum of **5c** recorded in Acetone-d_6.

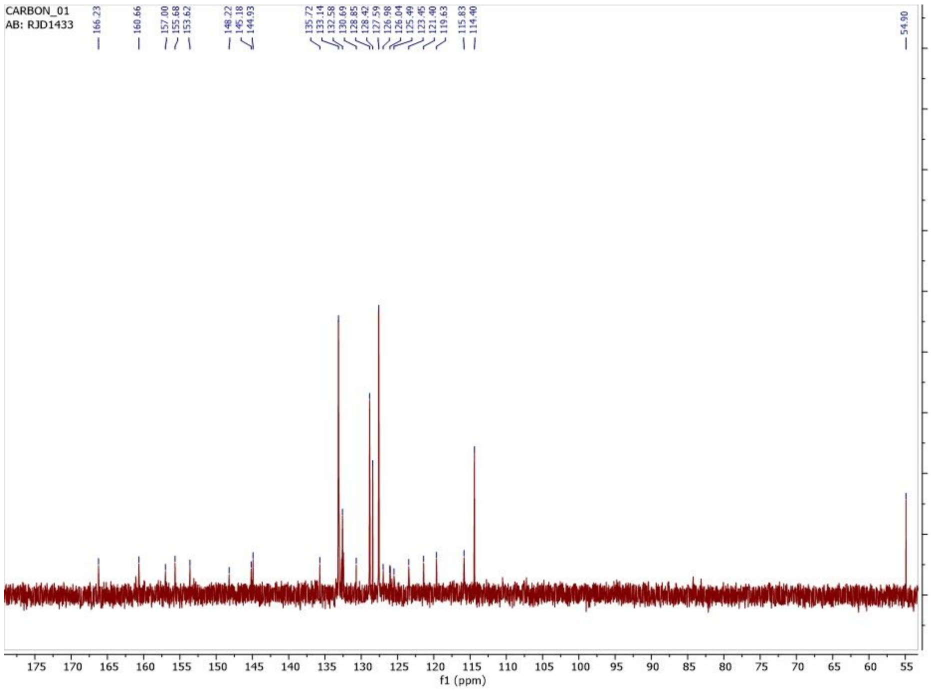

Figure S42a. $^{13}C\{^{1}H\}$ NMR spectrum of **5c** recorded in Acetone-d_6.

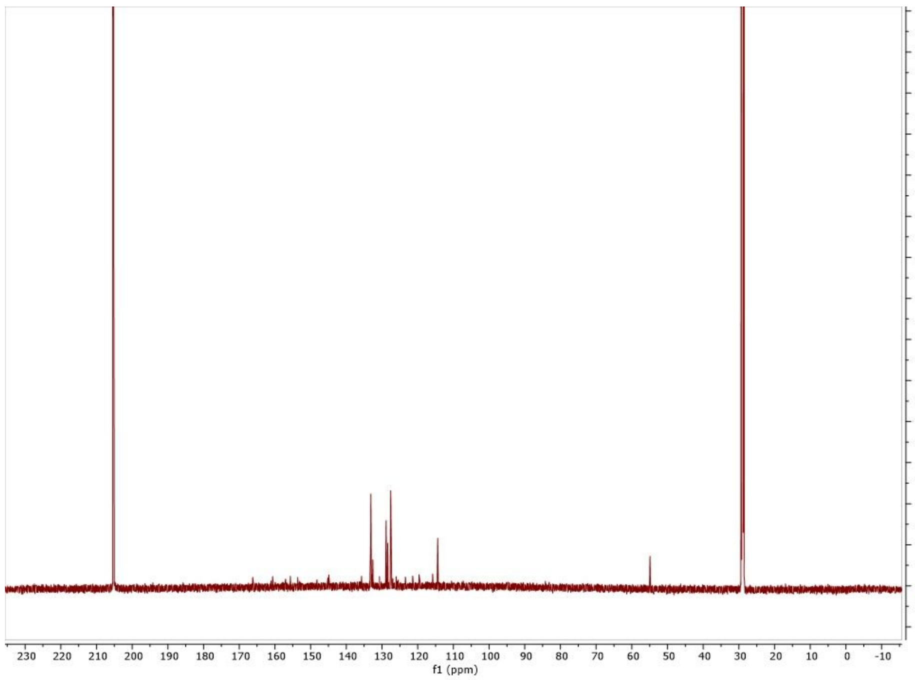

Figure S42b. $^{13}C\{^1H\}$ NMR spectrum of **5c** recorded in Acetone-d_6.

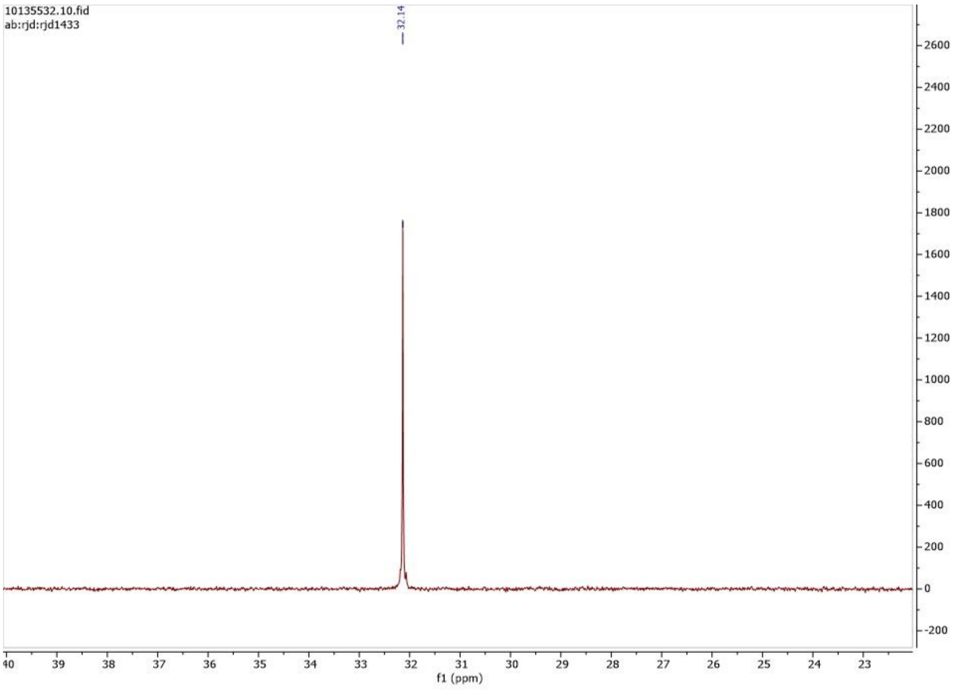

Figure S43. $^{31}P\{^1H\}$ NMR spectrum of **5c** recorded in Acetone-d_6.

S3. X-ray Crystallography

The X-ray single crystal data have been collected using λMoKα radiation (λ =0.71073Å) on an Agilent XCalibur (Sapphire-3 CCD detector, fine-focus sealed tube, graphite monochromator; compounds **2b** and **2c**) and Bruker D8Venture (Photon100 CMOS detector, IμS-microsource, focusing mirrors; all other compounds) diffractometers equipped with a Cryostream (Oxford Cryosystems) open-flow nitrogen cryostats at 120.0(2)K. All structures were solved by direct method and refined by full-matrix least squares on F^2 for all data using Olex2[1] and SHELXTL[2] software. All non-disordered non-hydrogen atoms were refined in anisotropic approximation, hydrogen atoms were placed in the calculated positions and refined in riding mode. Terminal groups in some studied structures and solvent molecules in most of them were found to be disordered. Disordered atoms were refined isotropically with various fixed SOF and some restraints (mainly SADI) were applied where it deemed to be necessary. Crystal data and parameters of refinement are listed in Tables S1-S5. Crystallographic data for the structure have been deposited with the Cambridge Crystallographic Data Centre as supplementary publication CCDC 2205853 – 2205864, 2368041, and 2359149.

Table S1. Crystal data and structure refinement for structures **Cla and Clc**.

	**Cla**	**Clc**
Empirical formula	$C_{62}H_{48}ClN_3P_2Ru \times CH_2Cl_2$	$C_{59}H_{47}ClN_2OP_2Ru \times 1.5\ CH_2Cl_2$
Formula weight	1118.42	1125.83
Temperature/K	120.0	120.0
Crystal system	triclinic	monoclinic
Space group	P-1	$P2_1/n$
a/Å	12.2959(5)	14.6644(9)
b/Å	12.6766(5)	24.7084(15)
c/Å	18.8666(8)	14.7573(9)
α/°	84.0866(15)	90.00
β/°	85.2445(15)	102.238(2)
γ/°	61.0110(13)	90.00
Volume/Å^3	2556.78(18)	5225.6(6)
Z	2	4
ρ_{calc}g/cm^3	1.453	1.431
μ/mm^{-1}	0.572	0.610
F(000)	1148.0	2308.0
Radiation	MoKα (λ = 0.71000)	MoKα (λ = 0.71073)
2Θ range for data collection/°	4.18 to 58	4.34 to 54
Index ranges	$-16 \leq h \leq 16$, $-17 \leq k \leq 17$, $-25 \leq l \leq 25$	$-18 \leq h \leq 18$, $-31 \leq k \leq 31$, $-18 \leq l \leq 18$
Reflections collected	53415	71069
Independent reflections	13607 [R_{int} = 0.0344, R_{sigma} = 0.0355]	11401 [R_{int} = 0.0954, R_{sigma} = 0.0799]
Data/restraints/parameters	13607/6/666	11401/15/612
Goodness-of-fit on F^2	1.022	1.102
Final R indexes [I>=2σ (I)]	R_1 = 0.0428, wR_2 = 0.1041	R_1 = 0.0860, wR_2 = 0.1819
Final R indexes [all data]	R_1 = 0.0586, wR_2 = 0.1131	R_1 = 0.1308, wR_2 = 0.2002

Table S2. Crystal data and structure refinement for structures **1a**, **1c**, and **2a**.

Identification code	**1a**	**1c**	**2a**
Empirical formula	$C_{70}H_{52}N_5P_2Ru$ × PF_6	$C_{67}H_{51}N_4OP_2Ru$ × PF_6 × 2 CH_2Cl_2	$C_{71}H_{52}N_4P_3Ru$ × PF_6 × 0.5 CH_2Cl_2
Formula weight	1271.15	1405.95	1312.62
Temperature/K	120.0	120.0	120.0
Crystal system	orthorhombic	triclinic	triclinic
Space group	$Pna2_1$	P-1	P-1
a/Å	20.3395(9)	13.5351(9)	15.8190(9)
b/Å	13.8586(6)	14.6267(9)	18.2465(10)
c/Å	20.2982(9)	18.2792(12)	22.9807(13)
α/°	90.00	106.270(2)	107.9808(17)
β/°	90.00	93.328(2)	103.1179(17)
γ/°	90.00	114.342(2)	92.7074(19)
Volume/Å^3	5721.6(4)	3102.1(3)	6095.1(6)
Z	4	2	4
ρ_{calc}g/cm^3	1.476	1.505	1.430
μ/mm^{-1}	0.427	0.569	0.446
F(000)	2600.0	1432.0	2684.0
Radiation	MoKα (λ = 0.71073)	MoKα (λ = 0.71073)	MoKα (λ = 0.71073)
2Θ range for data collection/°	4.48 to 60	4.44 to 58	3.8 to 54
Index ranges	-28 ≤ h ≤ 28, -19 ≤ k ≤ 19, -28 ≤ l ≤ 28	-18 ≤ h ≤ 18, -19 ≤ k ≤ 19, -24 ≤ l ≤ 24	-20 ≤ h ≤ 20, -23 ≤ k ≤ 23, -29 ≤ l ≤ 29
Reflections collected	92545	64564	111223
Independent reflections	16693 [R_{int} = 0.0630, R_{sigma} = 0.0554]	16473 [R_{int} = 0.0472, R_{sigma} = 0.0460]	26477 [R_{int} = 0.1718, R_{sigma} = 0.1978]
Data/restraints/parameters	16693/97/755	16473/72/818	26477/27/1332
Goodness-of-fit on F^2	1.047	1.020	1.003
Final R indexes [I>=2σ (I)]	R_1 = 0.0451, wR_2 = 0.0928	R_1 = 0.0381, wR_2 = 0.0846	R_1 = 0.0780, wR_2 = 0.1503
Final R indexes [all data]	R_1 = 0.0647, wR_2 = 0.1006	R_1 = 0.0546, wR_2 = 0.0922	R_1 = 0.1869, wR_2 = 0.1896

Table S3. Crystal data and structure refinement for structures **2b**, **2c**, and **3c**.

	**2b**	**2c**	**3c**
Empirical formula	$C_{63}H_{47}N_6P_2Ru \times PF_6$	$C_{68}H_{54}N_3OP_2Ru \times PF_6 \times 2\ CH_2Cl_2$	$C_{61}H_{47}N_5OP_2Ru$
Formula weight	1196.05	1404.96	1029.05
Temperature/K	120.0	120.0	120.0
Crystal system	triclinic	triclinic	triclinic
Space group	P-1	P-1	P-1
a/Å	10.4792(8)	13.7311(6)	11.7128(6)
b/Å	16.1602(10)	14.6261(7)	15.3632(8)
c/Å	16.4627(8)	18.3134(7)	15.4938(8)
α/°	87.504(5)	105.532(4)	97.7958(18)
β/°	75.438(5)	93.753(4)	111.4352(18)
γ/°	76.365(6)	115.148(4)	98.5832(19)
Volume/Å^3	2621.9(3)	3139.2(2)	2510.8(2)
Z	2	2	2
ρ_{calc}g/cm^3	1.515	1.486	1.361
μ/mm^{-1}	0.462	0.562	0.424
F(000)	1220.0	1432.0	1060.0
Radiation	MoKα (λ = 0.71073)	MoKα (λ = 0.71073)	MoKα (λ = 0.71073)
2Θ range for data collection/°	5.12 to 58	4.78 to 58	4.38 to 60
Index ranges	$-14 \le h \le 14$, $-22 \le k \le 22$, $-22 \le l \le 22$	$-18 \le h \le 18$, $-19 \le k \le 19$, $-24 \le l \le 24$	$-17 \le h \le 17$, $-22 \le k \le 22$, $-22 \le l \le 22$
Reflections collected	44262	39400	58900
Independent reflections	13934 [R_{int} = 0.0792, R_{sigma} = 0.0994]	16668 [R_{int} = 0.0384, R_{sigma} = 0.0560]	14631 [R_{int} = 0.0663, R_{sigma} = 0.0890]
Data/restraints/parameters	13934/91/724	16668/51/767	14631/0/632
Goodness-of-fit on F^2	0.999	1.029	1.043
Final R indexes [I>=2σ (I)]	R_1 = 0.0525, wR_2 = 0.0967	R_1 = 0.0571, wR_2 = 0.1344	R_1 = 0.0425, wR_2 = 0.0906
Final R indexes [all data]	R_1 = 0.0940, wR_2 = 0.1142	R_1 = 0.0744, wR_2 = 0.1471	R_1 = 0.0721, wR_2 = 0.0969

Table S4. Crystal data and structure refinement for structures **4a**, **4b**, and **4c**.

	**4a**	**4b**	**4c**
Empirical formula	$C_{132}H_{100}N_8P_4Ru_2$ × 2 PF_6 × 4 C_3H_6O × 0.5 $C_4H_{10}O$	$C_{116}H_{88}N_{12}P_4Ru$ × 2 PF_6 × CH_2Cl_2 × $C_4H_{10}O$	$C_{126}H_{98}N_6O_2P_4Ru_2$ × 2 PF_6 × 6 CH_2Cl_2
Formula weight	2683.52	2839.58	2853.62
Temperature/K	120.0	120.0	120.0
Crystal system	triclinic	triclinic	monoclinic
Space group	P-1	P-1	$P2_1/c$
a/Å	14.3386(10)	11.1268(7)	11.0244(8)
b/Å	20.1475(14)	15.6044(10)	19.7661(15)
c/Å	22.8558(16)	18.1746(11)	28.938(2)
α/°	96.429(2)	88.578(2)	90.00
β/°	93.701(2)	84.960(2)	90.487(3)
γ/°	100.496(2)	77.705(2)	90.00
Volume/$Å^3$	6427.0(8)	3071.2(3)	6305.7(8)
Z	2	1	2
$\rho_{calc}g/cm^3$	1.387	1.535	1.503
μ/mm^{-1}	0.387	0.661	0.643
F(000)	2766.0	1442.0	2900.0
Radiation	MoKα (λ = 0.71073)	MoKα (λ = 0.71073)	MoKα (λ = 0.71073)
2Θ range for data collection/°	4.306 to 58	4.216 to 57	4.36 to 56
Index ranges	$-18 \leq h \leq 19$, $-27 \leq k \leq 27$, $-31 \leq l \leq 31$	$-14 \leq h \leq 14$, $-20 \leq k \leq 20$, $-24 \leq l \leq 24$	$-14 \leq h \leq 14$, $-26 \leq k \leq 26$, $-38 \leq l \leq 38$
Reflections collected	135011	62066	121096
Independent reflections	34157 [R_{int} = 0.0614, R_{sigma} = 0.0700]	15536 [R_{int} = 0. 0490, R_{sigma} = 0. 0521]	15216 [R_{int} = 0.0558, R_{sigma} = 0.0359]
Data/restraints/parameters	34157/37/1609	15536/97/764	15216/45/772
Goodness-of-fit on F^2	1.030	1.012	1.023
Final R indexes [I>=2σ (I)]	R_1 = 0.0521, wR_2 = 0.1228	R_1 = 0.0676, wR_2 = 0.1620	R_1 = 0.0672, wR_2 = 0.1585
Final R indexes [all data]	R_1 = 0.0920, wR_2 = 0.1409	R_1 = 0.0948, wR_2 = 0.1776	R_1 = 0.0864, wR_2 = 0.1717

Table S5. Crystal data and structure refinement for structures **5a**, **5b**, and **5c**.

	**5a**	**5b**	**5c**
Empirical formula	$C_{126}H_{96}N_9P_4Ru_2$ × PF_6 × CH_3OH × 3 CH_2Cl_2	$C_{110}H_{84}N_{13}P_4Ru_2$ × PF_6 × 4 CH_3OH	$C_{120}H_{94}N_7O_2P_4Ru_2$ × PF_6
Formula weight	2493.93	2187.06	2137.01
Temperature/K	120.0	120.0	120.0
Crystal system	monoclinic	triclinic	triclinic
Space group	$P2_1/c$	P-1	P-1
a/Å	22.657(2)	15.4097(8)	12.9351(6)
b/Å	26.281(2)	17.6941(9)	19.0687(9)
c/Å	19.1541(18)	21.7118(11)	23.0997(10)
α/°	90.00	67.6098(18)	75.0712(15)
β/°	93.045(3)	86.109(2)	87.8681(15)
γ/°	90.00	74.2336(19)	84.4065(15)
Volume/$Å^3$	11389.5(18)	5263.5(5)	5478.7(4)
Z	4	2	2
$\rho_{calc}g/cm^3$	1.454	1.380	1.295
μ/mm^{-1}	0.544	0.433	0.412
F(000)	5104.0	2248.0	2192.0
Radiation	MoKα (λ = 0.71073)	MoKα (λ = 0.71073)	MoKα (λ = 0.71073)
2Θ range for data collection/°	3.76 to 56	4.22 to 56	4.38 to 56
Index ranges	$-29 \leq h \leq 29$, $-34 \leq k \leq 34$, $-25 \leq l \leq 25$	$-22 \leq h \leq 22$, $-26 \leq k \leq 26$, $-31 \leq l \leq 31$	$-19 \leq h \leq 19$, $-27 \leq k \leq 28$, $-34 \leq l \leq 34$
Reflections collected	218131	125334	133209
Independent reflections	27475 [R_{int} = 0.0998, R_{sigma} = 0.0758]	25385 [R_{int} = 0.0846, R_{sigma} = 0.1154]	26417 [R_{int} = 0.1144, R_{sigma} = 0.1626]
Data/restraints/parameters	27475/6/1431	25385/1/1304	26417/94/1244
Goodness-of-fit on F^2	1.050	1.104	1.105
Final R indexes [I>=2σ (I)]	R_1 = 0.0552, wR_2 = 0.1136	R_1 = 0.0504, wR_2 = 0.1335	R_1 = 0.0629, wR_2 = 0.1554
Final R indexes [all data]	R_1 = 0.1095, wR_2 = 0.1396	R_1 = 0.0849, wR_2 = 0.1447	R_1 = 0.1063, wR_2 = 0.1678

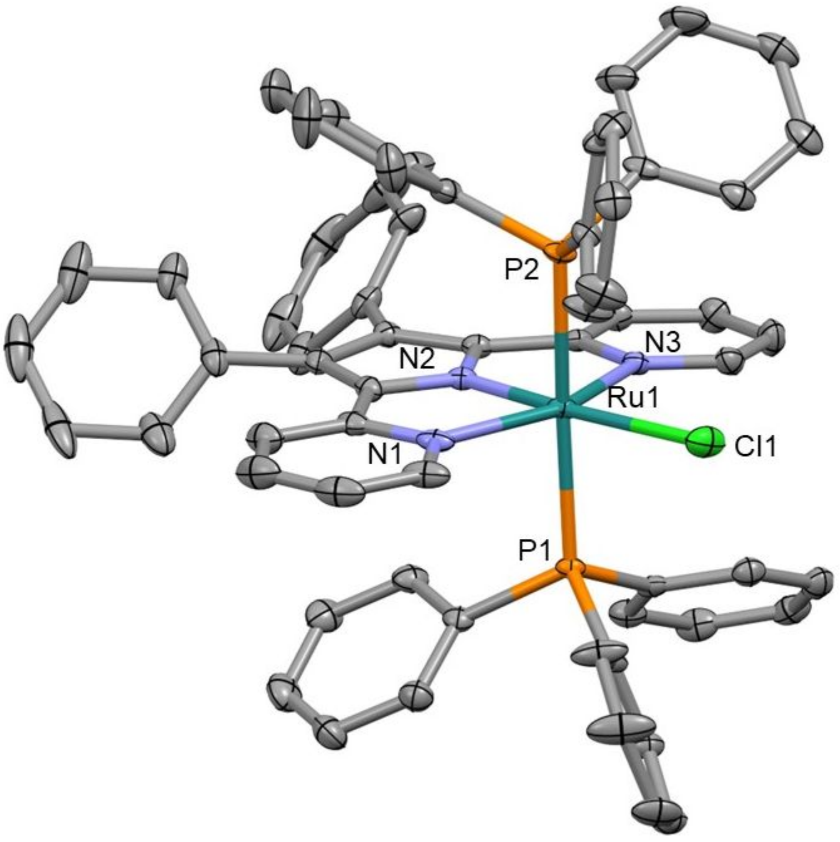

Figure S44. Crystal structure of **Cla**; solvent molecule, disorder removed for clarity, thermal ellipsoids displayed at 50% probability.

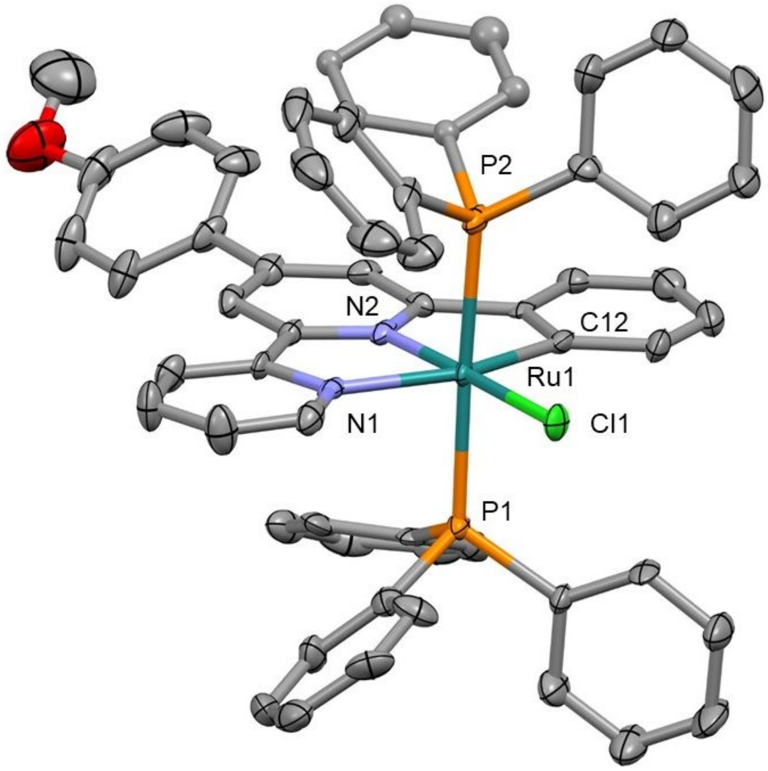

Figure S45. Crystal structure of **Clc**; solvent molecule, disorder removed for clarity, thermal ellipsoids displayed at 50% probability.

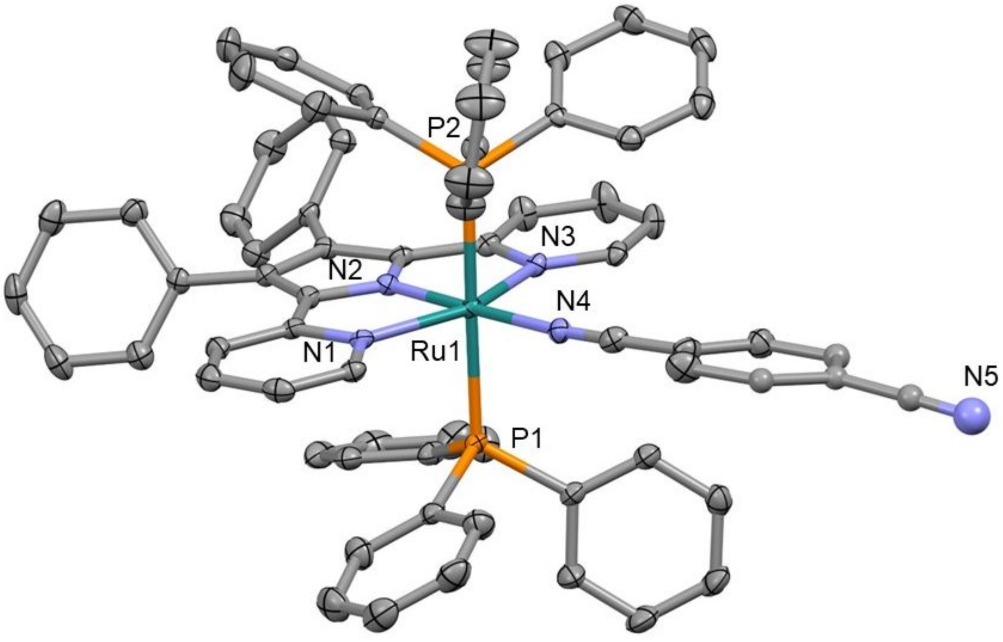

Figure S46. Crystal structure of **1a**; counter ion, solvent molecule, disorder removed for clarity, thermal ellipsoids displayed at 50% probability.

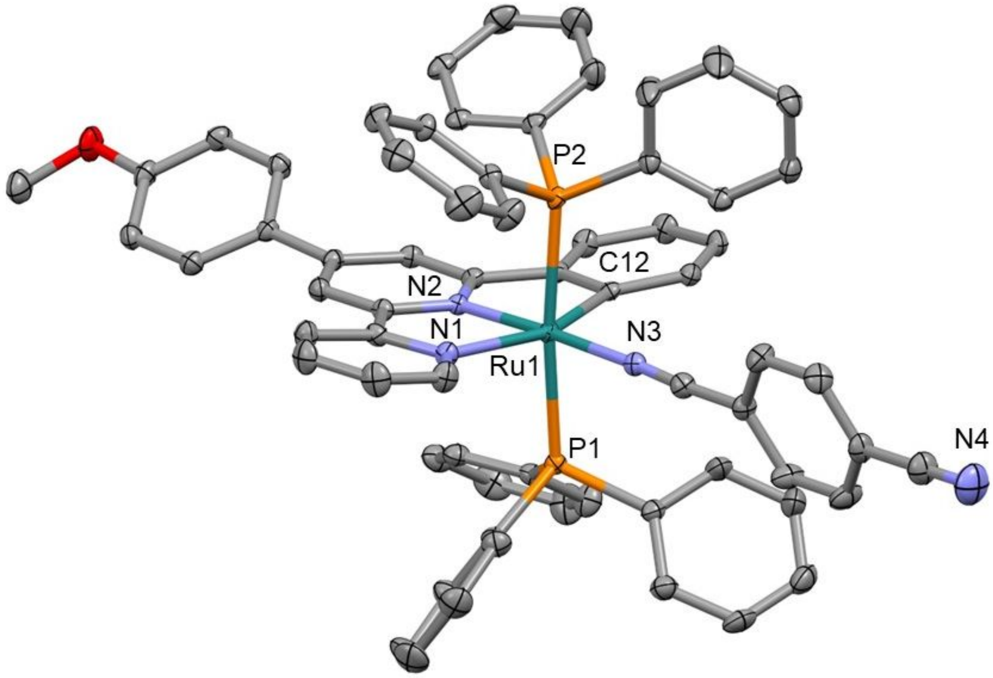

Figure S47. Crystal structure of **1c**; counter ion, solvent molecule, disorder removed for clarity, thermal ellipsoids displayed at 50% probability.

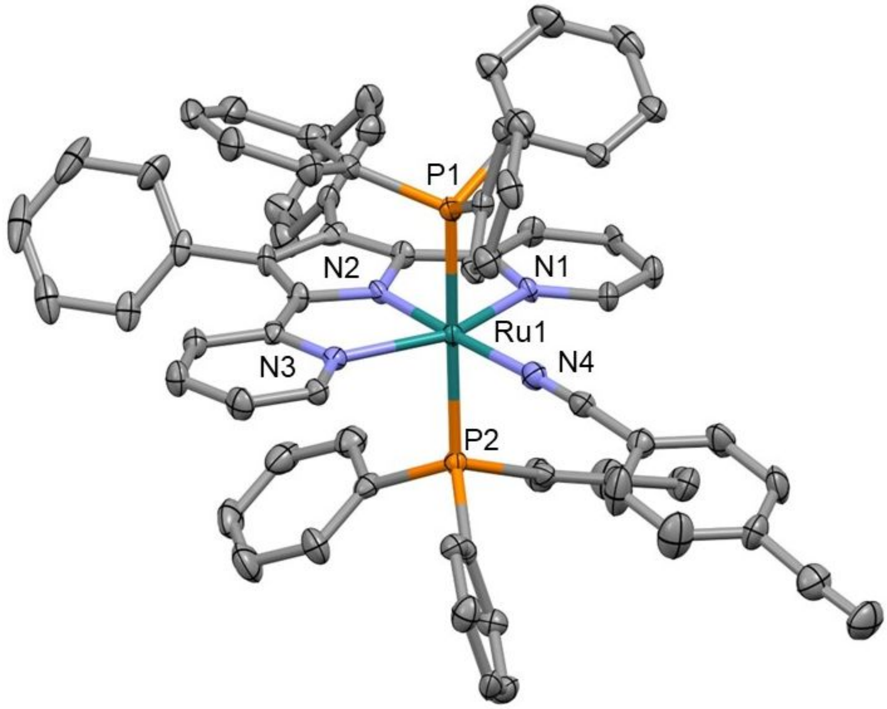

Figure S48. Crystal structure of **2a**; counter ion, solvent molecule, disorder removed for clarity, thermal ellipsoids displayed at 50% probability.

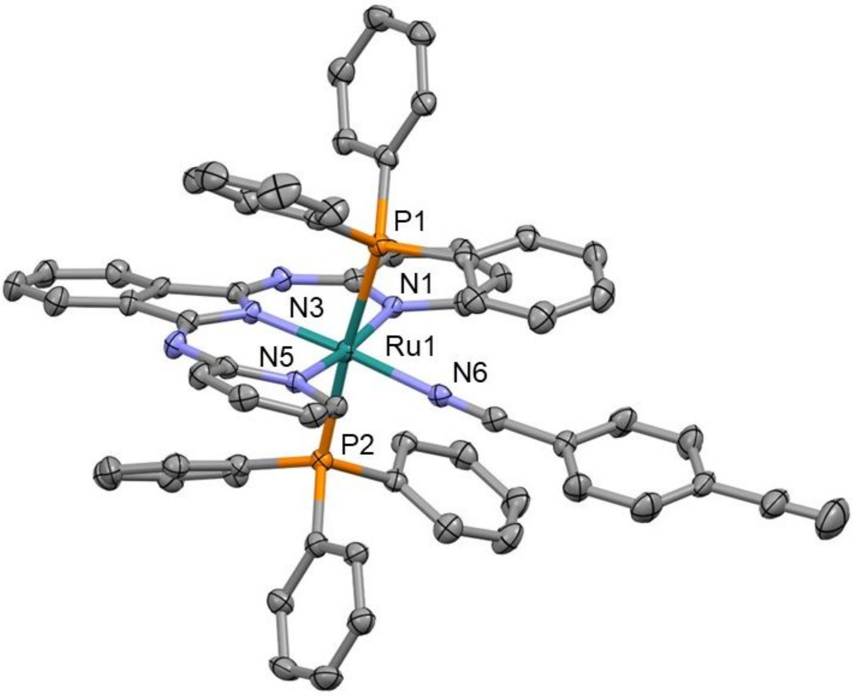

Figure S49. Crystal structure of **2b**; counter ion, solvent molecule, disorder removed for clarity, thermal ellipsoids displayed at 50% probability.

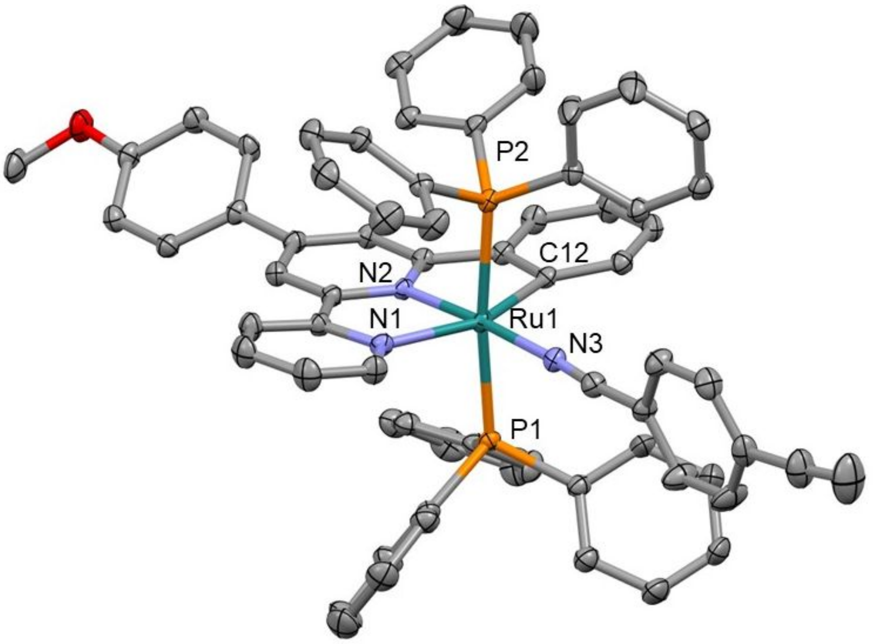

Figure S50. Crystal structure of **2c**; counter ion, solvent molecule, disorder removed for clarity, thermal ellipsoids displayed at 50% probability.

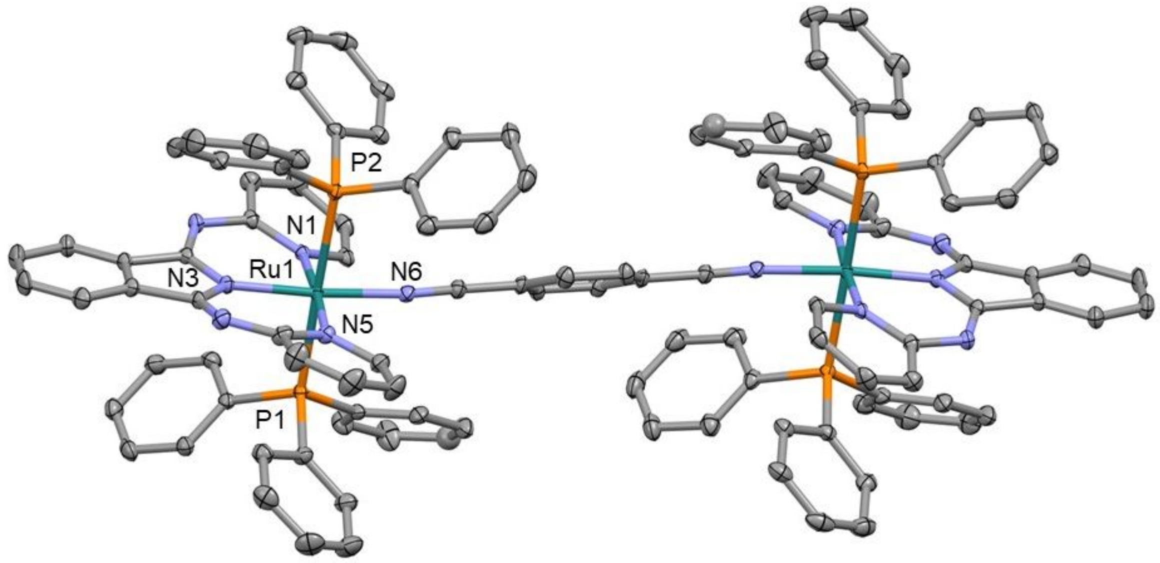

Figure S51. Crystal structure of **4b**; counter ion, solvent molecule, disorder removed for clarity, thermal ellipsoids displayed at 50% probability.

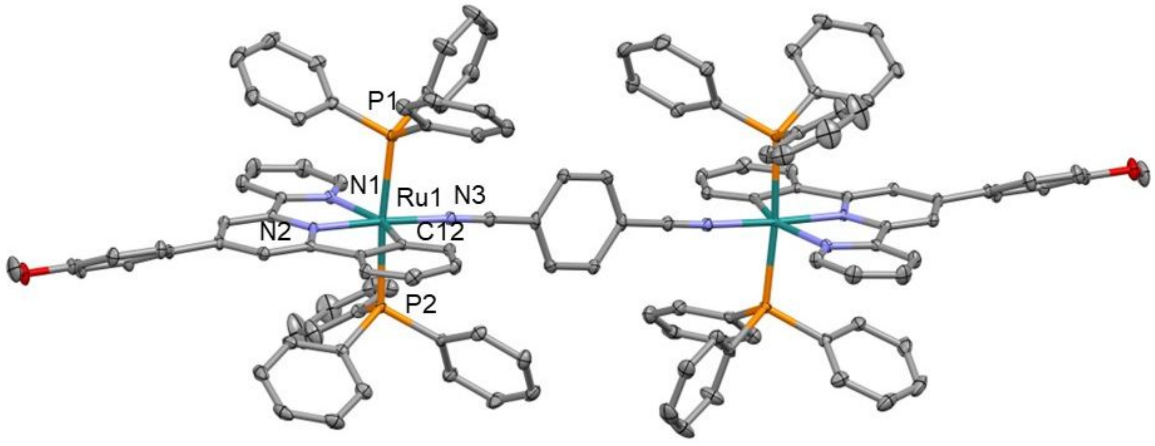

Figure S52. Crystal structure of **4c**; counter ion, solvent molecule, disorder removed for clarity, thermal ellipsoids displayed at 50% probability.

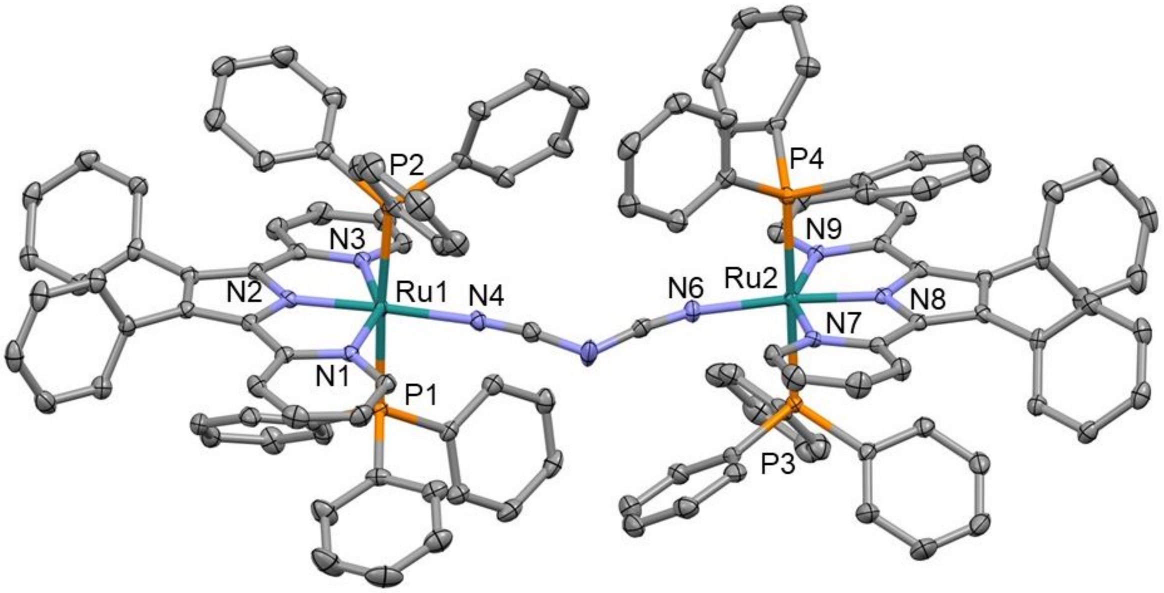

Figure S53. Crystal structure of **5a**; counter ion, solvent molecule, disorder removed for clarity, thermal ellipsoids displayed at 50% probability.

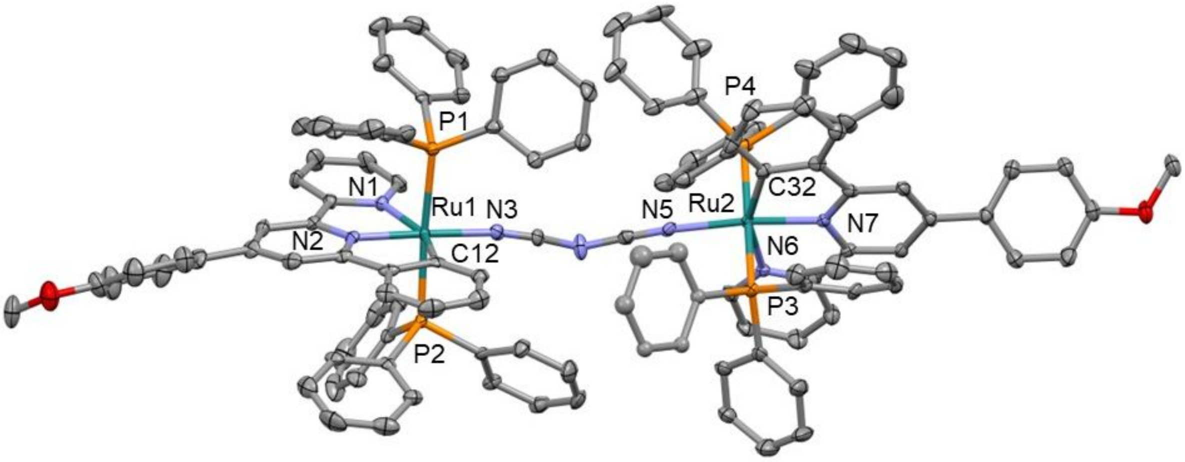

Figure S54. Crystal structure of **5c**; counter ion, solvent molecule, disorder removed for clarity, thermal ellipsoids displayed at 50% probability

S4. Physical Measurements

Electrochemistry

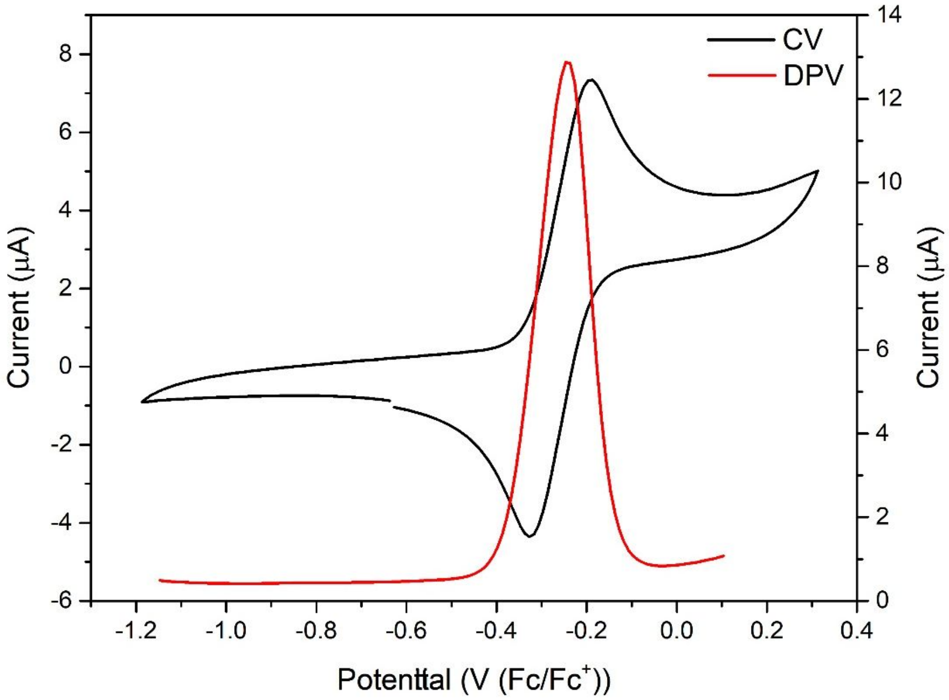

Figure S55. Cyclic voltammogram (CV) and differential pulse voltammogram (DPV) of **Cla** recorded in DCM/$TBAPF_6$.

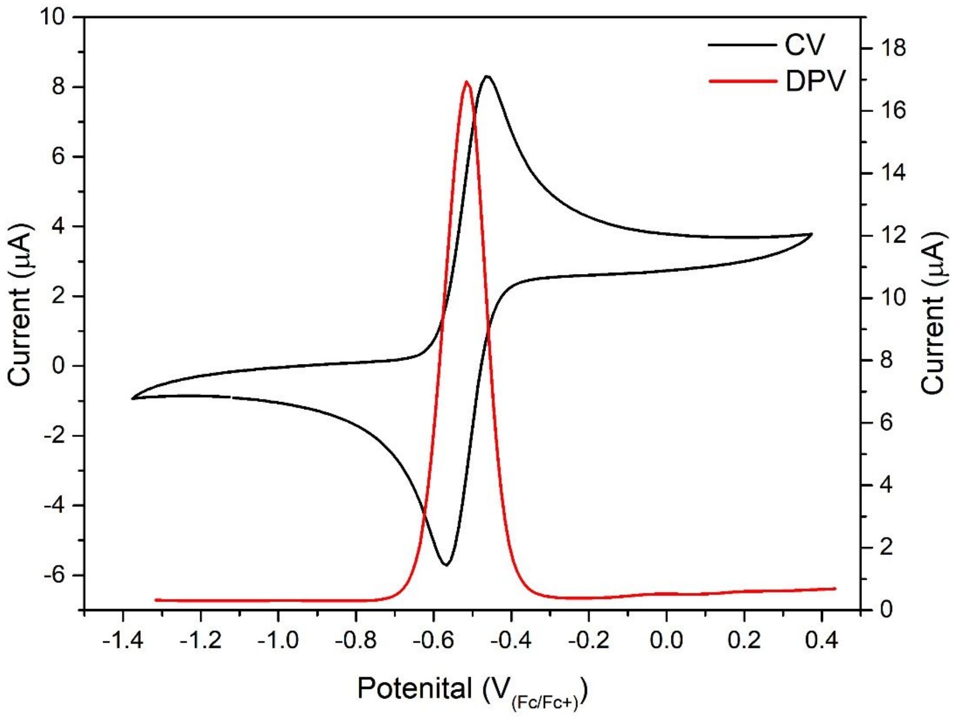

Figure S56. Cyclic voltammogram (CV) and differential pulse voltammogram (DPV) of **Clc** recorded in DCM/$TBAPF_6$.

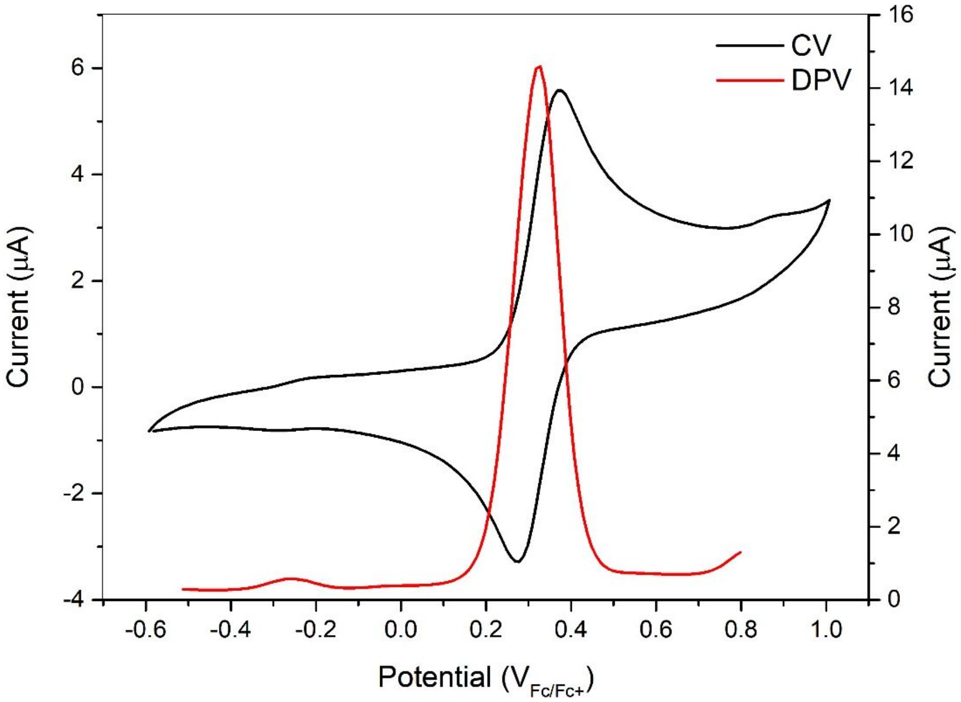

Figure S57. Cyclic voltammogram (CV) and differential pulse voltammogram (DPV) of **1a** recorded in DCM/$TBAPF_6$.

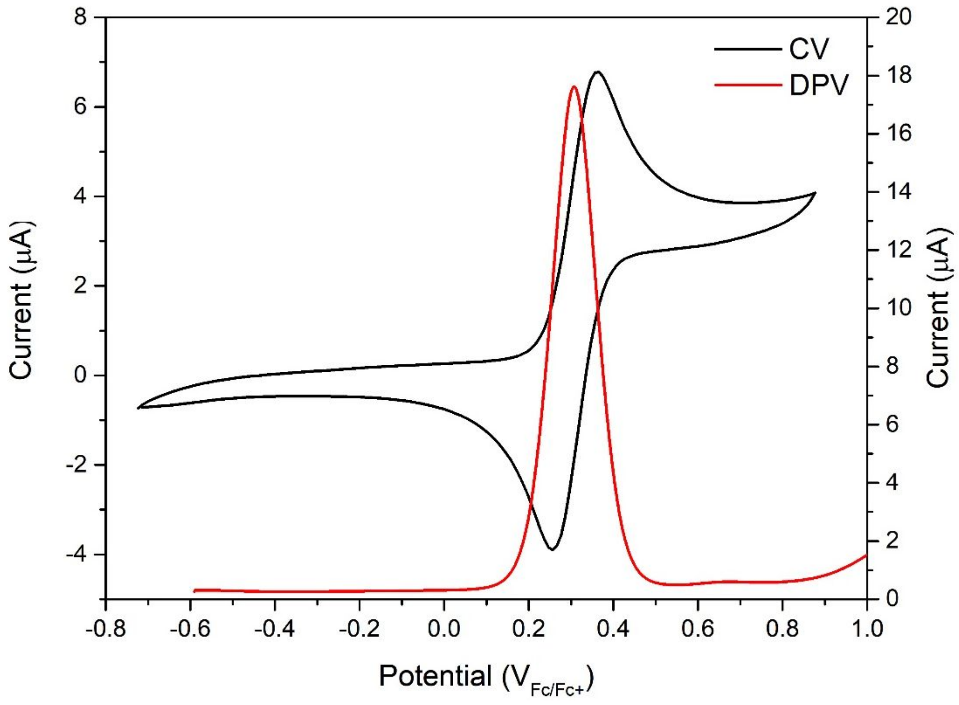

Figure S58. Cyclic voltammogram (CV) and differential pulse voltammogram (DPV) of **1c** recorded in DCM/$TBAPF_6$.

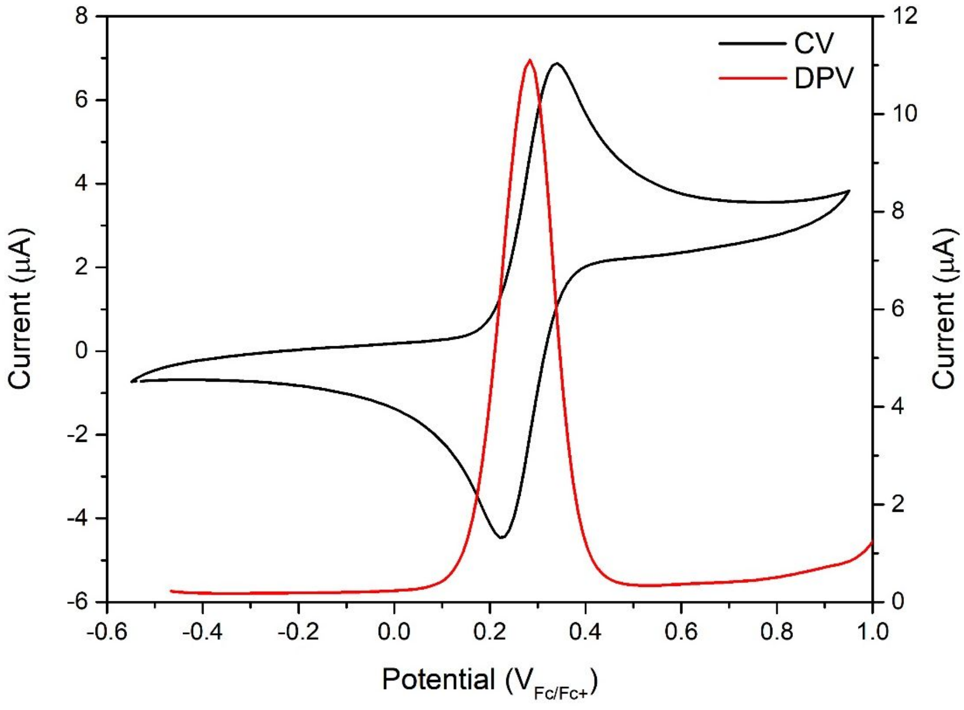

Figure S59. Cyclic voltammogram (CV) and differential pulse voltammogram (DPV) of **2a** recorded in DCM/$TBAPF_6$.

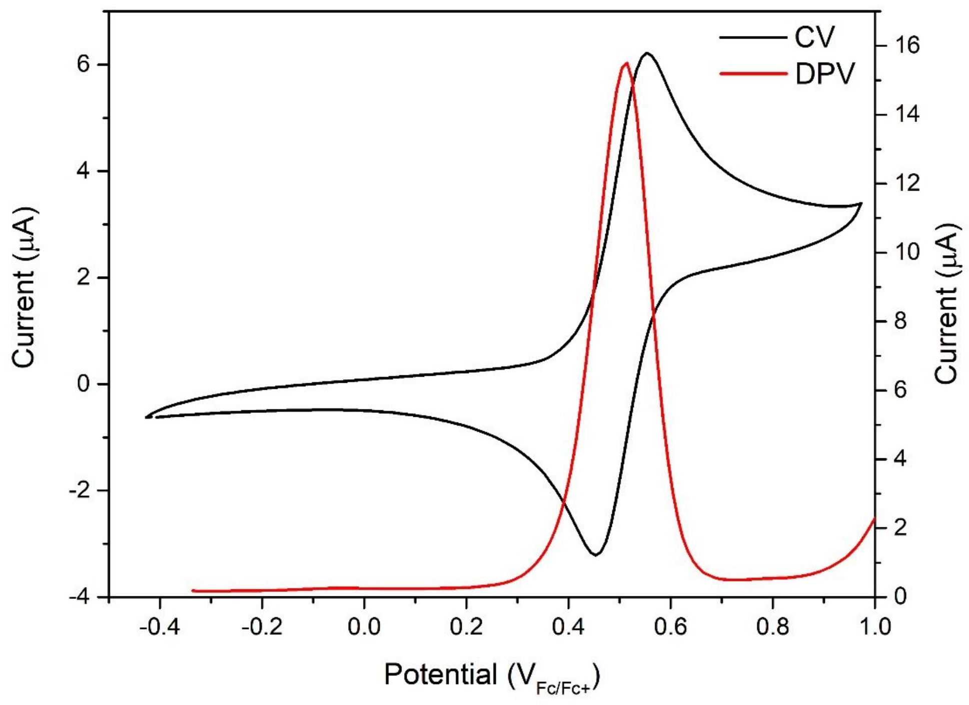

Figure S60. Cyclic voltammogram (CV) and differential pulse voltammogram (DPV) of **2b** recorded in DCM/$TBAPF_6$.

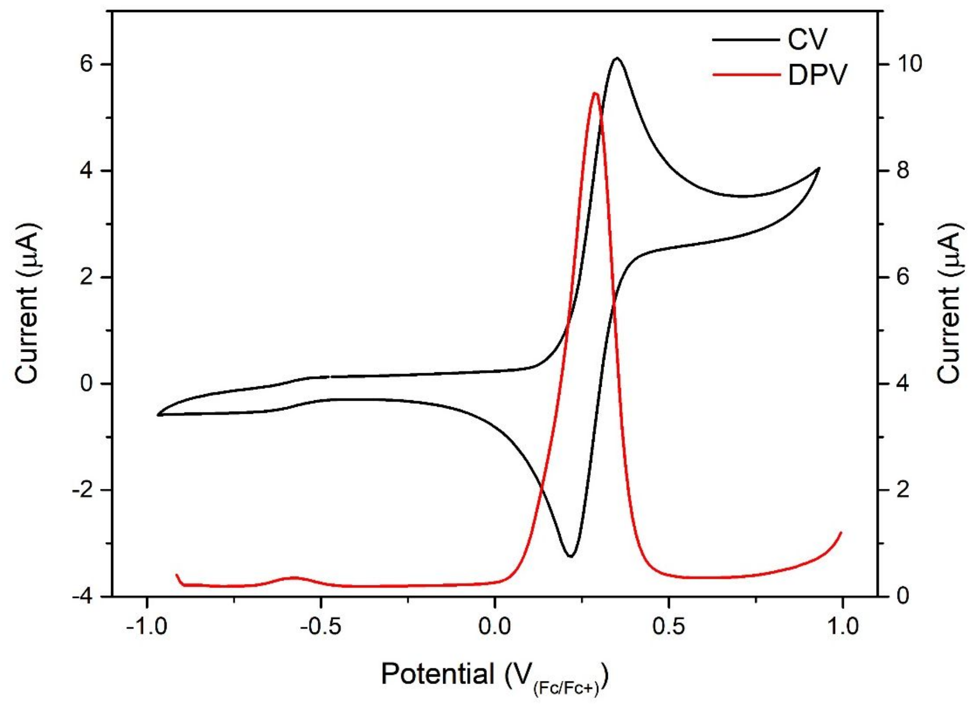

Figure S61. Cyclic voltammogram (CV) and differential pulse voltammogram (DPV) of **2c** recorded in DCM/$TBAPF_6$.

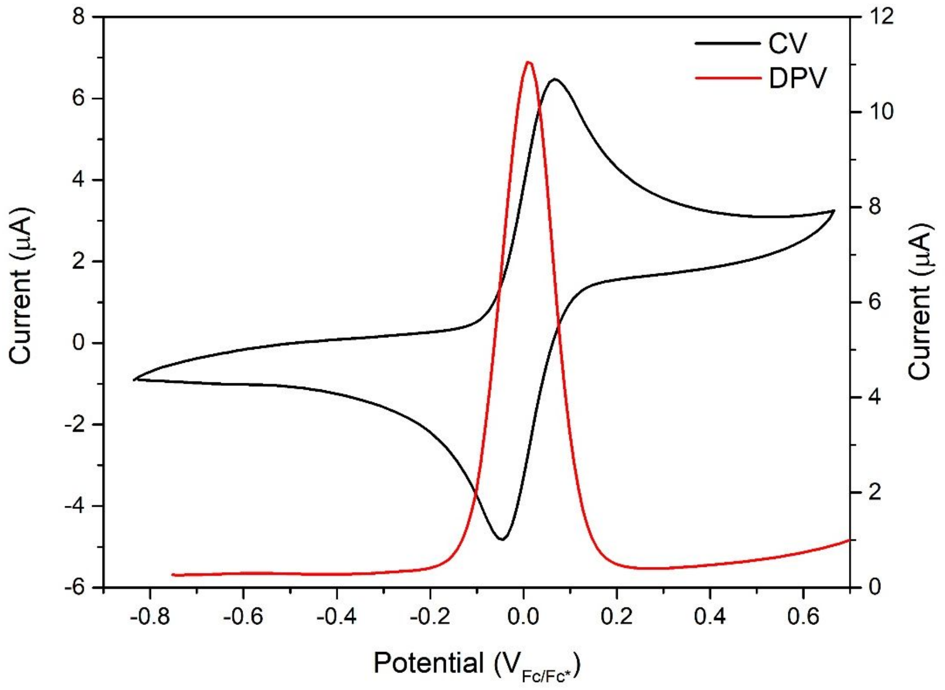

Figure S62. Cyclic voltammogram (CV) and differential pulse voltammogram (DPV) of **3a** recorded in DCM/$TBAPF_6$.

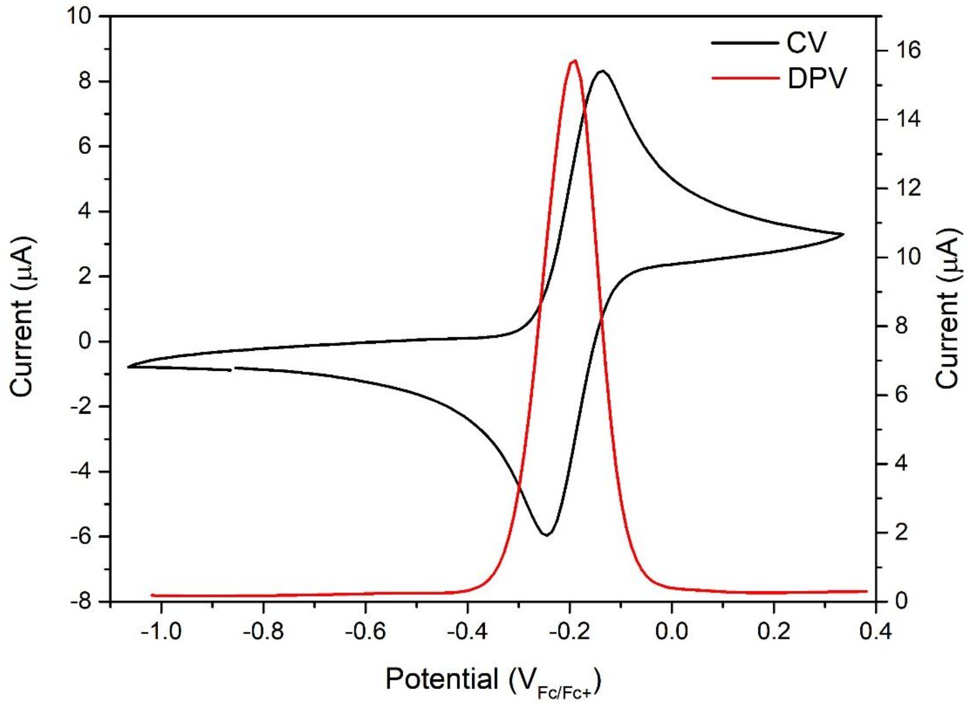

Figure S63. Cyclic voltammogram (CV) and differential pulse voltammogram (DPV) of **3c** recorded in DCM/$TBAPF_6$.

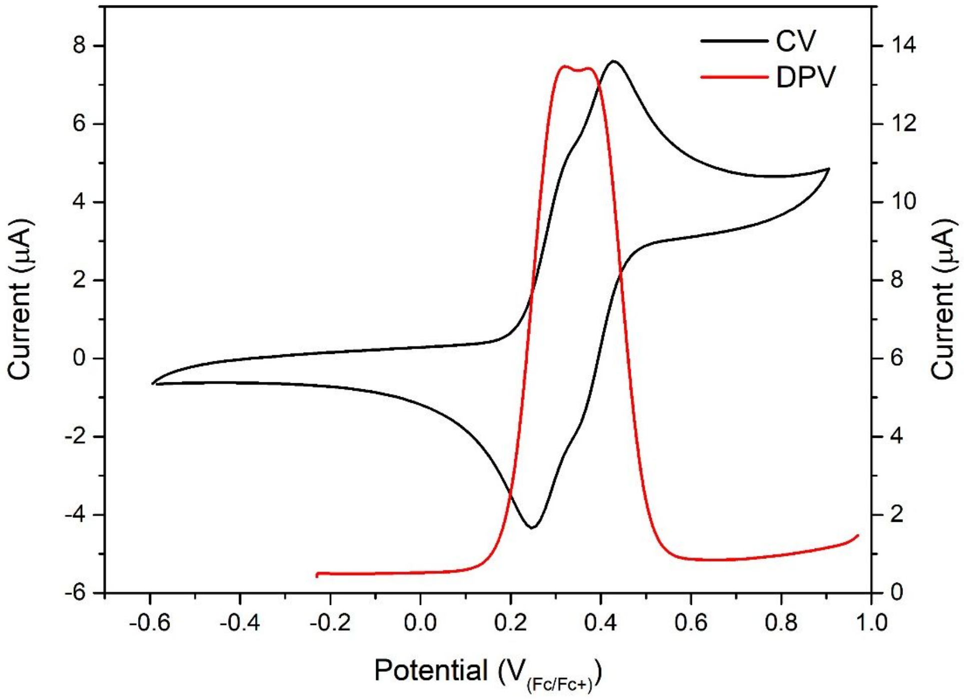

Figure S64. Cyclic voltammogram (CV) and differential pulse voltammogram (DPV) of **4a** recorded in DCM/$TBAPF_6$.

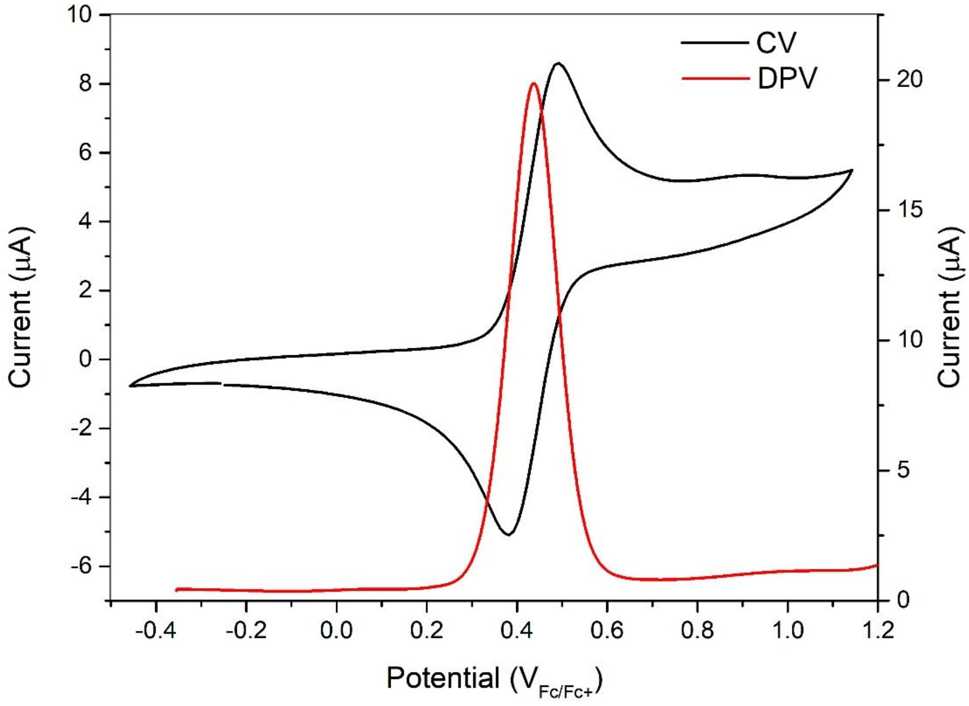

Figure S65. Cyclic voltammogram (CV) and differential pulse voltammogram (DPV) of **4a** recorded in DCM/ $TBArF_{24}$.

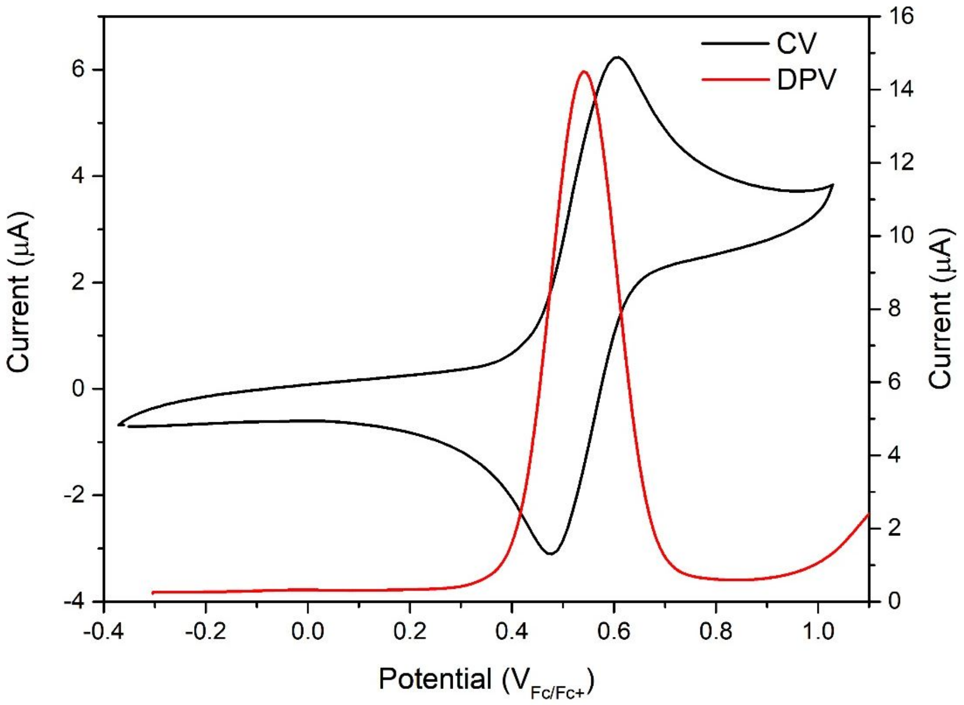

Figure S66. Cyclic voltammogram (CV) and differential pulse voltammogram (DPV) of **4b** recorded in DCM/$TBAPF_6$.

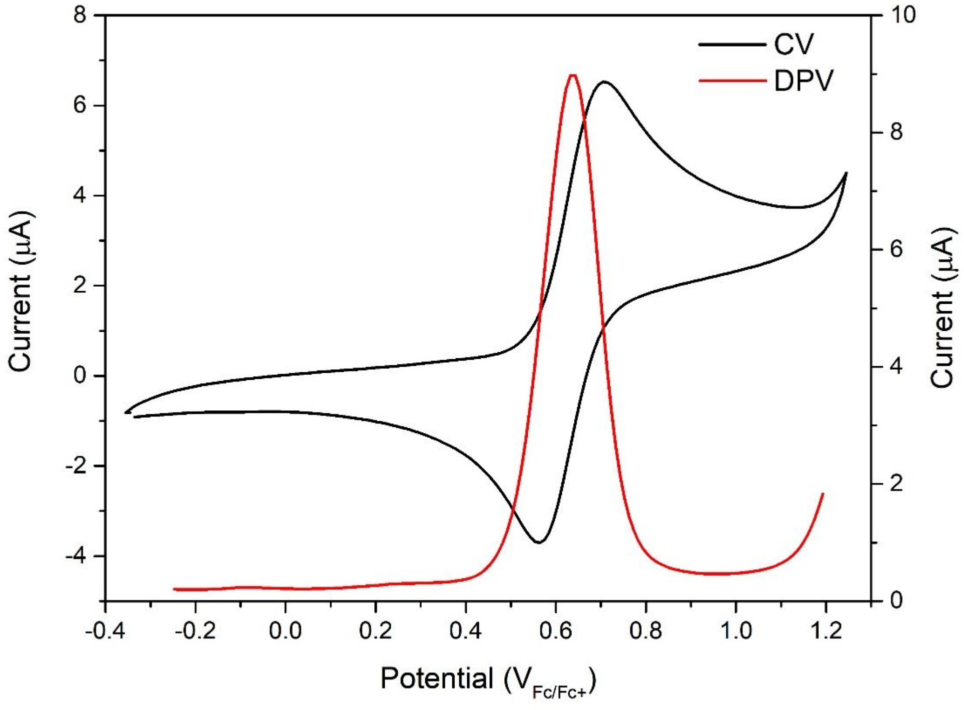

Figure S67. Cyclic voltammogram (CV) and differential pulse voltammogram (DPV) of **4b** recorded in DCM/ $TBArF_{24}$.

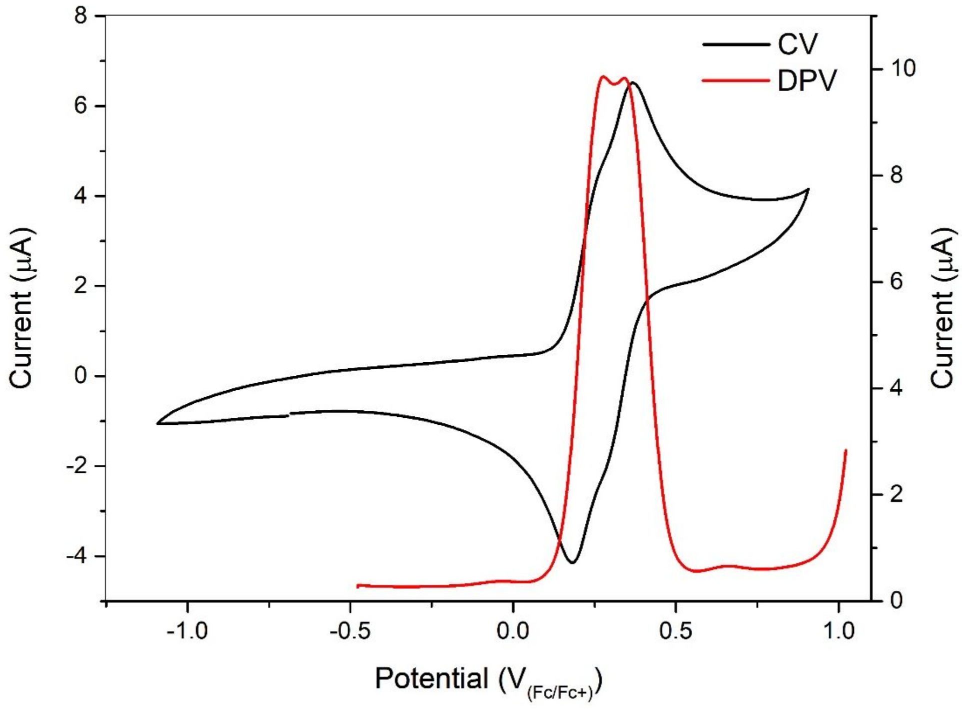

Figure S68. Cyclic voltammogram (CV) and differential pulse voltammogram (DPV) of **4c** recorded in DCM/TBAPF$_6$.

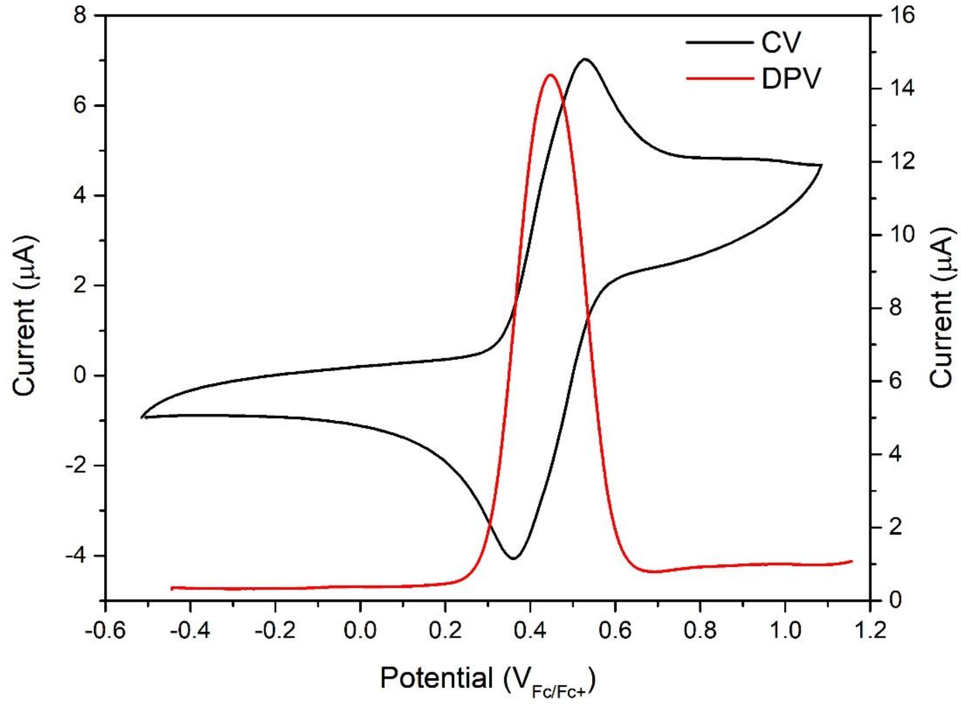

Figure S69. Cyclic voltammogram (CV) and differential pulse voltammogram (DPV) of **4c** recorded in DCM/ TBArF$_{24}$.

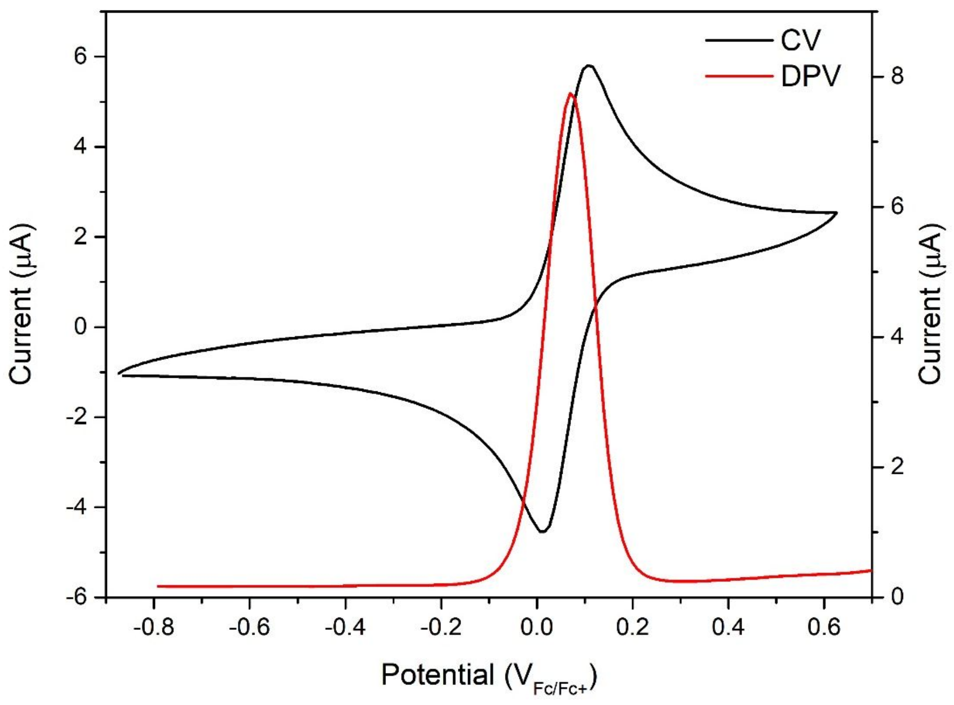

Figure S70. Cyclic voltammogram (CV) and differential pulse voltammogram (DPV) of **5a** recorded in DCM/$TBAPF_6$.

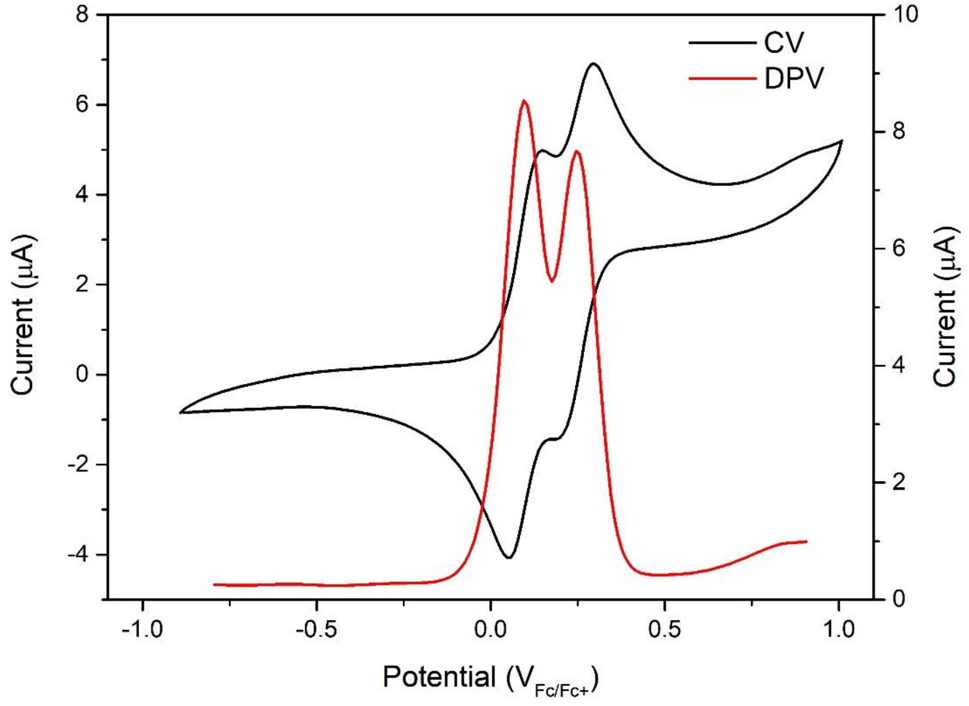

Figure S71. Cyclic voltammogram (CV) and differential pulse voltammogram (DPV) of **5a** recorded in DCM/ $TBArF_{24}$.

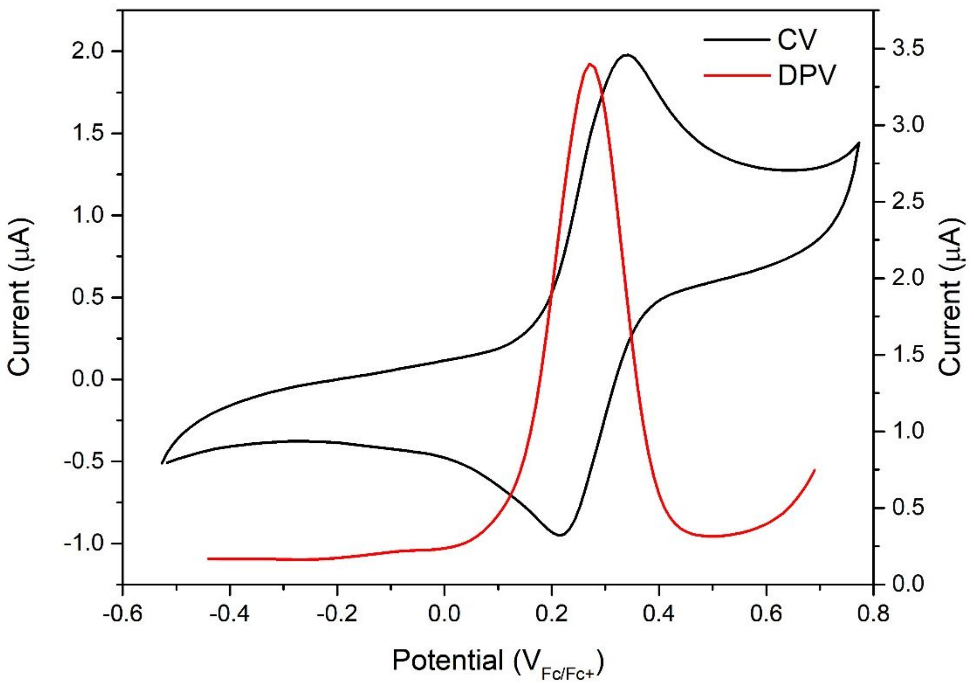

Figure S72. Cyclic voltammogram (CV) and differential pulse voltammogram (DPV) of **5b** recorded in DCM/$TBAPF_6$.

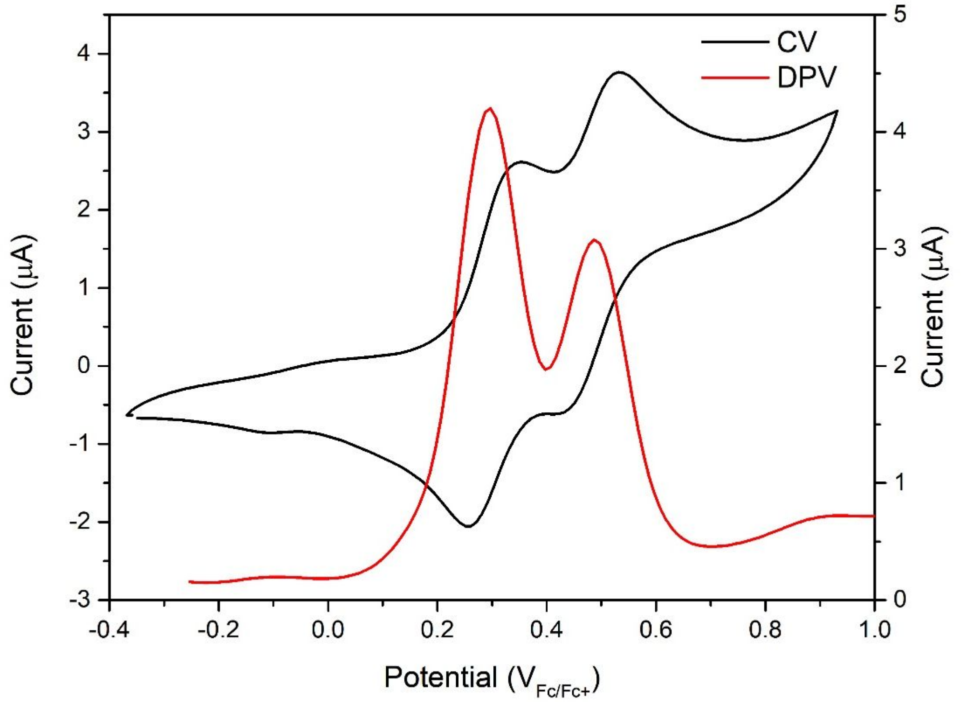

Figure S73. Cyclic voltammogram (CV) and differential pulse voltammogram (DPV) of **5b** recorded in DCM/ $TBArF_{24}$.

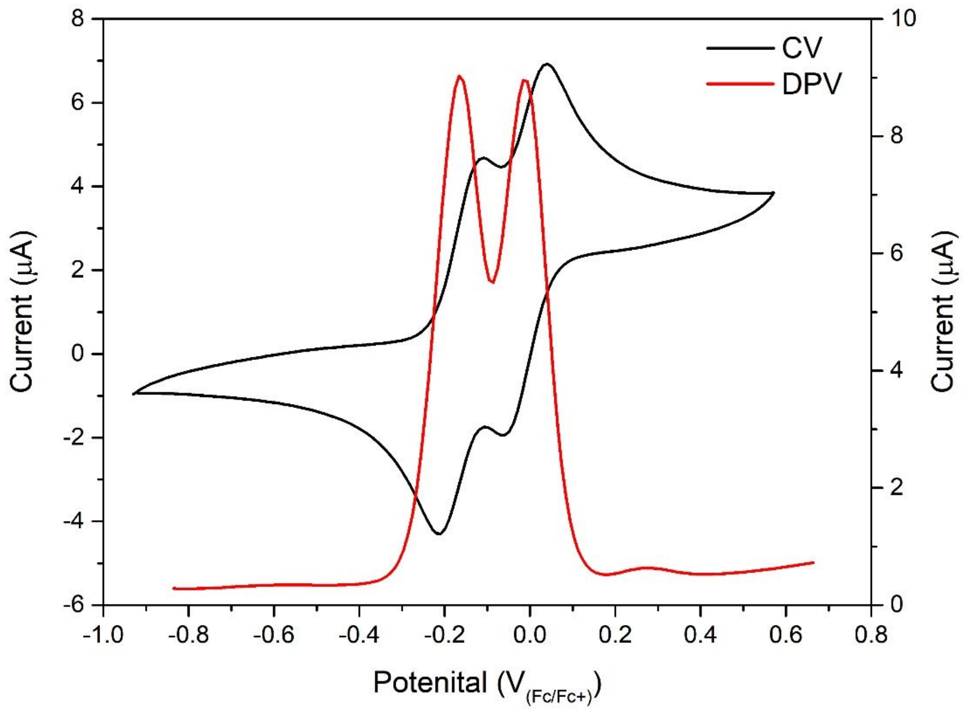

Figure S74. Cyclic voltammogram (CV) and differential pulse voltammogram (DPV) of **5c** recorded in DCM/$TBAPF_6$.

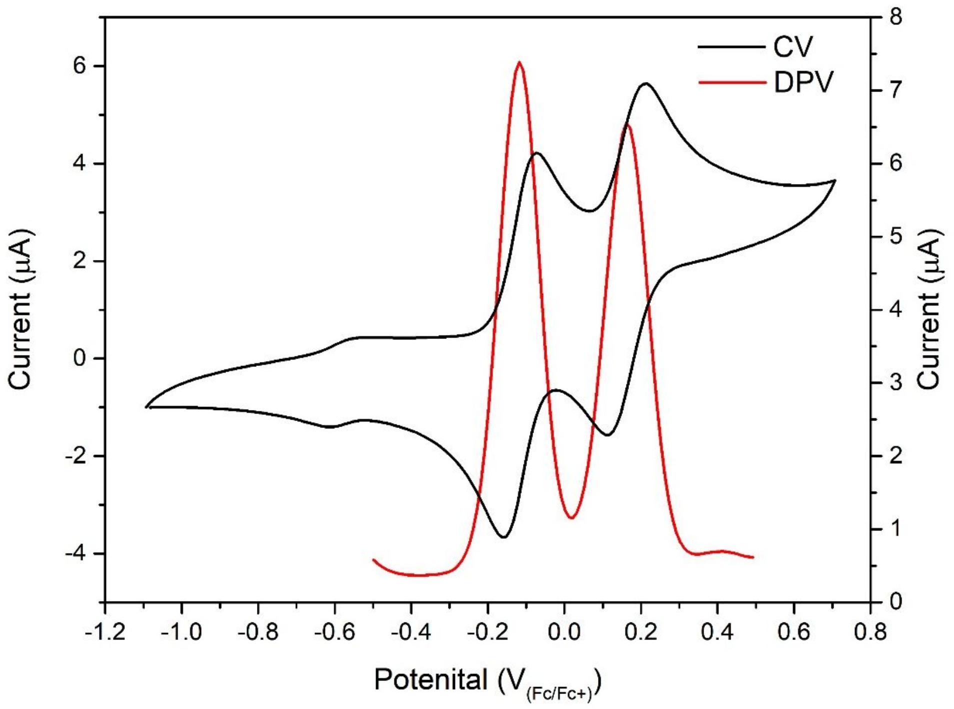

Figure S75. Cyclic voltammogram (CV) and differential pulse voltammogram (DPV) of **5c** recorded in DCM/ $TBArF_{24}$.

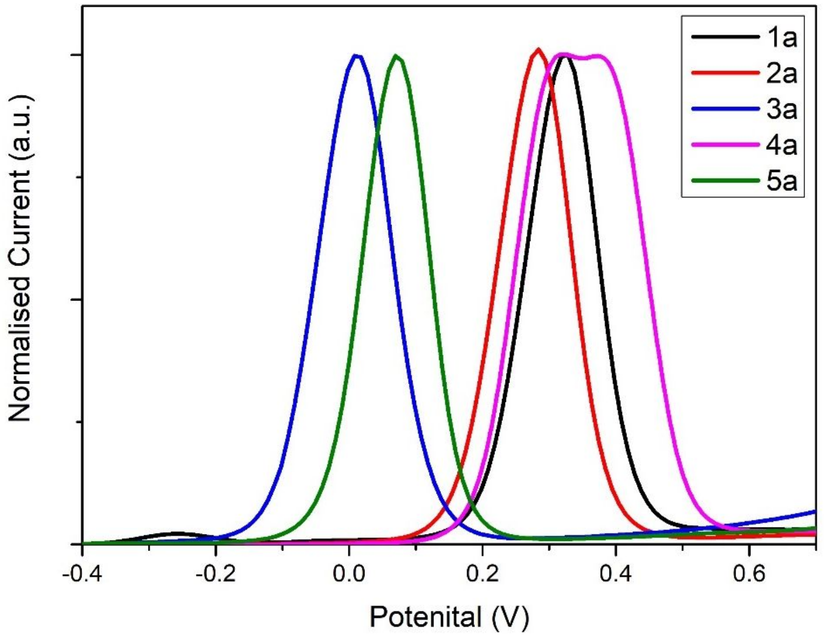

Figure S76. Differential pulse trace of complexes **1a**, **2a**, **3a**, **4a**, and **5a** recorded in $TBAPF_6$.

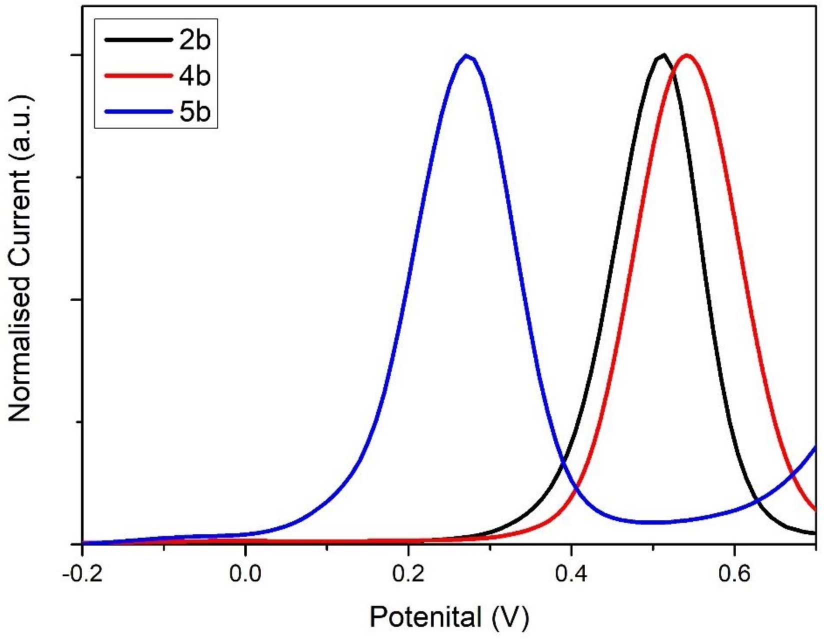

Figure S77. Differential pulse trace of complexes **2b**, **4b**, **5b** recorded in $TBAPF_6$.

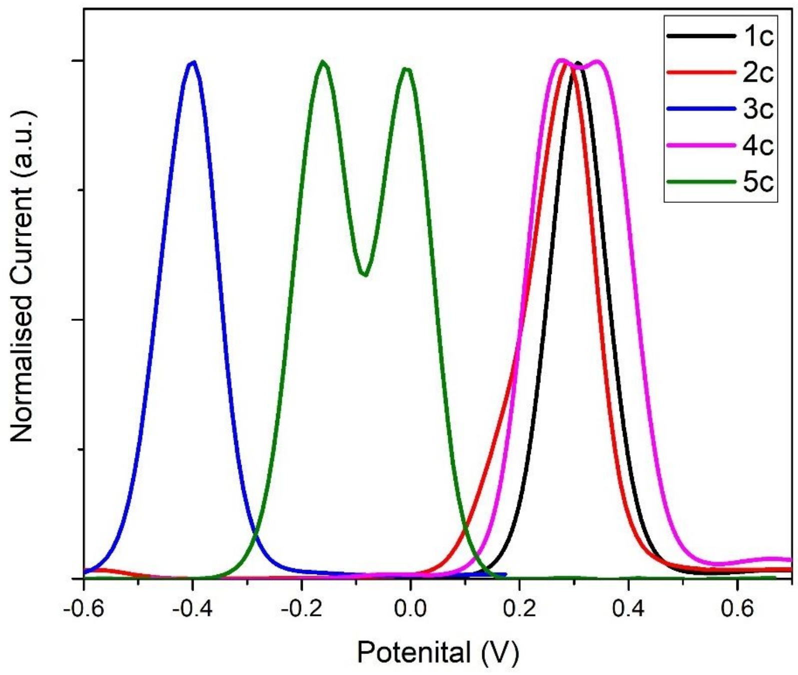

Figure S78. Differential pulse trace of complexes **1c**, **2c**, **3c**, **4c**, and **5c** recorded in $TBAPF_6$.

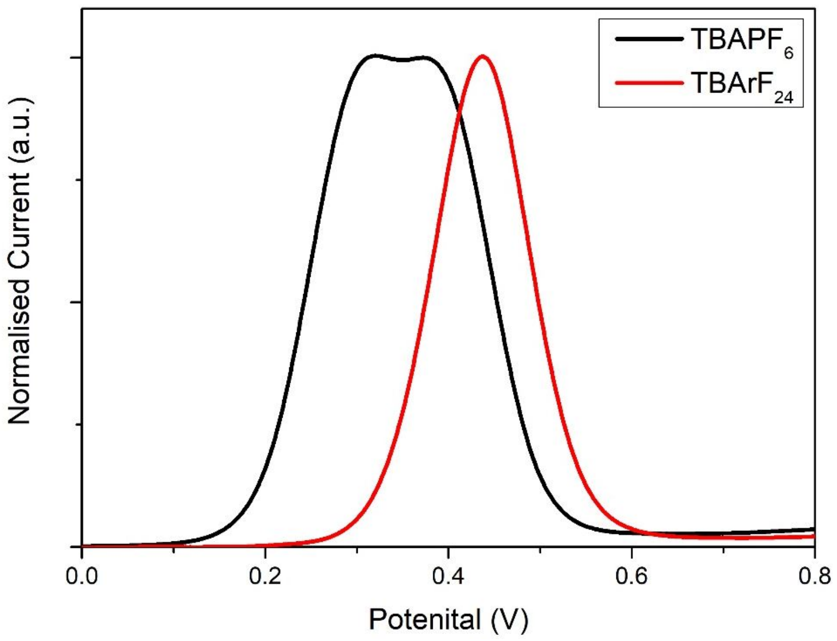

Figure S79. Differential pulse trace of complexes **4a** recorded in $TBAPF_6$ and $TBArF_{24}$.

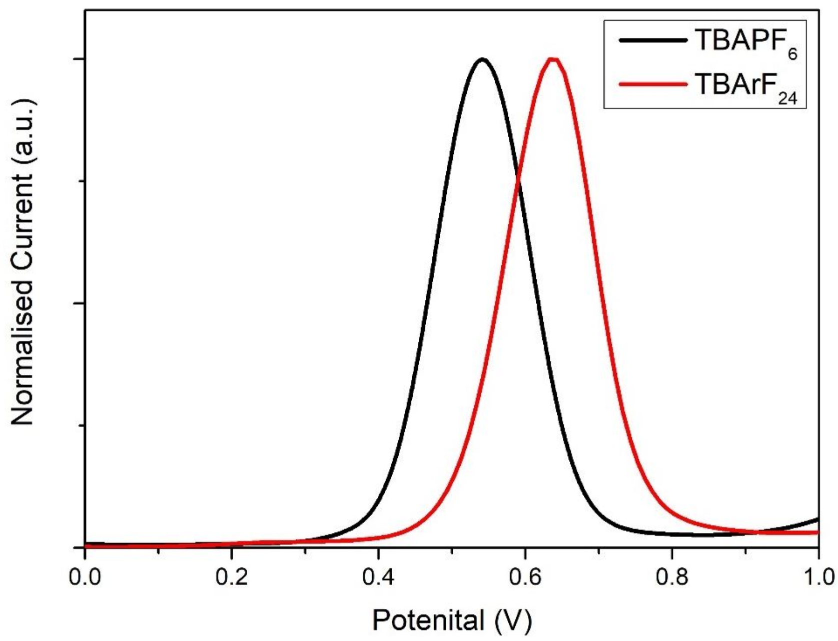

Figure S80. Differential pulse trace of complexes **4b** recorded in $TBAPF_6$ and $TBArF_{24}$.

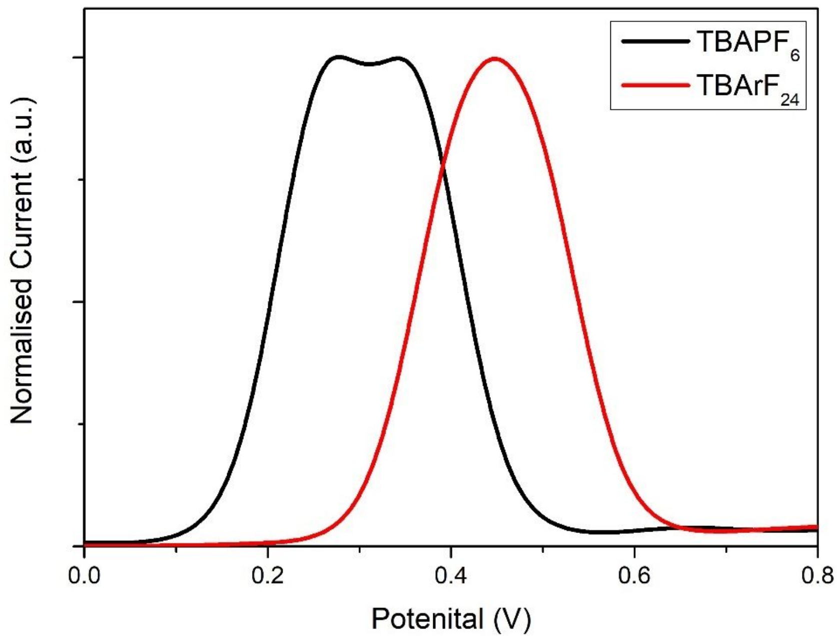

Figure S81. Differential pulse trace of complexes **4c** recorded in $TBAPF_6$ and $TBArF_{24}$.

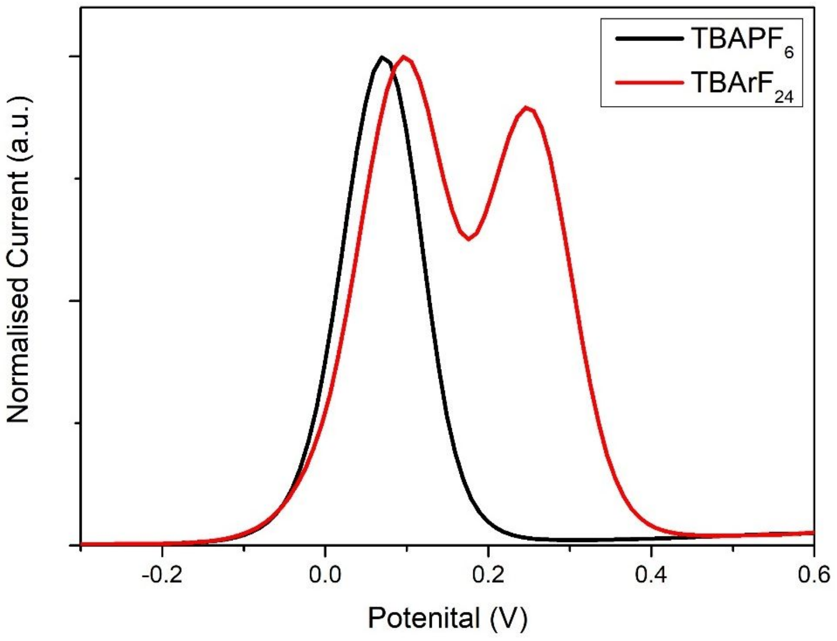

Figure S82. Differential pulse trace of complexes **5a** recorded in $TBAPF_6$ and $TBArF_{24}$.

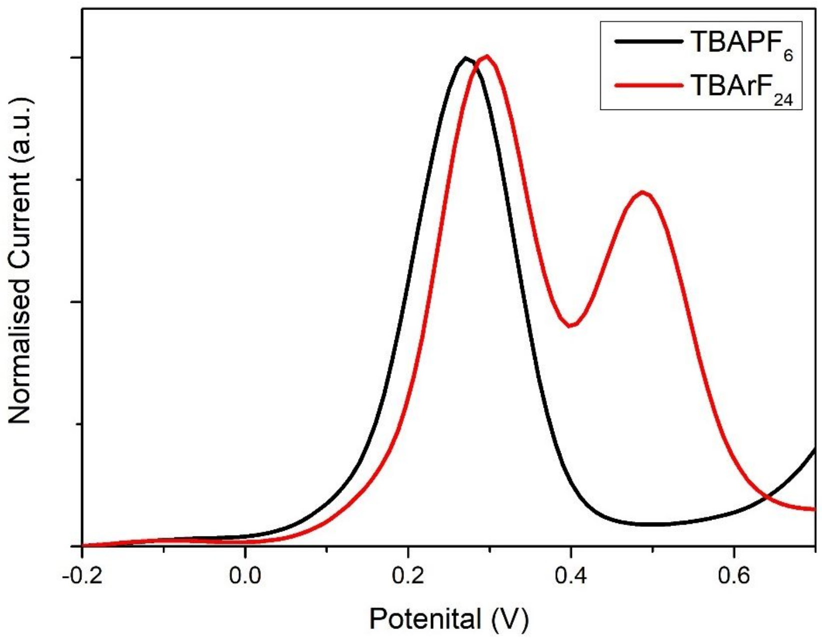

Figure S83. Differential pulse trace of complexes **5b** recorded in $TBAPF_6$ and $TBArF_{24}$.

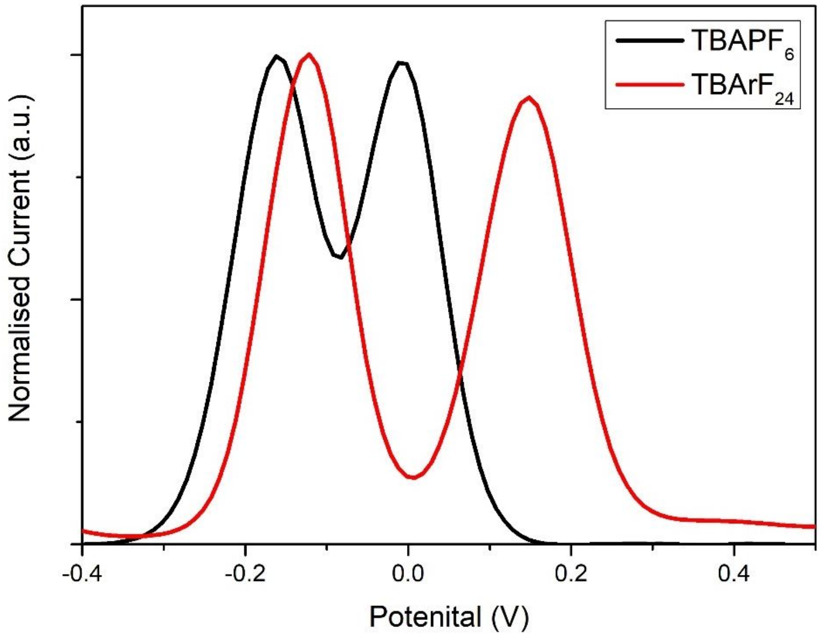

Figure S84. Differential pulse trace of complexes **5c** recorded in $TBAPF_6$ and $TBArF_{24}$.

Electronic Absorbance

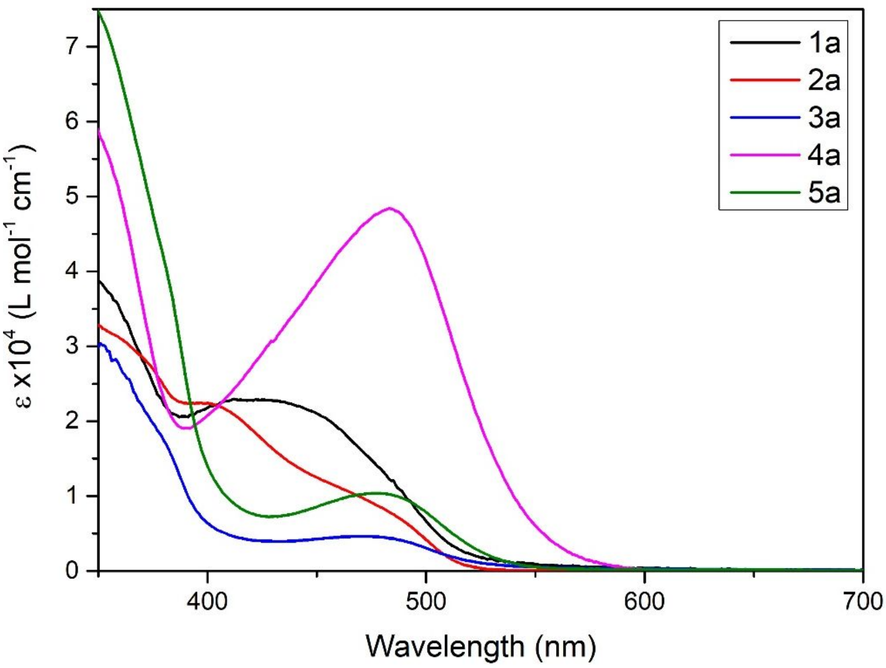

Figure S85. Electronic absorbance spectra of complexes **1a**, **2a**, **3a**, **4a**, and **5a**.

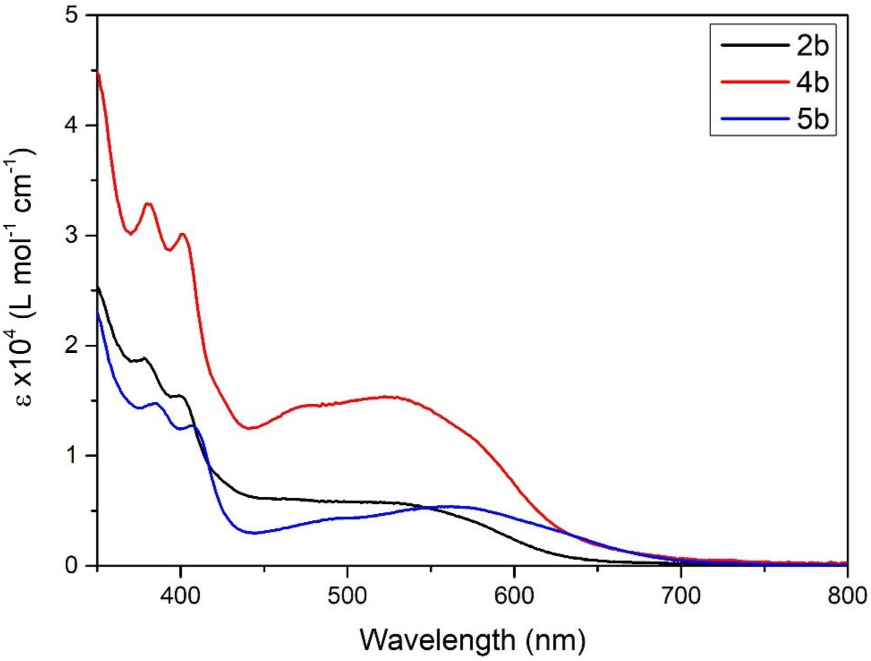

Figure S86. Electronic absorbance spectra of complexes **2b**, **4b**, and **5b**.

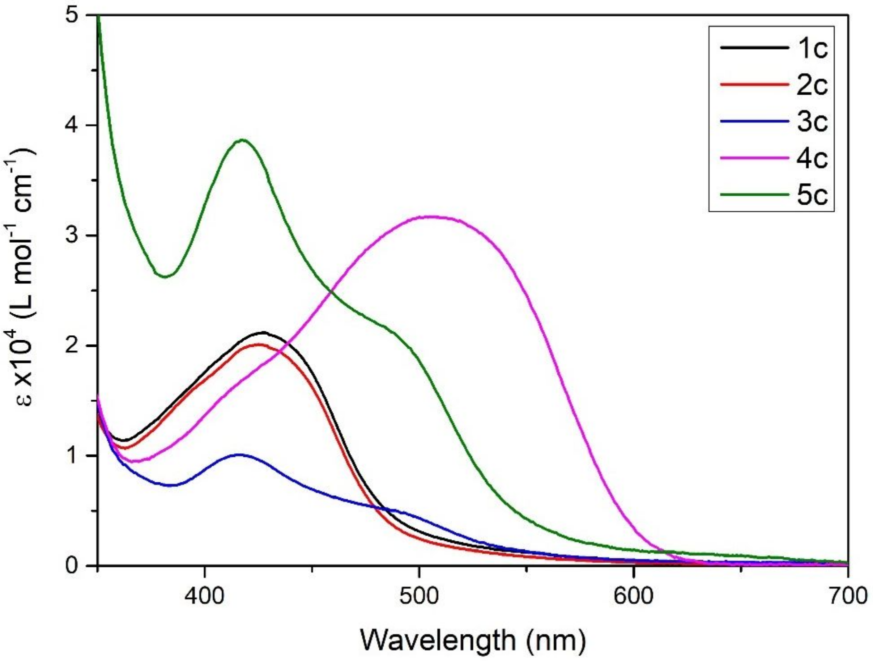

Figure S87. Electronic absorbance spectra of complexes **1c**, **2c**, **3c**, **4c**, and **5c**.

Photodissociation – Electronic Absorbance

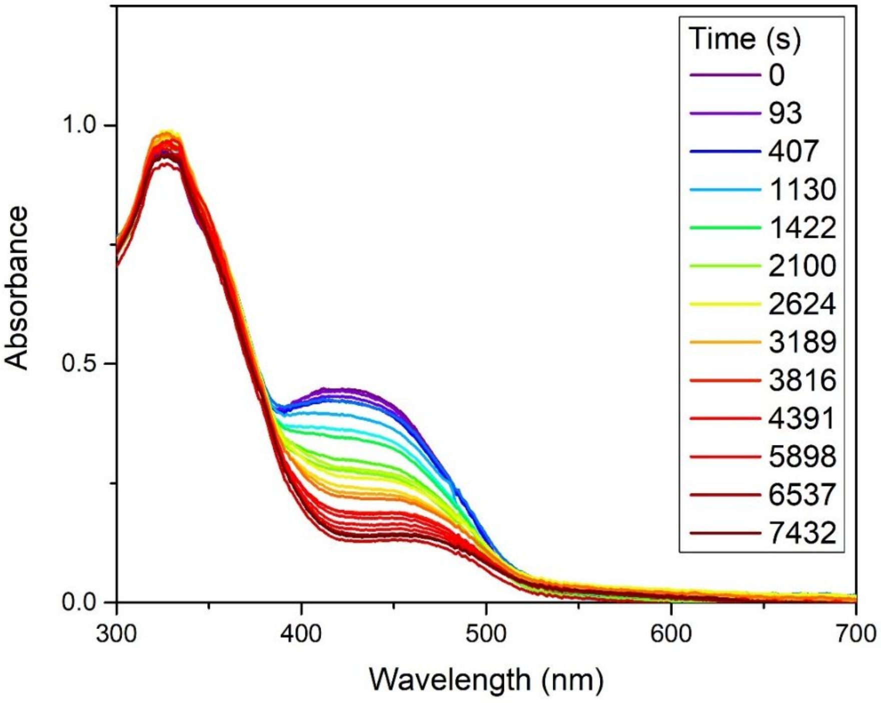

Figure S88. UV-Visible spectra of **1a** recorded during irradiation.

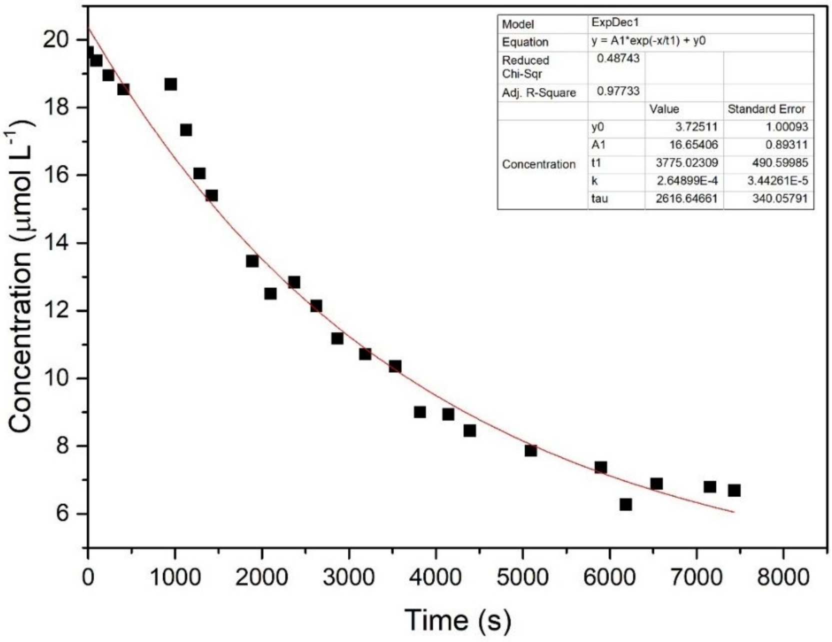

Figure S89. Plot of concentration of **1a** during the course of irradiation.

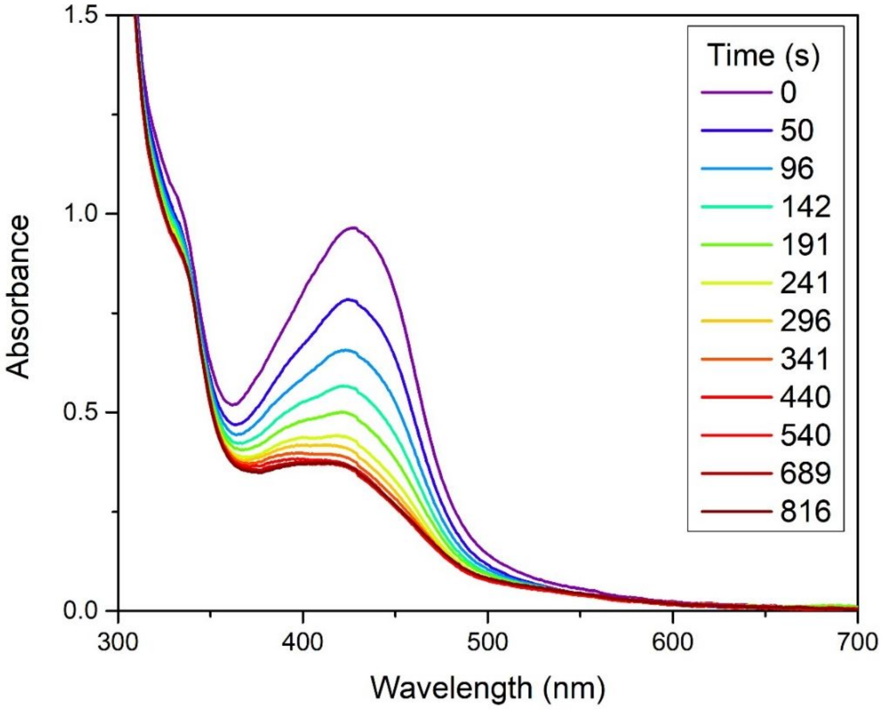

Figure S90. UV-Visible spectra of **1c** recorded during irradiation.

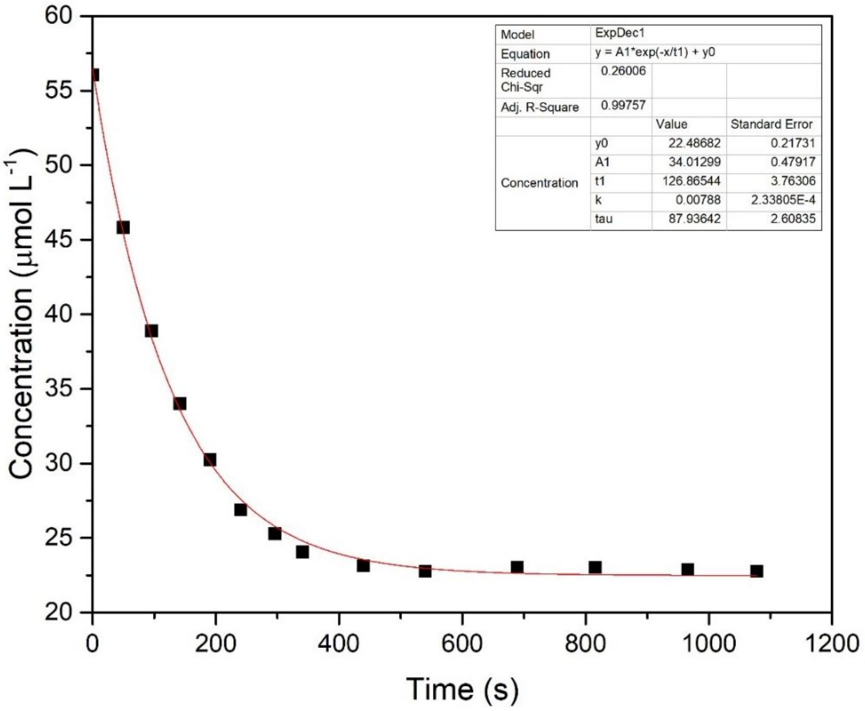

Figure S91. Plot of concentration of **1c** during the course of irradiation.

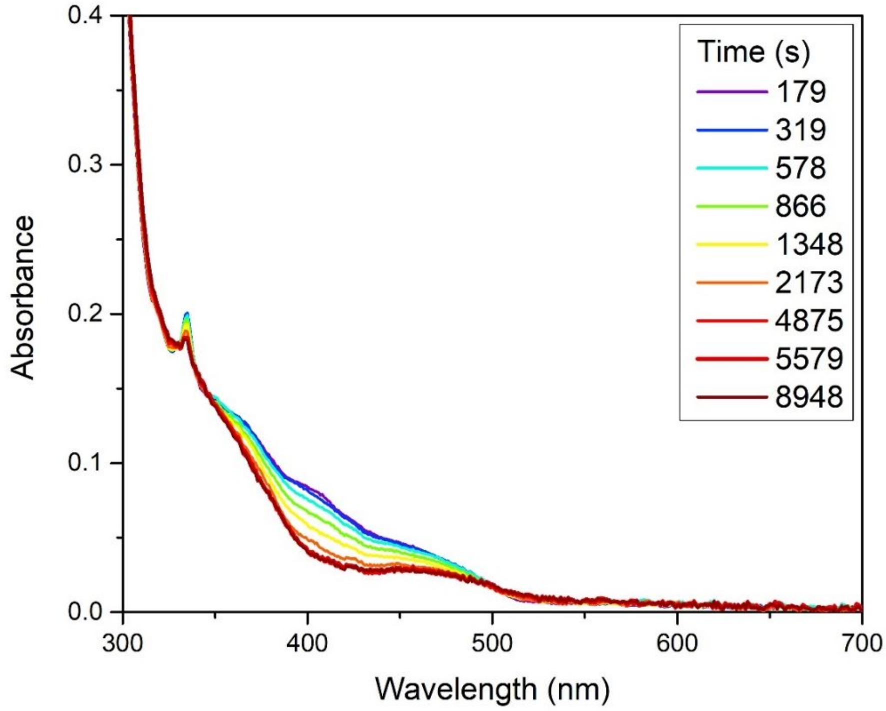

Figure S92. UV-Visible spectra of **2a** recorded during irradiation.

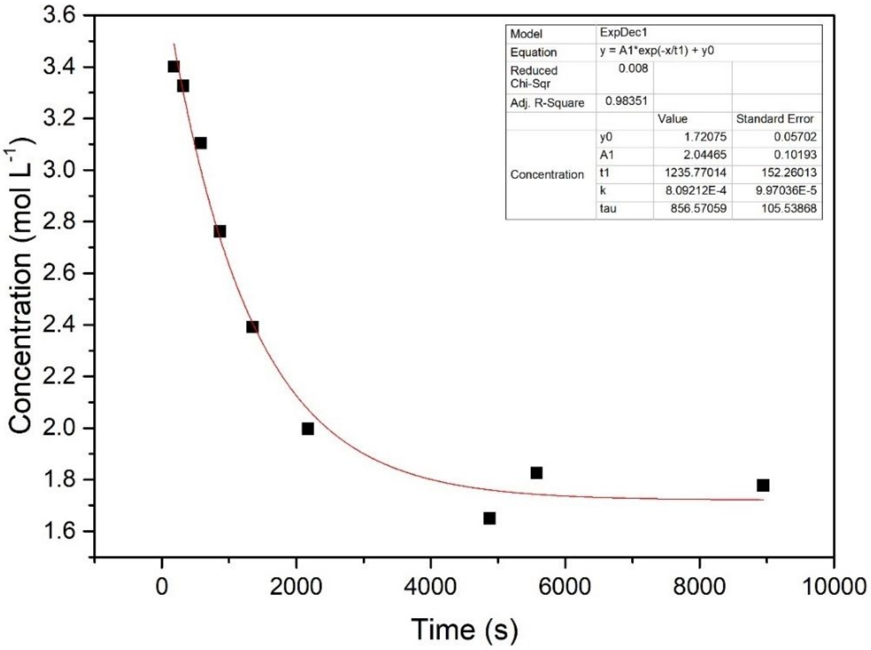

Figure S93. Plot of concentration of **2a** during the course of irradiation.

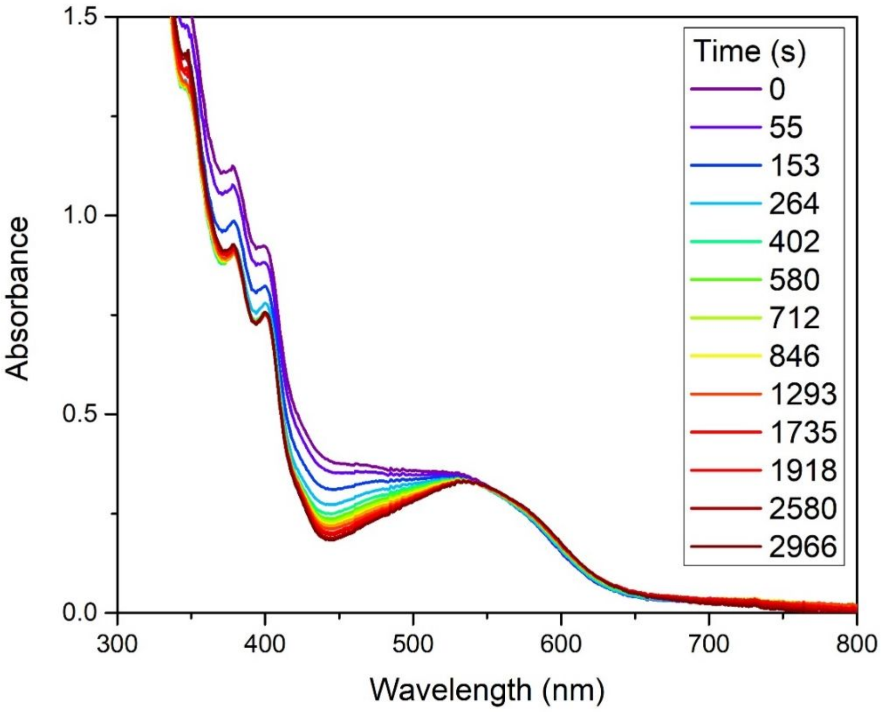

Figure S94. UV-Visible spectra of **2b** recorded during irradiation.

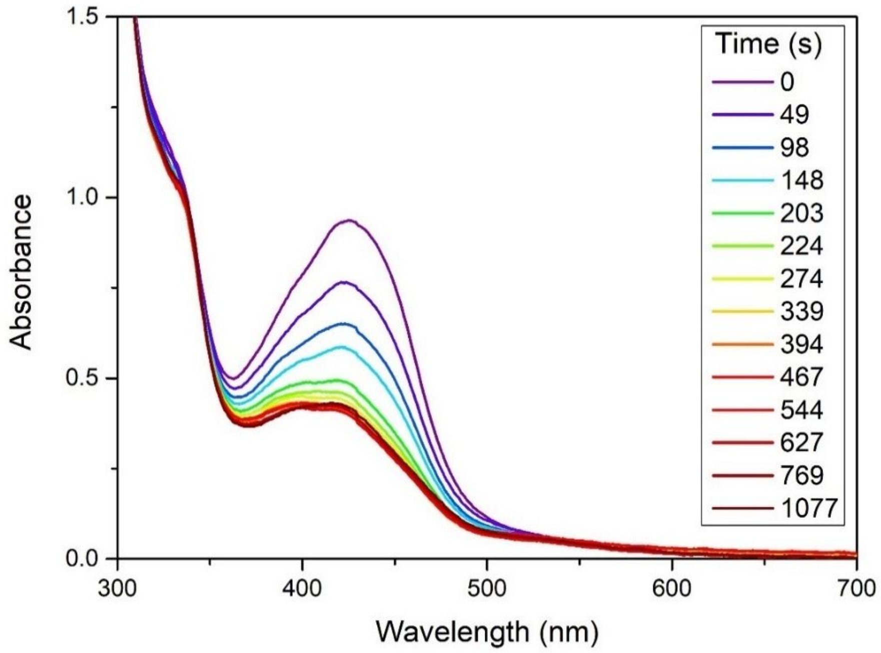

Figure S95. UV-Visible spectra of **2c** recorded during irradiation.

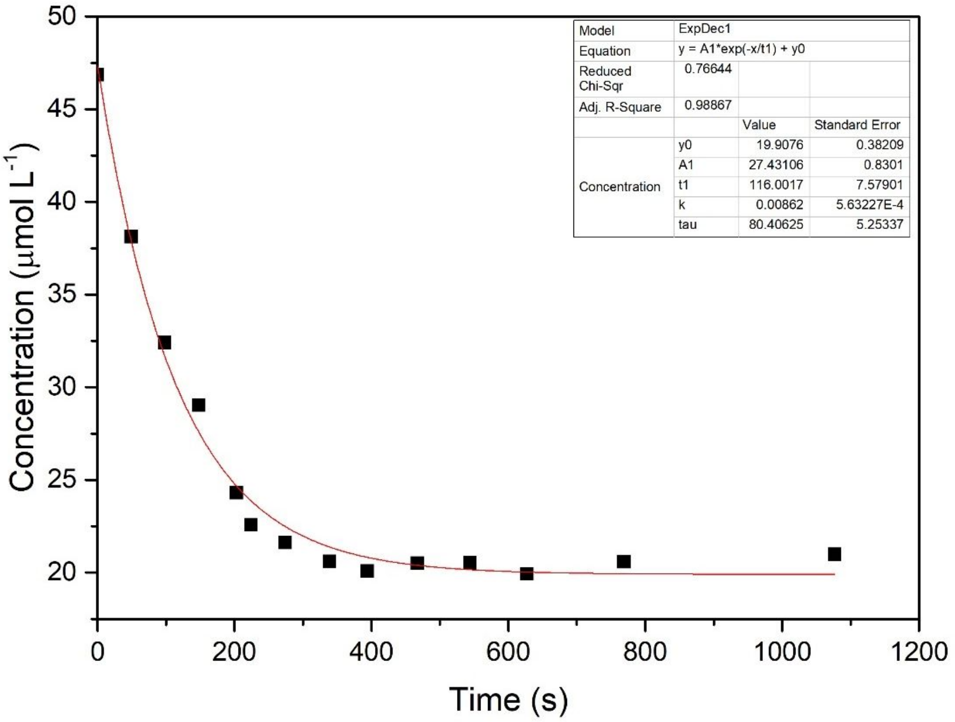

Figure S96. Plot of concentration of **2c** during the course of irradiation.

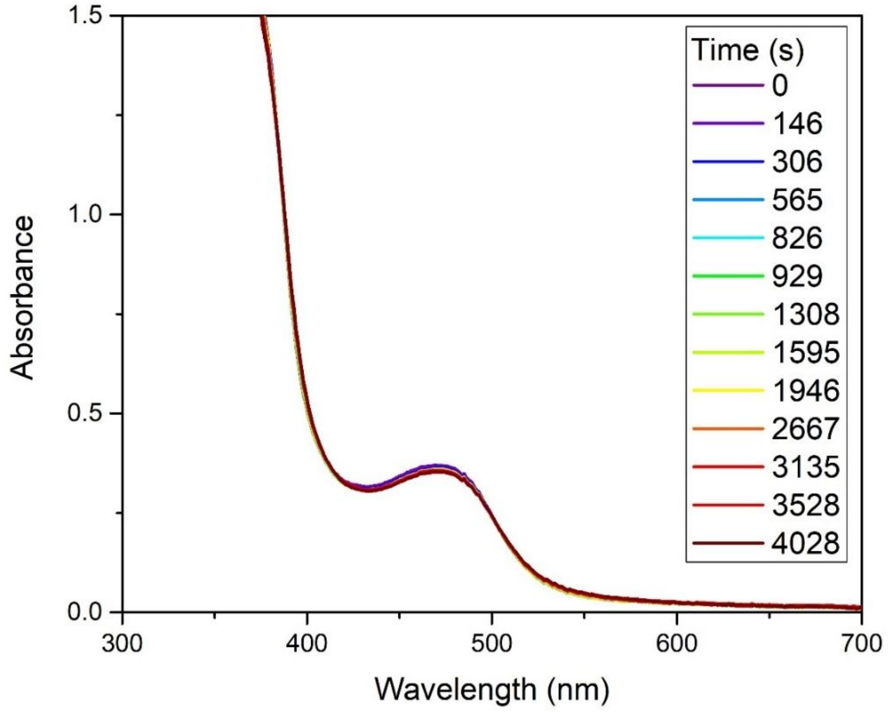

Figure S97. UV-Visible spectra of **3a** recorded during irradiation.

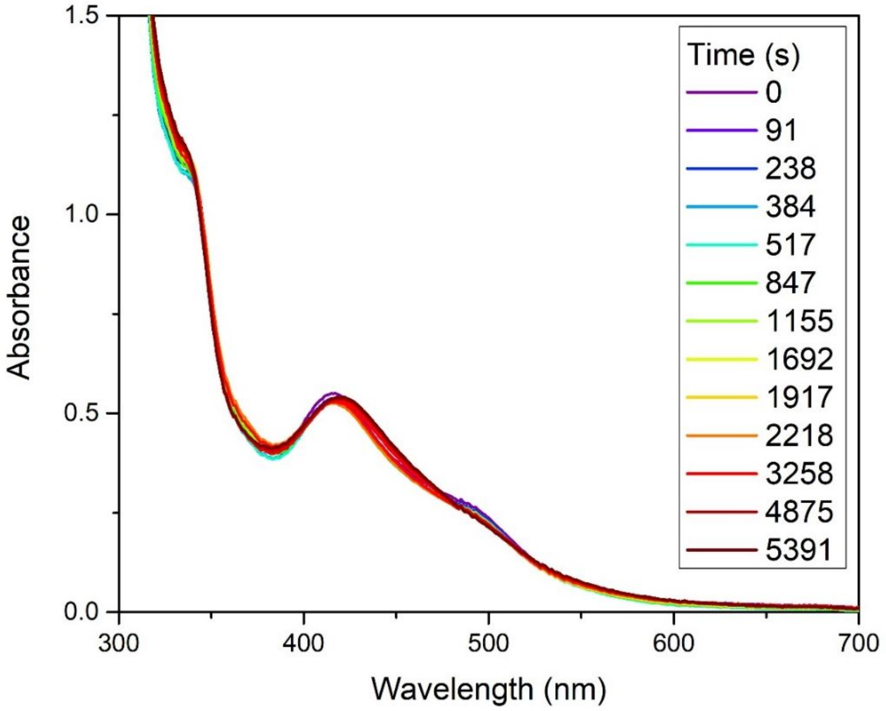

Figure S98. UV-Visible spectra of **3c** recorded during irradiation.

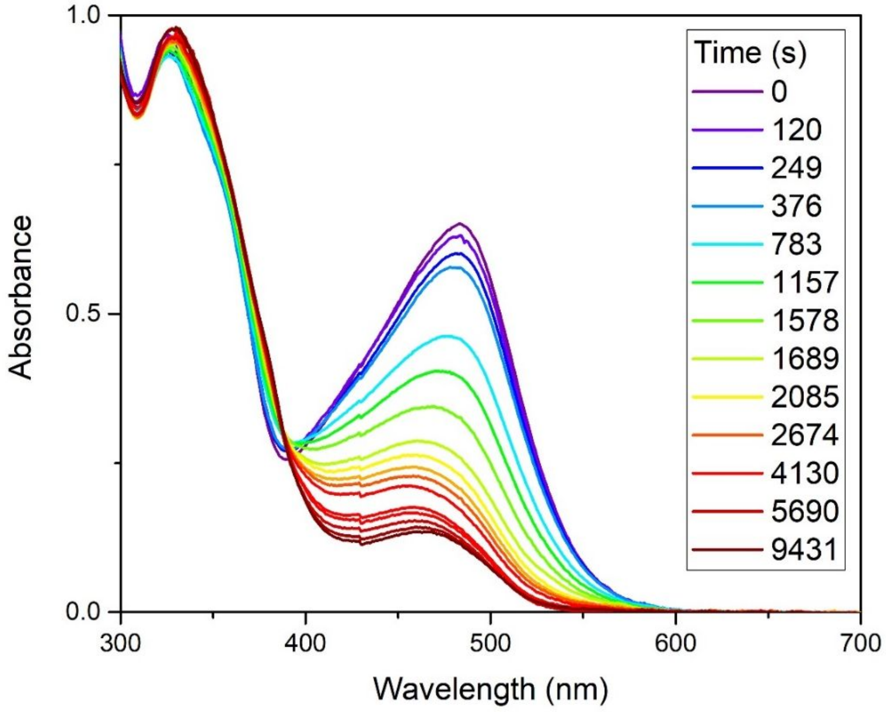

Figure S99. UV-Visible spectra of **4a** recorded during irradiation.

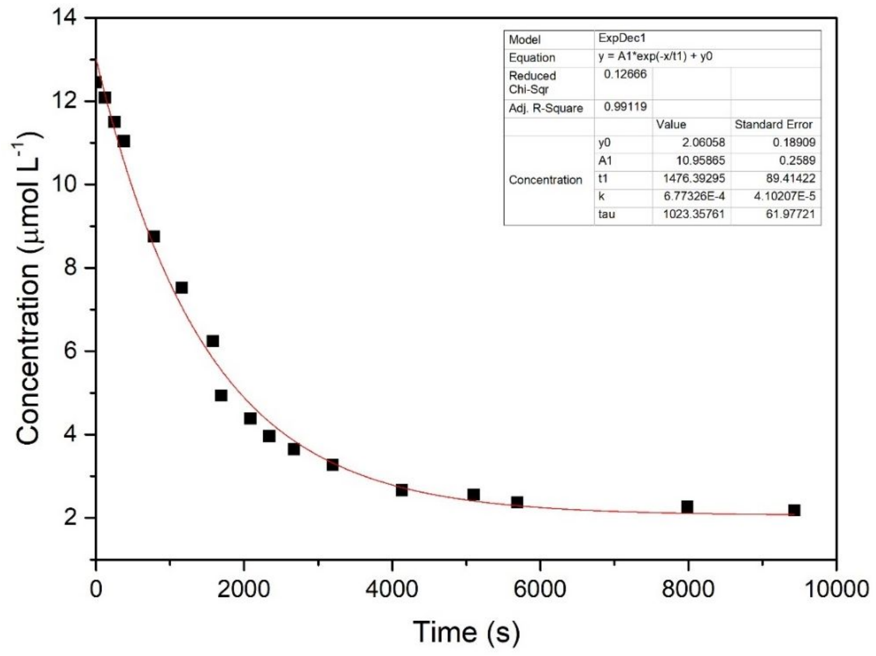

Figure S100. Plot of concentration of **4a** during the course of irradiation.

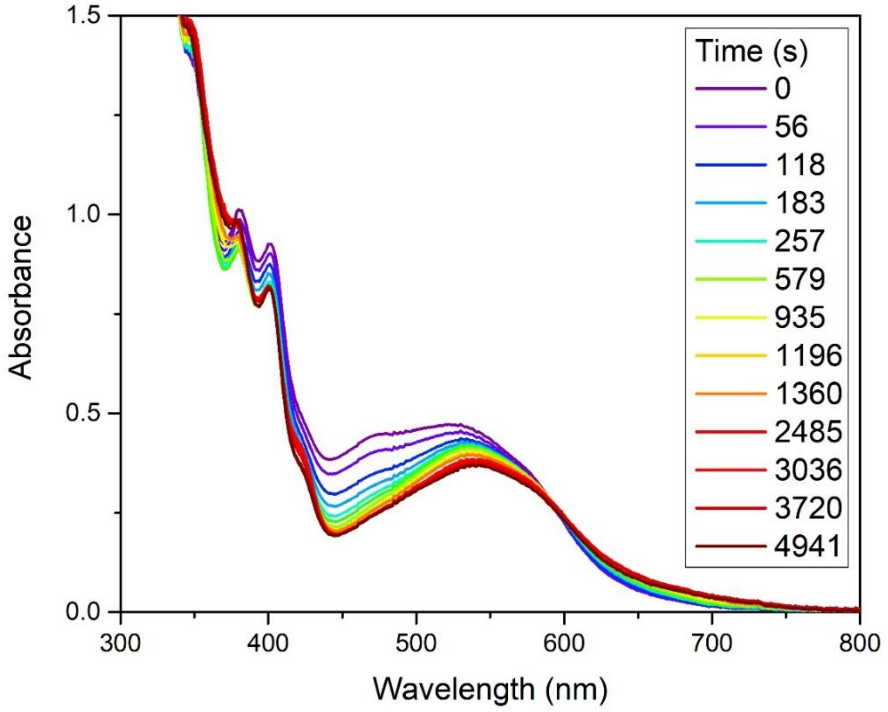

Figure S101. UV-Visible spectra of **4b** recorded during irradiation.

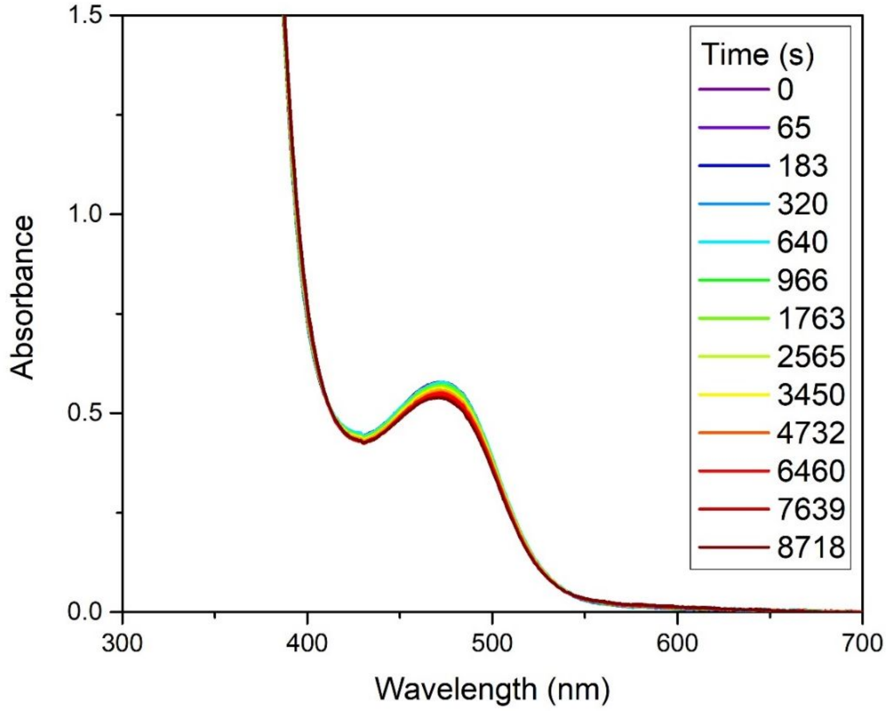

Figure S102. UV-Visible spectra of **5a** recorded during irradiation.

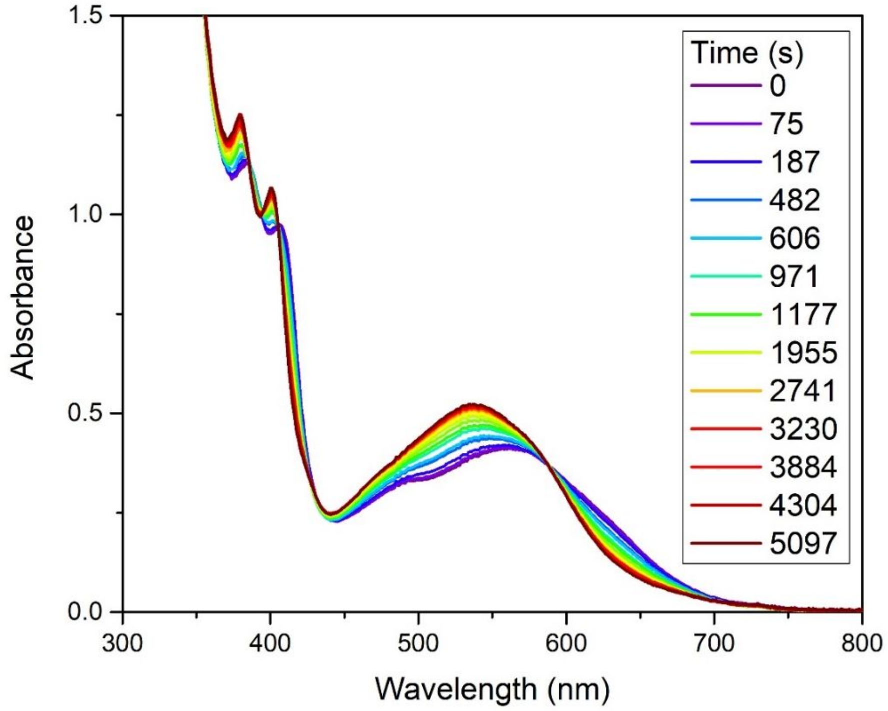

Figure S103. UV-Visible spectra of **5b** recorded during irradiation.

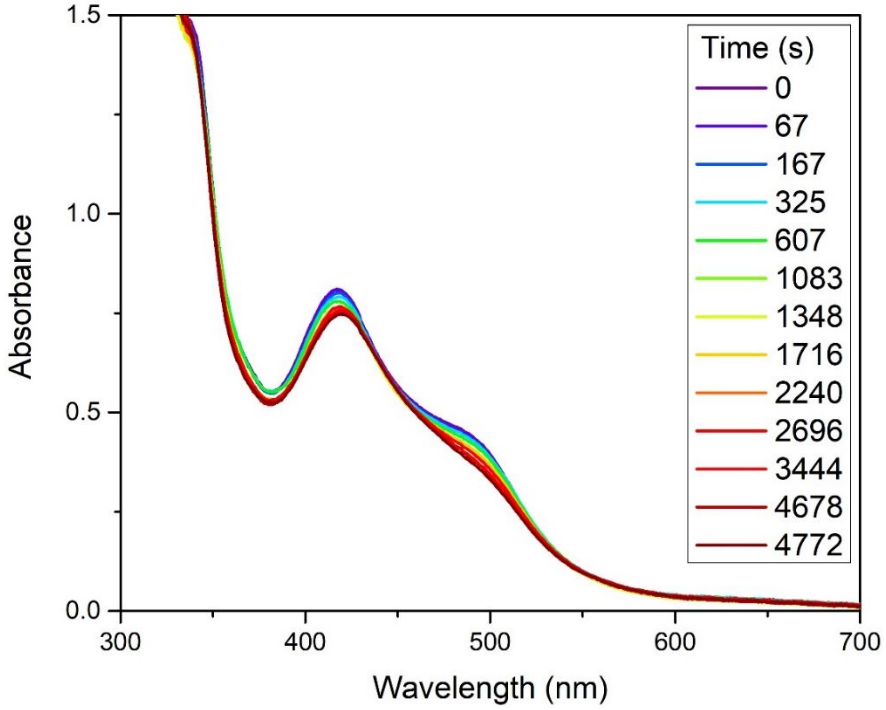

Figure S104. UV-Visible spectra of **5c** recorded during irradiation.

Photodissociation – NMR

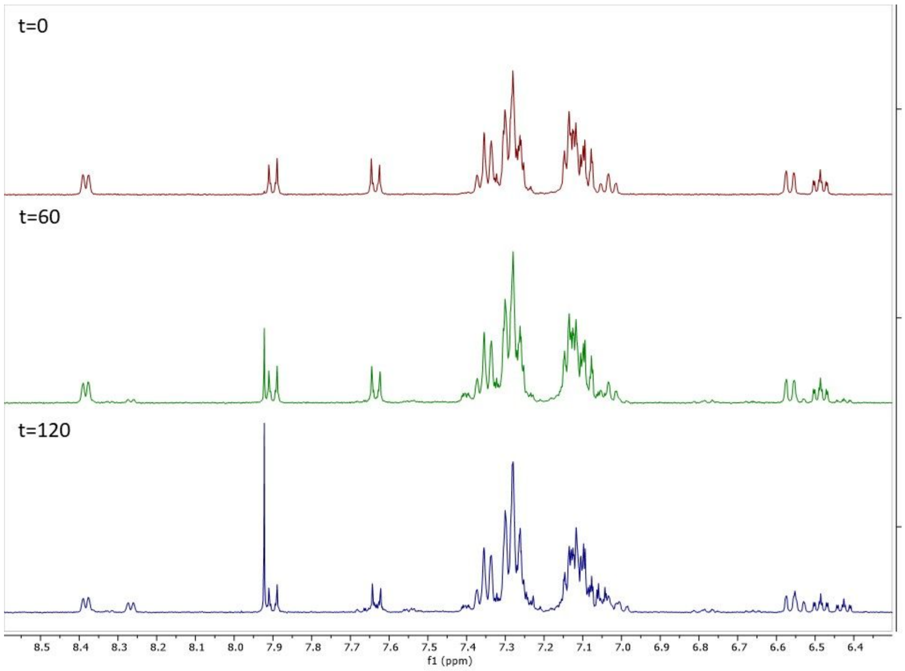

Figure S105. ^{1}H NMR spectra of **1a** recorded in CD_3CN after 0, 60, and 120 seconds irradiation.

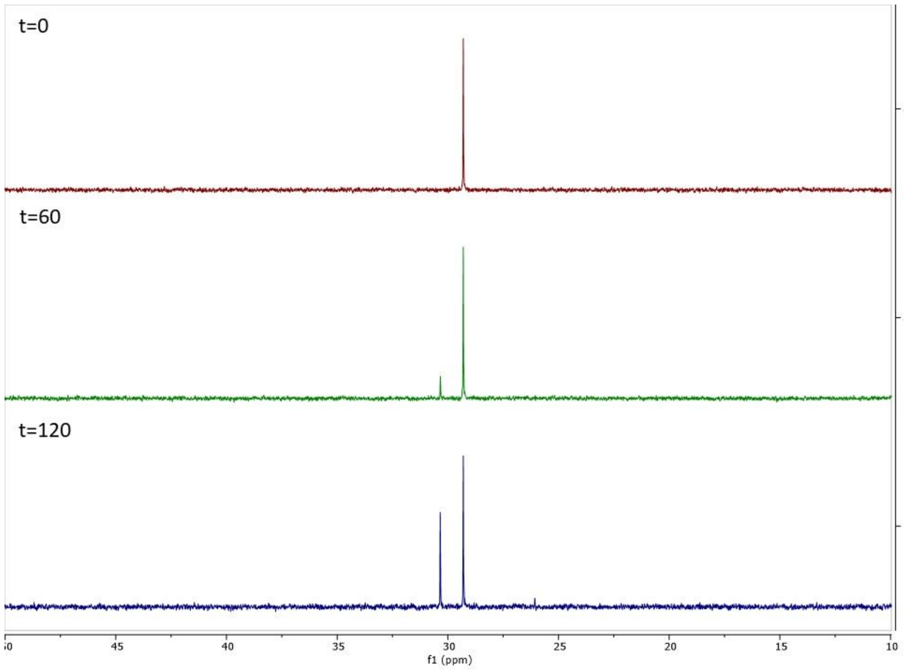

Figure S106. ^{13}P NMR spectra of **1a** recorded in CD_3CN after 0, 60, and 120 seconds irradiation.

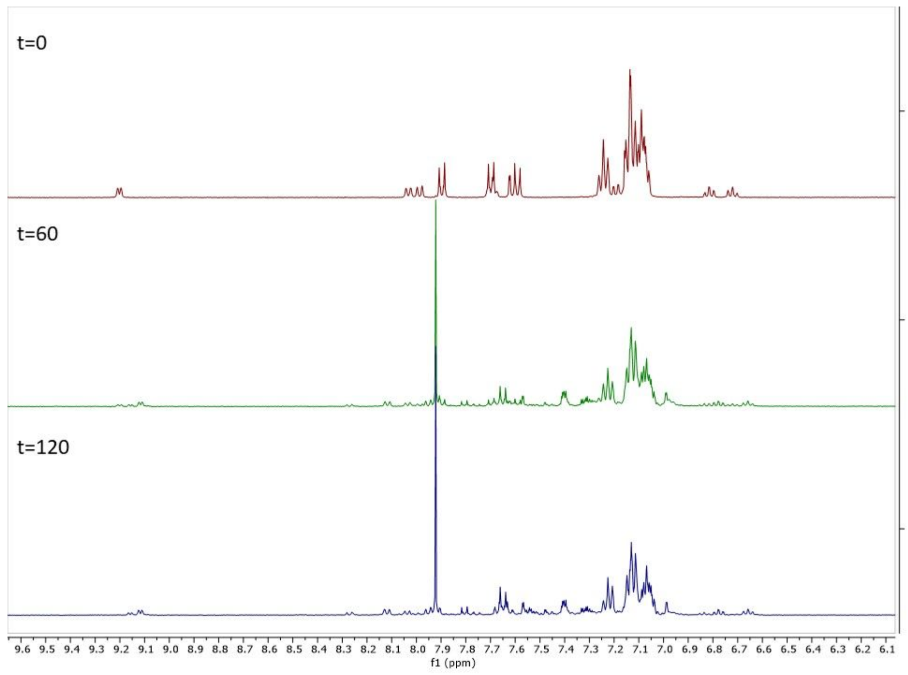

Figure S107. ^{1}H NMR spectra of **1c** recorded in CD_3CN after 0, 60, and 120 seconds irradiation.

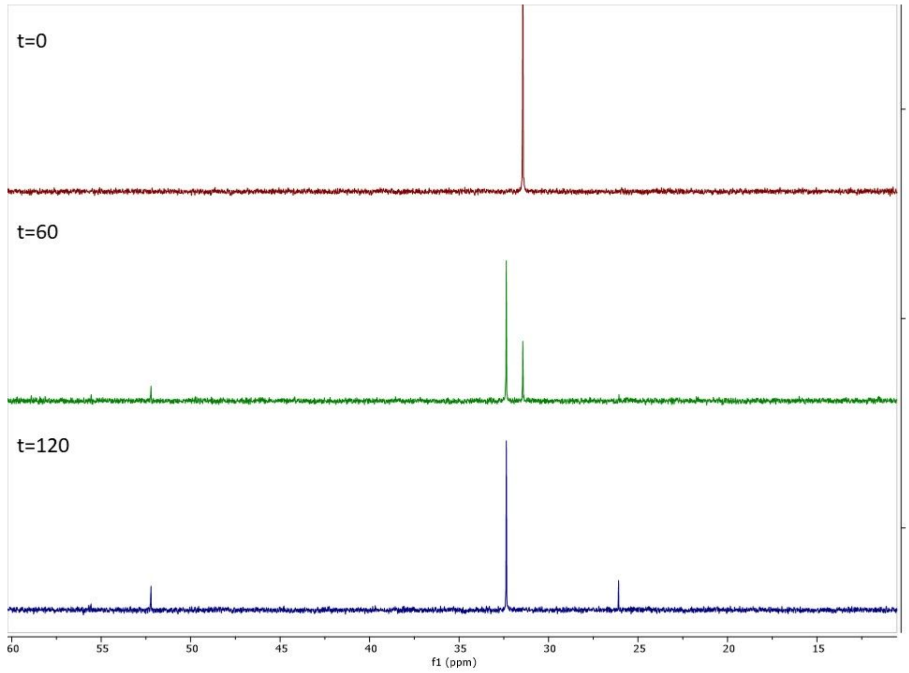

Figure S108. ^{13}P NMR spectra of **1c** recorded in CD_3CN after 0, 60, and 120 seconds irradiation.

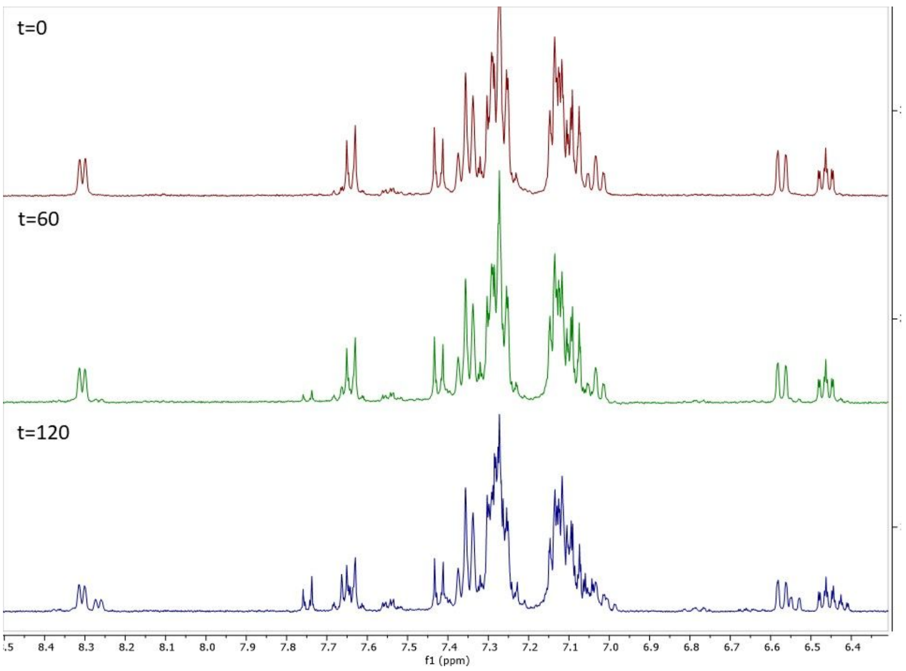

Figure S109. ^{1}H NMR spectra of **2a** recorded in CD_3CN after 0, 60, and 120 seconds irradiation.

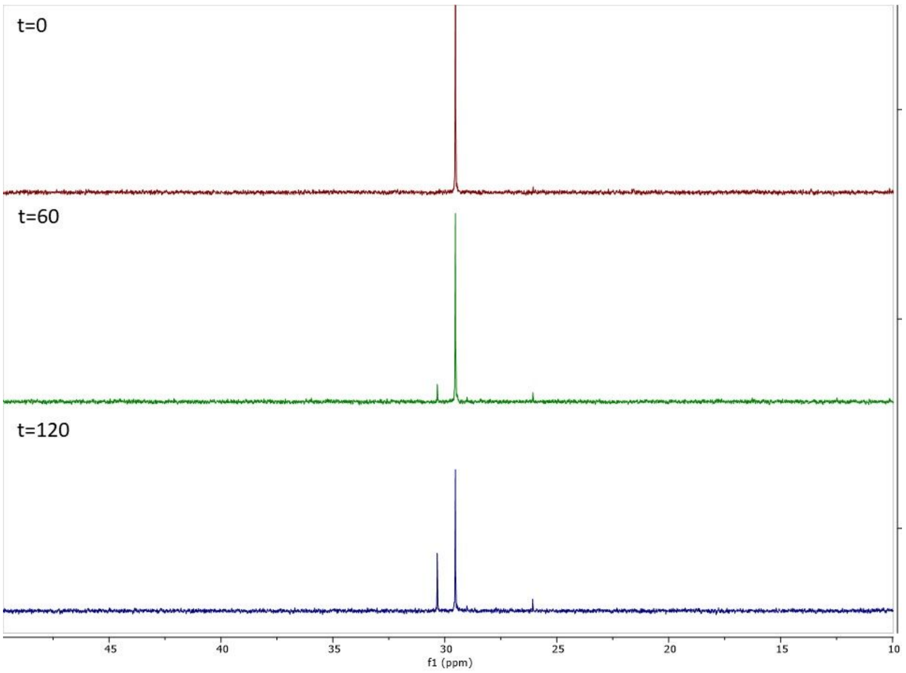

Figure S110. ^{13}P NMR spectra of **2a** recorded in CD_3CN after 0, 60, and 120 seconds irradiation.

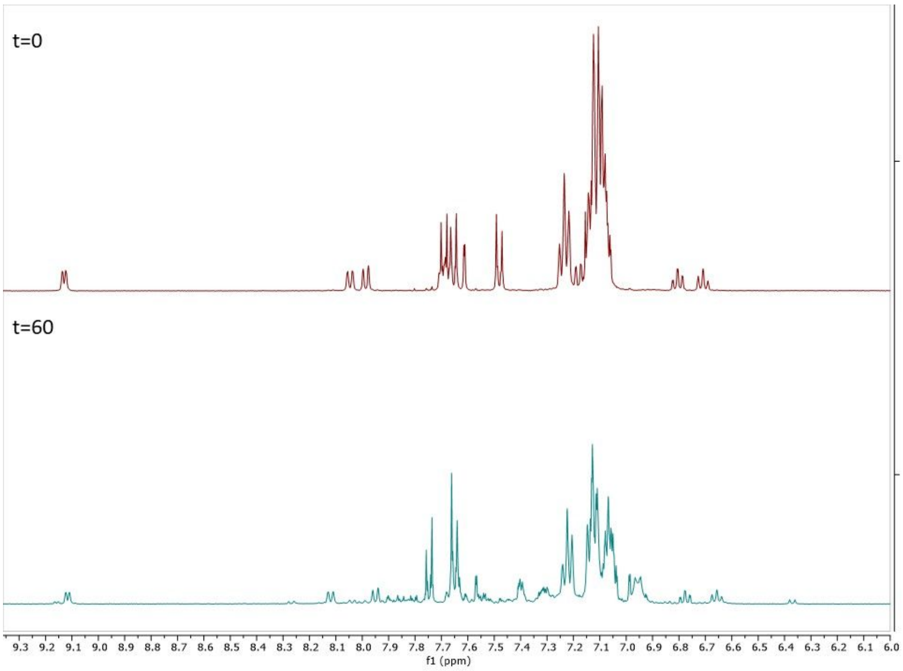

Figure S111. ^{1}H NMR spectra of **2c** recorded in CD_3CN after 0, and 60 seconds irradiation.

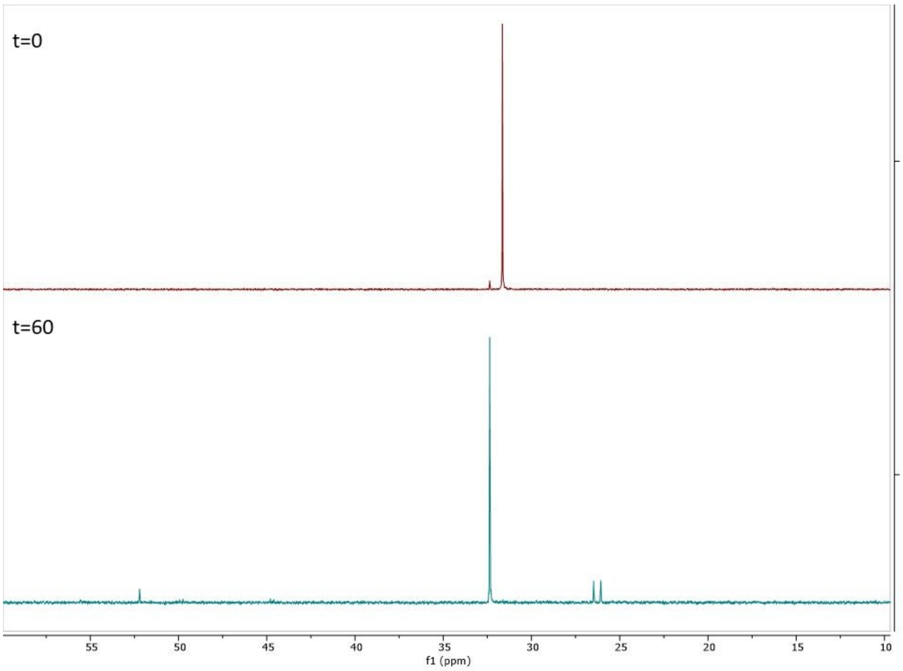

Figure S112. ^{13}P NMR spectra of **2c** recorded in CD_3CN after 0, and 60 seconds irradiation.

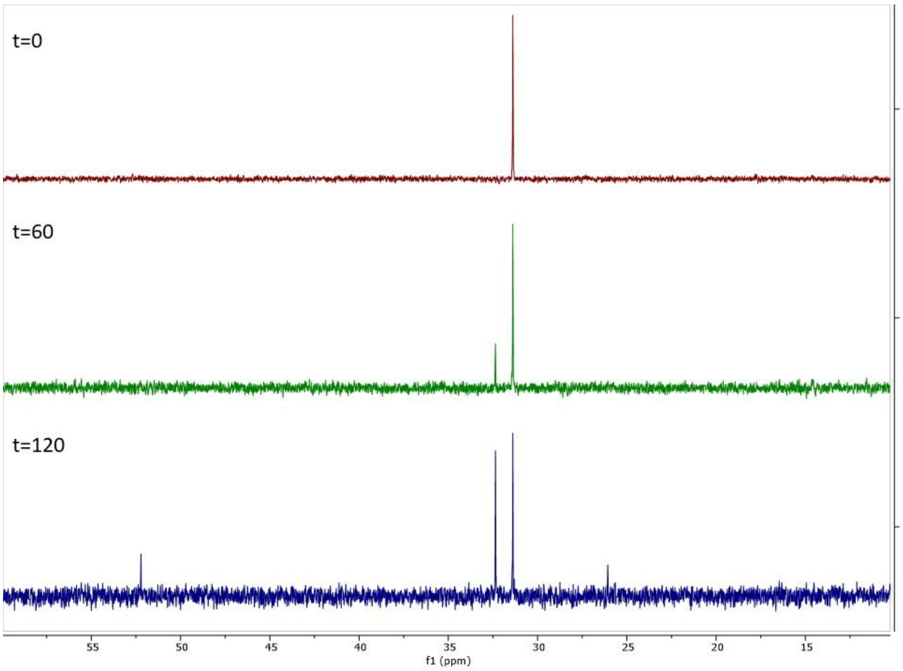

Figure S113. [13]P NMR spectra of **4c** recorded in CD_3CN after 0, 60, and 120 seconds irradiation.

S5. DFT Calculations

Orbital character

Table S6. Orbital contributions of **1a**

Orbitals	Energy (eV)	Contributions (%)			
		Ru	dpp	nitrile	PPh_3
L+1	-1.97	3	93	2	2
LUMO	-2.70	4	6	91	0
HOMO	-5.49	7	90	0	2
H-1	-5.58	30	62	3	6

Table S7. Orbital contributions of **1c**

Orbitals	Energy (eV)	Contributions (%)			
		**Ru**	**Pbpy**	**nitrile**	**PPh_3**
L+1	-2.31	3	92	3	2
LUMO	-2.60	5	4	91	0
HOMO	-5.59	50	44	2	3
H-1	-5.68	68	17	13	2

Table S8. Orbital contributions of **2a**

Orbitals	Energy (eV)	Contributions (%)			
		**Ru**	**dpp**	**nitrile**	**PPh_3**
L+1	-1.93	3	91	4	2
LUMO	-2.33	3	4	93	1
HOMO	-5.46	9	88	0	2
H-1	-5.52	30	61	3	5

Table S9. Orbital contributions of **2b**

**Orbitals**	**Energy (eV)**	**Contributions (%)**			
		**Ru**	**bpi**	**nitrile**	**PPh_3**
L+1	-2.40	3	1	96	1
LUMO	-2.70	3	96	0	1
HOMO	-5.68	32	58	3	7
H-1	-6.22	72	18	8	2

Table S10. Orbital contributions of **2c**

**Orbitals**	**Energy (eV)**	**Contributions (%)**			
		**Ru**	**Pbpy**	**Nitrile**	**PPh_3**
L+1	-2.19	5	22	72	5
LUMO	-2.31	2	73	22	2
HOMO	-5.51	56	36	6	56
H-1	-5.58	64	24	10	64

Table S11. Orbital contributions of **3a**

**Orbitals**	**Energy (eV)**	**Contributions (%)**			
		**Ru**	**dpp**	**nitrile**	**PPh_3**
L+1	-1.11	28	5	0	67
LUMO	-1.57	4	93	0	2
HOMO	-5.01	43	48	4	5
H-1	-5.05	66	9	25	1

Table S12. Orbital contributions of **3c**

**Orbitals**	**Energy (eV)**	**Contributions (%)**			
		**Ru**	**Pbpy**	**nitrile**	**PPh_3**
L+1	-1.39	2	96	0	2
LUMO	-1.95	4	93	0	2
HOMO	-4.88	75	12	12	1
H-1	-5.06	55	41	0	3

Table S13. Orbital contributions of **4a**

**Orbitals**	**Energy (eV)**	**Contributions (%)**			
		**Ru**	**dpp**	**nitrile**	**PPh_3**
L+1	-2.10	3	93	1	3
LUMO	-3.03	7	2	90	1
HOMO	-5.60	7	90	0	2
H-1	-5.61	6	92	0	2

Table S14. Orbital contributions of **4b**

**Orbitals**	**Energy (eV)**	**Contributions (%)**			
		**Ru**	**bpi**	**nitrile**	**PPh_3**
L+1	-2.87	3	96	0	1
LUMO	-3.17	6	12	82	0
HOMO	-5.89	30	62	2	6
H-1	-5.89	29	62	2	6

Table S15. Orbital contributions of **4c**

**Orbitals**	**Energy (eV)**	**Contributions (%)**			
		**Ru**	**Pbpy**	**nitrile**	**PPh_3**
L+1	-2.42	3	94	1	2
LUMO	-2.82	10	4	86	0
HOMO	-5.61	69	18	13	1
H-1	-5.70	47	48	1	4

Table S16. Orbital contributions of **5a**

**Orbitals**	**Energy (eV)**	**Contributions (%)**			
		**Ru**	**dpp**	**nitrile**	**PPh_3**
L+1	-1.76	4	93	0	3
LUMO	-1.77	3	93	1	3
HOMO	-5.12	44	43	10	3
H-1	-5.27	29	65	2	4

Table S17. Orbital contributions of **5b**

**Orbitals**	**Energy (eV)**	**Contributions (%)**			
		**Ru**	**bpi**	**nitrile**	**PPh_3**
L+1	-2.54	4	96	0	1
LUMO	-2.55	4	95	0	1
HOMO	-5.29	48	39	10	3
H-1	-5.46	37	55	2	6

Table S18. Orbital contributions of **5c**

**Orbitals**	**Energy (eV)**	**Contributions (%)**			
		**Ru**	**Pbpy**	**nitrile**	**PPh_3**
L+1	-2.11	4	94	0	2
LUMO	-2.13	4	94	0	2
HOMO	-5.05	74	13	12	1
H-1	-5.10	73	13	14	1

Electronic spectra assignment

On the basis that the computational models are valid, the two lowest energy singlet ($S_0 \rightarrow S_n$) and triplet ($S_0 \rightarrow T_n$) transitions were calculated for the optimised structure of each complex to aid in assigning the both the assignment of the transitions associated with the CT band and their potential involvement in the photodissociation of the nitrile ligand.

Table S19. Assignment of the transitions for **1a**

**Energy (eV)**	**Wavelength (nm)**	**Osc. Strength**	**Symmetry**	**Major Contributions (%)**		**Assignment**	
2.2046	562.388	0.000	Triplet	H-2 → L+2	69	Ru(d) → Ru(d) + $PPh_3(\sigma^*)$	^{3}MLCT
				H-2 → L+14	11	Ru(d) → Ru(d) + $PPh_3(\sigma^*)$	
2.2613	548.28	0.000	Triplet	H-2 → LUMO	35	Ru(d) → nitrile(π*)	^{3}MLLCT
				H-1 → LUMO	38	Ru(d) + dpp(π) → nitrile(π*)	
				HOMO → LUMO	13	dpp(π) → nitrile(π*)	
2.3121	536.24	0.026	Singlet	H-1 → LUMO	52	Ru(d) + dpp(π) → nitrile(π*)	^{1}MLLCT
				HOMO → LUMO	43	dpp(π) → nitrile(π*)	
2.3291	532.32	0.004	Singlet	H-1 → LUMO	42	Ru(d) + dpp(π) → nitrile(π*)	^{1}MLLCT
				HOMO → LUMO	55	dpp(π) → nitrile(π*)	

Table S20. Assignment of the transitions for **1c**

**Energy (eV)**	**Wavelength (nm)**	**Osc. Strength**	**Symmetry**	**Major Contributions (%)**		**Assignment**	
2.0150	615.30	0.000	Triplet	H-1 → LUMO	64	Ru(d) + Pbpy(π) + nitrile(π) → nitrile(π*)	^{3}MLLCT
				HOMO → LUMO	27	Ru(d) + Pbpy(π) → nitrile(π*)	
2.3698	523.18	0.000	Triplet	H-1 → LUMO	11	Ru(d) + Pbpy(π) + nitrile(π) → nitrile(π*)	^{3}MLLCT
				HOMO → LUMO	35	Ru(d) + Pbpy(π) → nitrile(π*)	
				HOMO → L+1	47	Ru(d) + Pbpy(π) → Pbpy(π*)	
2.4252	511.23	0.012	Singlet	HOMO → LUMO	87	Ru(d) + Pbpy(π) → nitrile(π*)	^{1}MLLCT
2.4382	508.50	0.008	Singlet	H-1 → LUMO	10	Ru(d) + Pbpy(π) + nitrile(π) → nitrile(π*)	^{1}MLLCT
				H-1 → L+1	28	Ru(d) + Pbpy(π) + nitrile(π) → Pbpy(π*)	
				HOMO → L+1	60	Ru(d) + Pbpy(π) → Pbpy(π*)	

Table S21. Assignment of the transitions for **2a**

Energy (eV)	Wavelength (nm)	Osc. Strength	Symmetry	Major Contributions (%)		Assignment	
2.1747	571.32	0.000	Triplet	H-2 → L+2	68	Ru(d) + dpp(π) + nitrile(π) → Ru(d) + PPh_3(σ*)	^{3}MLLCT
				H-2 → L+14	13	Ru(d) + dpp(π) + nitrile(π) → Ru(d) + PPh_3(σ*)	
2.3094	535.91	0.000	Triplet	H-5 → L+2	10	Ru(d) + PPh_3(σ) → Ru(d) + PPh_3(σ*)	^{3}MLLCT
				H-1 → L+2	58	Ru(d) + dpp(π) → Ru(d) + PPh_3(σ*)	
2.3368	530.86	0.044	Singlet	H-1 → LUMO	68	Ru(d) + dpp(π) → nitrile(π*)	^{1}MLLCT
				HOMO → LUMO	17	dpp(π) → nitrile(π*)	
2.3368	525.40	0.007	Singlet	H-3 → L+2	15	Ru(d) + dpp(π) → Ru(d) + PPh_3(σ*)	^{1}MLLCT
				H-1 → L+2	12	Ru(d) + dpp(π) → Ru(d) + PPh_3(σ*)	
				HOMO → LUMO	50	dpp(π) → nitrile(π*)	

Table S22. Assignment of the transitions for **2b**

**Energy (eV)**	**Wavelength (nm)**	**Osc. Strength**	**Symmetry**	**Major Contributions (%)**		**Assignment**	
1.8675	663.90	0.000	Triplet	HOMO → LUMO	96	Ru(d) + bpi(π) → bpi(π*)	^{3}MLCT
2.3306	531.98	0.085	Singlet	HOMO → LUMO	93	Ru(d) + bpi(π) → bpi(π*)	^{1}MLCT
2.3393	530.00	0.000	Triplet	H-3 → L+2	14	Ru(d) + bpi(π) → Ru(d) + PPh_3(σ*)	^{3}MLLCT
				HOMO → L+2	66	Ru(d) + bpi(π) → Ru(d) + PPh_3(σ*)	
2.4746	501.02	0.0109	Singlet	H-2 → LUMO	13	Ru(d) + bpi(π) → bpi(π*)	^{1}MLLCT
				H-1→ LUMO	49	Ru(d) + bpi(π) → bpi(π*)	
				HOMO → L+2	28	Ru(d) + bpi(π) → Ru(d) + PPh_3(σ*)	

Table S23. Assignment of the transitions for **2c**

Energy (eV)	Wavelength (nm)	Osc. Strength	Symmetry	Major Contributions (%)		Assignment	
2.2267	556.80	0.000	Triplet	H-1→ LUMO	11	Ru(d) + Pbpy(π) → Pbpy(π*) + nitrile(π*)	^{3}MLLCT
				H-1→ L+1	28	Ru(d) + Pbpy(π) → Pbpy(π*) + nitrile(π*)	
				HOMO → LUMO	19	Ru(d) + Pbpy(π) → Pbpy(π*) + nitrile(π*)	
				HOMO → L+1	30	Ru(d) + Pbpy(π) → Pbpy(π*) + nitrile(π*)	
2.3488	527.86	0.000	Triplet	HOMO → LUMO	65	Ru(d) + Pbpy(π) → Pbpy(π*) + nitrile(π*)	^{3}MLLCT
				HOMO → L+1	23	Ru(d) + Pbpy(π) → Pbpy(π*) + nitrile(π*)	
2.4406	508.00	0.0034	Singlet	H-1→ LUMO	12	Ru(d) + Pbpy(π) → Pbpy(π*) + nitrile(π*)	^{1}MLLCT
				HOMO → LUMO	69	Ru(d) + Pbpy(π) → Pbpy(π*) + nitrile(π*)	
				HOMO → L+1	15	Ru(d) + Pbpy(π) → Pbpy(π*) + nitrile(π*)	
2.4462	506.84	0.0069	Singlet	H-1→ LUMO	74	Ru(d) + Pbpy(π) → Pbpy(π*) + nitrile(π*)	^{1}MLLCT
				HOMO → LUMO	12	Ru(d) + Pbpy(π) → Pbpy(π*) + nitrile(π*)	

Table S24. Assignment of the transitions for **3a**

Energy (eV)	Wavelength (nm)	Osc. Strength	Symmetry	Major Contributions (%)		Assignment	
1.9413	638.66	0.000	Triplet	H-1 → L+1	57	Ru(d) + nitrile(π) → Ru(d) + PPh_3(σ*)	^{3}MLLCT
				H-1 → L+16	10	Ru(d) + nitrile(π) → Ru(d) + PPh_3(σ*)	
2.2803	543.71	0.000	Triplet	HOMO → L+1	65	Ru(d) + dpp(π) → Ru(d) + PPh_3(σ*)	^{3}MLLCT
2.3480	528.04	0.000	Singlet	H-2 → LUMO	11	Ru(d) + nitrile(π) → dpp(π*)	^{1}MLLCT
				H-1 → LUMO	26	Ru(d) + nitrile(π) → dpp(π*)	
				H-1→ L+1	40	Ru(d) + nitrile(π) → Ru(d) + PPh_3(σ*)	
2.4468	506.71	0.001	Singlet	H-1 → LUMO	70	Ru(d) + nitrile(π) → dpp(π*)	^{1}MLLCT
				H-1 → L+1	19	Ru(d) + nitrile(π) → Ru(d) + PPh_3(σ*)	

Table S25. Assignment of the transitions for **3c**

**Energy (eV)**	**Wavelength (nm)**	**Osc. Strength**	**Symmetry**	**Major Contributions (%)**		**Assignment**	
2.0443	606.48	0.000	Triplet	HOMO → LUMO	97	Ru(d) + Pbpy(π) + nitrile(π) → Pbpy(π*)	^{3}MLLCT
2.1185	585.24	0.000	Singlet	HOMO → LUMO	97	Ru(d) + Pbpy(π) + nitrile(π) → Pbpy(π*)	^{1}MLLCT
2.1912	565.82	0.000	Triplet	H-1 → LUMO	88	Ru(d) + Pbpy(π) → Pbpy(π*)	^{3}MLCT
2.3114	536.40	0.006	Singlet	H-1 → LUMO	89	Ru(d) + Pbpy(π) → Pbpy(π*)	1MLCT

Table S26. Assignment of the transitions for **4a**

Energy (eV)	Wavelength (nm)	Osc. Strength	Symmetry	Major Contributions (%)		Assignment	
2.0431	606.84	0.000	Triplet	H-4 → LUMO	50	Ru(d) + dpp(π) + nitrile(π) → nitrile(π*)	^{3}MLLCT
				H-2 → LUMO	35	Ru(d) + dpp(π) → nitrile(π*)	
2.1946	564.95	0.000	Triplet	H-5 → L+4	31	Ru(d) + dpp(π) + nitrile(π) → Ru(d) + PPh_3(σ*)	^{3}MLLCT
				H-4 → L+3	38	Ru(d) + dpp(π) + nitrile(π) → Ru(d) + PPh_3(σ*)	
2.2448	552.31	0.136	Singlet	H-2 → LUMO	41	Ru(d) + dpp(π) → nitrile(π*)	^{1}MLLCT
				HOMO → LUMO	55	dpp(π) → nitrile(π*)	
2.2726	545.56	0.000	Singlet	H-3 → LUMO	11	Ru(d) + dpp(π) → nitrile(π*)	^{1}MLLCT
				H-1 → LUMO	87	dpp(π) → nitrile(π*)	

Table S27. Assignment of the transitions for **4b**

Energy (eV)	Wavelength (nm)	Osc. Strength	Symmetry	Major Contributions (%)		Assignment	
1.8904	655.86	0.000	Triplet	H-1 → L+1	25	Ru(d) + bpi(π) → bpi(π*)	^{3}MLCT
				H-1 → L+2	23	Ru(d) + bpi(π) → bpi(π*)	
				HOMO → L+1	24	Ru(d) + bpi(π) → bpi(π*)	
				HOMO → L+2	24	Ru(d) + bpi(π) → bpi(π*)	
1.8905	655.82	0.000	Triplet	H-1 → L+1	23	Ru(d) + bpi(π) → bpi(π*)	^{3}MLCT
				H-1 → L+2	25	Ru(d) + bpi(π) → bpi(π*)	
				HOMO → L+1	24	Ru(d) + bpi(π) → bpi(π*)	
				HOMO → L+2	23	Ru(d) + bpi(π) → bpi(π*)	
2.3044	538.03	0.003	Singlet	HOMO → LUMO	97	Ru(d) + bpi(π) → bpi(π*) + nitrile(π*)	^{1}MLLCT
2.3118	536.31	0.000	Singlet	H-1 → LUMO	97	Ru(d) + bpi(π) → bpi(π*) + nitrile(π*)	^{1}MLLCT

Table S28. Assignment of the transitions for **4c**

Energy (eV)	Wavelength (nm)	Osc. Strength	Symmetry	Major Contributions (%)		Assignment	
1.8106	684.76	0.000	Triplet	HOMO → LUMO	88	Ru(d) + Pbpy(π) + nitrile(π) → nitrile(π*)	^{3}MLLCT
1.8106	571.93	0.000	Triplet	H-3 → LUMO	77	Ru(d) + Pbpy(π) + nitrile(π) → nitrile(π*)	^{3}MLLCT
				H-1 → LUMO	14	Ru(d) + Pbpy(π) → nitrile(π*)	
2.3627	524.75	0.772	Singlet	H-2 → LUMO	15	Ru(d) + Pbpy(π) → nitrile(π*)	^{1}MLLCT
				HOMO → LUMO	80	Ru(d) + Pbpy(π) + nitrile(π) → nitrile(π*)	
2.3697	523.20	0.000	Singlet	H-1 → LUMO	96	Ru(d) + Pbpy(π) → nitrile(π*)	^{1}MLLCT

Table S29. Assignment of the transitions for **5a**

Energy (eV)	Wavelength (nm)	Osc. Strength	Symmetry	Major Contributions (%)		Assignment	
2.0054	618.25	0.000	Triplet	H-5 → L+2	30	Ru(d) + dpp(π) → Ru(d) + PPh_3(σ*)	^{3}MLLCT
				H-4 → L+2	32	Ru(d) + dpp(π) + nitrile(π) → Ru(d) + PPh_3(σ*)	
2.0939	592.12	0.000	Triplet	H-5 → L+3	25	Ru(d) + dpp(π) → Ru(d) + PPh_3(σ*)	^{3}MLLCT
				H-4 → L+3	35	Ru(d) + dpp(π) + nitrile(π) → Ru(d) + PPh_3(σ*)	
2.2025	562.92	0.005	Singlet	H-1 → L+2	19	Ru(d) + dpp(π) → Ru(d) + PPh_3(σ*)	^{1}MLLCT
				HOMO → L+2	45	Ru(d) + dpp(π) + nitrile(π) → Ru(d) + PPh_3(σ*)	
2.2080	561.52	0.005	Singlet	H-1 → L+3	20	Ru(d) + dpp(π) → Ru(d) + PPh_3(σ*)	^{1}MLLCT
				HOMO → L+3	43	Ru(d) + dpp(π) + nitrile(π) → Ru(d) + PPh_3(σ*)	

Table S30. Assignment of the transitions for **5b**

Energy (eV)	Wavelength (nm)	Osc. Strength	Symmetry	Major Contributions (%)		Assignment	
1.7480	709.29	0.000	Triplet	H-1 → LUMO	38	Ru(d) + bpi(π) → bpi(π*)	^{3}MLLCT
				HOMO → LUMO	51	Ru(d) + bpi(π) + nitrile(π) → bpi(π*)	
1.7564	705.89	0.000	Triplet	H-1 → L+1	36	Ru(d) + bpi(π) → bpi(π*)	^{3}MLLCT
				HOMO → L+1	52	Ru(d) + bpi(π) + nitrile(π) → bpi(π*)	
2.1242	583.67	0.0161	Singlet	H-1 → LUMO	12	Ru(d) + bpi(π) → bpi(π*)	^{1}MLLCT
				H-1 → L+1	13	Ru(d) + bpi(π) → bpi(π*)	
				HOMO → LUMO	65	Ru(d) + bpi(π) + nitrile(π) → bpi(π*)	
2.1348	580.77	0.083	Singlet	H-1 → LUMO	13	Ru(d) + bpi(π) → bpi(π*)	^{1}MLLCT
				H-1 → L+1	11	Ru(d) + bpi(π) → bpi(π*)	
				HOMO → L+1	65	Ru(d) + bpi(π) + nitrile(π) → bpi(π*)	

Table S31. Assignment of the transitions for **5c**

**Energy (eV)**	**Wavelength (nm)**	**Osc. Strength**	**Symmetry**	**Major Contributions (%)**		**Assignment**	
2.0526	604.03	0.000	Triplet	H-1 → LUMO	11	Ru(d) + Pbpy(π) + nitrile(π) → nitrile(π*)	^{3}MLLCT
				HOMO → LUMO	71	Ru(d) + Pbpy(π) + nitrile(π) → nitrile(π*)	
				HOMO → L+1	10	Ru(d) + Pbpy(π) + nitrile(π) → nitrile(π*)	
2.0875	593.93	0.000	Triplet	H-1 → LUMO	10	Ru(d) + Pbpy(π) + nitrile(π) → nitrile(π*)	^{3}MLLCT
				H-1 → L+1	66	Ru(d) + Pbpy(π) + nitrile(π) → nitrile(π*)	
				HOMO → L+1	16	Ru(d) + Pbpy(π) + nitrile(π) → nitrile(π*)	
2.1249	583.48	0.002	Singlet	HOMO → LUMO	74	Ru(d) + Pbpy(π) + nitrile(π) → nitrile(π*)	^{1}MLLCT
				HOMO → L+1	10	Ru(d) + Pbpy(π) + nitrile(π) → nitrile(π*)	
2.1551	575.30	0.000	Singlet	H-1 → LUMO	10	Ru(d) + Pbpy(π) + nitrile(π) → nitrile(π*)	^{1}MLLCT
				H-1 → L+1	64	Ru(d) + Pbpy(π) + nitrile(π) → nitrile(π*)	
				HOMO → L+1	18	Ru(d) + Pbpy(π) + nitrile(π) → nitrile(π*)	

Ruthenium-nitrile bond dissociation

To assess the energy associated with the ruthenium-nitrile bond dissociation a scan was performed along the Ru-$N_{nitrile}$ bond sequentially increasing the distance in 0.02 Å steps. The bond length started at the unrestricted optimised length and was extended to 4.00 Å a distance greater than the sum of Van der Waal radii of ruthenium and nitrogen, at this distance it is reasonable to assume that there is no bond between the nitrile group and ruthenium atom. In the bi-metallic cases only a single Ru-$N_{nitrile}$ bond distance was extended leaving the second to freely optimise.

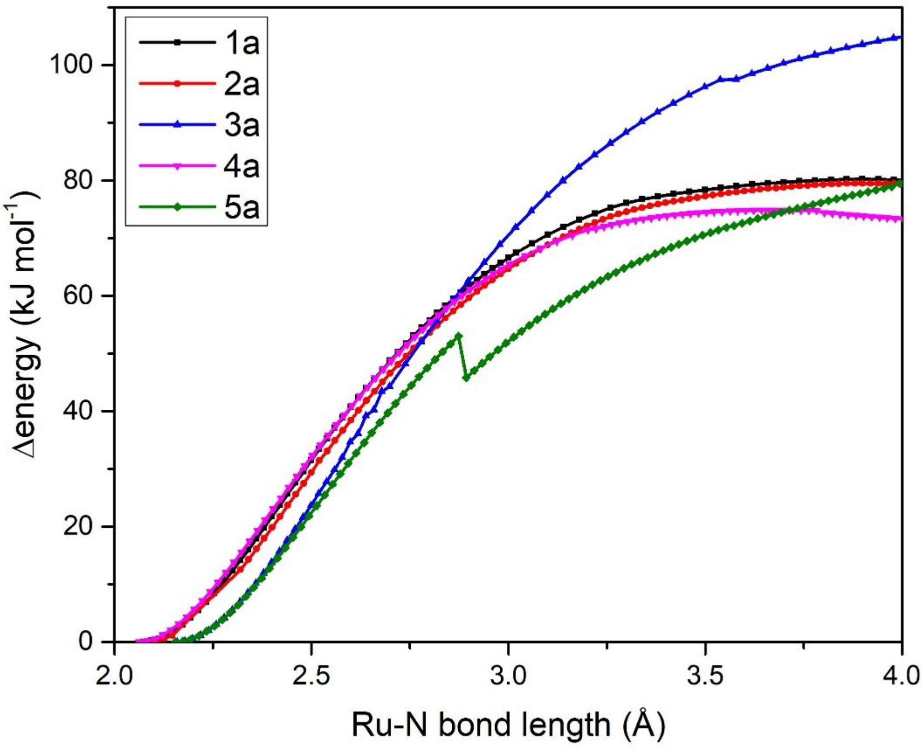

Figure S114. Energy difference vs. bond length for the dpp complexes (**1a**, **2a**, **3a**, **4a**, and **5a**).

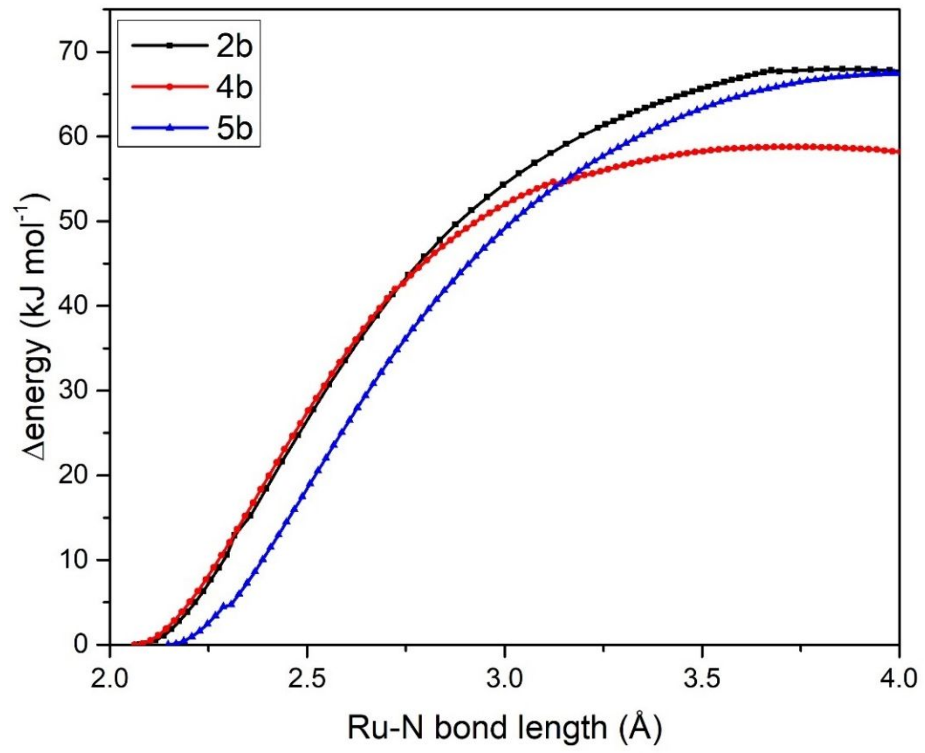

Figure S115. Energy difference vs. bond length for the bpi complexes (**2b**, **4b**, and **5b**).

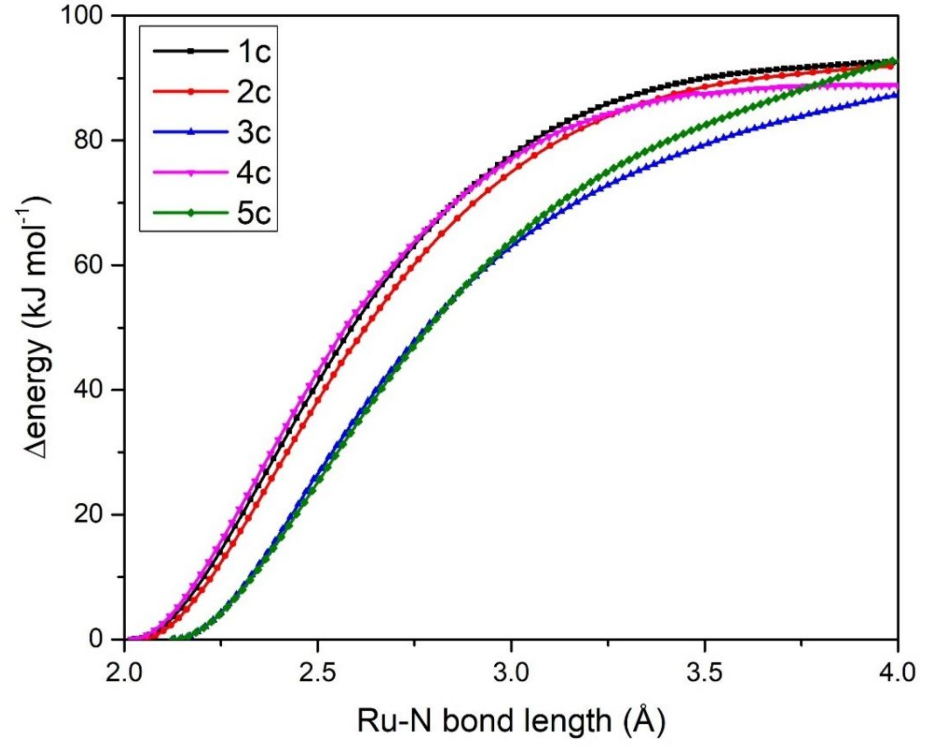

Figure S116. Energy difference vs. bond length for the Pbpy complexes (**1c**, **2c**, **3c**, **4c**, and **5c**).

Table S32 displays the energy difference (ΔE) between the fully optimised structure and that of the corresponding structure where the $Ru-N_{nitrile}$ has been extended to 4 Å.

Table S32. ΔE of complexes at length 4 Å.

**Complex**	**ΔE (kJ mol^{-1})**
1a	80.08
1c	92.53
2a	79.45
2b	67.68
2c	91.88
3a	105.15
3c	87.40
4a	73.33
4b	58.19
4c	88.84
5a	79.47
5b	67.45
5c	92.68

Singlet-Triplet energy

Given the T_1 plays a critical role in the dissociation of the Ru-$N_{nitrile}$ a comparison of S_1 and T_1 were calculated as the Ru-$N_{nitrile}$ bond length is varied.

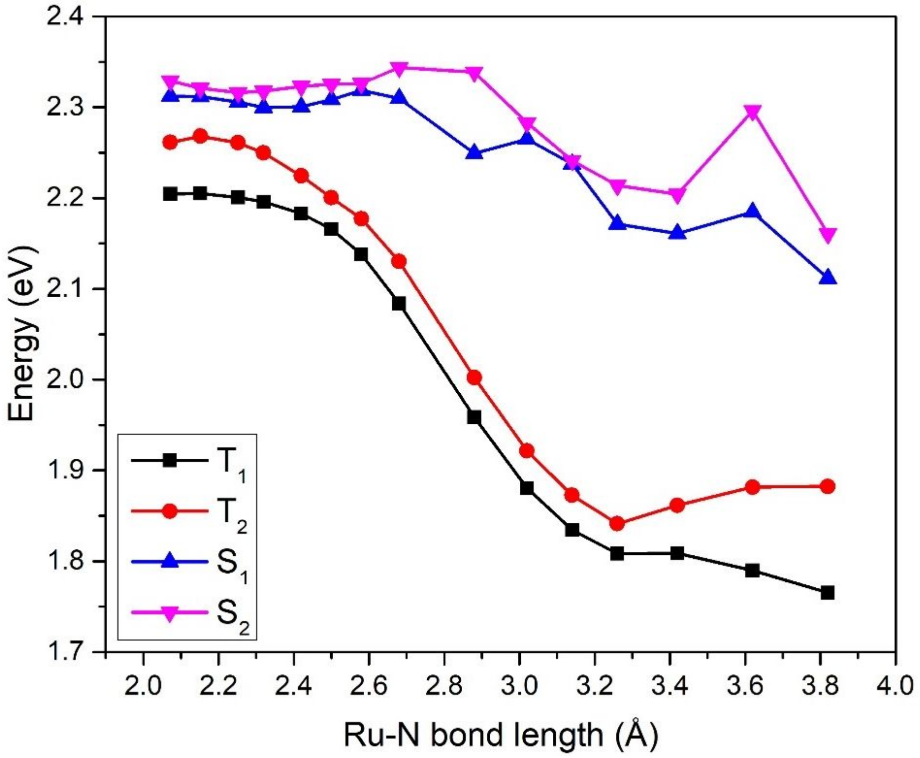

Figure S117. T_1, T_2, S_1, and S_2 energy levels versus Ru-$N_{nitrile}$ bond length for complex **1a**.

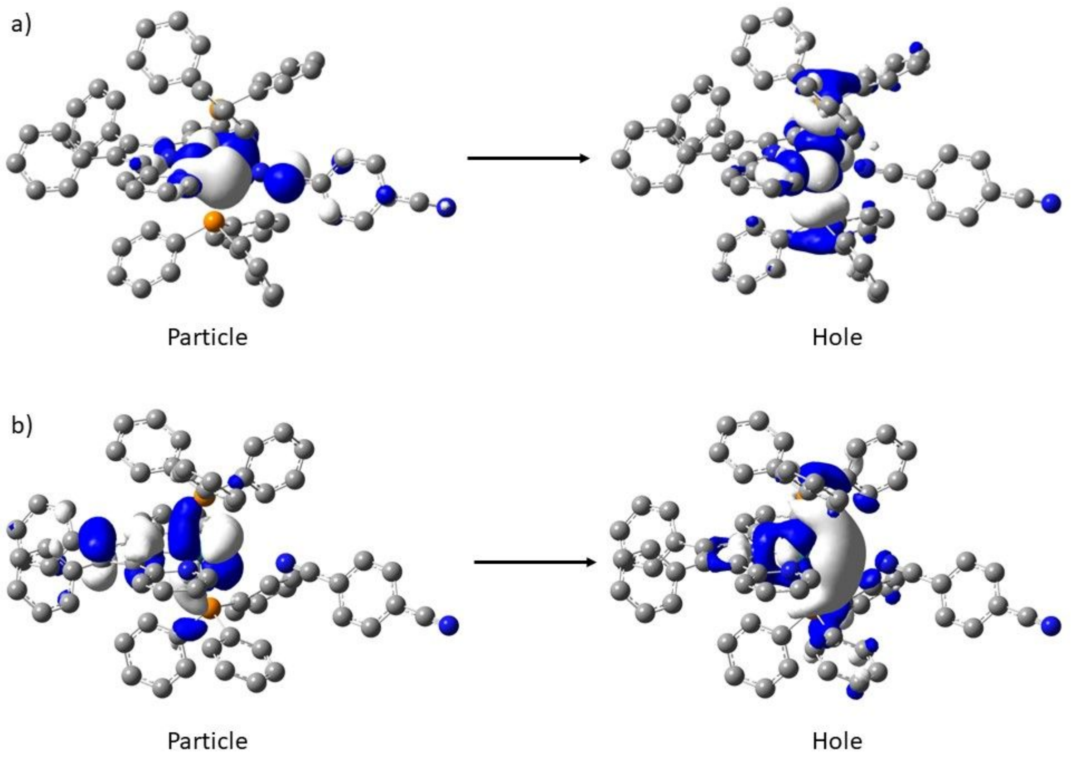

Figure S118. Plot of natural transition orbitals of **1a** for $S_0 \rightarrow T_1$ for Ru-$N_{nitrile}$ length: a) 2.07 Å and b) 3.82 Å.

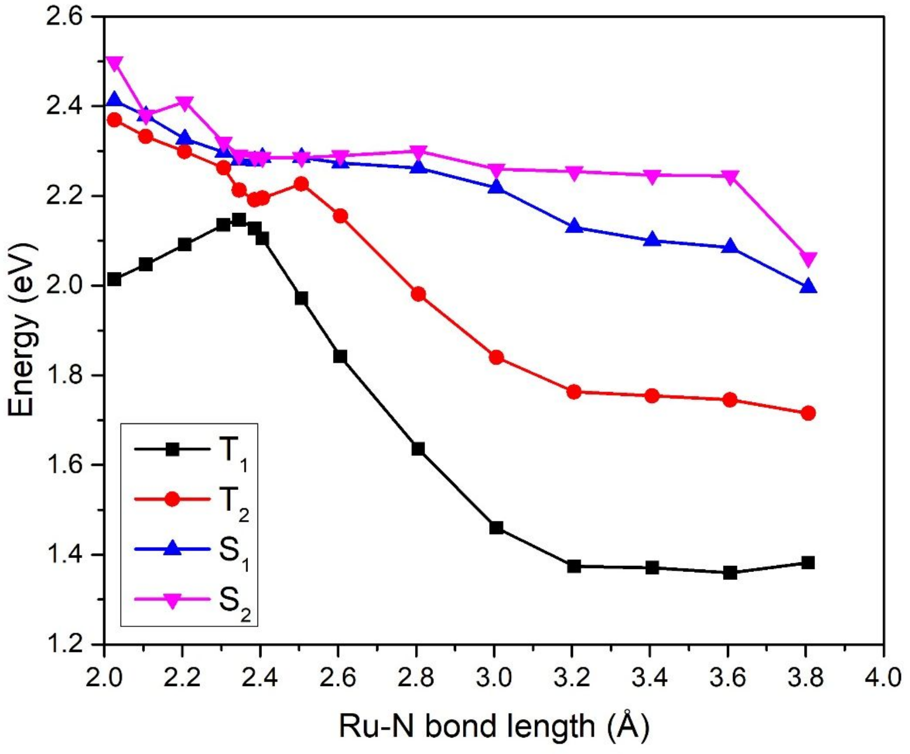

Figure S119. T_1, T_2, S_1, and S_2 energy levels versus Ru-$N_{nitrile}$ bond length for complex **1c**.

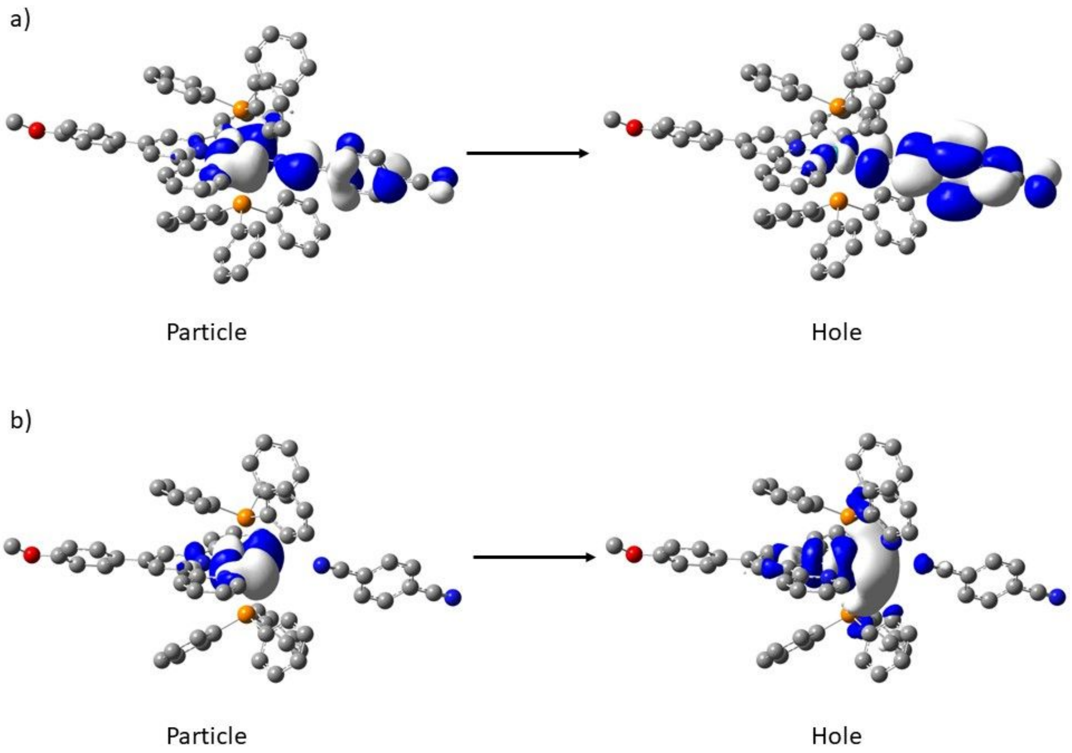

Figure S120. Plot of natural transition orbitals of **1c** for $S_0 \rightarrow T_1$ for Ru-$N_{nitrile}$ length: a) 2.08 Å and b) 3.80 Å.

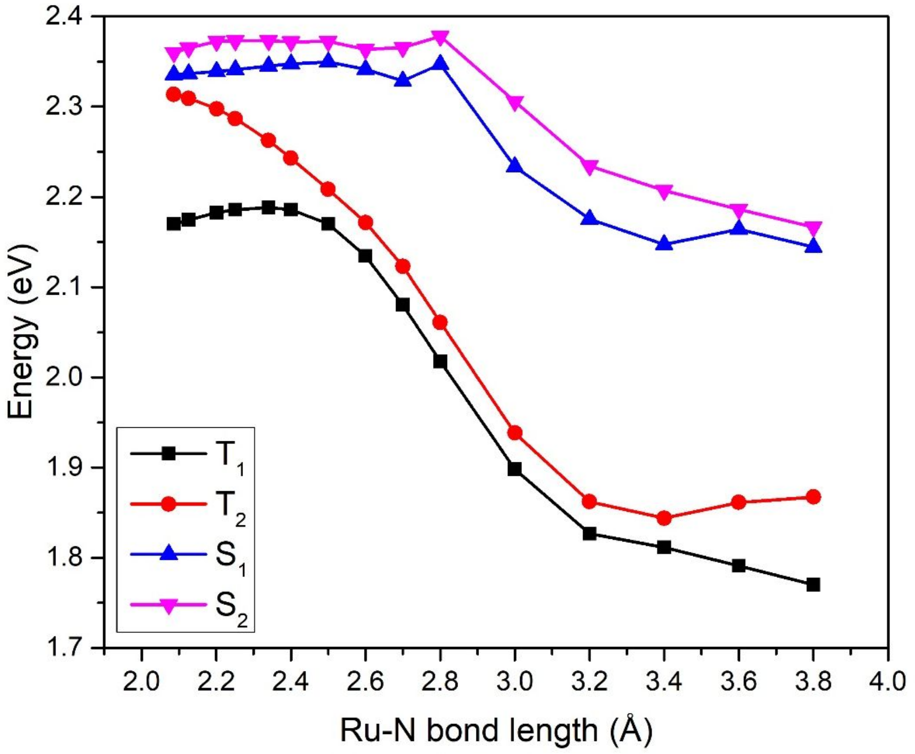

Figure S121. T_1, T_2, S_1, and S_2 energy levels versus Ru-$N_{nitrile}$ bond length for complex **2a**.

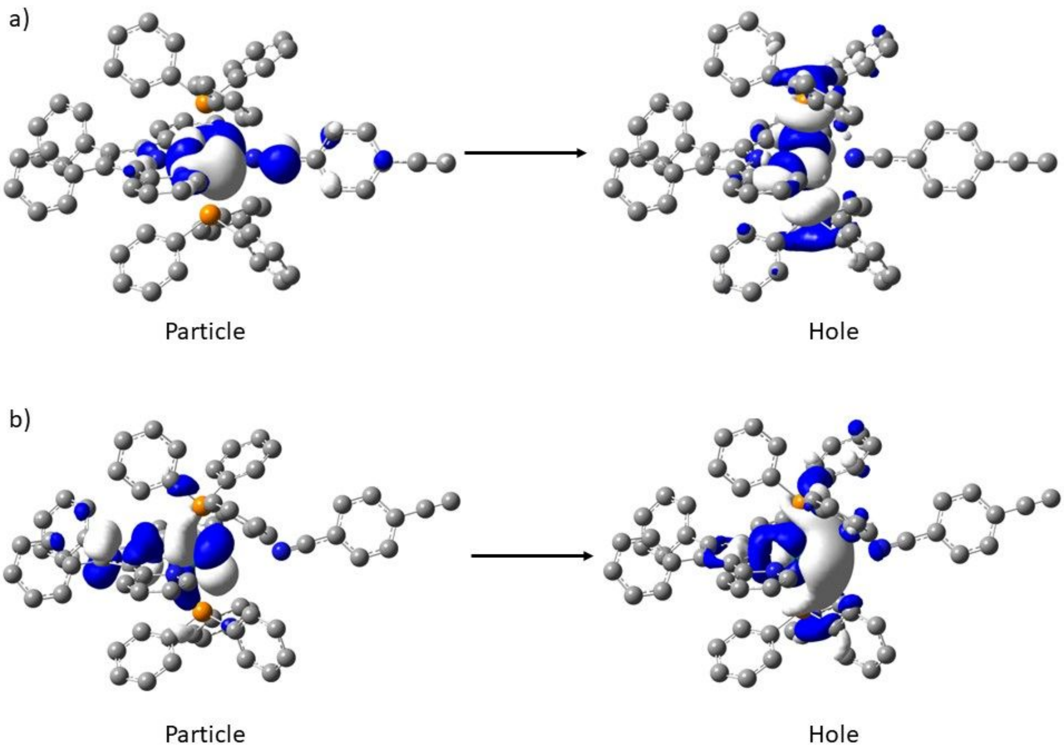

Figure S122. Plot of natural transition orbitals of **2a** for $S_0 \rightarrow T_1$ for Ru-N$_{nitrile}$ length: a) 2.06 Å and b) 3.80 Å.

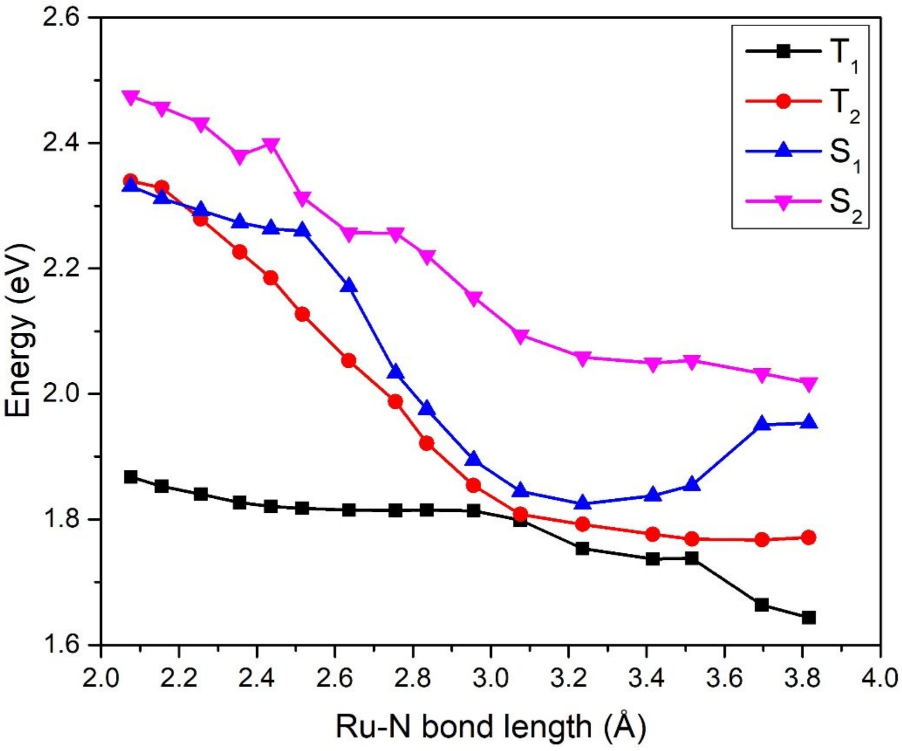

Figure S123. T_1, T_2, S_1, and S_2 energy levels versus Ru-N$_{nitrile}$ bond length for complex **2b**.

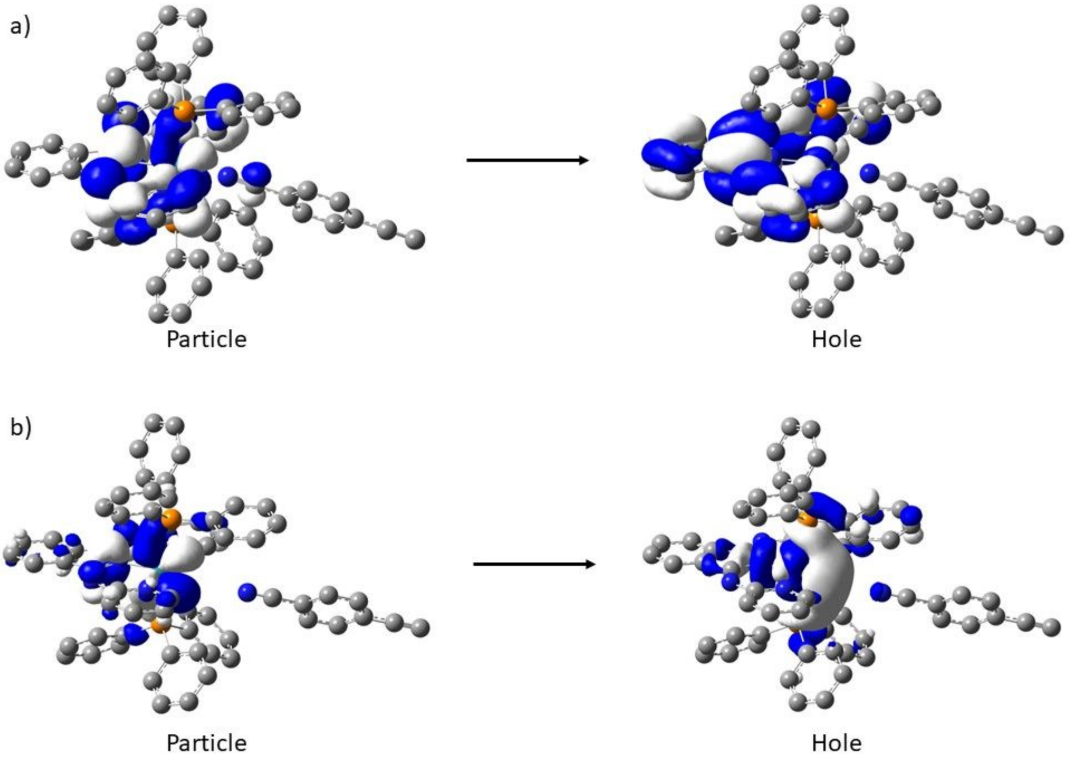

Figure S124. Plot of natural transition orbitals of **2b** for $S_0 \rightarrow T_1$ for Ru-$N_{nitrile}$ length: a) 2.07 Å and b) 3.81 Å.

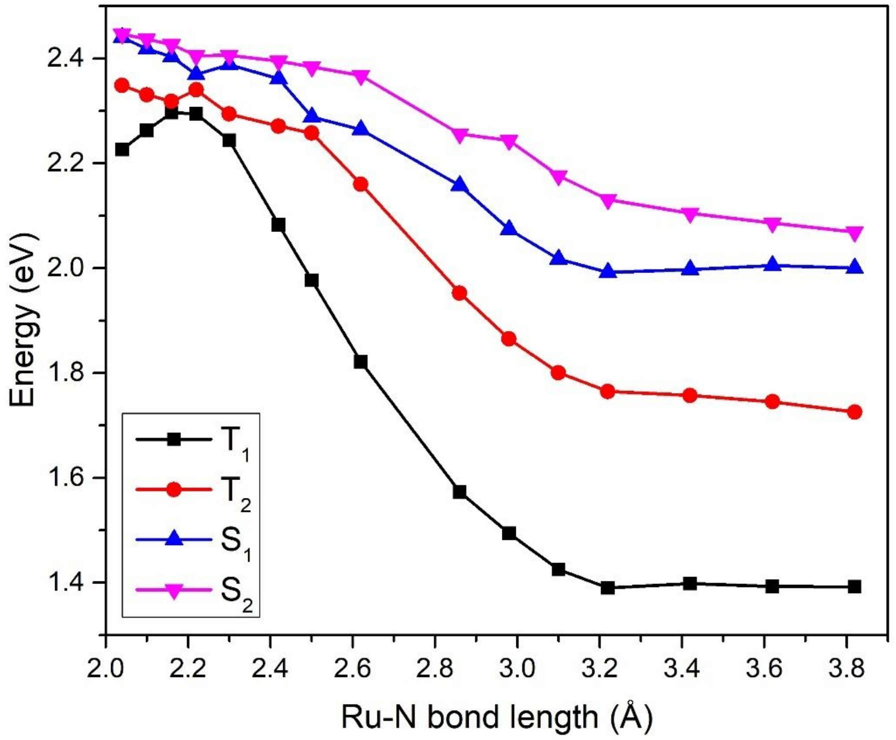

Figure S125. T_1, T_2, S_1, and S_2 energy levels versus Ru-$N_{nitrile}$ bond length for complex **2c**.

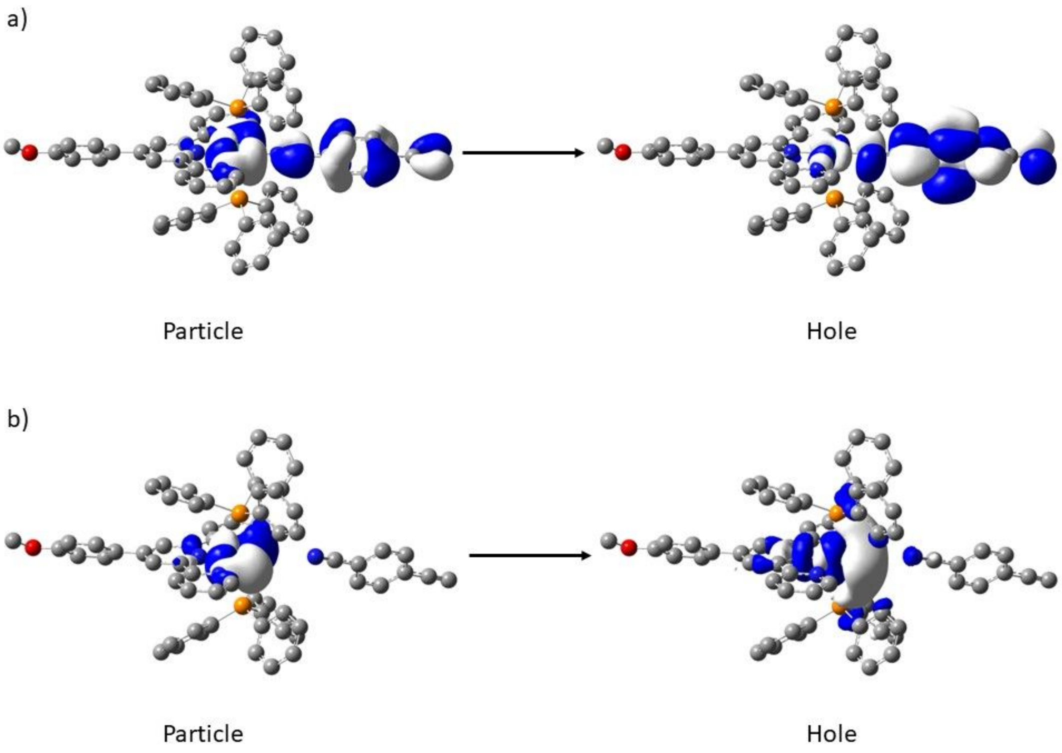

Figure S126. Plot of natural transition orbitals of **2c** for $S_0 \rightarrow T_1$ for Ru-$N_{nitrile}$ length: a) 2.04 Å and b) 3.82 Å.

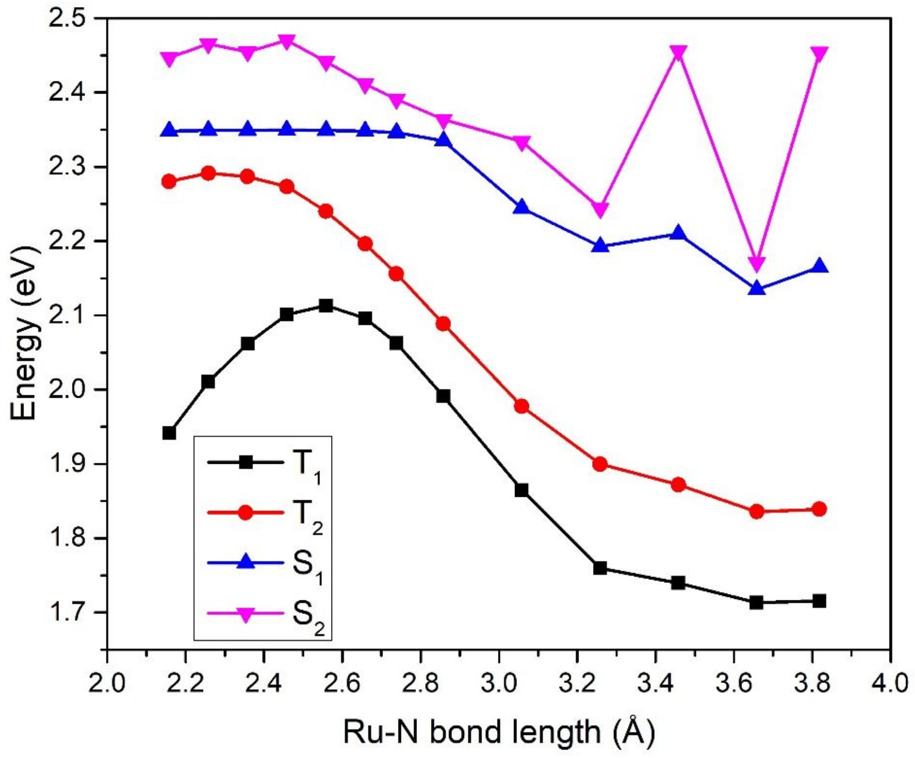

Figure S127. T_1, T_2, S_1, and S_2 energy levels versus Ru-$N_{nitrile}$ bond length for complex **3a**.

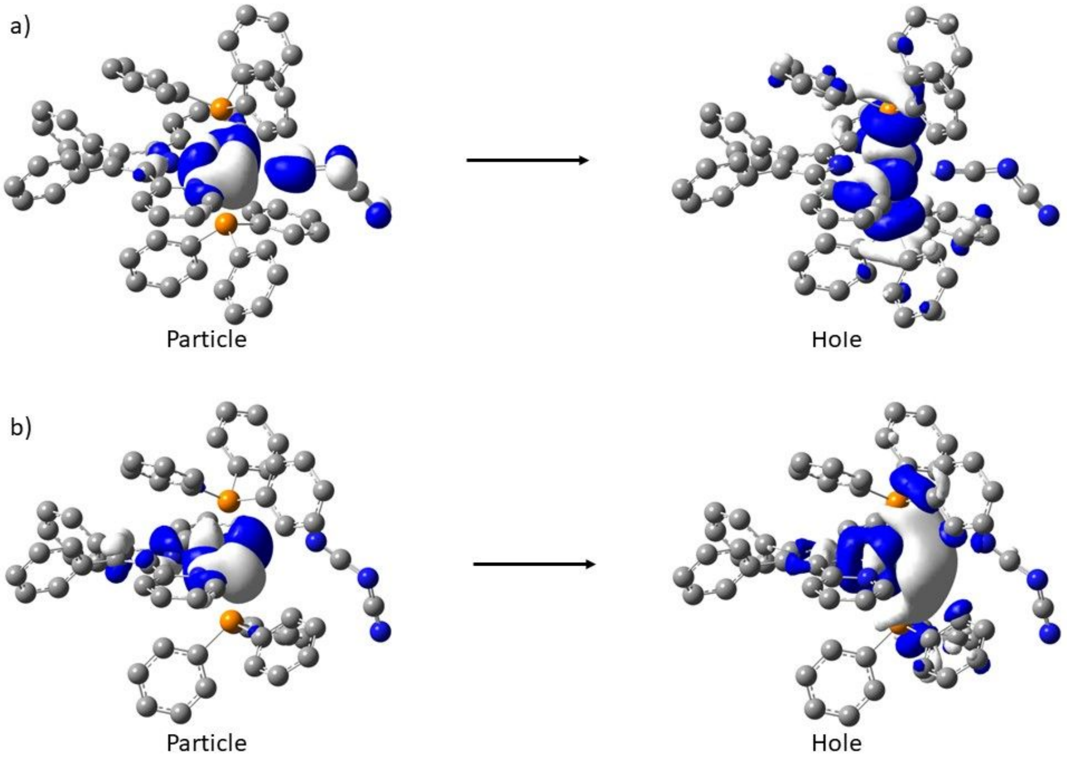

Figure S128. Plot of natural transition orbitals of **3a** for $S_0 \rightarrow T_1$ for Ru-$N_{nitrile}$ length: a) 2.15 Å and b) 3.81 Å.

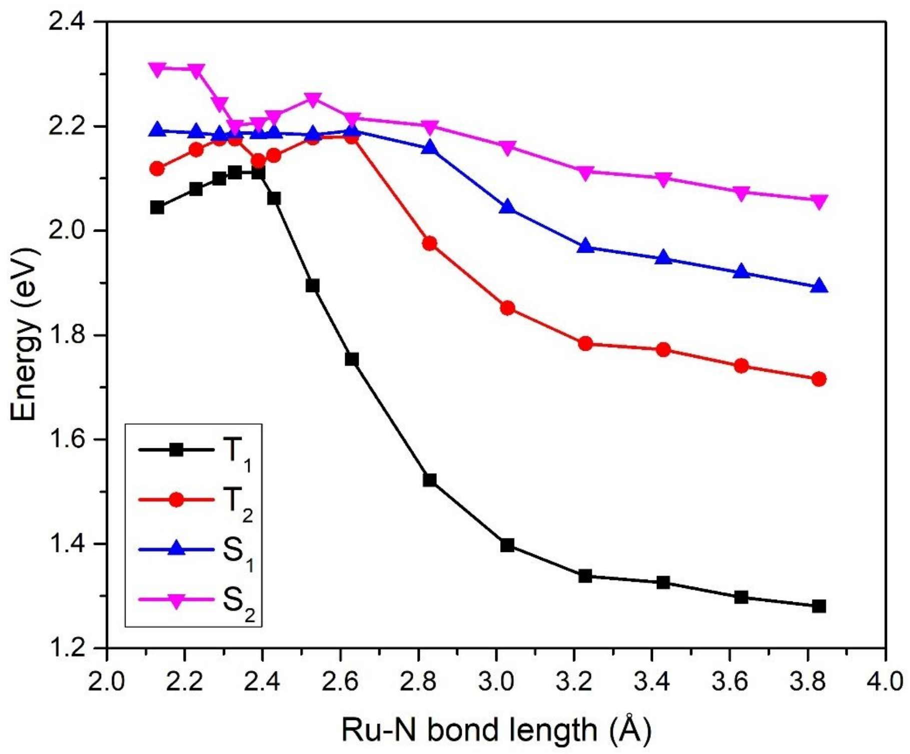

Figure S129. T_1, T_2, S_1, and S_2 energy levels versus Ru-$N_{nitrile}$ bond length for complex **3c**.

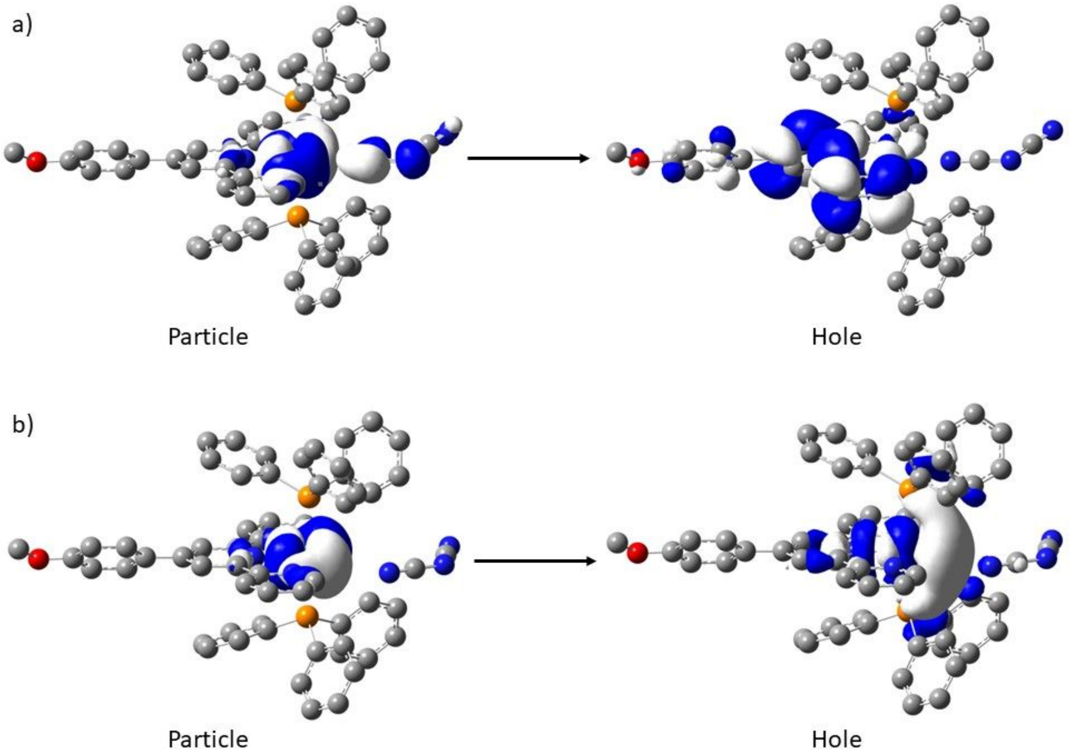

Figure S130. Plot of natural transition orbitals of **3c** for $S_0 \rightarrow T_1$ for Ru-$N_{nitrile}$ length: a) 2.12 Å and b) 3.82 Å.

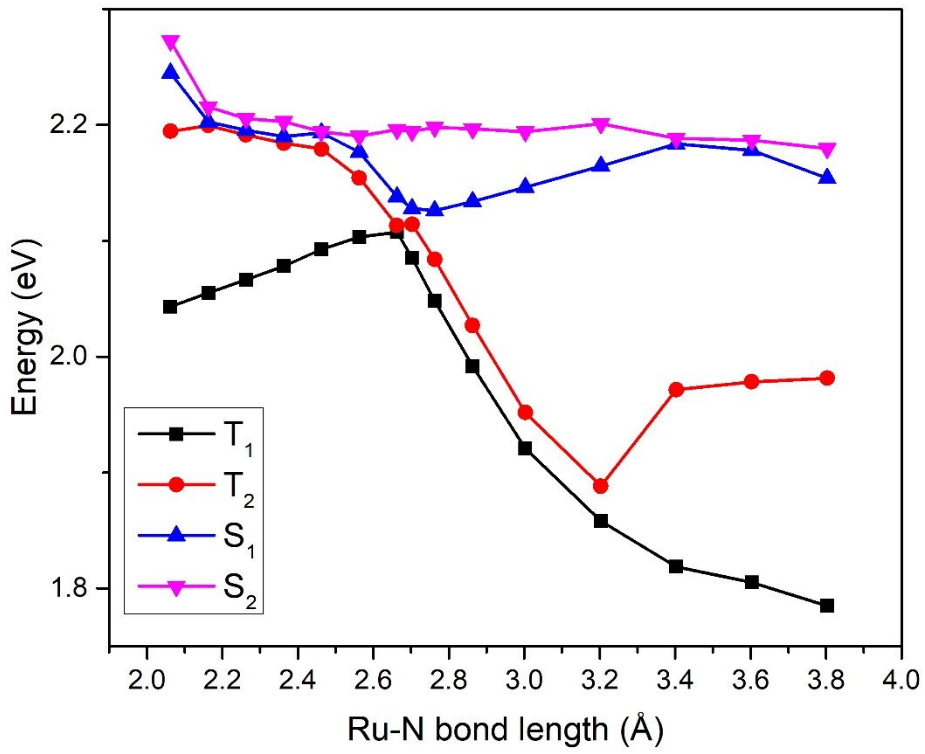

Figure S131. T_1, T_2, S_1, and S_2 energy levels versus Ru-$N_{nitrile}$ bond length for complex **4a**.

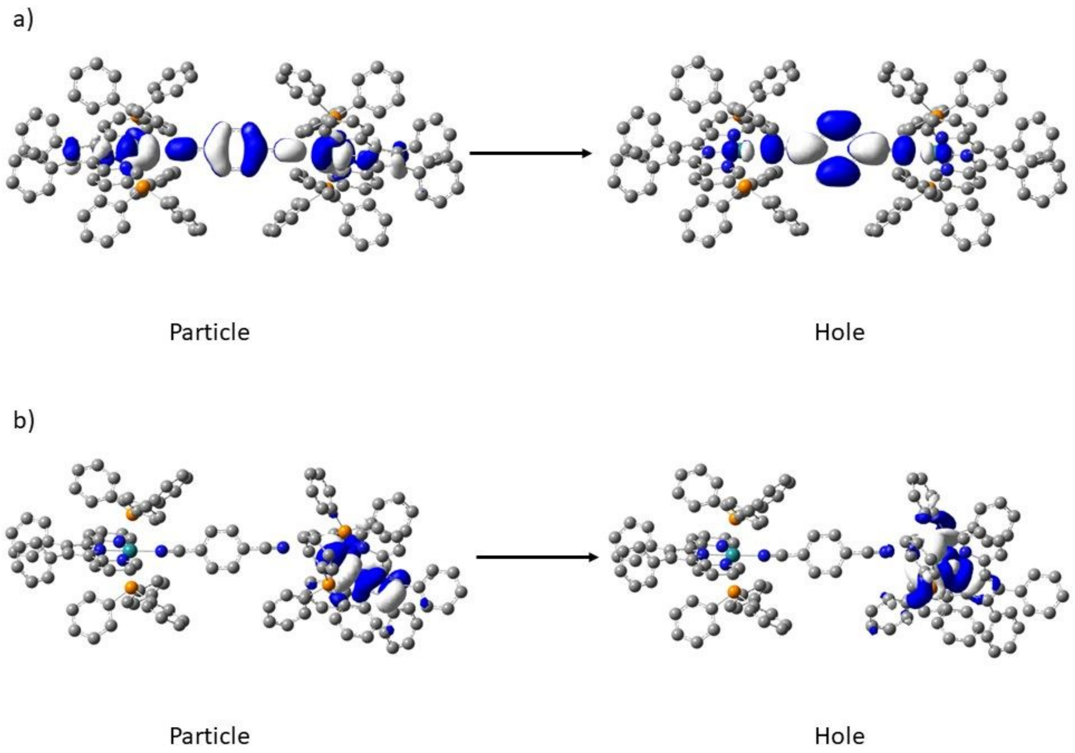

Figure S132. Plot of natural transition orbitals of **4a** for $S_0 \rightarrow T_1$ for Ru-$N_{nitrile}$ length: a) 2.06 Å and b) 3.80 Å.

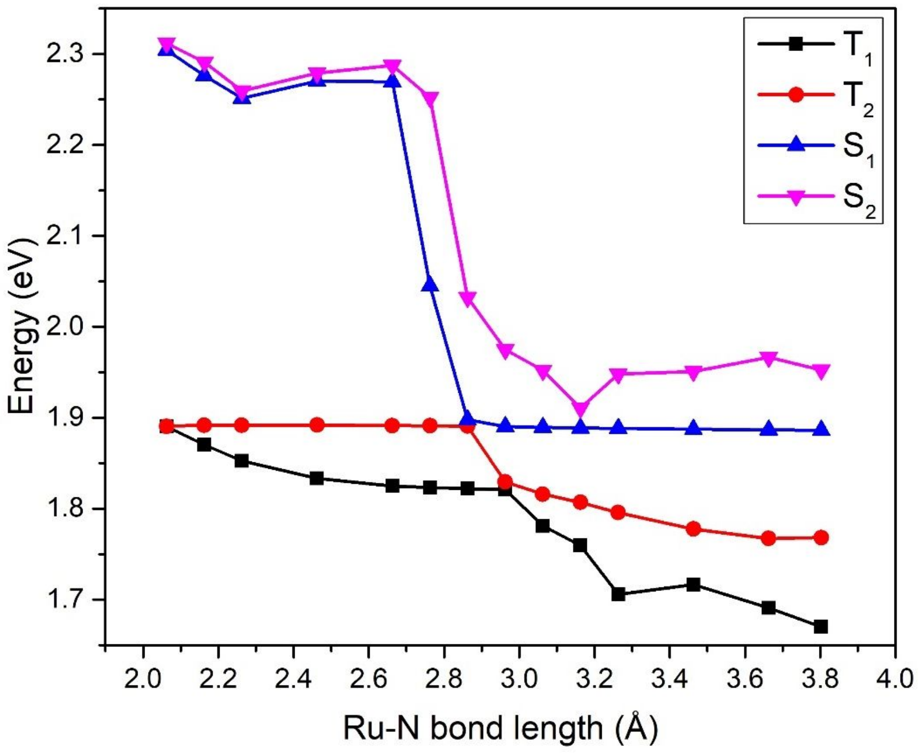

Figure S133. T_1, T_2, S_1, and S_2 energy levels versus Ru-$N_{nitrile}$ bond length for complex **4b**.

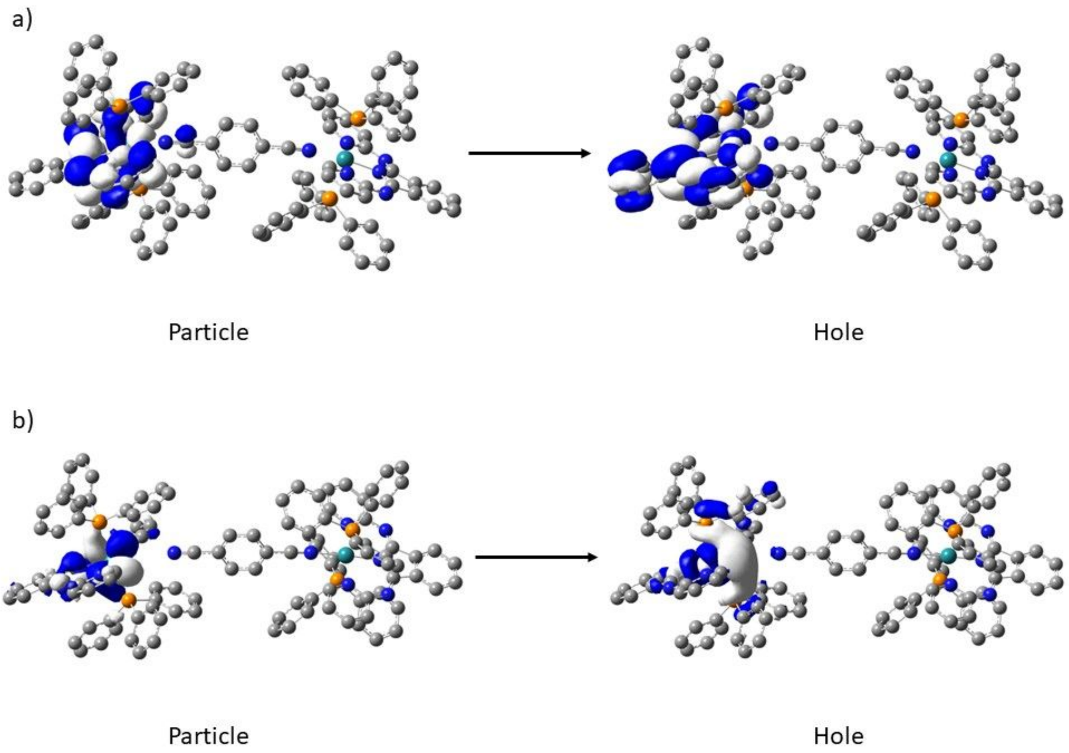

Figure S134. Plot of natural transition orbitals of **4b** for $S_0 \rightarrow T_1$ for Ru-N$_{nitrile}$ length: a) 2.06 Å and b) 3.80 Å.

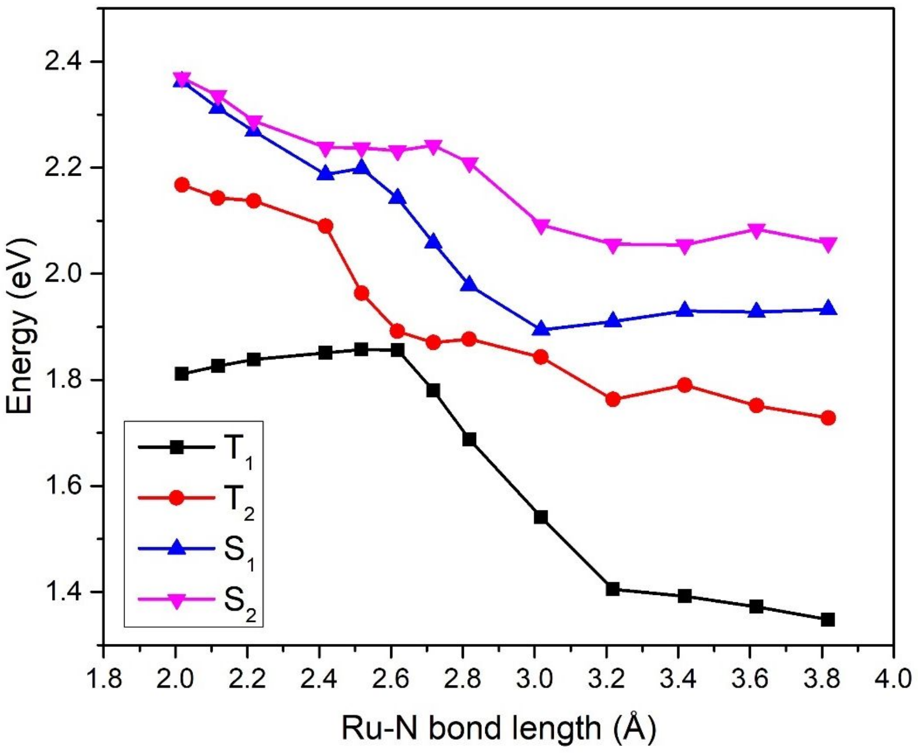

Figure S135. T_1, T_2, S_1, and S_2 energy levels versus Ru-N$_{nitrile}$ bond length for complex **4c**.

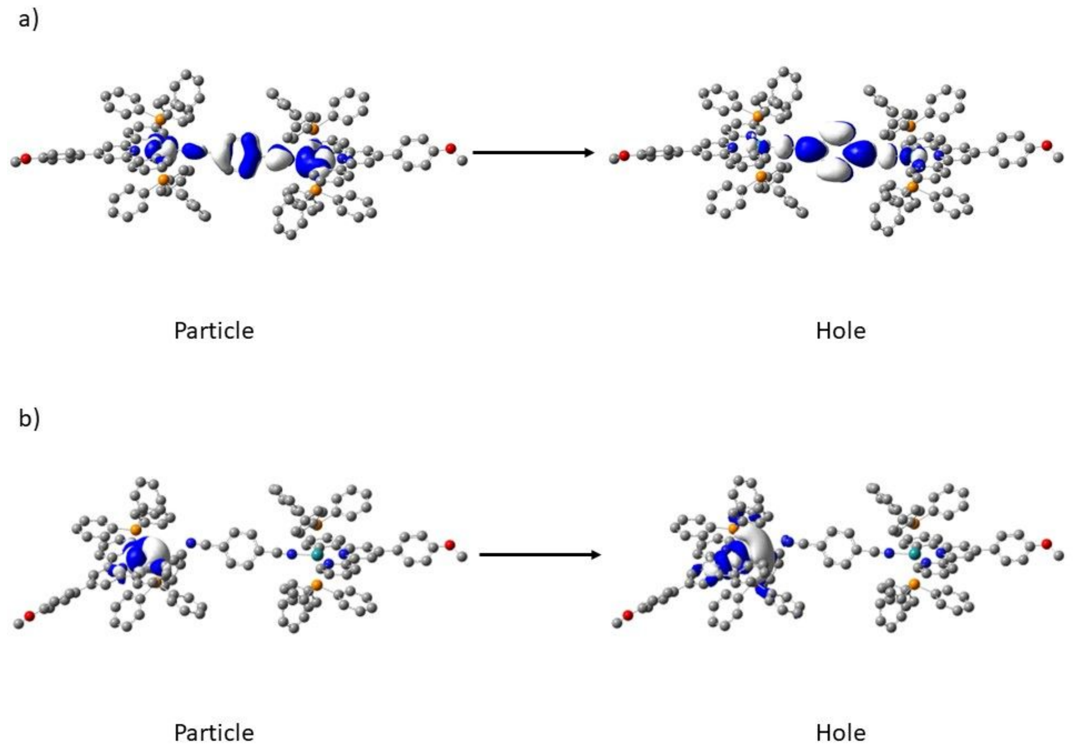

Figure S136. Plot of natural transition orbitals of **4c** for $S_0 \rightarrow T_1$ for Ru-$N_{nitrile}$ length: a) 2.01 Å and b) 3.81 Å.

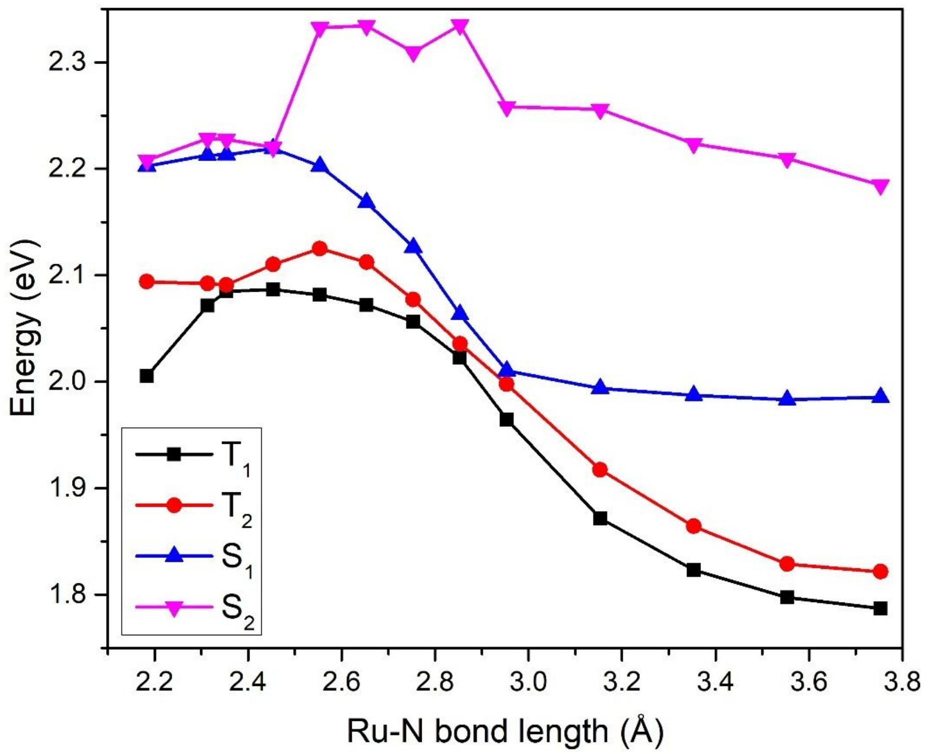

Figure S137. T_1, T_2, S_1, and S_2 energy levels versus Ru-$N_{nitrile}$ bond length for complex **5a**.

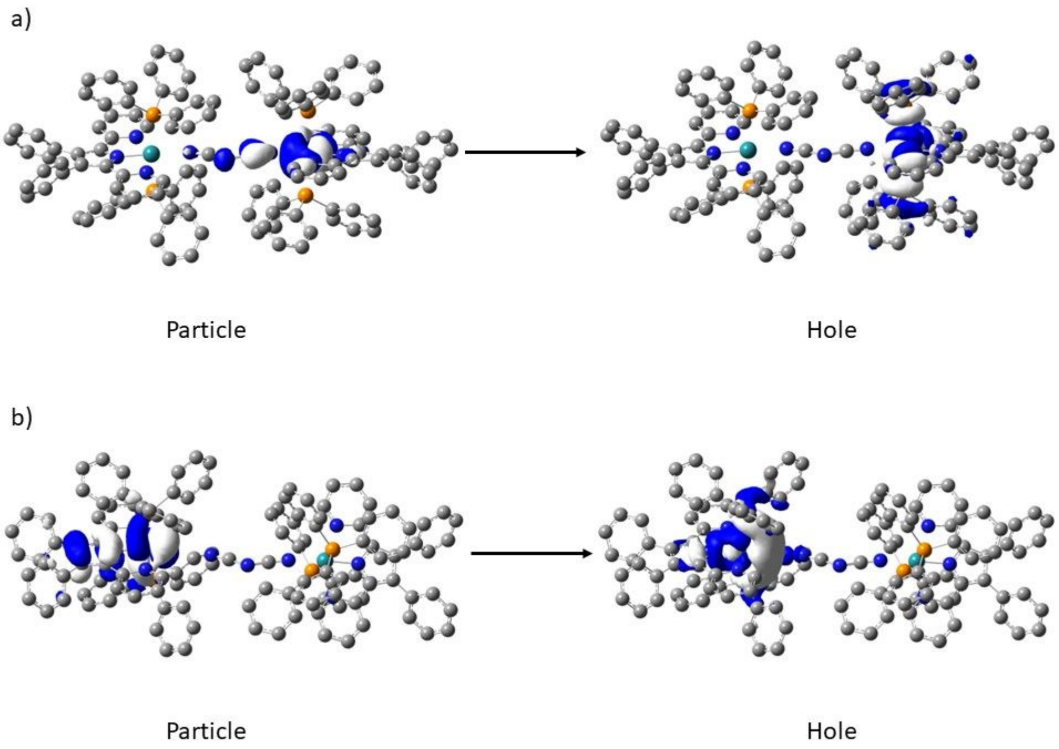

Figure S138. Plot of natural transition orbitals of **5a** for $S_0 \rightarrow T_1$ for Ru-$N_{nitrile}$ length: a) 2.18 Å and b) 3.75 Å.

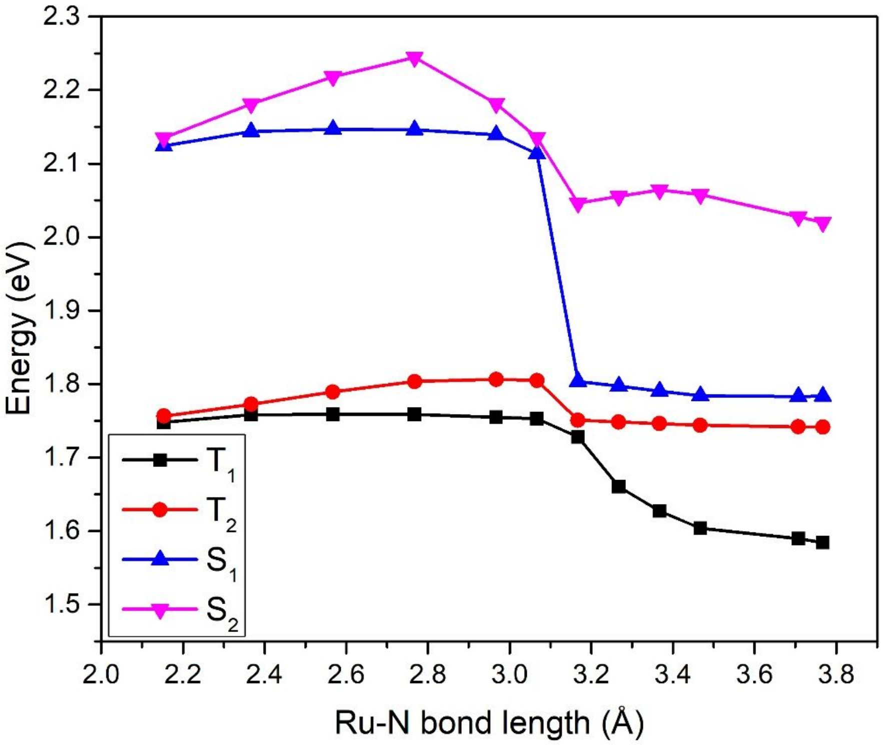

Figure S139. T_1, T_2, S_1, and S_2 energy levels versus Ru-$N_{nitrile}$ bond length for complex **5b**.

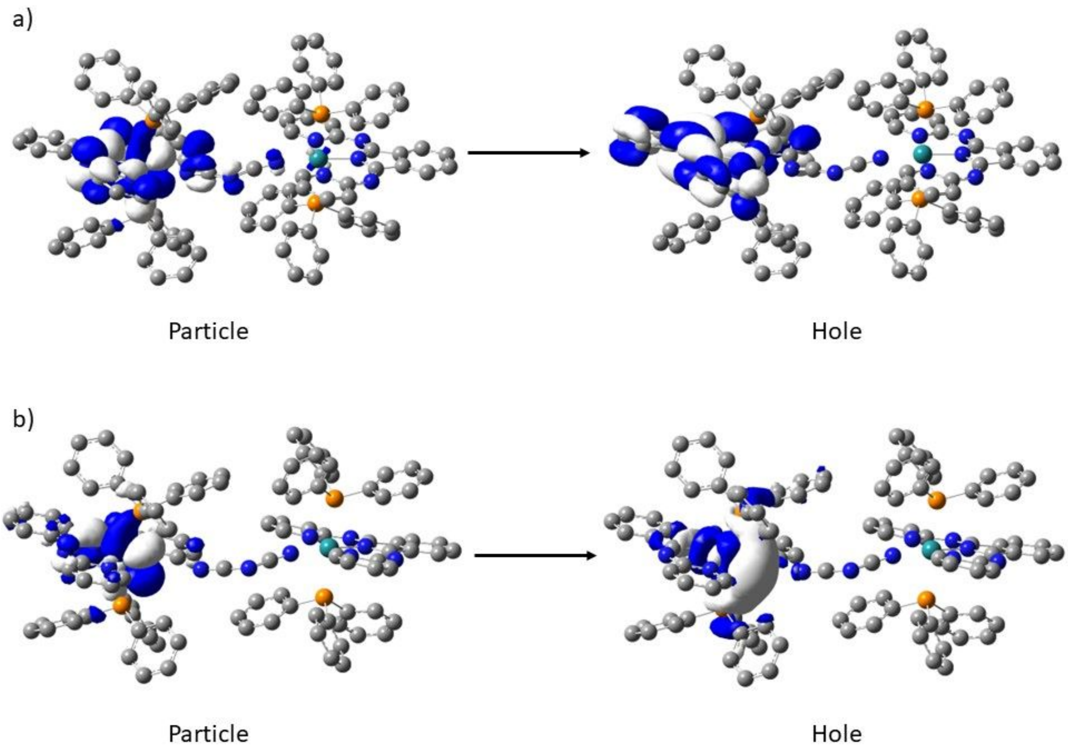

Figure S140. Plot of natural transition orbitals of **5b** for $S_0 \rightarrow T_1$ for Ru-$N_{nitrile}$ length: a) 2.15 Å and b) 3.76 Å.

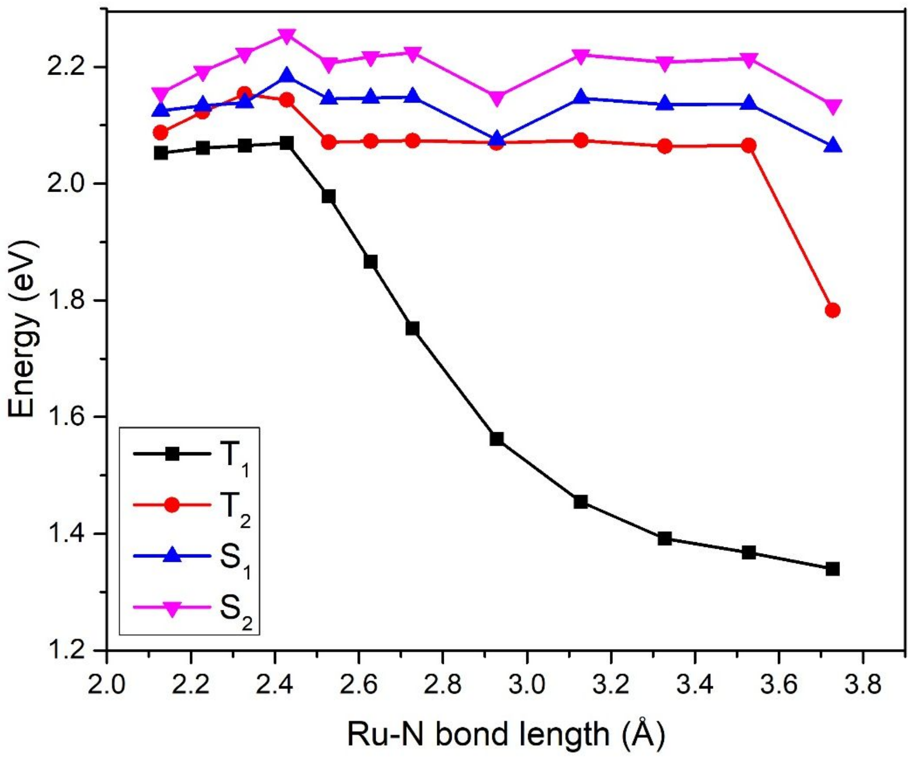

Figure S141. T_1, T_2, S_1, and S_2 energy levels versus Ru-$N_{nitrile}$ bond length for complex **5c**.

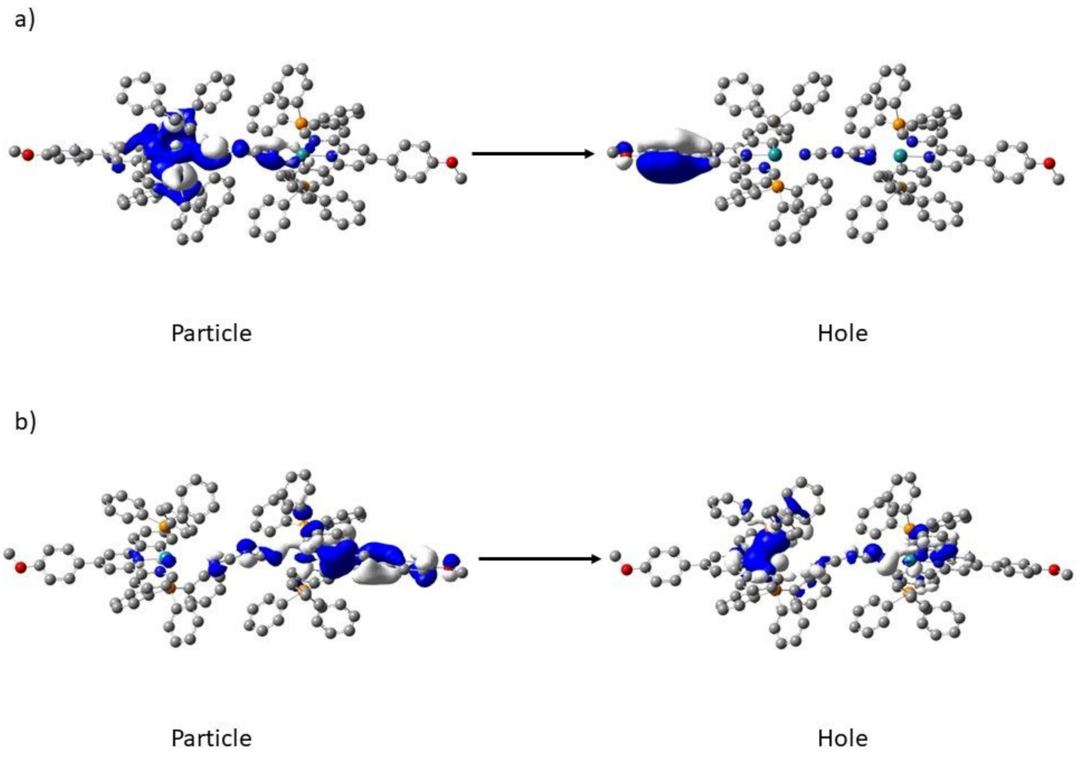

Figure S142. Plot of natural transition orbitals of **5c** for $S_0 \rightarrow T_1$ for Ru-$N_{nitrile}$ length: a) 2.12 Å and b) 3.72 Å.

S6. Photodissociation Products

In order to establish the photodissociation products from each of the complexes a comparison was made between the TD-DFT calculated spectra of the parent complex and proposed products. Under the conditions of the measurements it is proposed that either the respective chloride complexes (Cla, Clb, or Clc) or acetonitrile complexes (CH_3CN-a, CH_3CN-b, or CH_3CN-c) were formed.

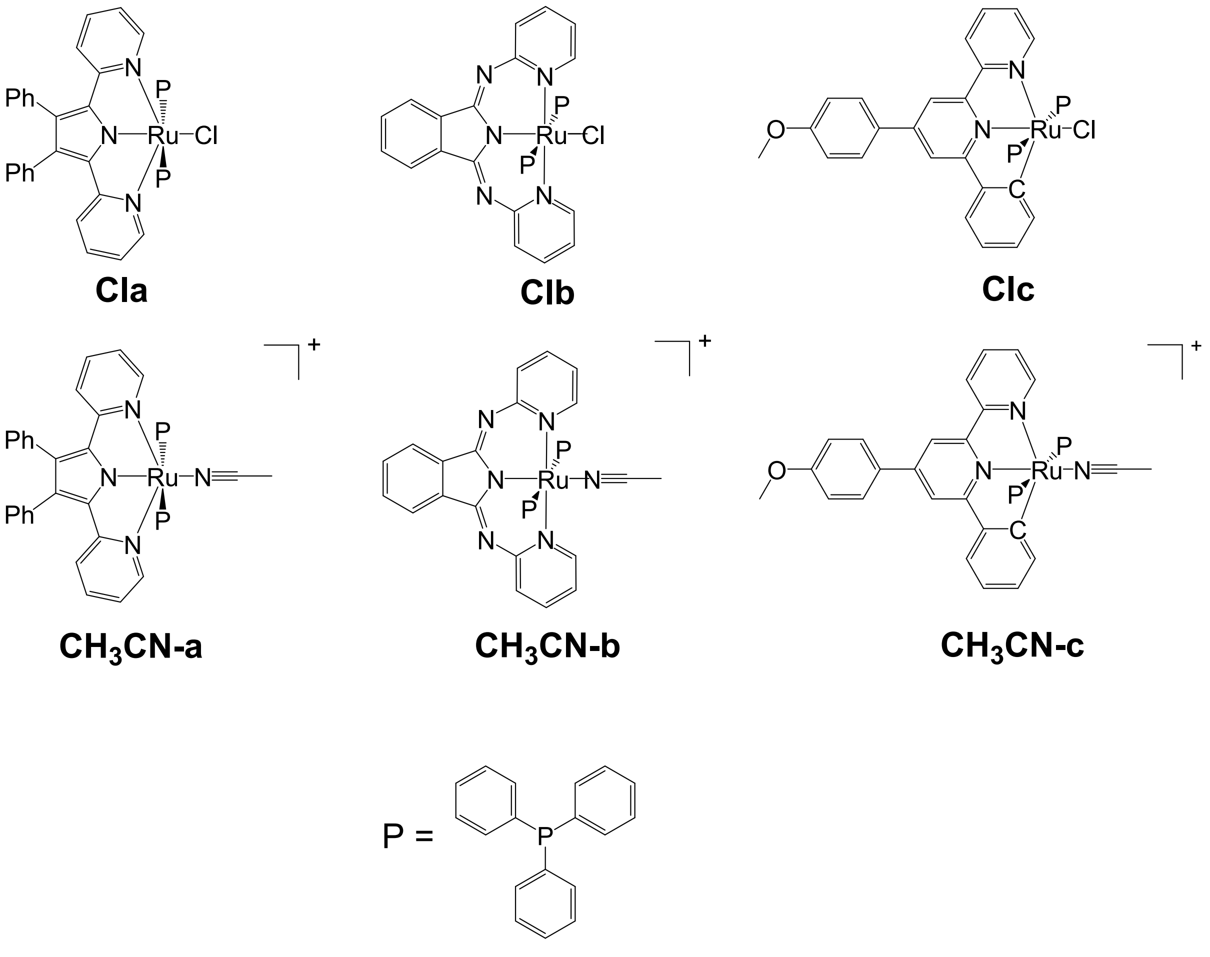

Figure S143. Proposed structures for the photodissociation products.

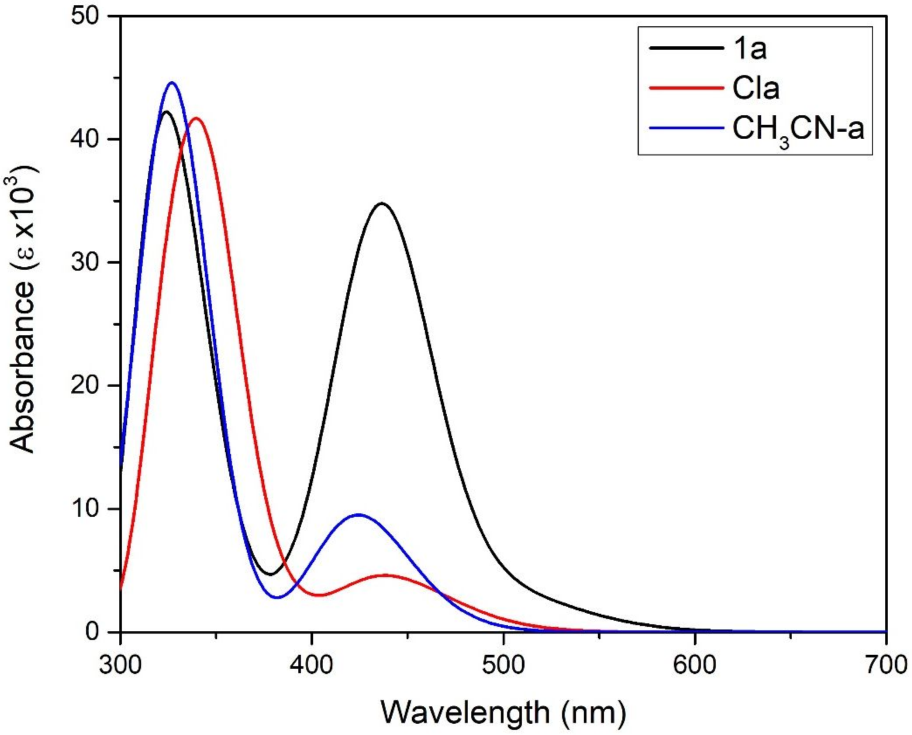

Figure S144. TD-DFT calculated absorbance spectra of **1a**, **Cla** and **CH_3CN-a**.

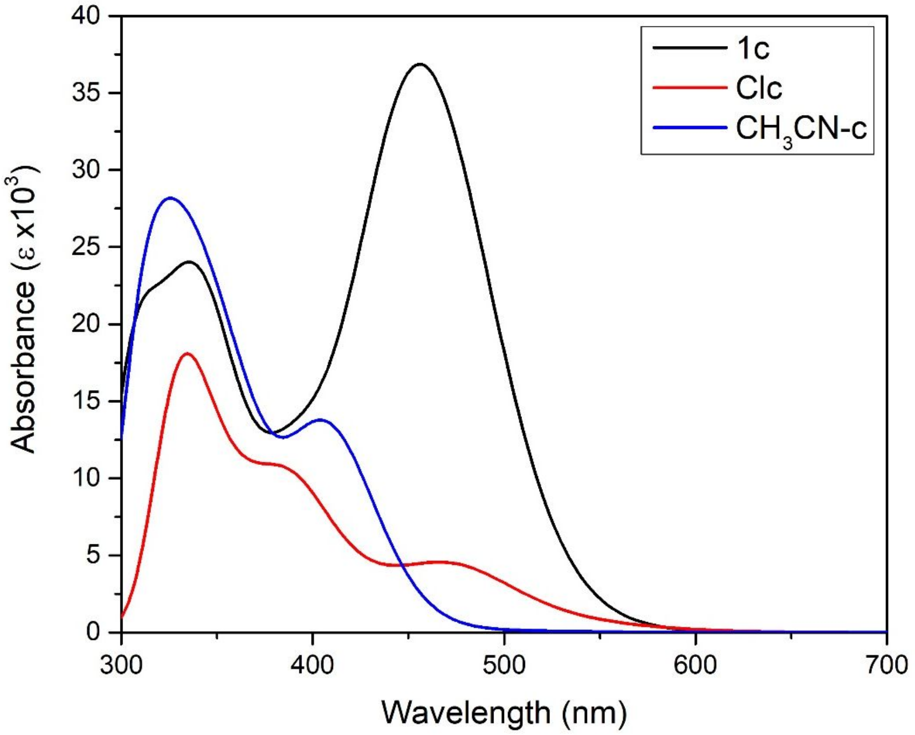

Figure S145. TD-DFT calculated absorbance spectra of **1c**, **Clc** and **CH_3CN-c**.

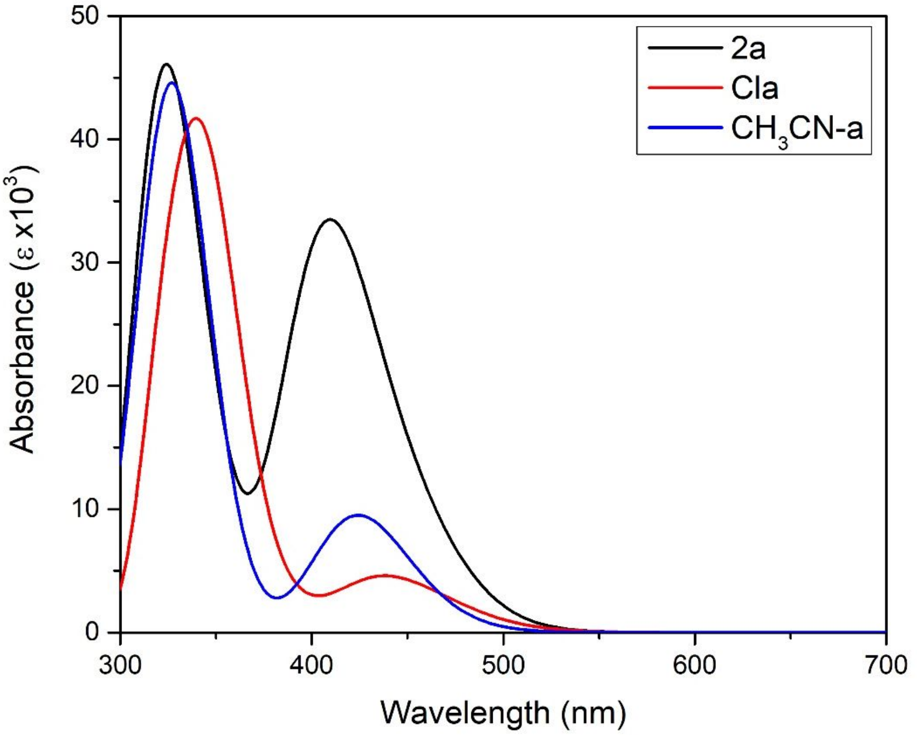

Figure S146. TD-DFT calculated absorbance spectra of **2a**, **Cla** and **CH_3CN-a**.

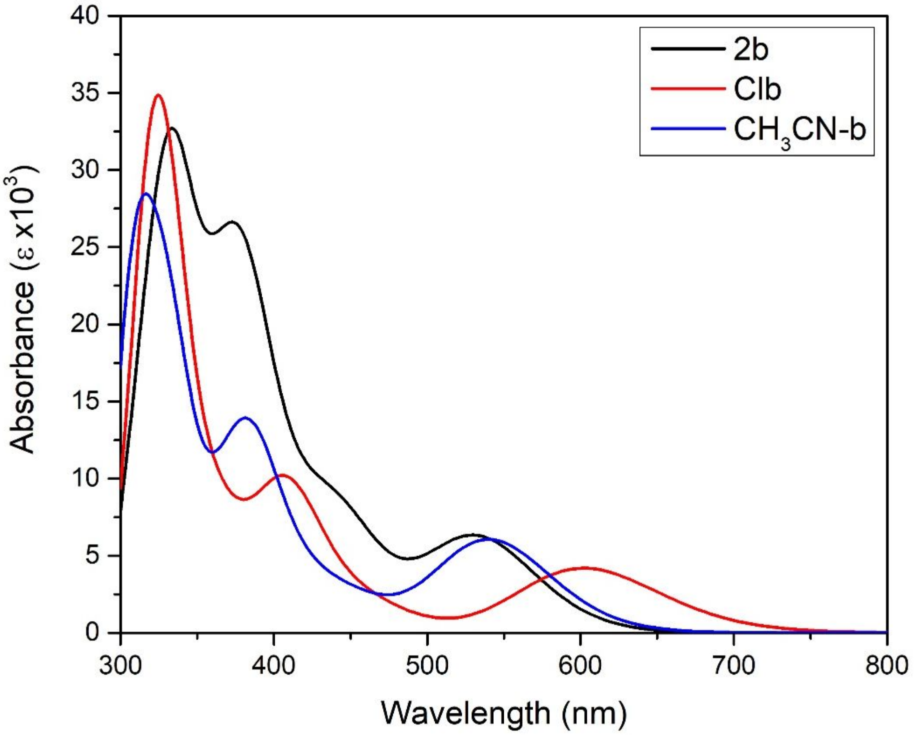

Figure S147. TD-DFT calculated absorbance spectra of **2b**, **Clb** and **CH_3CN-b**.

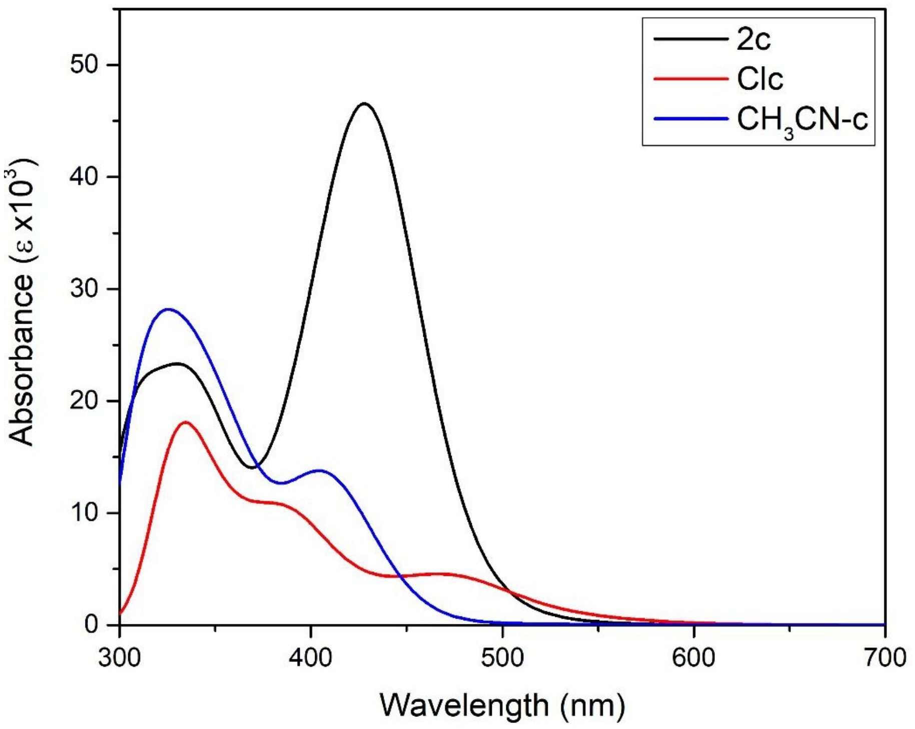

Figure S148. TD-DFT calculated absorbance spectra of **2c**, **Clc** and **CH_3CN-c**.

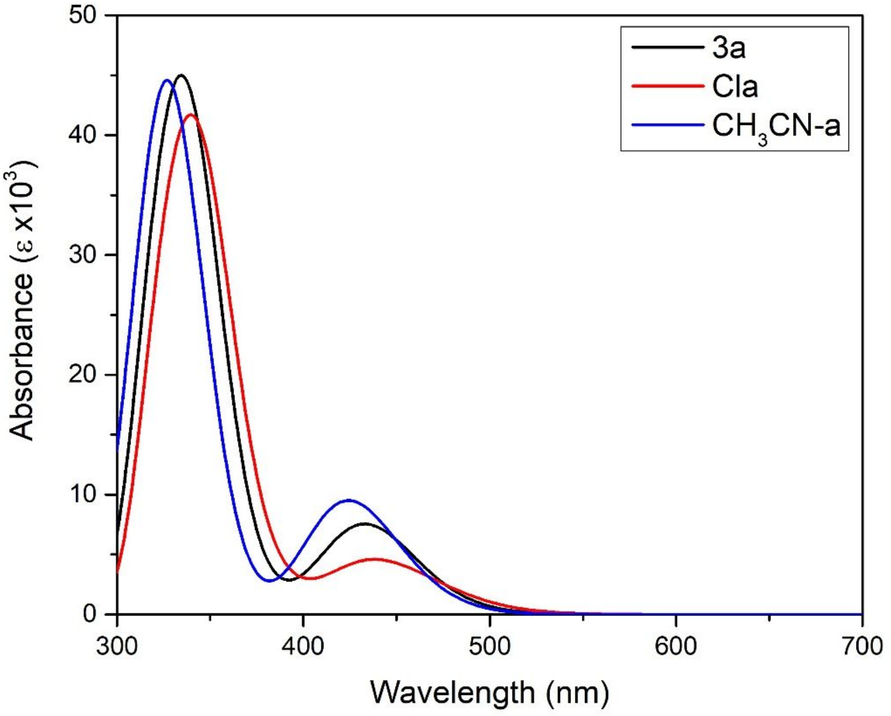

Figure S149. TD-DFT calculated absorbance spectra of **3a**, **Cla** and **CH_3CN-a**.

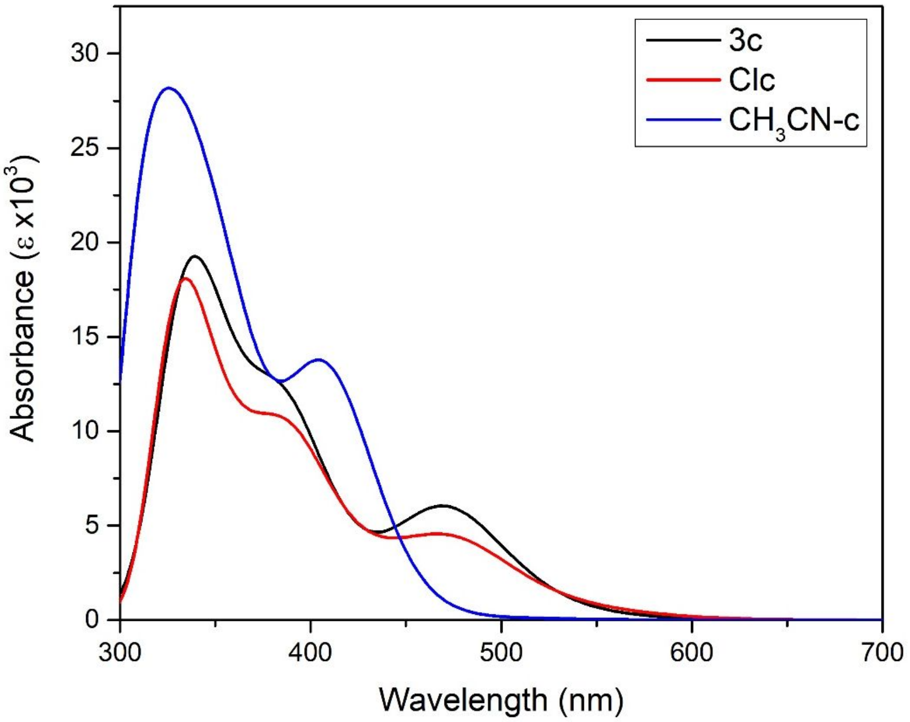

Figure S150. TD-DFT calculated absorbance spectra of **3c**, **Clc** and **CH_3CN-c**.

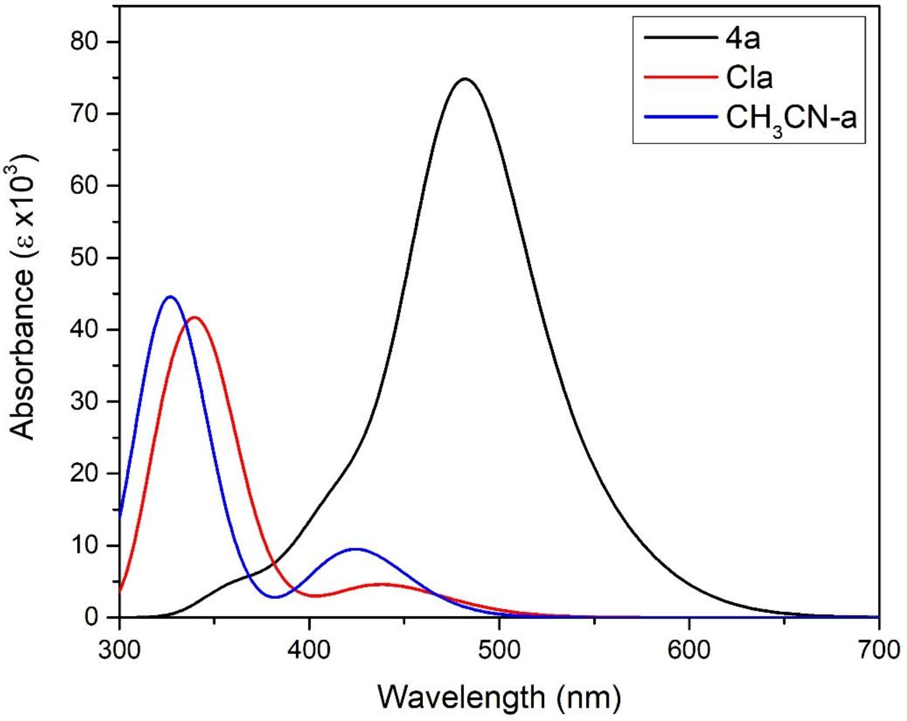

Figure S151. TD-DFT calculated absorbance spectra of **4a**, **Cla** and **CH_3CN-a**.

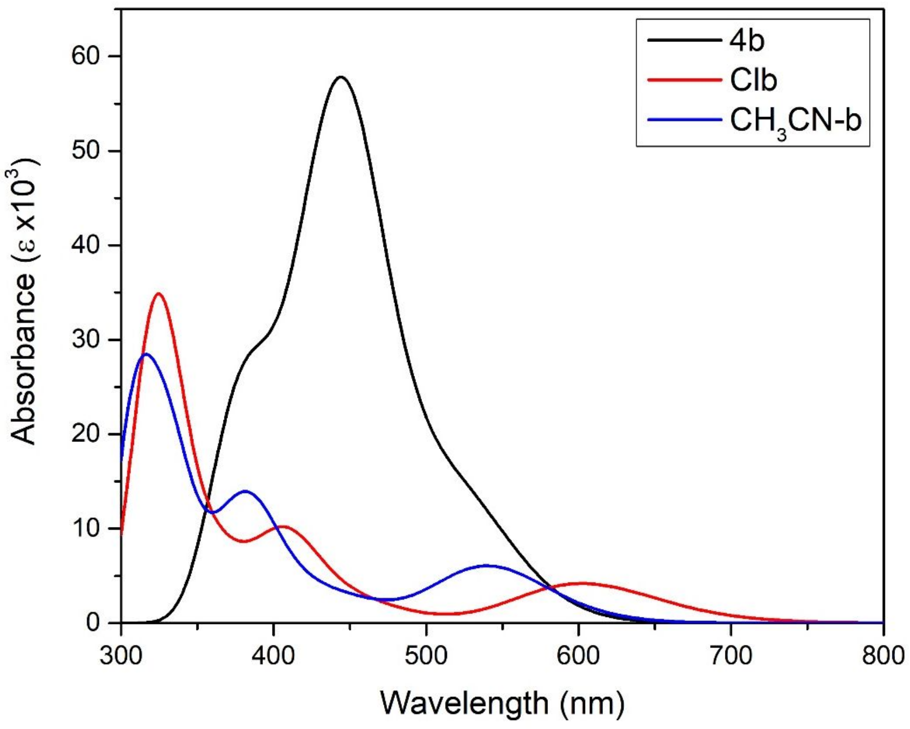

Figure S152. TD-DFT calculated absorbance spectra of **4b**, **Clb** and **CH_3CN-b**.

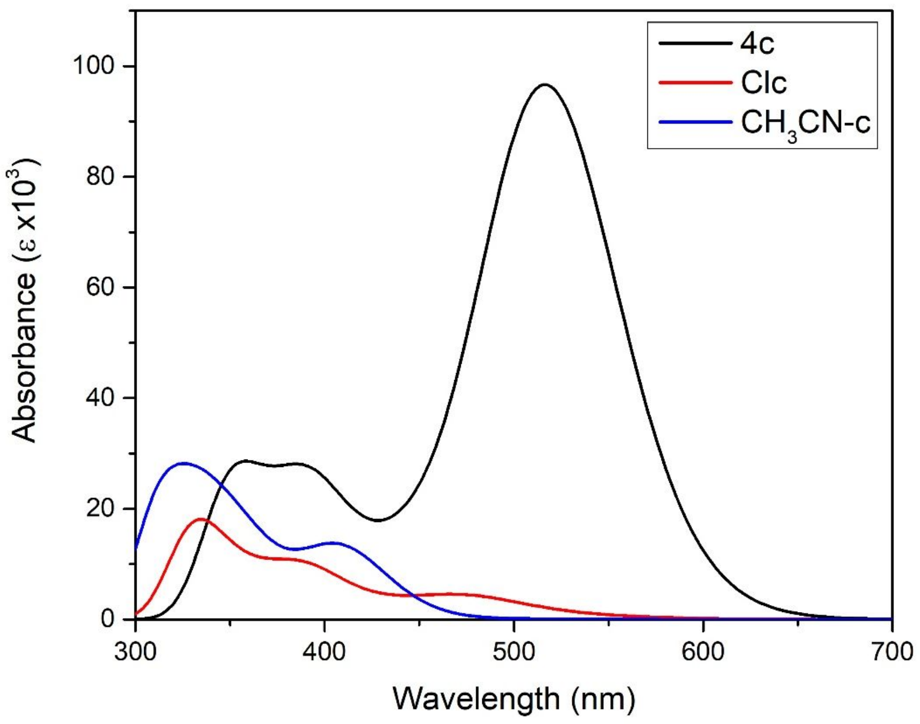

Figure S153. TD-DFT calculated absorbance spectra of **4c**, **Clc** and **CH_3CN-c**.

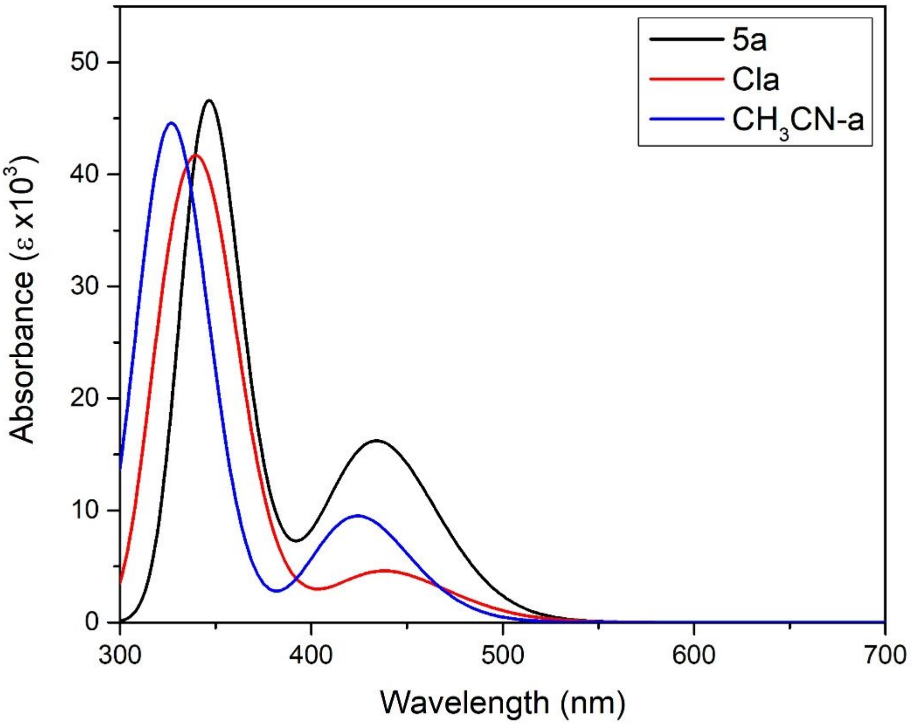

Figure S154. TD-DFT calculated absorbance spectra of **5a**, **Cla** and **CH_3CN-a**.

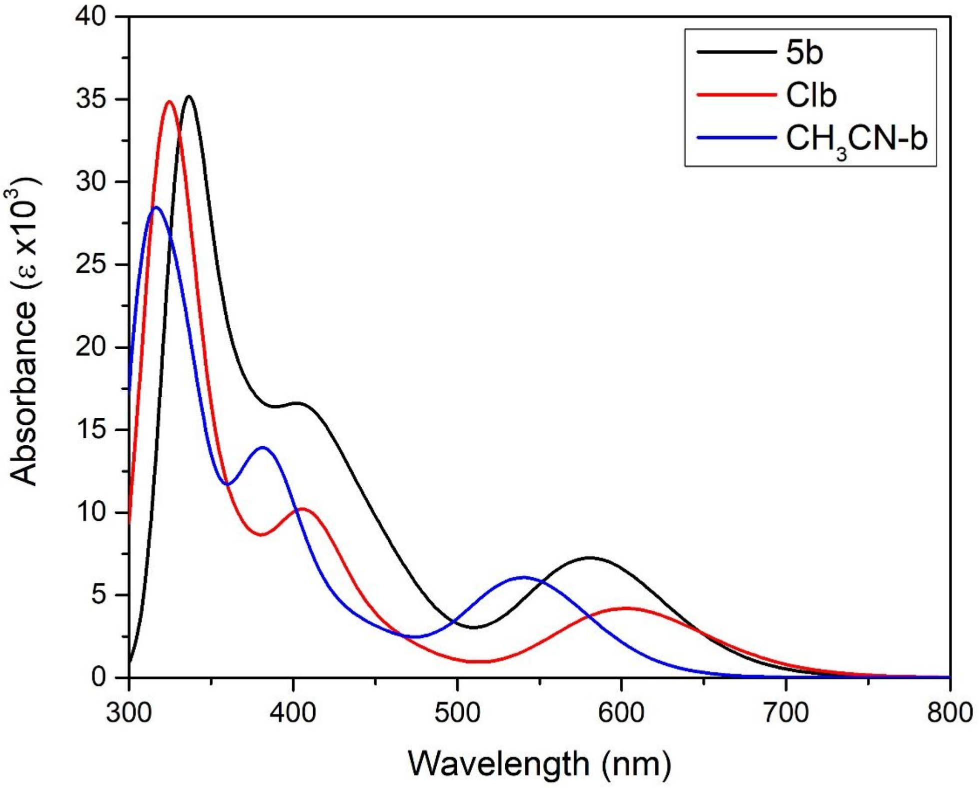

Figure S155. TD-DFT calculated absorbance spectra of **5b**, **Clb** and **CH_3CN-b**.

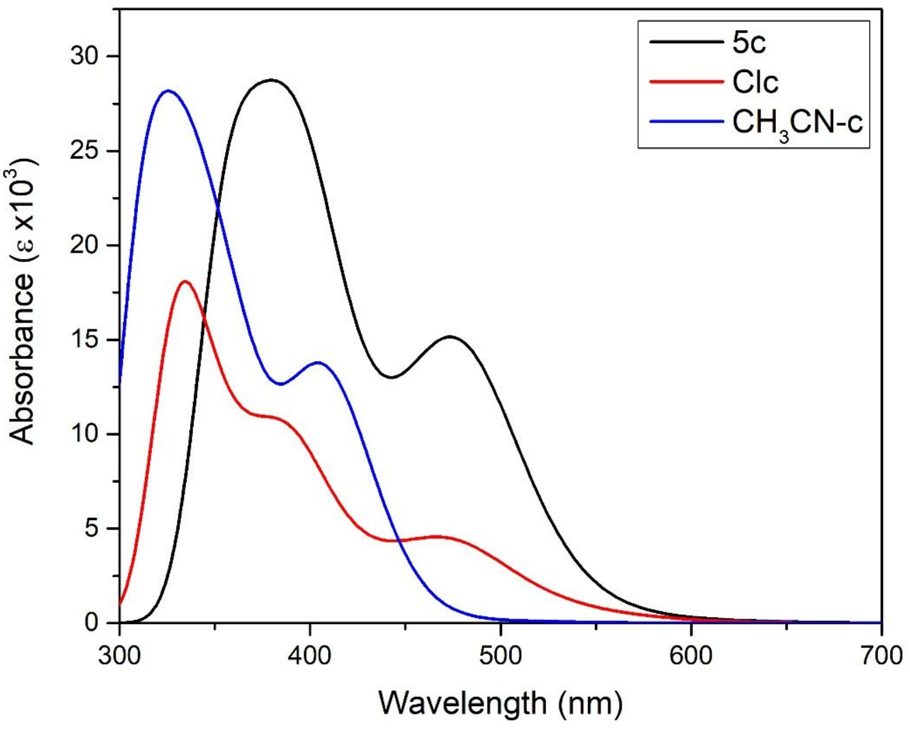

Figure S156. TD-DFT calculated absorbance spectra of **5c**, **Clc** and **CH_3CN-c**.

REFERENCES

1. Dolomanov, O. V.; Bourhis, L. J.; Gildea, R. J.; Howard, J. A. K.; Puschmann, H. OLEX2: a complete structure solution, refinement and analysis program. *Journal of Applied Crystallography* **2009,** 42, 339-341 DOI: 10.1107/s0021889808042726.

2. Sheldrick, G. M. *SHELXL: Suite of Programs for Crystal Structure Analysis*, Tammanstrasse 4: Gottingen, 1998.
